# Sequence-Defined Heteromultivalent Precision Glycomacromolecules Bearing Sulfonated/Sulfated Nonglycosidic Moieties Preferentially Bind Galectin-3 and Delay Wound Healing of a Galectin-3 Positive Tumor Cell Line in an In Vitro Wound Scratch Assay

**DOI:** 10.1002/mabi.202000163

**Published:** 2020-07-26

**Authors:** Tanja Freichel, Viktoria Heine, Dominic Laaf, Eleanor E. Mackintosh, Sophia Sarafova, Lothar Elling, Nicole L. Snyder, Laura Hartmann

**Affiliations:** Institute of Organic and Macromolecular Chemistry, Heinrich-Heine University Düsseldorf, Universitätsstraße 1, Düsseldorf 40225, Germany; Laboratory for Biomaterials, Institute for Biotechnology and Helmholtz-Institute for Biomedical Engineering, RWTH Aachen University, Pauwelsstr. 20, Aachen 52074, Germany; Laboratory for Biomaterials, Institute for Biotechnology and Helmholtz-Institute for Biomedical Engineering, RWTH Aachen University, Pauwelsstr. 20, Aachen 52074, Germany; Department of Chemistry, Davidson College, Box 7120, Davidson, NC 28035, USA; Department of Biology, Davidson College, Box 7188, Davidson, NC 28035, USA; Laboratory for Biomaterials, Institute for Biotechnology and Helmholtz-Institute for Biomedical Engineering, RWTH Aachen University, Pauwelsstr. 20, Aachen 52074, Germany; Department of Chemistry, Davidson College, Box 7120, Davidson, NC 28035, USA; Institute of Organic and Macromolecular Chemistry Heinrich-Heine University Düsseldorf Universitätsstraße 1, Düsseldorf 40225, Germany

**Keywords:** galectins, glycomimetics, heteromultivalency, in vitro wound scratch assay, solid phase synthesis

## Abstract

Within this work, a new class of sequence-defined heteromultivalent glycomacromolecules bearing lactose residues and nonglycosidic motifs for probing glycoconjugate recognition in carbohydrate recognition domain (CRD) of galectin-3 is presented. Galectins, a family of β-galactoside-binding proteins, are known to play crucial roles in different signaling pathways involved in tumor biology. Thus, research has focused on the design and synthesis of galectin-targeting ligands for use as diagnostic markers or potential therapeutics. Heteromultivalent precision glycomacromolecules have the potential to serve as ligands for galectins. In this work, multivalency and the introduction of nonglycosidic motifs bearing either neutral, amine, or sulfonated/sulfated groups are used to better understand binding in the galectin-3 CRD. Enzyme-linked immunosorbent assays and surface plasmon resonance studies are performed, revealing a positive impact of the sulfonated/sulfated nonglycosidic motifs on galectin-3 binding but not on galectin-1 binding. Selected compounds are then tested with galectin-3 positive MCF 7 breast cancer cells using an in vitro would scratch assay. Preliminary results demonstrate a differential biological effect on MCF 7 cells with high galectin-3 expression in comparison to an HEK 293 control with low galectin-3 expression, indicating the potential for sulfonated/sulfated heteromultivalent glycomacromolecules to serve as preferential ligands for galectin-3 targeting.

## 1. Introduction

Many processes in tumorigenesis are the result of dysregulated protein expression and the presentation of abnormal glycan motifs on the cell surface.^[1-3]^ One family of proteins known to be involved in tumor biology is the galectins. Galectins consist of a conserved carbohydrate recognition domain that is known to bind β-galactoside terminating glycans such as those terminating in lactose (Lac) or poly *N*-acetyl-lactosamine (LacNAc). Galectin-3 is the only chimeric galectin within the galectin family, and contains a proline-rich *N*-terminal domain which can self-oligomerize into pentameric lectin lattices.^[4-7]^ Galectin-3 is normally found in the nucleus and cytoplasm, but can also be secreted and can interact with glycoproteins on cell surfaces. Galectin-3 has been shown to play both positive and negative roles in tumor metastasis and migration.^[2,8-17]^ In cases where galectin-3 has been shown to be responsible for tumor metastasis and migration, several groups have endeavored to design compounds to inhibit them. One group of examples involves the multivalent presentation of carbohydrate residues on macromolecular scaffolds as described by Gabius et al.,^[18,19]^ Roy et al.,^[20,21]^ Nilsson et al.,^[22,23]^ Wang et al.,^[24]^ Argueso et al.,^[25]^ Cloninger et al.,^[26,27]^ Lecommandoux et al.,^[28]^ Elling and co-workers,^[29-31]^ Percec et al.,^[32-35]^ Putnam et al.,^[36]^ and more recently Rosencrantz et al.^[37]^ This approach takes advantage of the ability of galectin-3 to oligomerize in the presence of multivalent ligands leading to an effective increase in binding avidity.^[18-35,38]^

Other studies using monovalent carbohydrates have revealed that the introduction of nonglycosidic moieties can enhance the affinity of ligands targeting galectin-3. For example, Nilsson and co-workers have demonstrated that galactose monosaccharides functioned with aromatic moieties at the 3-*O* position can be selective, potent inhibitors of galectin-3,^[39-47^] and that 3-substituted oxazoles could provide selectivity for galectin-3 over galectin-1.^[48]^ In addition, thiodigalactoside analogs substituted with hydrophobic groups at the 3-positions have shown significant selectivity and binding to galectin-3 in the nm range.^[47-56]^ For example, TD139, is currently in clinical trials for treating idiopathic pulmonary fibrosis.^[57]^

Several groups have also demonstrated that sulfonation/sulfation can be used to alter the affinity and selectivity of ligands for galectin-3.^[7,34,58-62]^ For example, Nilsson and co-workers revealed that compounds that combine sulfated glycosides and nonglycosidic moieties increased binding to galectin-3 from a *K*_d_ value of 5900 × 10^−6^ m for an unsubstituted methyl galactoside, to 2800 × 10^−6^ m for the 2-*O*-sulfated and 87 × 10^−6^ m for the 3-*O*-methylbenzamido- and 2-*O*-sulfated derivatives.^[63,64]^

The aforementioned studies and others^[65-68]^ inspired us to combine the concepts of multivalency, the incorporation of nonglycosidic moieties and sulfation to develop a series of heteromultivalent glycomacromolecules for targeting galectin-3. Unlike previous studies, these novel compounds were specifically designed to compare how neutral, amine, and sulfonated/sulfated nonglycosidic aromatic moieties impact galectin binding. Herein, we report the solid phase polymer synthesis (SPPoS) of a library of sequence-defined lactose-based glycooligo(amidoamines) bearing nonglycosidic, sulfonated, and sulfated moieties as potential ligands for galectin-3. The resulting glycomacromolecules were evaluated for binding to galectin-3 using an enzyme-linked immunosorbent assay (ELISA) and compared to galectin-1. Results from ELISA studies revealed that glycomacromolecules bearing lactose and aryl sulfonate/sulfate motifs preferentially bound galectin-3 over galectin-1. Binding studies with galectin-3 were then confirmed through surface plasmon resonance (SPR) studies. The best ligands were applied to an in vitro scratch wound assay using a galectin-3 positive MCF 7 cell line revealing their ability to interfere with wound closure. To the best of our knowledge this is the first systematic approach using heteromultivalent glycomacromolecules to better understand binding in the galectin family.

## 2. Experimental Section

### 2.1. General Methods

Acetic anhydride was purchased from VWR chemicals. Piperidine, trifluoro acetic acid, sodium methoxide, *N*-Boc phenylenediamine 4-pentynoic acid, and sodium diethyldithiocarbamate were purchased from Acros Organics. Dimethylformamide (DMF; for peptide synthesis) was purchased from Biosolve. 3-Amino-4-hydroxy benzene-sulfonic acid and triisopropyl silane (TIPS) were purchased from Sigma Aldrich. 4-Amino benzene sulfonic acid was purchased from J&K, and benzylamine and oxalyl chloride from Alfa Aesar. Hydroxybenzotriazole (HOBt) was purchased from Iris Biotech. *N*,*N*-Diisopropylethylamine (DIPEA) and diethylenetriamine were purchased from Carl Roth and lithium hydroxide, sodium ascorbate, and potassium carbonate from PanReac AppliChem. Dichloromethane (DCM) and triethyleneamine were purchased from Merck. HATU (1-[bis(dimethylamino) methylene]-1*H*-1,2,3-triazolo[4,5-*b*]pyridinium 3-oxide hexafluorophosphate, hexafluorophosphate azabenzotriazole tetramethyl uronium) was purchased from Abcr. PyBOP (benzotriazol-1-yl-oxytripyrrolidinophosphonium hexafluorophosphate) was purchased from Fluorochem and CuSO_4_ anhydrous from Fluka Chemika. Fmoc-L-Tyr(4-SO_3_H)-OH was purchased from Iris Biotech GmbH. Solid phase synthesis was performed on TentaGelSRam resin purchased from Rapp Polymere using polypropylene reactors with polyethylene frits closed with luerstoppers from MultiSyntech GmbH. Building blocks TDS (triple bond diethylenetriamine succinyl, 1-(fluorenyl)-3,11-dioxo-7-(pent-4-ynoyl)-2-oxa-4,7,10-triazatetra-decan-14-oic acid),^[69]^ EDS (ethylene glycol diamine succinyl, 1-(9*H*-fluoren-9-yl)-3,14-dioxo-2,7,10-trioxa-4,13-diazaheptadecan-17-oic acid),^[70]^ and MDS (methyl succinyl diethylenetriamine succinyl, 1-(9*H*-fluoren-9-yl)-7-(4-methoxy-4-oxobutanoyl)-3,11-dioxo-2-oxa-4,7,10-triazatetradecan-14-oic acid)^[71]^ were synthesized as reported earlier. (2-Azidoethyl)-2,3,4,6-tetra-*O*-acetyl-*β*-D-galactopyranoside, (2-Azidoethyl)-2,3,4,6-tetra-*O*-acetyl-*α*-D-glucopyranoside, and 2,3,4,6-tetra-*O*-acetyl-*α*-D-glucopyranoside were synthesized as known from literature.^[72]^ Reactions were monitored via analytical thin layer chromatography, performed on Merck silica gel 60 F254 plates and were visualized with ninhydrin staining. All reagents and solvents were used without further purification. ^1^H NMR and ^13^C NMR spectra were measured on Bruker Avance III 300 or Bruker Avance III 600. Analytic reversed phase high-performance liquid chromatography (RP-HPLC) measurements were performed on Agilent Technologies 6120 series coupled with an Agilent Quadrupol mass spectrometer. All spectra were measured with solvent A: 95% H_2_O, 5% acetonitrile (ACN), 0.1% formic acid, and solvent B: 5% H_2_O, 95% ACN, 0.1% formic acid with a gradient of 5–50% B in 30 min. Purities of the compounds were determined by the integrations of the signals given through the absorption at 214 nm. Preparative RP-HPLC was performed on an Agilent 1200 series. High-resolution electrospray ionization (HR-ESI) spectra were measured on UHR-QTOF maXis 4G (Bruker Daltonics).

### 2.2. Solid Phase Synthesis

General protocols for the solid phase synthesis were described for batch sizes of 0.1 mmol resin. All reactions were performed at room temperature in a polypropylene syringe reactor with a frit on a shaker.

#### 2.2.1. Resin Preparation and Fmoc Cleavage

0.1 mmol resin (800 mg, resin loading 0.25 mmol g^−1^,) was transferred into a 10 mL reactor and 5 mL DCM was added to swell the resin for 1 h. After washing the resin ten times with 5 mL DMF, Fmoc was cleaved by adding 5 mL of 25% piperidine in DMF three times for 10 min. In between the deprotection steps, the resin was washed three times with 5 mL DMF, and after the last deprotection, the resin was washed ten times with 5 mL DMF.

#### 2.2.2. Building Block and Amino Acid Coupling

0.5 mmol building block (5 eq) and 260 mg PyBOP (0.5 mmol, 5 eq) were dissolved in 3 mL DMF, and 0.2 mL DIPEA (1 mmol, 10 eq) was added. After flushing the solution with nitrogen for 1 min, the solution was added to the resin and the reaction was shaken for 1–1.5 h. After that, the liquid content was discarded, and the resin was washed ten times with 5 mL DMF.

#### 2.2.3. Terminal-NH_2_ Capping

The resin was treated with 3 mL acetic anhydride two times for 15 min. In between, the resin was washed with DMF. After the last capping step, the resin was washed five times with 5 mL MeOH and five times with 5 mL DMF.

#### 2.2.4. Deprotection of Carboxylic Side Chain^[71]^

For conditioning, the resin was washed ten times with 5 mL of tetrahydrofuran (THF)/H_2_O (1/1). For deprotection, the resin was treated two times for 1 h with 5 mL 0.2 m LiOH in THF/H_2_O (1/1). In between, the resin was washed three times with 5 mL THF/H_2_O (1/1). After deprotection, the resin was washed alternately five times with each 5 mL of H_2_O, DMF, and DCM.

#### 2.2.5. Carbohydrate Conjugation-CuAAC

Azido carbohydrate derivatives (3 eq/alkyne group) were dissolved in 2 mL DMF. Separately, CuSO_4_ (50 mol%/alkyne) and sodium ascorbate (50 mol%/alkyne) were each dissolved in 0.2 mL MilliQ water. The carbohydrate solution was first added to the resin, followed by sodium ascorbate and CuSO_4_. After shaking the reaction mixture overnight, the resin was washed sequentially with 5 mL of DMF, a solution of 0.2 m sodium diethyldithiocarbamate in DMF and water (1/1), and water, DMF, and DCM until no more color changes were observed after the treatment with the diethyldithiocarbamate solution.

#### 2.2.6. Side Chain Coupling

F.1 Coupling with HATU: 0.6 mmol of the amine residue (3 eq/acid) and 0.6 mmol HATU (3 eq/acid) were each dissolved in 1.5 mL DMF. 0.4 mL DIPEA (2 mmol, 10 eq/acid) was added to HATU and the reaction mixture was added to the resin. After a 15 min preactivation of the resin, the amine was added and the reaction was shaken for 1.5 h. F.2 Coupling with PyBOP: 0.6 mmol of the amine residue (3 eq/acid) was dissolved in 1.5 mL DMF/DCM (1/1). 0.6 mmol PyBOP (3 eq/acid) and 0.6 mmol HOBt (3 eq/acid) were dissolved in 1.5 mL DMF/DCM (1/1) and 0.4 mL DIPEA (2 mmol, 10 eq/acid) was added. The reaction mixture was added to the resin, and after a 15 min preactivation, the amine was added and the reaction was shaken for 1.5 h.

#### 2.2.7. Carbohydrate Deprotection

The resin was treated two times for 30 min with 5 mL 0.2 m NaOMe in MeOH. In between, the resin was washed with three times with 5 mL MeOH. At the end, the resin was washed with each five times with 5 mL of MeOH, DMF, and DCM.

#### 2.2.8. Macro Cleavage

The resin was washed ten times with 5 mL DMF and DCM. A cleavage solution (5 mL) consisting of 95% trifluoroacetic acid (TFA), 2.5% TIPS, and 2.5% DCM was then added, and the reaction was shaken for 1 h. The supernatant was added dropwise to cooled Et_2_O (40 mL) to precipitate the product. The mixture was centrifuged, the supernatant was decanted, and the white precipitate was dried under a stream of nitrogen. The resulting solid was dissolved in water and lyophilized.

#### 2.2.9. TFA Removal

TFA removal was performed with a AG1-X8, quaternary ammonium, 100–200 mesh, acetate form resin from BioRad according to a protocol by Roux et al.^[73]^ A 1.6 m acetic acid solution was prepared by diluting 23 mL acetic acid in 227 mL water and a 0.16 m solution by diluting 2.3 mL acidic acid in 247.7 mL water. For 100 mg sample, 1000 mg of the ion exchange resin was used. The resin was activated by washing three times with 10 mL of the 1.6 m acetic acid solution, followed by three times with 10 mL of the 0.16 m acetic acid solution. The sample was dissolved in 10 mL water and the solution was loaded to the resin into the syringe. The syringe was shaken for 1 h. The supernatant was recovered, and the resin was washed three times with 2 mL water. The combined water phases were loaded onto new, freshly activated resin and shaken for 1 h. The supernatant was collected, and the resin was washed three times with 2 mL water. The combined liquid phases were lyophilized to obtain the crude product as a white solid.

#### 2.2.10. Additional Note 1

For the acetyl-capped heteroderivatives, precursor scaffold **15** (Figure S5, Supporting Information) *N*-terminus was capped with acetic anhydride followed by deprotection of the carboxylic side chain with lithium hydroxide in THF/H_2_O. In the next step, precursor **15** was conjugated with either a protected 2,3,6,2′,3′,4′,6′-hepta-*O*-acetyl-*β*-lactosyl azide (for **4–6**) or 2-azidoethyl 2,3,4,6-tetra-*O*-acetyl-*α*-D-glucopyranoside (for **10–12**) resulting in **16** and **17**, respectively (Figure S1, Supporting Information). After splitting each batch into three equal aliquots, the nonglycosidic motifs benzylamine (-Bz), *N*-Boc phenylenediamine (-*p*NH_2_Ph), and 4-amino benzene sulfonic acid (-*p*SO_3_HPh), were coupled to the carboxylic side chains using HATU and DIPEA.

#### 2.2.11. Additional Note 2

For the amine and fluorescein isothiocyanate (FITC) derivatives, the Fmoc group of the last EDS building block remained until the end of the solid phase assembly. Since the basic deprotection conditions for the MDS sidechain could result in loss of Fmoc groups, Boc-protected *β*-alanine was used as final building block for structures **6a,c** and **12a,c**. FITC conjugation was performed in solution on purified glycomacromolecules as reported previously,^[74]^ and the corresponding FITC conjugates were repurified by preparative chromatography.

#### 2.2.12. Additional Note 3

Glycomacromolecules were used as isolated after precipitation, TFA removal, and preparative purification. All samples have high purities (see RP-HPLC, Supporting Information), however, they contain small amounts of deletion sequences that are individually assigned and quantified according to HPLC spectra (see the Supporting Information). The ESI-MS-spectrum of the main peak is given, but the analysis of each individual peak is not further shown.

#### 2.2.13. Compound Characterization

##### *Lac(1)-2-NH_2_ (*1a):

^1^H NMR (300 MHz, deuterium oxide) *δ* [ppm]: 7.98–7.94 (m, 1H, triazole-C*H*), 5.66 (d, *J* = 9.2 Hz, 1H C*H*_anomer_Glc), 4.43 (d, *J* = 7.7 Hz, 1H, C*H*_anomer_-Gal), 4.02–3.45 (m, 20H, C*H*_pyranose_, C*H*_2 pyranose_, O-C*H*_2_−), 3.44–3.20 (m, 10H, C═ONH─C*H*_2_), 3.13 (t, *J* = 5.0 Hz, 2H, C*H*_2_─NH_2_), 2.96 (t, *J* = 7.0 Hz, 2H, CH═CH─C*H*_2_), 2.73 (t, *J* = 7.0 Hz, 2H, CH═CH─CH_2_─C*H*_2_), 2.50–2.34 (m, 8H, NHC═O─C*H*_2_). ^13^C NMR (75 MHz, deuterium oxide) *δ* [ppm]: 188.64, 175.21, 175.03, 87.38, 77.88, 77.55, 75.63, 72.73, 72.15, 71.17, 69.66, 69.07, 68.79, 66.72, 61.30, 60.24, 45.16, 41.31, 39.32, 39.05, 37.10, 32.07, 31.17, 31.16, 31.00, 30.85, 30.48. HR MS (ESI^+^) *m/z* calc. for C_35_H_63_N_9_O_17_ [M+2H]^2+^ 440.72; found 440.72. Yield: 48 mg (54%).

##### *Lac(1)-2-Ac (*1b):

^1^H NMR (300 MHz, deuterium oxide) *δ* [ppm]: 8.05 (s, 1 H, triazole-C*H*), 5.75 (d, *J* = 9.2 Hz, 1H, C*H*_anomer_Glc), 4.52 (d, *J* = 7.7 Hz, 1H, C*H*_anomer_-Gal), 4.10–3.73 (m, 10H, C*H*_pyranose_, C*H*_2 pyranose_, O─C*H*_2_−), 3.72–3.57 (m, 10H, C*H*_pyranose_, C*H*_2 pyranose_, O─C*H*_2_−), 3.47–3.31 (m 12 H, C═ONH─C*H*_2_), 3.05 (t, *J* = 7.1 Hz, 2H, CH═CH─C*H*_2_), 2.82 (t, *J* = 7.0 Hz, 2H, ─CH═CH─CH_2_─C*H*_2_), 2.58–2.43 (m, 8H, NHC═O─C*H*_2_), 2.00 (s, 3H, −C*H*_3_). ^13^C NMR (75 MHz, deuterium oxide) *δ* [ppm]: 176.64, 174.15, 173.97, 173.89, 173.80, 173.37, 146.24, 121.71, 102.05, 86.31, 76.81, 76.46, 74.55, 73.71, 71.66, 71.09, 70.10, 68.56, 67.93, 67.72, 65.16, 60.23, 58.89, 46.28, 44.13, 38.11, 38.05, 36.44, 36.02, 30.98, 29.99, 29.79, 29.36, 20.97, 19.80. HR MS (ESI^+^) *m/z* calc. for C_37_H_65_N_9_O_18_ [M+2H]^2+^ 461.7218; found 461.7217. Yield: 51 mg (55%).

##### *Lac(1)-2-FITC (*1c):

^1^H NMR (300 MHz, deuterium oxide) *δ* [ppm]: 8.21–7.88 (m, 3H; FITC-C*H*, triazole-C*H*), 7.65 (s, 1H, FITC-C*H*), 7.11–6.43 (m, 6, FITC-C*H*), 5.68 (d, *J* = 8.9 Hz, 1H, C*H*_anomer_Glc), 4.49 (d, *J* = 7.3 Hz, 1H, C*H*_anomer_-Gal), 4.12–3.11 (m, 32H, C*H*_pyranose_, C*H*_2 pyranose_, O─C*H*_2_─, C═ONH─C*H*_2_), 3.00–2.82 (m, 2H, CH═CH─C*H*_2_), 2.78–2.58 (m, 2H, CH═CH─CH_2_─C*H*_2_), 2.53–2.14 (m, 8H, NHC═O─C*H*_2_). HR MS (ESI^+^) *m/z* calc. for C_54_H_62_N_24_O_12_S [M+2H]^2+^ 635.2344; found 635.2344. Yield: 11 mg (56%).

##### *Lac(1,3,5)-6-NH_2_ (*2a):

^1^H NMR (300 MHz, deuterium oxide) *δ* [ppm]: 8.05 (s, 3H, triazol-C*H*), 5.75 (d, *J* = 9.2 Hz, 3H, C*H*_anomer_Glc), 4.52 (d, *J* = 7.7 Hz, 3H, C*H*_anomer_Gal), 4.11–3.54 (m, 60H, C*H*_pyranose_, C*H*_2 pyranose_, O─C*H*_2_−), 3.54–3.28 (m, 34H, C*H*_pyranose_, C═ONH─C*H*_2_), 3.26–3.18 (m, 2H, C*H*_2_─NH_2_), 3.04 (t, *J* = 6.9 Hz, 6H, CH═CH─C*H*_2_), 2.81 (t, *J* = 6.9 Hz, 6H, CH═CH─CH_2_─C*H*_2_), 2.48 (h, *J* = 6.6 Hz, 24H, NHC═O─C*H*_2_). ^13^C NMR (75 MHz, deuterium oxide) *δ* [ppm]: 175.19, 175.00, 174.92, 174.83, 170.60, 147.30, 122.78, 103.15, 87.41, 77.90, 77.58, 75.65, 74.82, 72.76, 72.18, 71.19, 69.79, 69.66, 69.07, 68.81, 66.62, 61.31, 59.98, 47.34, 45.20, 39.33, 39.14, 37.53, 37.11, 32.07, 31.24, 31.08, 20.90. HR MS (ESI^+^) *m/z* calc. for C_105_H_180_N_25_O_51_ [M+3H]^3+^ 869.07; found: 869.08. Yield: 103 mg (40%).

##### *Lac(1,3,5)-6-Ac (*2b):

^1^H NMR (300 MHz, deuterium oxide) *δ* [ppm]: 8.05 (s, 3H, triazole-C*H*), 5.75 (d, *J* = 9.2 Hz, 3H, C*H*_anomer_Glc), 4.52 (d, *J* = 7.7 Hz, 3H, C*H*_anomer_-Gal), 4.13–3.56 (m, 60H, C*H*_pyranose_, C*H*_2 pyranose_, O─C*H*_2_−), 3.53–3.26 (m, 36H, C*H*_pyranose_, C*H*_2 pyranose_, NHC═O─C*H*_2_), 3.04 (t, *J* = 7.1 Hz, 6H, CH═CH─C*H*_2_), 2.81 (t, *J* = 7.1 Hz, 6H, CH═CH─CH_2_─C*H*_2_), 2.60–2.38 (m, 24H, NHC═O─C*H*_2_), 1.99 (s, 3H, C*H*_3_). ^13^C NMR (75 MHz deuterium oxide) *δ* [ppm]: 174.43, 174.24, 174.17, 174.08, 146.53, 122.02, 102.38, 86.64, 77.13, 76.80, 74.87, 74.05, 71.98, 71.42, 70.42, 68.88, 68.30, 68.04, 60.54, 59.22, 46.57, 44.42, 38.43, 38.37, 36.76, 36.34, 31.29, 30.48, 30.36, 30.27, 21.29, 20.13. HR MS (ESI^+^) *m/z* calc. for C_107_H_182_N_25_O_52_ [M+3H]^3+^ 883.0783; found: 883.0787. Yield: 109 mg (41%).

##### *Lac(1,3,5)-6-FITC (*2c):

^1^H NMR (600 MHz, deuterium oxide) *δ* [ppm]: 8.12 (s, 1H, FITC-C*H*), 8.07–7.95 (m, 3H, triazole-C*H*), 7.70 (s, 1H, FITC-C*H*), 7.07 (s, 1H, FITC-C*H*), 6.90 (s, 2H, FITC-C*H*), 6.79 (s, 2H, FITC-C*H*), 6.67 (d, *J* = 8.8 Hz, 2H, FITC-C*H*), 5.77–5.66 (m, 3H, C*H*_anomer_-Glc), 4.55–4.46 (m, 3H, C*H*_anomer_-Gal), 4.07–3.99 (m, 3H, C*H*_pyranose_), 3.97–3.79 (m, 20H, C*H*_pyranose_, C*H*_2 pyranose_, O─C*H*_2_−), 3.79–3.51 (m, 40H, C*H*_pyranose_, C*H*_2 pyranose_, O─C*H*_2_─, C═ONH─C*H*_2_), 3.48–3.20 (m, 34H, C*H*_pyranose_, C═ONH─C*H*_2_), 3.07–2.89 (m, 6H, CH═CH─C*H*_2_), 2.83–2.66 (m, 6H, CH═CH─CH_2_─C*H*_2_), 2.53–2.34 (m, 24H, NHC═O─C*H*_2_). ^13^C NMR (151 MHz, deuterium oxide) *δ* [ppm]: 177.48, 174.58, 146.96, 122.47, 102.92, 87.18, 77.65, 77.39, 75.40, 74.58, 72.52, 71.94, 70.94, 69.40, 68.81, 68.56, 61.06, 59.76, 47.10, 45.01, 38.89, 37.30, 36.88, 31.76, 31.00, 30.88, 30.83, 30.77, 30.21, 30.08, 20.64. HR MS (ESI^+^) *m/z* calc. for C_126_H_191_N_26_O_56_S [M+3H]^3+^ 998.7534; found 998.7514. Yield: 21 mg (60%).

##### *Lac(1,2,3,4,5,6)-7-NH_2_ (*3a):

^1^H NMR (300 MHz, deuterium oxide) *δ* [ppm]: 8.05–8.01 (m, 6H, triazole-C*H*), 5.74 (d, *J* = 9.2 Hz, 6H, C*H*_anomer_Glc), 4.52 (d, *J* = 7.6 Hz, 4H, C*H*_anomer_-Gal), 4.09–3.82 (m, 44H, C*H*_pyranose_, O─C*H*_2_−), 3.82–3.54 (m, 36H, C*H*_pyranose_, C*H*_2 pyranose_, O─C*H*_2_−), 3.52–3.27 (m, 50H, C═ONH─C*H*_2_), 3.24–3.19 (m, 2H, C*H*_2_─NH_2_), 3.07–2.95 (m, 12H, CH═CH─C*H*_2_), 2.85–2.73 (m, 12H, CH═CH─CH_2_─C*H*_2_), 2.56–2.37 (m, 28H, NHC═O─C*H*_2_). ^13^C NMR (126 MHz, deuterium oxide) *δ* [ppm]: 173.92, 146.27, 121.70, 102.13, 86.36, 76.86, 76.77, 74.59, 73.82, 71.78, 71.15, 70.18, 67.79, 65.08, 60.24, 59.06, 46.29, 44.15, 38.32, 38.04, 36.54, 36.12, 31.01, 30.18, 30.06, 30.01, 29.89, 19.89, 13.27. HR MS (ESI^+^) *m/z* calc. for C_160_H_265_N_39_O_82_ [M+4H]^4+^ 1011.1936; found 1011.1945. Yield: 174 mg (43%).

##### *Lac(1,2,3,4,5,6)-7-NHAc (*3b):

^1^H NMR (600 MHz, deuterium oxide) *δ* [ppm]: 8.04 (m, 6H, triazole-C*H*), 5.74 (d, *J* = 9.2 Hz, 6H, C*H*_anomer_Glc), 4.52 (d, *J* = 7.6 Hz, 6H, C*H*_anomer_-Gal), 4.10–3.74 (m, 62H, CH_pyranose_, O─CH_2_−), 3.72–3.54 (m, 20H, CH_pyranose_, C*H*_2 pyranose_, O─C*H*_2_−), 3.50–3.27 (m, 50H, C═ONH─C*H*_2_), 3.04–2.98 (m, 12H, CH═CH─C*H*_2_), 2.85–2.72 (m, 12H, CH═CH─CH_2_─C*H*_2_), 2.52–2.41 (m, 28H, NHC═O─C*H*_2_), 1.99 (s, 1H, C*H*_3_). ^13^C NMR (151 MHz, deuterium oxide) *δ* [ppm]: 215.59, 174.62, 174.05, 146.95, 122.42, 102.80, 87.07, 77.55, 77.24, 75.29, 74.47, 72.41, 71.84, 70.85, 69.29, 68.46, 60.96, 59.64, 46.96, 44.80, 38.84, 37.16, 36.75, 31.70, 30.67, 30.15, 29.89, 29.63, 29.37, 29.11, 28.85, 28.60, 20.56. HR MS (ESI^+^) *m/z* calc. for C_162_H_267_N_39_O_83_ [M+4H]^4+^ 1021.6962; found 1021.6962. Yield: 235.1 mg (55%).

##### *Lac(1,2,3,4,5,6)-7-FITC (*3c):

^1^H NMR (600 MHz, deuterium oxide) *δ* [ppm]: *δ* 8.11 (s, 1H, FITC-C*H*), 8.08–7.93 (m, 6H, triazole-C*H*), 7.71 (s, 1H, FITC-C*H*), 7.15 (d, *J* = 6.5 Hz, 1H, FITC-C*H*), 6.93 (d, *J* = 7.0 Hz, 2H, FITC-C*H*), 6.86 (s, 2H, FITC-C*H*), 6.71 (d, *J* = 7.0 Hz, 2H, FITC-C*H*), 5.79–5.66 (m, 6H, C*H*_anomer_Glc), 4.58–4.44 (m, 6H, C*H*_anomer_-Gal), 4.07–3.54 (m, 80H, C*H*_pyranose_, C*H*_2 pyranose_, O─C*H*_2_−), 3.51–3.18 (m, 52H, C*H*_pyranose_, C═ONH─C*H*_2_), 3.07–2.89 (m, 12H, CH═CH─C*H*_2_), 2.85–2.65 (m, 12H, CH═CH─CH_2_─C*H*_2_), 2.55–2.32 (m, 28H, NHC═O─C*H*_2_). ^13^C NMR (151 MHz, deuterium oxide) *δ* [ppm]: 174.61, 146.99, 122.46, 102.92, 87.17, 77.64, 77.39, 75.39, 74.57, 72.51, 71.94, 70.94, 68.55, 61.05, 59.76, 47.06, 44.95, 37.28, 36.87, 31.75, 30.79, 30.61, 20.64. HR MS (ESI^+^) *m/z* calc. for C_182_H_276_N_38_O_88_S [M+4H]^4+^ 1108.4497; found 1108.4503. Yield: 15 mg (33%).

##### *Lac(1,3,5)-Bz(2,4)-6-Ac (*4b):

^1^H NMR (300 MHz, deuterium oxide) *δ* [ppm]: 8.05–8.00 (m, 3H, triazole-C*H*), 7.41–7.25 (m, 10H, aromatic-C*H*), 5.79–5.68 (m, 3H, C*H*_anomer_Glc), 4.56–4.46 (m, 2H, C*H*_anomerGal_), 4.35 (s, 4H, aromatic C*H*_2_), 4.12–3.53 (m, 48H, C*H*_pyranose_, C*H*_2 pyranose_, O─C*H*_2_−), 3.52–3.22 (s, 44H, C*H*_pyranose_, C═ONH─C*H*_2_), 3.08–2.95 (m, 6H, CH═CH─C*H*_2_), 2.84–2.73 (m, 6H, CH═CH─CH_2_─C*H*_2_), 2.73–2.34 (m, 34H, C*H*_pyranose_, NHC═O─C*H*_2_), 1.99 (s, 3H, C*H*_3_). ^13^C NMR (126 MHz, deuterium oxide) *δ* [ppm]: 173.96, 173.91, 173.79, 146.27, 137.37, 127.92, 126.54, 126.34, 121.70, 102.14, 86.36, 76.86, 76.77, 74.59, 73.82, 71.79, 71.15, 70.17, 68.61, 68.01, 67.97, 67.78, 65.08, 60.23, 59.05, 46.32, 46.24, 44.21, 42.11, 38.16, 38.11, 36.55, 36.50, 36.13, 30.99, 30.25, 30.19, 30.13, 30.08, 30.03, 29.91, 29.47, 29.34, 27.35, 21.05, 19.88, 13.28. HR MS (ESI^+^) *m/z* calc. for C_125_H_198_N_29_O_52_ [M+3H]^3+^ 979.1241; found 979.1237. Yield: 104 mg (35%).

##### *Lac(1,3,5)-pNH_2_Ph(2,4)-6-Ac (*5b):

^1^H NMR (300 MHz, deuterium oxide) *δ* [ppm]: 8.09–7.97 (m, 3H, triazole-C*H*), 7.23 (d, *J* = 8.4 Hz, 4H, aromatic C*H*), 6.87 (d, *J* = 8.4 Hz, 4H, aromatic C*H*), 5.79–5.68 (m, 3H, C*H*_anomer_Glc), 4.55–4.45 (m, 3H, C*H*_anomer_Gal), 4.11–3.57 (m, 46H, C*H*_pyranose_, C*H*_2 pyranose_, O─C*H*_2_−), 3.56–3.23 (m, 46H, C*H*_pyranose_, C═ONH─C*H*_2_), 3.08–2.93 (m, 6H, CH═CH─C*H*_2_), 2.85–2.59 (m, 15H, CH═CH─CH_2_─C*H*_2_, C*H*_pyranose_), 2.55–2.30 (m, 24H, C*H*_pyranose_, NHC═O─C*H*_2_), 1.99 (s, 3H, C*H*_3_). ^13^C NMR (126 MHz, deuterium oxide) *δ* [ppm]: 173.91, 146.26, 122.48, 121.70, 116.63, 102.14, 86.36, 76.86, 76.78, 74.60, 73.83, 71.79, 71.15, 70.18, 68.61, 68.01, 67.97, 67.79, 60.24, 59.06, 46.33, 44.22, 38.16, 38.11, 36.55, 36.21, 36.14, 30.99, 30.09, 29.94, 27.29, 21.06, 19.89, 13.28. HR MS (ESI^+^) *m/z* calc. for C_123_H_197_N_31_O_52_ [M+4H]^4+^ 735.0925; found 735.0917. Yield: 110 mg (38%).

##### *Lac(1,3,5)-pSO_3_H Ph(2,4)-6-NH_2_ (*6a):

^1^H NMR (300 MHz, deuterium oxide) *δ* [ppm]: 8.06–7.98 (m, 3H, triazole-C*H*), 7.76 (d, *J* = 8.7 Hz, 4H, aromatic C*H*), 7.57 (d, *J* = 8.6 Hz, 4H, aromatic C*H*), 5.79–5.67 (m, 3H, C*H*_anomer_-Glc), 4.56–4.45 (m, 3H, C*H*_anomer_-Gal), 4.10–3.70 (m, 30H, C*H*_pyranose_, C*H*_2 pyranose_, O─C*H*_2_−), 3.68–3.20 (m, 60H, C*H*_pyranose_, C═ONH─C*H*_2_, C*H*_2_─NH_2_), 3.11–2.91 (m, 6H, CH═CH─C*H*_2_), 2.85–2.62 (m, 16H, CH═CH─CH_2_─C*H*_2_, NHC═O─C*H*_2_), 2.55–2.31 (m, 24H, NHC═O─C*H*_2_). ^13^C NMR (151 MHz, deuterium oxide) *δ* [ppm]: 173.90, 173.90, 173.76, 172.58, 171.31, 146.24, 139.24, 137.54, 125.68, 121.71, 119.55, 102.06, 86.32, 76.80, 76.52, 74.55, 73.74, 71.69, 71.13, 70.13, 68.54, 67.97, 67.88, 67.75, 60.23, 58.93, 48.46, 46.24, 44.12, 38.04, 36.43, 36.03, 34.87, 31.06, 30.92, 30.69, 29.99, 29.89, 29.80, 26.95, 19.81. HR MS (ESI^+^) *m/z* calc. for C_124_H_197_N_30_O_58_S_2_ [M+3H]^3+^ 1032.7604; found 1032.7593. Yield: 27 mg (10%).

##### *Lac(1,3,5)-pSO_3_H Ph(2,4)-6-Ac (*6b):

^1^H NMR (300 MHz, deuterium oxide) *δ* [ppm]: 8.09–7.96 (m, 3H, triazole-C*H*), 7.76 (d, *J* = 8.7 Hz, 4H, aromatic C*H*), 7.58 (d, *J* = 8.5 Hz, 4H, aromatic C*H*), 5.86–5.60 (m, 3H, C*H*_anomer_-Glc), 4.58–4.43 (m, 3H, C*H*_anomer_-Gal), 4.12–3.97 (m, 3H, C*H*_pyranose_), 3.97–3.49 (m, 45H, C*H*_pyranose_, C*H*_2 pyranose_, O─C*H*_2_−), 3.49–3.22 (m, 40H, C═ONH─C*H*_2_), 3.11–2.90 (m, 6H, CH═CH─C*H*_2_), 2.84–2.65 (m, 14H, CH═CH─CH_2_─C*H*_2_), 2.58–2.32 (m, 24H, NHC═O─C*H*_2_), 1.99 (s, 3H). ^13^C NMR (126 MHz, deuterium oxide) *δ* [ppm]: 173.90, 146.27, 141.84, 139.20, 125.72, 121.74, 119.67, 102.13, 86.37, 76.86, 76.76, 74.57, 73.80, 71.78, 71.15, 70.17, 68.60, 67.96, 67.79, 65.08, 60.22, 59.06, 46.33, 46.12, 44.23, 44.02, 38.17, 38.11, 36.54, 36.21, 36.14, 31.00, 30.79, 30.10, 27.07, 21.05, 19.87, 13.27. HR MS (ESI^+^) *m/z* calc. for C_123_H_194_N_29_O_58_S_2_ [M+3H]^3+^ 1023.0849; found 1023.0848. Yield: 23 mg (10%).

##### *Lac(1,3,5)-pSO_3_H Ph(2,4)-6-FITC (*6c):

^1^H NMR (300 MHz, deuterium oxide) *δ* [ppm]: 8.08–7.95 (m, 3H, triazole-C*H*), 7.79–7.65 (m, 4H, aromatic C*H*), 7.62–7.49 (m, 4H, aromatic C*H*), 7.33–7.19 (m, 2H, FITC-C*H*), 6.76–6.62 (m, 2H, FITC-C*H*), 5.78–5.66 (m, 3H, C*H*_anomer_-Glc), 4.55–4.46 (m, C*H*_anomer_-Gal), 4.12–3.56 (m, 45H, C*H*_pyranose_, C*H*_2 pyranose_, O─C*H*_2_−), 3.56–3.16 (m, 45H, C*H*_pyranose_, C═ONH─C*H*_2_, C*H*_2_─NH_2_), 3.07–2.91 (m, 6H, CH═CH─C*H*_2_), 2.82–2.60 (m, 17 H, CH═CH─CH_2_─C*H*_2_, NHC═O─C*H*_2_), 2.55–2.17 (m, 23H, NHC═O─C*H*_2_). ^13^C NMR (75 MHz, deuterium oxide) *δ* [ppm]: 174.96, 174.92, 174.79, 167.78, 126.73, 103.13, 93.57, 87.39, 77.87, 77.84, 77.60, 77.58, 75.60, 74.78, 72.73, 72.18, 71.17, 69.69, 69.65, 69.61, 69.55, 68.99, 68.77, 66.77, 61.28, 59.99, 47.08, 45.95, 45.15, 44.24, 37.50, 37.45, 37.23, 37.18, 37.08, 31.21, 31.13, 31.10, 31.05, 20.90. HR MS (ESI^+^) *m/z* calc. for C_146_H_209_N_29_O_64_S_3_ [M+4H]^4^+ 872.0783; found 872.0800. Yield: 7 mg (36%).

##### *Lac(1,3,5-1-SO_3_H,4-OH)Ph(2,4)-6-Ac (*7b):

^1^H NMR (300 MHz, deuterium oxide) *δ* [ppm]: 8.09–7.91 (m, 3H, triazole-C*H*), 7.53 (dd, *J* = 8.5, 2.2 Hz, 2H, aromatic C*H*), 7.03 (d, *J* = 8.5 Hz, 2H, aromatic C*H*), 5.81–5.63 (m, 3H, C*H*_anomer_-Glc), 4.58–4.40 (m, 3H, C*H*_anomer_-Gal), 4.13–3.68 (m, 38H, C*H*_pyranose_, C*H*_2 pyranose_, O─C*H*_2_−), 3.68–3.17 (m, 52H, C*H*_pyranose_, C═ONH─C*H*_2_), 3.10–2.89 (m, 6H, CH═CH─C*H*_2_), 2.83–2.62 (m, 14H, CH═CH─CH_2_─C*H*_2_, NHC═O─C*H*_2_), 2.59–2.30 (m, 24H), 1.99 (s, 3H, C*H*_3_). ^13^C NMR (126 MHz, deuterium oxide) *δ* [ppm]: 173.89, 121.71, 115.45, 102.11, 86.34, 76.84, 76.74, 74.56, 73.83, 71.82, 71.18, 70.20, 68.58, 67.95, 67.83, 65.07, 60.25, 59.09, 56.65, 44.22, 38.15, 36.53, 30.95, 30.11, 19.85, 16.01, 13.27. HR MS (ESI^+^) *m/z* calc. for C_123_H_194_N_29_O_60_S_2_ [M+3H]^3+^ 1033.7482; found 1033.7487. Yield: 20 mg (9%).

##### Lac(1,3,5)-Tyr(4-SO_3_H)(2,4)-6-NH_2_ (8a):

^1^H NMR (600 MHz, deuterium oxide) *δ* [ppm]: 8.08–798 (m, 3H, triazole-C*H*), 729–720 (m, 6H, aromatic C*H*), 720–6.81 (m, 2H, aromatic C*H*), 5.75–5.72 (m, 3H, C*H*_anomer_-Glc), 4.54–4.48 (m, 3H, C*H*anomer-Gal), 4.07–3.56 (m, 52H, Tyr C*H*, C*H*_pyranose_, C*H*_2 pyranose_, O─CH_2_−), 3.44–3.20 (m, 32H, C*H*_pyranose_, C═ONH─C*H*_2_, Tyr C*H*_2_), 3.13–2.72 (m, 20H, CH═CH─CH_2_─C*H*_2_, NHC═O─C*H*_2_), 2.54–2.25 (m, 18H, NHC═O─C*H*_2_). ^13^C NMR (75 MHz, deuterium oxide) *δ* [ppm]: 175.09, 174.95, 174.71, 174.62, 173.28, 150.50, 130.57, 122.74, 121.73, 103.19, 87.40, 77.88, 75.59, 74.84, 72.82, 72.21, 71.21, 69.81, 69.67, 69.08, 68.83, 66.57, 61.24, 60.12, 47.52, 45.29, 39.38, 39.10, 37.60, 37.19, 32.04, 31.14, 30.98, 30.44, 20.91. HR MS (ESI^+^) *m/z* calc. for C_103_H_162_N_23_O_53_S_2_ [M+3H]^3+^ 877.6704; found 877.6701. Yield: 82 mg (31%).

##### Lac(1,3,5)-Tyr(4-SO_3_H)(2,4)-6-FITC (8c):

^1^H NMR (300 MHz, deuterium oxide) *δ* [ppm]: 8.07–789 (m, 4H, triazole-C*H*), 771–757 (m, 1H, FITC-C*H*) 731–714 (m, 8H, phenyl-C*H*, FITC-C*H*), 714–6.96 (m, 2H, FITC-C*H*), 6.86–6.58 (m, 2H, FITC-C*H*), 5.75–5,63 (m, 3H, C*H*_anomer_-Glc), 4.53–4.49 (m, 3H, C*H*_anomer_-Gal), 4.08–3.53 (m, 49H, Tyr C*H*, C*H*_pyranose_, C*H*_2 pyranose_, O─C*H*_2_−), 3.49–3.15 (m, 29H, C*H*_pyranose_, C═ONH─C*H*_2_, Tyr C*H*_2_), 3.11–2.83 (m, 10H, CH═CH─CH_2_─C*H*_2_, NHC═O─C*H*_2_), 2.82–2.65 (m, 6H, CH═CH─CH_2_─C*H*_2_), 2.55–2.23 (m, 16H, NHC═O─C*H*_2_). ^13^C NMR (75 MHz, deuterium oxide) *δ* [ppm]: 175.11, 175.00, 174.97, 174.90, 174.90, 174.74, 174.65, 174.58, 173.52, 150.53, 147.36, 147.28, 131.07, 130.59, 122.76, 122.66, 122.63, 121.75, 113.48, 103.35, 103.23, 87.44, 77.91, 75.62, 74.87, 72.86, 72.25, 71.25, 69.84, 69.81, 69.71, 69.12, 68.87, 66.61, 61.27, 60.17, 55.24, 55.19, 55.18, 55.15, 47.56, 47.48, 47.42, 47.20, 47.13, 45.44, 45.38, 45.34, 45.32, 45.28, 39.42, 39.25, 39.14, 37.63, 37.61, 37.58, 37.45, 37.42, 37.23, 36.77, 36.75, 36.57, 32.08, 32.03, 31.33, 31.26, 31.18, 31.01, 30.92, 30.90, 30.60, 30.48, HR MS (ESI^+^) *m/z* calc. for C_124_H_173_N_24_O_58_S_3_ [M+3H]^3+^ 10073490; found 10073480. Yield: 5 mg (32%).

##### Glc(1,3,5)-6-NH_2_ (9a):

^1^H NMR (600 MHz, deuterium oxide) *δ* [ppm]: 8.46 (br s, 1 H, NH), 788 (m, 3H, triazole-C*H*), 4.80 (m, 3H, C*H*_anomer_Glc), 4.64 (m, 6H, −N─N─C*H*_2_−), 4.41 (d, ^3^JHH = 79 Hz, 0.6H, C*H*_anomer_Glc), 4.07 (m, 3H, O─C*H*_2_−), 3.91 (m, 3H, O─C*H*_2_−), 3.75 (dd, 3J = 5.6; 4.6 Hz, 2H, O─C*H*_2_−), 3.69 (s, 4H, O─C*H*_2_−), 3.65 (s, 8H, O─C*H*_2_−, C*H*_pyranose_), 3.63–3.28 (m, 59H, O─C*H*_2_−, C═ONH─C*H*_2_, C*H*_pyranose_), 3.21 (m, 2H, C*H*_2_─NH_2_), 2.98 (m, 6H, CH═C─C*H*_2_), 2.87–2.75 (m, 9H, C*H*_pyranose_, CH═C─CH_2_─C*H*_2_), 2.48 (m, 24H, NHC═O─C*H*_2_). ^13^C NMR (75 MHz, deuterium oxide) ^δ^ [ppm]: 177.79, 175.18, 175.01, 174.94, 174.85, 171.24, 147.07, 124.31, 102.74, 98.20, 76.20, 75.89, 73.23, 73.18, 72.04, 71.42, 69.85, 69.81, 69.68, 69.32, 68.42, 66.73, 66.12, 60.39, 50.23, 47.39, 45.27, 39.36, 39.16, 39.09, 37.57, 37.15, 32.23, 31.28, 31.15, 31.11, 31.03, 30.52, 20.90. HR MS (ESI^+^) *m/z* calc. for C_93_H_162_N_25_O_39_ [M+3H]^3+^ 751.04; found 751.25. Yield: 86 mg (38%).

##### Glc(1,3,5)-6-Ac (9b):

^1^H NMR (300 MHz, deuterium oxide) *δ* [ppm]: 791 (s, 3H, triazole-C*H*), 4.88 (d, *J* = 3.4 Hz, 3H, C*H*_a−nomer_Glc), 4.71–4.61 (m, 6H, −N─N─CH_2_−), 4.15–4.03 (m, 3H, C*H*_pyranose_), 3.99–3.89 (m, 3H, C*H*_pyranose_), 3.74–3.55 (m, 35H, CH-Glc, −O─C*H*_2_), 3.54–3.43 (m, 18H, −O─C*H*_2_, C*H*_pyranose_), 3.42–3.30 (m, 30H, −NH─C*H*_2_−), 3.00 (t, *J* = 73 Hz, 6H, CH═C─C*H*_2_), 2.91–2.74 (m, 9H, C*H*_pynnose_, NH─C═O─C*H*_2_−), 2.58–2.42 (m, 28H, NH─C═O─C*H*_2_−), 2.00 (s, 3H, C*H*_3_). ^13^C NMR (75 MHz, deuterium oxide) *δ* [ppm]: 173.96, 173.82, 173.78, 173.72, 173.63, 145.83, 123.07, 96.96, 71.94, 70.80, 70.19, 68.44, 68.08, 67.86, 67.80, 64.89, 59.14, 48.99, 46.13, 44.05, 37.92, 36.34, 35.91, 31.00, 30.05, 29.88, 29.80, 20.85, 19.66. HR MS (ESI^+^) *m/z* calc. for C_95_H_164_N_25_O_40_ [M+3H]^3+^ 765.0517; found 765.0527. Yield: 110 mg (48%).

##### Glc(1,3,5)-6-FITC (9c):

^1^H NMR (300 MHz, deuterium oxide) *δ* [ppm]: 8.02 (s, 1H, FITC-C*H*), 791–776 (m, 3H, triazole-C*H*), 764 (d, *J* = 71 Hz, 1H, FITC-C*H*), 701 (d, *J* = 75 Hz, 1H, FITC-C*H*), 6.80–6.64 (m, 4H, FITC-C*H*), 6.57 (d, *J* = 8.7 Hz, 2H, FITC-C*H*), 4.88–4.84 (m, 3H, C*H*_anomer_Glc), 4.66–4.52 (m, 6H, −N─N─C*H*_2_−), 4.13–3.96 (m, 3H, C*H*_pynnose_), 3.96–3.82 (m, 3H C*H*_pyranos_), 3.79–3.48 (m, 38H C*H*_pyranose_, C*H*_2 pyranose_, O─C*H*_2_−), 3.48–3.22 (m, 40H, C*H*_pynnose_, C═ONH─C*H*_2_), 3.02–2.82 (m, 8H, C*H*_pynnose_, CH═CH─C*H*_2_), 2.80–2.64 (m, 6H, CH═CH─CH_2_─C*H*_2_), 2.54–2.36 (m, 24H, NHC═O═C*H*_2_). ^13^C NMR (75 MHz, deuterium oxide) *δ* [ppm]: 175.10, 174.79, 174.69, 141.29, 131.57, 124.21, 98.20, 73.18, 72.04, 71.43, 69.67, 69.32, 69.09, 66.11, 60.38, 47.39, 46.04, 45.32, 44.48, 39.15, 37.58, 3715, 3712, 31.26, 31.11, 31.04, 20.90. HR MS (ESI^+^) *m/z* calc. for C_114_H_173_N_26_O_44_S [M+3H]^3+^ 880.7268; found 880.7260. Yield: 15 mg (42%).

##### Glc(1,3,5)-Bz(2,4)-6-Ac (10b):

^1^H NMR (300 MHz, deuterium oxide) *δ* [ppm]: 794–782 (m, 3H, triazole-C*H*), 744–724 (m, 10H, aromatic-C*H*), 4.90–4.85 (m, 3H, C*H*_anomer_Glc), 4.68–4.56 (m, 6H, −N─N─C*H*_2_−), 4.35 (s, 4H, aromatic C*H*_2_), 4.14–4.01 (m, 3H, C*H*_pyranose_), 3.97–3.84 (m, 3H, C*H*_pyranose_), 3.68–3.57 (m, 15H, C*H*_pyranose_, O─C*H*_2_−), 3.55–3.26 (m, 53H, C*H*_pyranose_ C═ONH─C*H*_2_), 3.04–2.92 (m, 6H, CH═C─C*H*_2_), 2.90–2.81 (m, 3H, C*H*_pyranose_), 2.81–2.66 (m, 10H, NH─C═O─C*H*_2_−), 2.62–2.38 (m, 28H, NH─C═O─C*H*_2_−), 1.99 (s, 3H, −C*H*_3_). ^13^C NMR (126 MHz, deuterium oxide) *δ* [ppm]: 173.97, 173.91, 173.79, 146.00, 127.89, 126.52, 126.32, 123.14, 97.20, 72.16, 71.02, 70.39, 68.61, 68.01, 67.97, 65.08, 59.42, 49.13, 46.33, 44.28, 42.10, 38.16, 36.56, 36.13, 31.15, 30.19, 30.12, 30.07, 30.03, 29.92, 27.35, 19.85, 0.00. HR MS (ESI^+^) *m/z* calc. for C_113_H_180_N_29_O_40_ [M+3H]^3+^ 861.0975; found 861.0978. Yield: 81 mg (31%).

##### Glc(1,3,5)-pNH_2_Ph(2,4)-6-Ac (11b):

^1^H NMR (300 MHz, deuterium oxide) *δ* [ppm]: 795–780 (m, 3H, triazole-C*H*), 722 (d, *J* = 8.5 Hz, 4H, aromatic-C*H*), 6.85 (d, *J* = 8.2 Hz, 4H, aromatic-C*H*), 4.89–4.84 (m, 3H, C*H*_anomer_Glc), 4.69–4.56 (m, 6H, −N─N─C*H*_2_−), 4.13–4.00 (m, 3H, C*H*_pyranose_), 3.96–3.85 (m, 3H, C*H*_pyranose_), 3.70–3.58 (m, 14H, C*H*_pyranose_, O─C*H*_2_−), 3.58–3.25 (m, 53H, C*H*_pyranose_ C═ONH─C*H*_2_), 3.02–2.91 (m, 6H, CH═C─C*H*_2_), 2.89–2.81 (m, 3H, C*H*_pyranose_), 2.81–2.61 (m, 14H, −N─C═O─C*H*_2_−), 2.56–2.35 (m, 24H, NH─C═O─C*H*_2_−), 1.99 (s, 3H, −C*H*_3_). ^13^C NMR (75 MHz, deuterium oxide) *δ* [ppm]: 175.53, 175.34, 175.27, 175.18, 174.77, 14764, 123.13, 103.49, 87.75, 78.24, 77.91, 75.99, 75.15, 73.09, 72.53, 71.53, 69.99, 69.41, 69.15, 66.58, 61.65, 60.33, 47.67, 45.54, 39.54, 39.48, 37.87, 3745, 32.41, 31.59, 31.42, 30.80, 22.40, 21.24, 14.66. HR MS (ESI^+^) *m/z* calc. for C_111_H_178_N_31_O_40_ [M+3H]^3+^ 861.7610; found 861.7597 Yield: 84 mg (33%).

##### Glc(1,3,5)-pSO_3_H Ph(2,4)-6-NH_2_ (12a):

^1^H NMR (300 MHz deuterium oxide) *δ* [ppm]: 8.26 (br s, 1H, NH), 793–784 (m, 3H, triazole-C*H*), 775 (d, *J* = 8.6 Hz, 4H, aromatic-C*H*), 756 (d, *J* = 8.6 Hz, 4H, aromatic-C*H*), 4.89–4.84 (m, 3H, C*H*_anomer_Glc), 4.69–4.55 (m, 6H, −N─N─C*H*_2_), 4.12–4.00 (m, 3H, C*H*_pyranose_), 3.97–3.83 (m, 3H, C*H*_pyranose_), 3.69–3.52 (m, 23H, C*H*_pyranose_, C*H*_2 pyranose_, O─C*H*_2_−) 3.50–3.22 (m 47H C*H*_pyranose_, C*H*_2 pyranose_, C═ONH─C*H*_2_, C*H*_2_─NH_2_), 3.03–2.91 (m, 6H, CH═CH─C*H*_2_), 2.91–2.82 (m, 3H, C*H*_pyranose_), 2.81–2.64 (m, 16H, CH═CH─CH_2_─C*H*_2_, NH─C═O─C*H*_2_), 2.57–2.32 (m, 24H, NH─C═O─C*H*_2_). ^13^C NMR (75 MHz, deuterium oxide) *δ* [ppm]: 174.68, 140.06, 138.34, 126.46, 120.30, 9790, 72.89, 71.74, 69.36, 69.03, 65.82, 60.09, 49.94, 45.03, 38.86, 36.88, 35.69, 31.89, 30.84, 20.62. HR MS (ESI^+^) *m/z* calc. for C_112_H_179_N_30_O_46_S_2_ [M+3H]^3+^ 914.7338; found 914.7332. Yield: 30 mg (15%).

##### Glc(1,3,5)-pSO_3_H Ph(2,4)-6-Ac (12b):

^1^H NMR (300 MHz, deuterium oxide) *δ* [ppm]: *δ* 7.93–7.82 (m, 3H, triazole-C*H*), 775 (d, *J* = 8.5 Hz, 4H, aromatic-C*H*), 757 (d, *J* = 8.5 Hz, 4H, aromatic-C*H*), 4.88–4.84 (m, 3H, C*H*_anomer_Glc), 4.69–4.56 (m, 6H, −N─N─C*H*_2_−), 4.12–4.00 (m, 3H, C*H*_pyranose_), 3.97–3.85 (m, 3H, C*H*_pyranose_), 3.69–3.58 (m, 36H, C*H*_pyranose_, O─C*H*_2_−), 3.57–3.24 (m, 59H, O─C*H*_2_−, C═ONH─C*H*_2_, C*H*_pyranose_), 3.03–2.91 (m, 6H, CH═C─C*H*_2_), 2.90–2.81 (m, 4H, C*H*_pyranose_), 2.80–2.68 (m, 14H, C*H*_pyranose_ CH═C, −CH_2_─C*H*_2_), 2.55–2.34 (m, 26H, NHC═O─C*H*_2_), 1.99 (s, 3H, C*H*_3_). ^13^C NMR (126 MHz, deuterium oxide) *δ* [ppm]: 174.05, 173.89, 125.69, 119.61, 99.01, 97.19, 72.16, 71.00, 70.40, 68.60 68.36, 67.96, 65.08, 59.42, 46.34, 44.28, 38.16, 38.11, 36.56, 36.22, 36.15, 31.13, 30.10, 19.85, 13.27 HR MS (ESI^+^) *m/z* calc. for C_111_H_176_N_29_O_46_S_2_ [M+3H]^3+^ 905.0583; found 905.0574. Yield: 23 mg (9%).

##### Glc(1,3,5)-pSO_3_H Ph(2,4)-6-FITC (12c):

^1^H NMR (300 MHz deuterium oxide) *δ* [ppm]: 792–780 (m, 3H, triazole-C*H*), 780–763 (m, 5H, aromatic-C*H*, FITC C*H*), 762–747 (m, aromatic-C*H*, FITC C*H*), 732–715 (m, 1H, FITC C*H*), 6.74–6.53 (m, 2H, FITC C*H*), 4.89–4.84 (m, 3H, C*H*_anomer_Glc), 4.66–4.54 (m, 6H, −N─N─C*H*_2_), 4.13–3.98 (m, 3H, C*H*_pyranose_), 3.96–3.83 (m, 3H, C*H*_pyranos_), 3.69–3.23 (m 23H C*H*_pyranose_, C*H*_2 pyranose_, O─C*H*_2_ C═ONH─C*H*_2_), 3.03–2.65 (m, 28H, CH─CH─CH_2_─C*H*_2_, NH─C═O─C*H*_2_), 2.55–2.33 (m, 26H, NH─C═O─C*H*_2_). ^13^C NMR (75 MHz, deuterium oxide) *δ* [ppm]: 174.92, 147.00, 140.31, 126.70, 124.21, 120.53, 98.14, 73.12, 71.97, 71.39, 69.27, 60.33, 50.15, 47.34, 45.27, 39.09, 37.49, 37.44, 37.13, 37.11, 32.16, 31.09, 30.48, 20.85. HR MS (ESI^+^) *m/z* calc. for C_133_H_191_N_31_O_51_S_3_ [M+4H]^4+^ 783.5611; found 783.5627 Yield: 9 mg (33%).

##### Glc(1,3,5)-Tyr(4-SO_3_H)(2,4)-6-NH_2_ (13a):

^1^H NMR (600 MHz, deuterium oxide) *δ* [ppm]: 795–787 (m, 3H, triazole-C*H*), 729–719 (m, 8H, aromatic C*H*), 4.90–4.85 (m, 3H, C*H*_anomer_Glc), 4.68–4.57 (m, 6H, −N─N─C*H*_2_), 4.55–4.48 (m, 3H, C*H*_pyranose_), 4.12–4.04 (m, 3H, C*H*_pyranose_), 3.96–3.86 (m, 3H, C*H*_pyranose_), 3.78–3.74 (m, 2H, O─C*H*_2_−), 3.71–3.54 (m, 14H, Tyr C*H*, C*H*_pyranose_, C*H*_2 pyranose_, O─C*H*_2_−), 3.51–3.19 (m, 36H, O─C*H*_2_−, C═ONH─C*H*_2_, C*H*_pyranose_), 3.11–2.85 (m, 14H, C*H*_2_─NH_2_, CH═C─C*H*_2_, C*H*_pyranose_), 2.80–2.72 (m, 6H, CH═CH─CH_2_─C*H*_2_), 2.55–2.40 (m, 12H, NH─C═O─C*H*_2_), 2.36–2.27 (m, 4H, NH─C═O─C*H*_2_). HR MS (ESI^+^) *m/z* calc. for C_91_H_144_N_23_O_41_S_2_ [M+3H]^3+^ 759.6438; found 759.6430. Yield: 82 mg (36%).

##### Glc(1,3,5)-Tyr(4-SO_3_H)( 2,4)-6-FITC (13c):

^1^H NMR (300 MHz, deuterium oxide) *δ* [ppm]: 789–777 (m, 4H, triazole-C*H*, FITC C*H*), 770–758 (m, 1H, FITC C*H*), 731–714 (m, 9H, aromatic C*H*, FITC C*H*), 6.82–6.67 (m, 4H, FITC C*H*), 4.88–4.84 (m, 3H, C*H*_anomer_Glc), 4.64–4.53 (m, 1H), 4.12–3.98 (m, m, 3H, C*H*_pyranose_), 3.95–3.13 (m, 55H, Tyr C*H*, Tyr C*H*_2_, C*H*_pyranose_, C*H*_2 pyranose_, O─C*H*_2_−, C═ONH─C*H*_2_), 3.02–2.81 (m, 14H, CH═C─C*H*_2_, C*H*_pyranose_), 2.79–2.63 (m, 6H, CH═CH─C*H*_2_─C*H*_2_), 2.56–2.24 (m, 16H, NH─C═O─C*H*_2_). ^13^C NMR (75 MHz, deuterium oxide) *δ* [ppm]: 215.95, 174.91, 174.74, 166.67, 158.04, 150.42, 148.46, 146.96, 138.65, 130.53, 121.72, 98.17, 73.14, 71.97, 71.43, 69.77, 69.29, 69.28, 60.32, 54.57, 50.13, 47.10, 37.54, 37.13, 32.16, 31.11, 30.89, 20.83. HR MS (ESI^+^) *m/z* calc. for C_112_H_155_N_24_O_46_S_3_ [M+3H]^3+^ 889.3224; found 889.3218. Yield: 5 mg (25%).

EDS-FITC (14): ^1^H NMR (300 MHz, deuterium oxide) *δ* [ppm]: 8.34 (s, 1H, FITC-C*H*), 783 (s, 1H), 739 (s, 1H, FITC-C*H* H), 6.88 (s, 2H, FITC-C*H*), 6.39 (d, *J* = 29.0 Hz, 4H, FITC-C*H*), 3.75–3.10 (m, 14H), 3.02–2.88 (m, 2H), 2.49–2.16 (m, 4H). ^13^C NMR (75 MHz, deuterium oxide) *δ* [ppm]: 215.96, 175.75, 174.69, 170.78, 69.89, 69.84, 69.06, 39.45, 36.97, 31.13, 31.04, 30.90, 30.25, 29.99, 29.73, 29.47, 29.21. HR MS (ESI^+^) *m/z* calc. for C_33_H_39_N_5_O_9_S [M+2H]^2+^ 340.6229; found 340.6233. Yield: 56 mg (82%).

### 2.3. ELISA-Inspired Inhibition Studies

ELISA-type inhibition studies were performed according to an already established protocol by Elling and co-workers.^[30]^ Inhibition of Gal-1 and Gal-3 binding to asialofetuin was investigated for glycoligands **1b**–**13b** (and **8a** for Gal-1) with final concentrations between 0.1 × 10^−6^ and 2000 × 10^−6^ m using phosphate buffered saline (PBS) buffer (150 × 10^−3^ m NaCl, 50 × 10^−3^ m NaH_2_PO_4_, pH 75). All measurements were performed two times in triplicates.

### 2.4. SPR-Inhibition Studies

The SPR-inhibition studies were performed on a lactose-functionalized CM5 senor chip on a Biacore X100 from GE Healthcare Life Science. The lactose-functionalized CM5 chip was prepared using the “surface preparation wizard” for the sensor chip CM5. An amine-coupling procedure with NHS (*N*-hydroxysuccinimide) /EDC (1-ethyl-3-(3-dimethylaminopropyl) carbodiimide; contact time 420 s, flow rate 10 μL min^−1^) was used for the functionalization of the two flow cells. Therefore, flow cell 1, as reference cell was blank immobilized with ethanolamine according to the software. For flow cell 2 (mess cell), 1 × 10^−3^ m Lac(1,3,5)-6, **2a**, in HBS-P buffer from GE Health-Care was used with a contact time of 600 s. As running buffer, HBS-P buffer from GE Healthcare was used. The immobilization levels reached were 411 RU for flow cell 2 and 186 RU for flow cell 1. The inhibition assay was performed in a “Custom Assay Wizard-Binding Analysis” in a multi cycle measurement. For the inhibition studies, stock solutions of 100 × 10^−6^ m of each ligand and 200 μg mL^−1^ of Gal-3 both in PBS buffer (150 × 10^−3^ m NaCl, 50 × 10^−3^ m NaH_2_PO_4_, pH 7.5) were prepared. Gal-3 was incubated with each ligand by mixing the solutions of the protein and ligands in a 1:1 ratio, resulting in final concentrations of 100 μg mL^−1^ for Gal-3 and 50 × 10^−6^ m for the ligands. PBS was used as running buffer during the measurements choosing an association time of 90 s and a dissociation time of 60 s with a flow rate of 10 μL min^−1^ over both flow cells. For regeneration of the cell surface, 3 m MgCl_2_ in MilliQ water was injected for 60 s with a flow rate of 10 μL min^−1^ after each cycle. The evaluation was performed using the evaluation software provided by GE Healthcare. The response unit of the Gal-3 binding event without and after incubation with the ligands was taken 155 s after start of the sample injection. The response unit (RU) for only Gal-3 represents the 100% binding and 0% inhibition event. The value of the inhibition with the glycomacromolecules was referred to the response unit of only Gal-3. All measurements were performed in triplicates.

### 2.5. Cell Lines and Tissue Culturing

Tissue culture was performed in a SterilGARD III Advance SG 603 laminar flow hood from Baker Company. Cell cultures were observed using a Zeiss Axiovert 25 inverted microscope. All cell lines and culture media were purchased from ATCC. Cell line HEK-293 (#CRL-1573) was grown in Eagle’s minimum essential medium (EMEM) (# 30–2003) and supplemented with 10% fetal bovine serum (FBS) and 1% Pen/Strep. Cell line MCF7 (ATCC HTB-22) was cultured in EMEM with 0.1 mg mL^−1^ insulin, 10% FBS, and 1% Pen/Strep. Cells were cultured in 75 cm^2^ tissue culture flask from TPP at 37 °C and 5% CO_2_ in a CO_2_ water jacketed incubator 3110 from Scientific Inc. Once weekly, the medium was changed and the trypsinization of confluent cells was performed with trypsin-ethylenediaminetet- raacetic acid solution from ATCC (#30-2101) for subculturing as recommended by the supplier. Cell counting was performed with a 2%-trypan-blue solution in PBS from VWR and dispos-able hemacytometers from Incyto C-chip.

### 2.6. Flow Cytometry

#### 2.6.1. Galectin-Antibody Analysis with Flow Cytometry

Human galectin-1 biotinylated antibody, human galectin-3 biotinylated antibody, and goat IgG biotinylated antibody as isotype control were purchased from R&D systems (#BAF1152, BAF1154, and BAF108). For surface staining, cells were suspended at 5 × 10^6^ cells mL^−1^ in Dulbecco’s phosphate-buffered saline buffer containing 0.1% w/w bovine serum albumin (BSA) and 0.1% w/w sodium azide. 100 μL of the cell suspension (500 000 cells) were incubated with 3 μL of human BD Fc block (#564220) from BD biosciences for 10 min at room temperature. Without washing in between, either 3 μL of the biotinylated human galectin antibodies or the isotype control (each 0.5 μg μL^−1^ stock solution) was added and the cells were incubated for an additional 1 h on ice. After that, the cells were washed three times with cooled PBS+ buffer (containing BSA and sodium azide) by centrifugation at 780 rpm and 4 °C for 5 min, followed by resuspension of the pellet in PBS. After the last centrifugation step, the cell pellet was resuspended in 100 μL of PBS+ and the cells were stained with 10 μL of a 0.002 μg mL^−1^ solution of Streptavidin-PE conjugate for 20 min at room temperature. The cells were washed three time with on ice cooled PBS+, followed by the resuspension of the cell pellet in 300 μL of PBS+ buffer for the flow cytometry measurements using the Accuri C6 flow cytometer. For intracellular staining, the cells were fixed before staining using a Fixation/Permeabilization Solution Kit from BD Bioscience. Therefore, a cell pellet of 500 000 cells was suspended in 1 mL Fix/Perm. Solution provided by the kit for 20 min on ice. Cells were washed three times with 1 mL BD Perm/Wash Buffer. After that, the staining was performed as described for the surface staining using permeabilization buffer for the washing steps in between to ensure permeability. A total of 100 000 cells were counted with a medium flow rate. Evaluation of the FACS result was performed with the FCS Express 4 program.

#### 2.6.2. Studies of FITC-Conjugated Glycooligomers using Flow Cytometry

The entire procedure was performed while avoiding light exposure to the samples. 500 000 cells in 90 μL were seeded into 96-well plates. 10 μL of FITC-conjugated derivatives of the glycoconjugates, prepared as 2000 × 10^−6^ and 1000 × 10^−6^ m stock solutions in water, were added to the cells resulting in a total volume of 100 μL containing 200 × 10^−6^ and 100 × 10^−6^ m of the ligands, respectively. After incubation of the cells for 3 h at 37 °C and 5% CO_2_, the content of each well was transferred to a centrifuge tube and the well was washed one time with 200 μL PBS, which was afterward transferred to the same corresponding centrifuge tube. After washing the cells three times with 1 mL PBS buffer, the cells were fixed with 100 μL of a Cytofix solution for 20 min on ice. Fixed cells were washed three times with 1 mL PBS+ before measuring with an Accuri 6 flow cytometer. 20 000 cells were collected with a slow flow rate of 14 μL min^−1^. In between samples, two washing steps were performed at a high flow rate of 66 μL min^−1^ for 1 min, the first of which involved a bleach solution containing 4% of hypochlolrite, followed by MilliQ water. A slow flow rate and the washing steps were needed to avoid clogging the system.

### 2.7. Fluorescence Microscopy

Cells were grown on 24 mm cover slips (# 229174) in 6-well plates (#229106) purchased from Celltreat. Cover slips were first coated with a 0.01% poly-L-lysine solution from Sigma Aldrich for 5 min, followed by three washing steps with sterile MilliQ water. Cover slips were transferred into 6-well plates and allowed to dry at room temperature for 2 h. The cell lines were trypsinized, counted, centrifuged, and resuspended to a final concentration of 70 000 cells mL^−1^ in the corresponding total growth medium. Next, 3 mL of the cell suspension was transferred to each well. The cells were grown on the cover slips for 2 days at 37 °C at 5% CO_2_. The staining procedure was performed in petri dishes (60 mm x 15 mm) from Fisher Brand. After removing the cell medium, the cover slips were carefully washed twice with prewarmed PBS and transferred into a new clean petri dish for staining. First, the staining with FITC-labeled glycoconjugates was performed. Cover slips were incubated with 200 μL of 200 × 10^−6^ m FITC-labeled glycoconjugate solution for 3 h at 37 °C and 5% CO_2_. The supernatant was removed, and the cover slips were washed three times with PBS. As a negative control, the glycoconjugate staining was skipped. The cover slips were then transferred into small petri dishes for the permeabilization with a solution of 0.1% Tween 20 in PBS for 5 min at room temperature. The cover slips were washed two more times with PBS and then subjected to reference staining. For reference staining, Hoechst 33342 (#H1399) staining for the nucleus and Alexa Fluor 647 Phalloidin (#A22287) staining for actin were used; both were purchased from Invitrogen. The dilutions were prepared as recommended by supplier: 100 mg of 33342 Hoechst was dissolved in 10 mL deionized H_2_O. 5 μL of 33342 Hoechst stock solution was further diluted in 10 mL PBS (1:2000 ratio). The content of the Alexa Fluor 647 Phalloidin vial was dissolved in 1.5 mL methanol and further diluted by mixing 5 μL of the phalloidin stock solution in 200 μL PBS. For staining, 200 μL of phalloidin staining and 200 μL of Hoechst staining solution were simultaneously added on the cover slips and coincubated for 40 min at room temperature. The supernatant was removed and the cover slips were washed three times with PBS. TrueBlack Lipofuscin autofluorescence quencher from Biotium (#23007) was diluted by mixing 50 μL of the 20 x stock solution in 1 mL 70% ethanol right before usage. The cover slips were coated with the dilution for 30 s. After that, the cover slip was washed carefully three times with PBS buffer, freed from an excess of liquid by dapping on dust-free tissue and flipped onto glass slides for microscopy, using one drop of ProLong Gold as antifade mountant (#P10144). Fluorescence microscopy was performed with an Olympus DP80 coupled with Prior Scientific Launches L200S fluorescence illumination system.

### 2.8. MTT Cell Viability Assay

An MTT (3-(4,5-dimethylthiazol-2-yl)-2,5-diphenyltetrazolium bromide) Assay Kit was purchased from Abcam (#ab211091). Cells were seeded into 96-well plates with a volume of 90 μL per well and 40 000 cells for the HEK 293 cell line and 30 000 cells for the MCF 7 cell line in complete growth medium. 10 μL of amine derivatives of the glycoconjugates, prepared as 2000 × 10^−6^ m stock solutions in water were added to the cells resulting in a total volume of 100 μL containing 200 × 10^−6^ m of the ligands. After incubation of the cells for 48 h at 37 °C and 5% CO_2_, the plates were centrifuged at 1000 rpm, 4 °C for 5 min. 60 μL of the supernatant was carefully removed, followed by the addition of 50 μL PBS and 50 μL MTT reagent. After incubation for 3 h at 37 °C and 5% CO_2_, the plates were centrifuged and 75 μL of the supernatant was removed. The purple precipita-tion was then dissolved by adding 200 μL of dimethyl sulfoxide to each well and shaking the plate for 3 h at room temperature covered with aluminum foil. The plates were read out with a Synergy H1 microplate reader from Biotek at 590 nm without a lid.

### 2.9. Scratch Wound In Vitro Test

Cells were seeded at 400 000 cells per well in 12-well plates with a final volume of 1 mL cell culture medium. Cells were grown for 48 h until a dense monolayer was reached. A wound field was created with a 10 μL pipet tip using a line on the back of the plate as guide. After creation of the wound field, the medium was removed and replaced by 360 μL total growth medium and 40 μL of a 2000 × 10^−6^ m glycoconjugate or lactose solution in MiliQ water resulting in a final glycoconjugate concentration of 200 × 10^−6^ m. For the unstained control, 400 μL complete growth medium and for the vehicle control 360 μL complete growth medium and 40 μL of MiliQ water were added. For the dosing experiments, an extra 30 μL of complete growth medium and 10 μL of the 2000 × 10^−6^ m glycomacromolecule solution was added after 12, 24, 36, and 48 h. For the migration experiments, the amine derivatives of the glycomacromole- cules were used, as the direct precursor of the FITC derivatives, used in the other cell studies. Pictures were taken with a Nikon Eclipse TS100 using a Nikon E Plan 4 × 0.10 ∞ /WD 30 Microscope Objective and coupled with a Nikon ELWD 0.3/OD75 condenser. Distances of the wound field were analyzed with the free software ImageJ.

## 3. Results and Discussion

In this study, we endeavored to better understand the role of nonglycosidic motifs in binding galectin-3. Therefore, we synthesized a series of glycomacromolecules of similar size and molecular weight, bearing either lactose alone (homomultivalent) or lactose alternating with several nonglycosidic motifs (heteromultivalent). Nonglycosidic motifs were designed to present aromatic residues with either neutral, amine, or sulfonated/sulfated groups to explore the role of these groups on galectin-3 binding.^[75]^

**Scheme 1.**
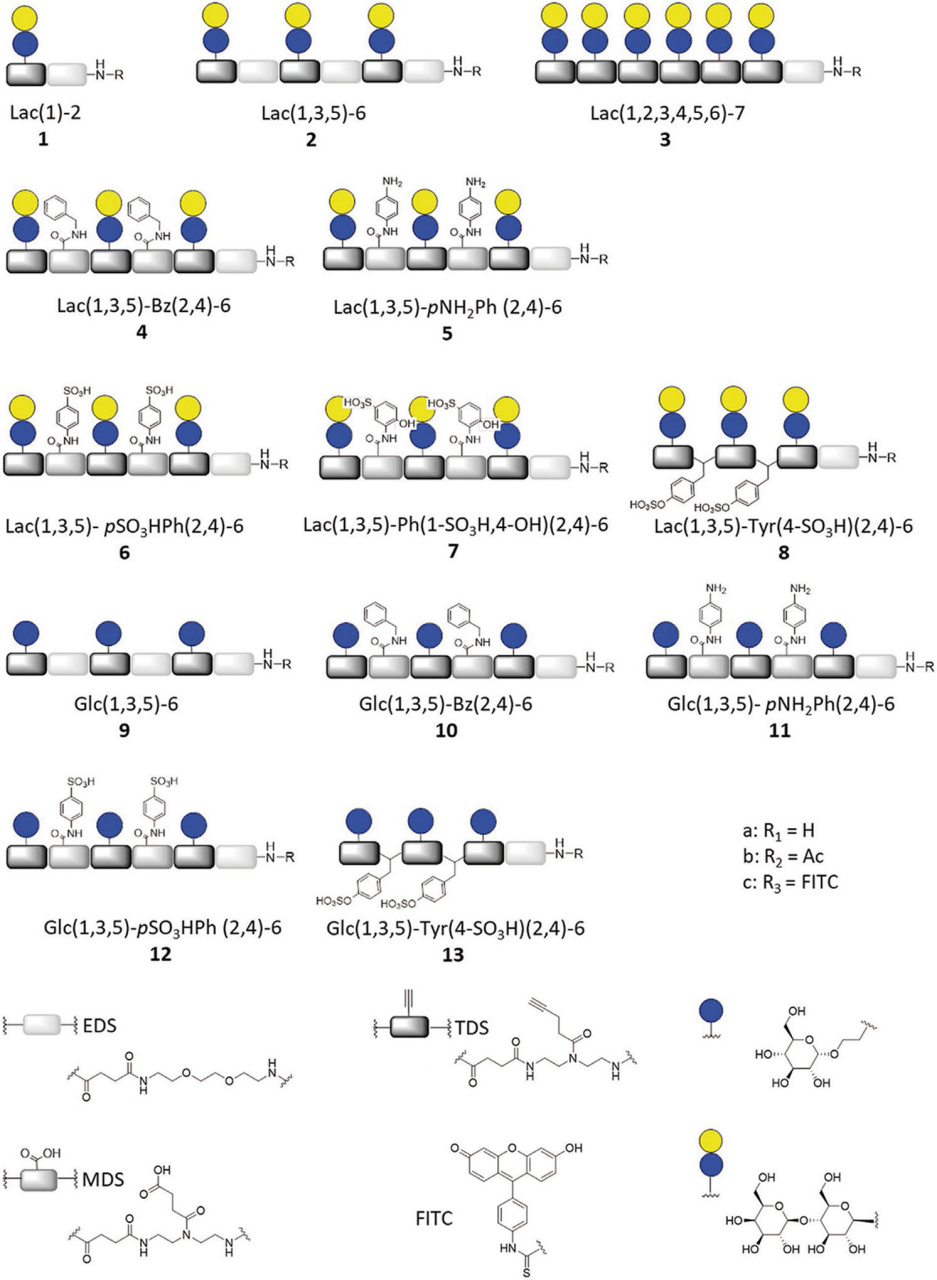
Overview of synthesized structures. The terminal number for each glycomacromolecule represents the overall number of combined building blocks (EDS, TDS, and MDS).

SPPoS was used to synthesize lactose-based glycomacro-molecules **1**–**8** as potential ligands for galectin-3, and glucose derivatives **9**–**13** as nonbinding controls (Scheme 1). SPPoS uses tailor-made building blocks for the stepwise assembly of monodisperse, sequence-defined oligo(amidoamines) on solid support by applying standard Fmoc-peptide coupling protocols. The building blocks used for this study include: TDS^[69]^ for introducing an alkyne moiety in the side chain that can be used for site-selective conjugation of azido-functionalized carbohydrates via copper-catalyzed azide-alkyne cycloaddition (CuAAC), MDS^[71]^ for introducing a carboxylic group in the side chain for conjugation via amide coupling, and EDS^[70]^ for introducing an ethylene glycol motif in the main chain. Homomultivalent glycomacromolecules **1**–**3**, and **9** were synthesized according to previously established methods using TDS and EDS (see the Supporting Information), and vary in the number of glyco-sidic residues.[^69,70,76]^ With the exception of **1** and **3**, which were designed to represent mono- and higher valent analogs, respectively, all heteromultivalent glycomacromolecules carry three glycosidic residues and two nonglycosidic moieties.

Heteromultivalent glycomacromolecules were synthesized by replacing the EDS building blocks of compound **2** with MDS.^[71]^ The carboxylic side chain of the MDS building block and the alkyne moiety of the TDS building block enabled orthogonal post-modification of the scaffolds via amine coupling and CuAAC, respectively. As nonglycosidic moieties, aryl residues bearing different amine or sulfonic acid and sulfonate function-alities were used.^[77]^ For Lac(1,3,5)-Ph(1-SO_3_H,4-OH)(2,4)-6,**7**, attachment of the nonglycosidic sidechain after carbohydrate conjugation as described above was unsuccessful, potentially due to steric hindrance of the lactose residues. Therefore, the synthetic route was altered to reverse the amide and CuAAC coupling steps. This strategy resulted in the desired product after deprotection, cleavage, and purification (see Figures S57-S60, Supporting Information). Furthermore, shorter sulfated heterostructures **8** and **13** were synthesized using Fmoc-L-Tyr(4-SO_3_H)-OH instead of the MDS building block, by applying standard protocols to give the desired products after deprotection, cleavage, and purification.

The final glycomacromolecules were deprotected, cleaved from the resin, purified by ion-exchange chromatography, and preparative RP-HPLC, and isolated with high purities (as determined by RP-HPLC analysis) (see Figures S2-S110, Supporting Information). Notably, glycomacromolecules were synthesized with different end-functionalities: free (subgroup a) and capped amine (subgroup b) or FITC-conjugated derivatives (subgroup c) for different studies (Scheme 1).

To investigate the binding avidities of the heteromultivalent glycomacromolecules as ligands for galectin-3, competitive-inhibition binding studies were performed. These studies test the ability of the glycomacromolecules to competitively inhibit the binding of galectin-3 to the glycoprotein asialofetuin in an ELISA-type assay. We chose to compare the results from our galectin-3 studies to studies with galectin-1 to determine preferential binding between these two lectins, which have a conserved carbohydrate recognition domain (CRD), but are members of different galectin classes. The ELISA-type assay performed gives the half-maximum inhibitory concentration (IC_50_) of each ligand as a measure of avidity toward galectin-3 or galectin-1, where lower IC_50_ values correspond to higher avidity (Table 1).

As expected, for studies with galectin-3, homomultivalent structures show increased binding with increasing valency, while studies with galectin-1 revealed only small changes in binding with increasing valency. This is expected since galectin-3 forms pentameric oligomers and galectin-1 is known to form dimers.^[78]^ However, when comparing trivalent homomultivalent glycomacromolecule **2b** lacking aromatic residues with trivalent heteromultivalent analogs **4b**–**8b** bearing aromatic residues, binding to galectin-3 increased, while binding to galectin-1 decreased.

For better comparison, IC_50_ values were normalized to the IC_50_ value of free lactose giving the relative inhibitory potential (Table 1: RIP) with respect to free lactose. Heteromultivalent glycomacromolecules containing aromatic residues and three lactose units (**4b**–**8a/b**) revealed a trend of increased binding to galectin-3 by a factor of 1.5- to 3-fold, and decreased binding with galectin-1 by a factor of 1.5- to 2-fold in comparison to **2b**. Notably, more significant increases in binding were observed for glycomacromolecules bearing sulfonated (**6b** and **7b**) and sulfated (**8b**) aromatic motifs in binding studies with galectin-3 in comparison to galectin-1. In addition, no statistically significant differences for different end groups were noted demonstrating that both capped and free amines resulted in similar outcomes for the compounds tested. One explanation for the increased binding results observed with galectin-3 is that hydrophobic interactions may be forming between the Galectin-3 CRD and the aromatic motifs of the glycomacromolecules. Preliminary support for this hypothesis is provided by previous studies focusing on the role of aromatic residues in galectin-3 binding^[79-81]^ However, future studies involving 15N-^1^H HSQC (heteronuclear single quantum coherence) experiments would need to be performed to confirm specific binding interactions within the binding pocket.

**Figure 1.**
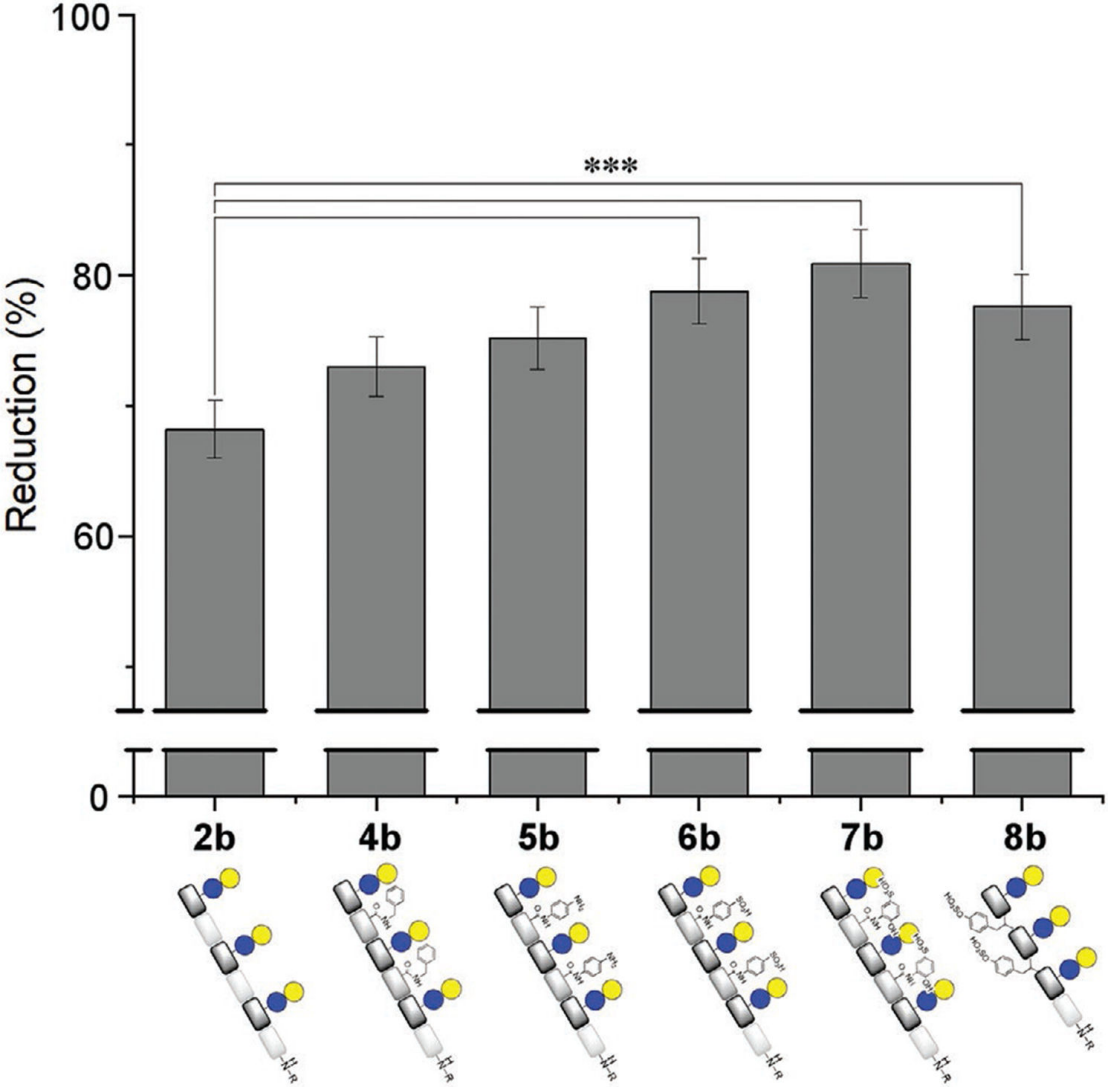
Results from the SPR inhibition studies of galectin-3 and samples **2b–8b**. Values are calculated as a percent reduction in galectin-3 binding to a fixed ligand on an SPR chip. Statistical significance is indicated at the 99% CI (***). For inhibition studies, stock solutions of 100 × 10^−6^ m of each ligand and 200 μg mL^−1^ of Gal-3 both in PBS buffer (150 × 10^−3^ m NaCl, 50 × 10^−3^ m NaH_2_PO_4_, pH 7.5) were prepared. Galectin-3 was incubated with each ligand by mixing the solutions of the protein and ligands in a 1:1 ratio, resulting in final concentrations of 100 μg mL^−1^ for galectin-3 and 50 × 10^−6^ m for the ligands prior to analysis with a lactosefunctionalized CM5 chip.

Similar trends were observed by applying the aforementioned glycomacromolecules in SPR bind assays.^[82]^ For SPR experiments, a fixed concentration of galectin-3 and ligand was used to determine the ability of each ligand to reduce galectin-binding to a lactose-functionalized CM5 chip. A statistically significant reduction in binding was observed between sul-fonated and sulfated derivatives (**6b**–**8b**) in comparison to **2b** indicating that these glycomolecules had a greater inhibitory effect than compounds **4b** and **5b** (Figure 1). This finding is in accordance with studies investigating the influence of negatively charged glycans on galectin-3 binding which showed that higher affinities could be obtained in comparison to uncharged glycans.^[29,31^,^32]^ No statistical significance was observed between the binding of **5b** (the best nonsulfonated/sufated binder) and **6b/8b**. As expected, when replacing the binding carbohydrate ligands (lactose) with a nonbinding carbohydrate ligand (glucose, **9b**-**13b**), we see no inhibition in both ELISA and SPR studies (see Figures S111-S117, Supporting Information) confirming that the lactose is required for binding to galectin-3 and that only the specific combination of lactose and sulfonated or sulfated nonglycosidic motifs leads to higher avidity ligands.

To further investigate the effect of glycomacromolecules as ligands of galectin-3, we performed in vitro cell studies with a human breast cancer cell line MCF 7 which is known to overexpress galectin-3.^[83]^ Immunostaining of untreated live cells confirmed that MCF 7 cells exhibit surface expression of galectin-3 and no expression of galectin-1, while internal staining of fixed, permeabilized MCF 7 cells revealed increased staining, demonstrating that a substantial intracellular reserve of galectin-3 is present in these cells (Figure S118, Supporting Information).

**Figure 2.**
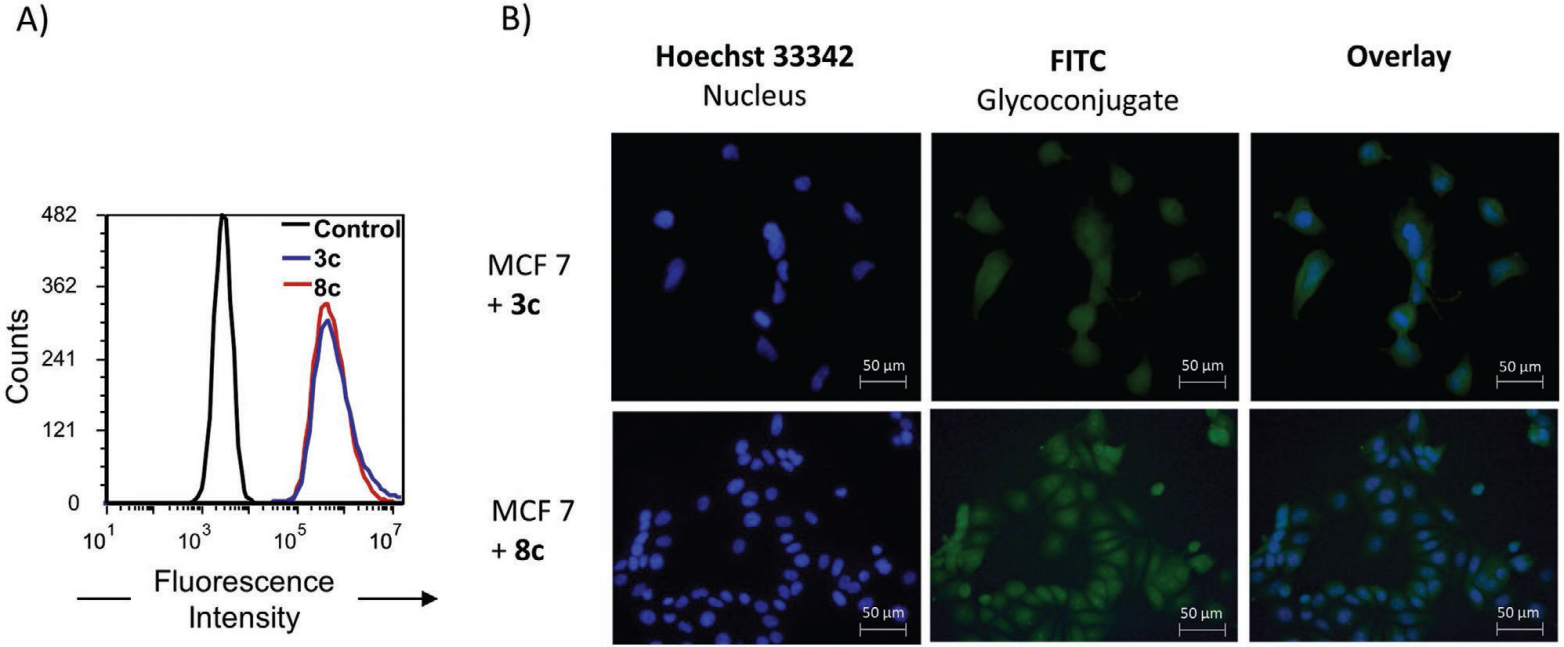
Interaction and localization studies of FITC-derivatives **3c** and **8c** on MCF 7 cells. A) Flow cytometry histogram of the FITC-channel for blank cells and cells stained with 200 × 10^−6^ m **3c** (red) and **8c** (blue). B) Fluorescence images of MCF 7 cells stained with 200 × 10^−6^ m **3c** and **8c** (3 h at 37 °C) and as references Hoechst 33342 staining for nucleus localization.

As a prerequisite for further testing, cell toxicity of glycomacromolecules selected for cell experiments (**1**–**3**, **6**, **8**,**9**,**12**–**14**) was determined via MTT cell viability assay. Results demonstrated no detectable differences in viability between the vehicle control and cells treated with the glycomacromolecules after 48 h incubation (Figure S119/120, Supporting Information). To test the general ability of glycomacromolecules to associate with the cells, flow cytometry studies were performed using FITC-conjugated derivatives **1c**–**3c, 6c, 8c, 9c, 12c, 13c** at two different concentrations. After fixation of the cells, analysis of stained cells via flow cytometry showed a dose-dependent staining for both cell lines for all compounds (Figure S121/122, Supporting Information). The lack of a significant difference in the mean-FITC values suggests a nonse-lective association (Figure 2A).

Preliminary fluorescence microscopy studies were then performed to analyze localization of the glycomacromolecules. Exemplary comparison of compounds **3c** (**3b** as best homomul-tivalent binder) and **8c** (**8b** as best heteromultivalent binder) shows a general staining (Figure 2). However, for compound **8c**, there appears to be an enrichment of fluorescence around the nucleus in comparison to the cytosol. A similar pattern was observed for cells stained with **6c**, **12c**, and **13c** (Figures S123-S127, Supporting Information). This seems to be related to the presence of the aryl sulfonated/sulfated motifs. One possible explanation for this finding is that the negative charge of these motifs might lead to an attractive interaction with positively charged nucleoporins.^[84]^ However, at this point, it is not possible to determine the exact nature of the glycomacromolecule-cellular association.

**Figure 3.**
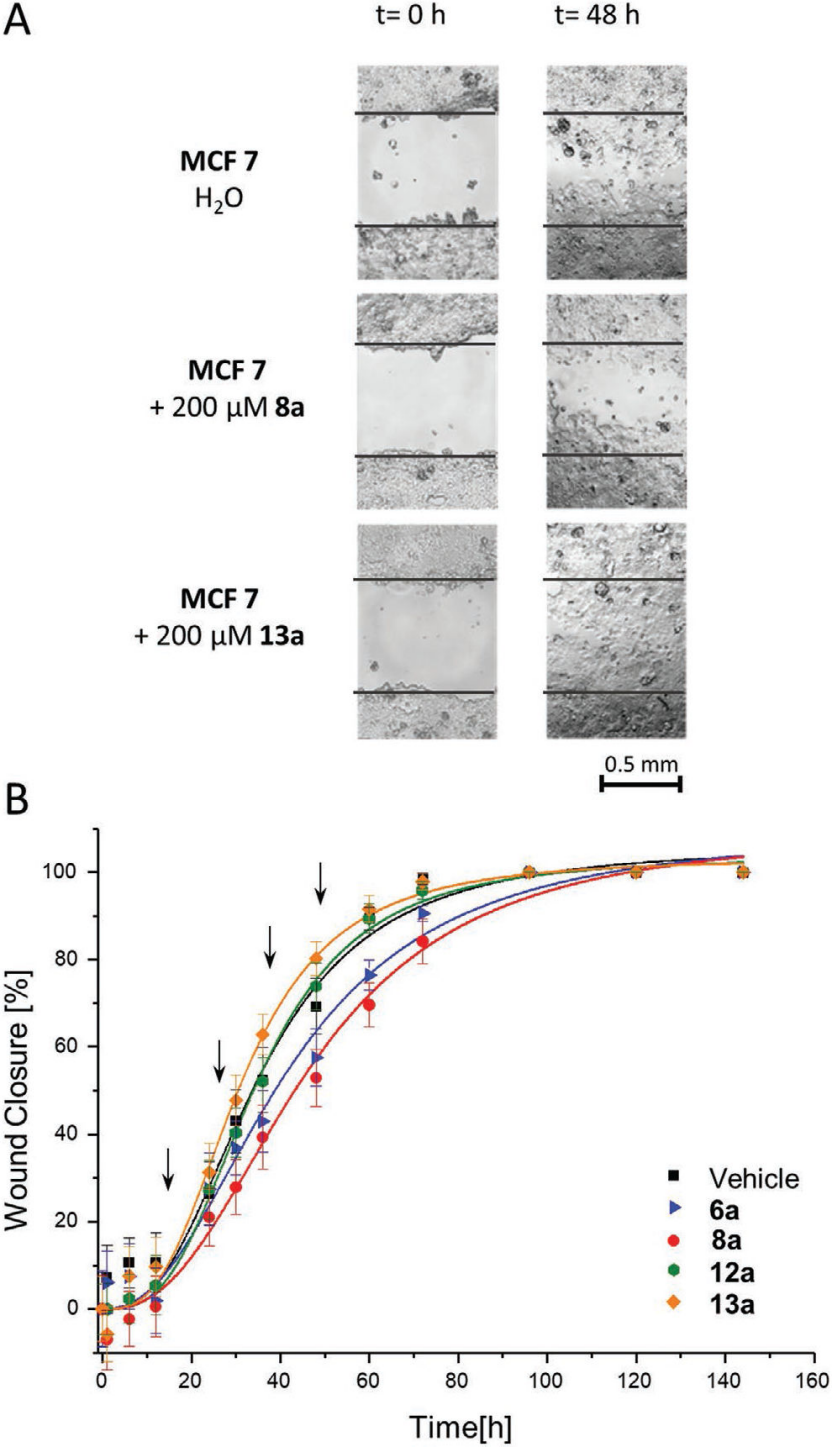
In vitro wound scratch assay with a galectin-3 positive MCF 7 cell line. A) Exemplary images of the wound area at time point *t* = 0 h and *t* = 48 h for MCF 7 treated with the vehicle control H_2_O (top), lactose structure **8a** (middle), and corresponding glucose structure **13a** (bottom). B) Results of the time-dependent wound closure for dosing tests with compounds **6a, 8a, 12a**, and **13a** (for more data, see Figures S128-S137, Supporting Information).

Since the tested glycomacromolecules showed no cytotoxic behavior and positive association with the cells, we decided to study their influence on wound closure, which is known to be mediated by galectin-3.^[85-87]^ This was accomplished by performing an in vitro scratch wound assay as described by Dion and co-workers^[88,89]^ using compounds **1a**–**3a**, **6a**, **8a**–**9a**, and **12**–**13a**. These compounds represent the direct precursor of the FITC derivatives. Cells were cultivated as a monolayer and a “wound field” was created (Figure 3A). Cells were then incubated with the glycomacromolecules, and the width of the wound field was observed under an inverted microscope. The distance analysis at different time points was used to create a wound closure curve (Figure 3B). In this model, ligand binding to galectin-3 is expected to result in a reduction in wound closure over time.

In this study, different effects were observed for wound closure for the different glycomacromolecules (Figures S128-S137 and Tables S1-S3, Supporting Information). Generally, glucose-functionalized glycomacromolecules resulted in a slightly faster wound closure^[90]^ In comparison, the lactose derivatives led to a delayed wound closure, especially sulfonic acid derivative **6a** and sulfated derivative **8a**. For example, after 48 h compound **8a** differed with 57 ± 5% an almost 20% difference from the corresponding glucose derivative **13a** showing 76 ± 5% closure (Table S2, Supporting Information). These results are in agreement with similar studies on other ligand systems targeting lectins involved in cell migration.^[77,86-89]^ For example, Dion and coworkers observed a delay in wound healing of around 20% for the treatment of keratinocytes with lactosamine-based (2-naphthyl)methyl compounds inspired by TD139, which is currently in clinical trials.^[57,88,89]^ Raman and co-workers achieved a delay of 20–30% through the incubation of MCF 7 cells with a nucleoside analog addressing RNA helicase.^[91]^

To examine the effects of sustained exposure of the glyco-macromolecules on the MCF 7 cells, we performed a “dosing” study by introducing additional aliquots of glycomacromolecules **6a**, **8a**, **12a**, and **13a** after 12, 24, 36, and 48 h giving in total an additional 100 mol% (Figure 3b). In this experiment, the differences in wound closure were even more significant, yielding delays of 53 ± 7% for **8a** compared to 80 ± 4% for **13a** after 48 h (Figures S131, S135, and Table S3, Supporting Information) indicating a dose-specific response, further demonstrating that glycomacromolecule ligands with higher inhibition potentials in the ELISA and SPR studies delayed wound closing in a galectin-3 cell line. Negative controls replacing lactose by glucose side chains showed no effects in similar studies, confirming that it is the combination of carbohydrate and nonglycosidic motifs that enables high avidity and selective binding.

While our preliminary results demonstrate the potential for the aforementioned glycomacromolecules to serve as preferential ligands for galectin-3, additional studies including the use of additional cell lines as well MCF 7 cells transfected with galectin-3 siRNA are necessary to further confirm the involvement of galectin-3 in delaying wound closure as observed in this study.

## 4. Conclusion

In conclusion, we introduced the synthesis of heteromulti-valent lactose-functionalized glycomacromolecules bearing nonglycosidic motifs to investigate the impact of these motifs on galectin-3 binding. The aforementioned structures were successfully tested for binding to galectin-3 using ELISA and SPR. Results revealed that the incorporation of nonglycosidic motifs, especially sulfonated or sulfated motifs, could be used to achieve RIPs that were similar to more highly glycosylated structures demonstrating that binding could be modulated by nonglycosidic components. An in vitro wound scratch assay using a galectin-3 positive MCF 7 breast-cancer cell line further revealed that structures containing sulfonated and sulfated nonglycosidic motifs delayed wound closing by almost 20%. This work demonstrates the potential for heteromultivalent glycomacromolecules bearing nonglycosidic sulfonated or sulfated motifs to serve as preferential ligands for galectin-3 in comparison to analogs without these motifs. Future studies will involve the investigating heteromultivalent glycomacromolecules-lectin binding with 15N-^1^H HSQC to better understand the nature of the binding events investigated here as well as with other members of the galectin family. In addition, we plan to investigate the biological effects of the glycomacromolecules-bearing sulfonated/sulfated motifs in in vitro scratch wound assays with additional cell lines expressing galectin-3.

## Supplementary Material

Freichel_Supplementary

## Figures and Tables

**Table 1.** Results of the inhibition-competition binding studies of glycomacromolecules **1b–8b** to Gal-1 and Gal-3 in the ELISA-type assay.

Glycomacromolecule	Number of carbohydrate residues	Gal-1 IC_50_ ± SD [× 10^−6^m]^a)^	RIP_Gal-1_^b)^	Gal-3 IC_50_ ± SD [× 10^−6^m]^a)^	RIP_Gal-3_^b)^
Lactose	1	420 ± 94	1.0	159 ± 13	1
**1b**	1	296 ± 70	1.4	123 ± 3	1.3
**2b**	3	55 ± 12	7.6	38 ± 2	4.2
**3b**	6	64 ± 5	6.5	16 ± 4	9.9
**4b**	3	105 ± 14	4.0	25 ± 1	6.3
**5b**	3	134 ± 22	3.1	22 ± 2	7.4
**6b**	3	100 ± 21	4.2	16 ± 1	9.9
**7b**	3	127 ± 19	3.3	15 ± 0.3	10.5
**8a/b**	3	89 ± 18	4.7	14 ± 1	11.4

a) IC_50_ values were determined in ELISA-type inhibition studies for galectin-1 and galectin-3 binding to asialofetuin. Measurements were performed two times in triplicates;

b) Relative inhibitory potency (RIP): Calculated referring to the IC_50_ value of lactose.
