# Supplementary material for: Sequence-Defined Heteromultivalent Precision Glycomacromolecules Bearing Sulfonated/Sulfated Nonglycosidic Moieties Preferentially Bind Galectin-3 and Delay Wound Healing of a Galectin-3 Positive Tumor Cell Line in an In Vitro Wound Scratch Assay
Source: Macromol Biosci. Author manuscript; Available in PMC 2023 Jan 10. (PMC9831253; doi:10.1002/mabi.202000163)
Supplement: Freichel_Supplementary [file NIHMS1859982-supplement-Freichel_Supplementary.pdf]

© 2020 Wiley-VCH GmbH

Supporting Information

for *Macromol. Biosci.*, DOI: 10.1002/mabi.202000163

Sequence-Defined Heteromultivalent Precision Glycomacromolecules Bearing Sulfonated/Sulfated Nonglycosidic Moieties Preferentially Bind Galectin-3 and Delay Wound Healing of a Galectin-3 Positive Tumor Cell Line in an In Vitro Wound Scratch Assay

Tanja Freichel, Viktoria Heine, Dominic Laaf, Eleanor E. Mackintosh, Sophia Sarafova, Lothar Elling, Nicole L. Snyder,* and Laura Hartmann*

Supporting Information

Sequence-defined Heteromultivalent Precision Glycomacromolecules Bearing Sulfonated/Sulfated Non-Glycosidic Moieties Preferentially Bind Galectin-3 and Delay Wound Healing of a Galectin-3 Positive Tumor Cell Line in an *in vitro* Wound Scratch Assay

Tanja Freichel[a], Viktoria Heine[b], Dominic Laaf[b], Eleanor E. Mackintosh[c], Sophia Sarafova[c], Lothar Elling[b], Nicole L. Snyder [c], and Laura Hartmann* [a]*

Figures

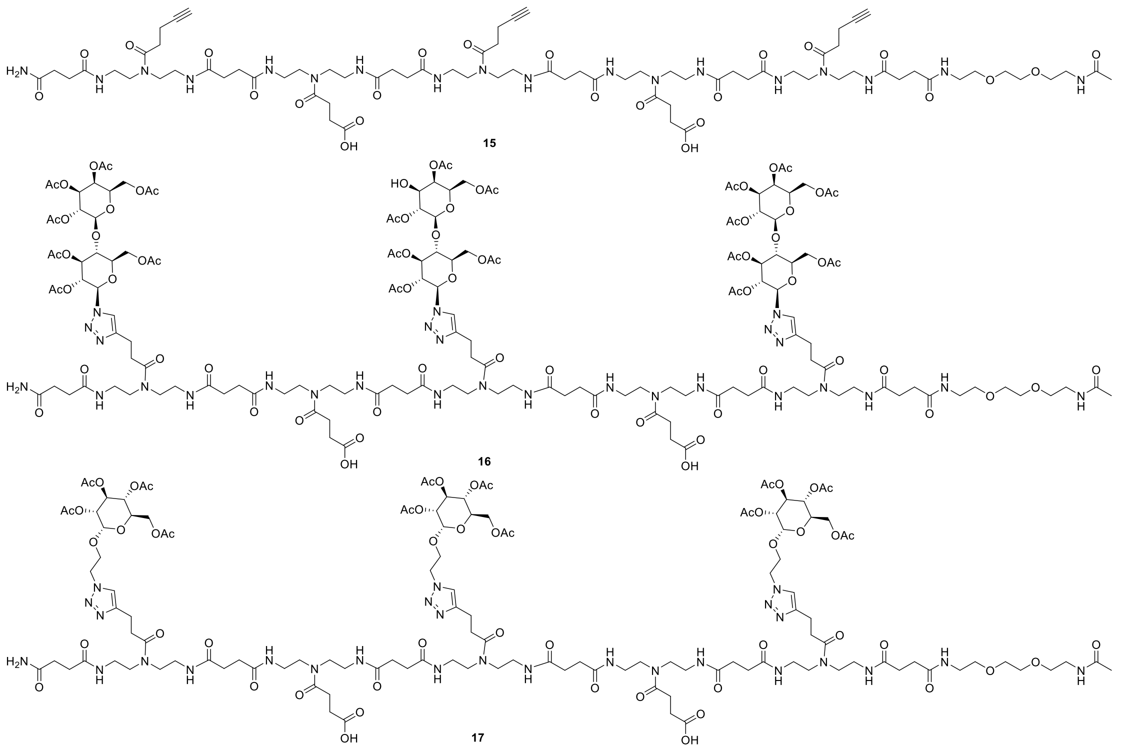

Figure S 1: Precursors for the synthesis of the heteroglycoconjugates.

Analytics Glycomacromolecules

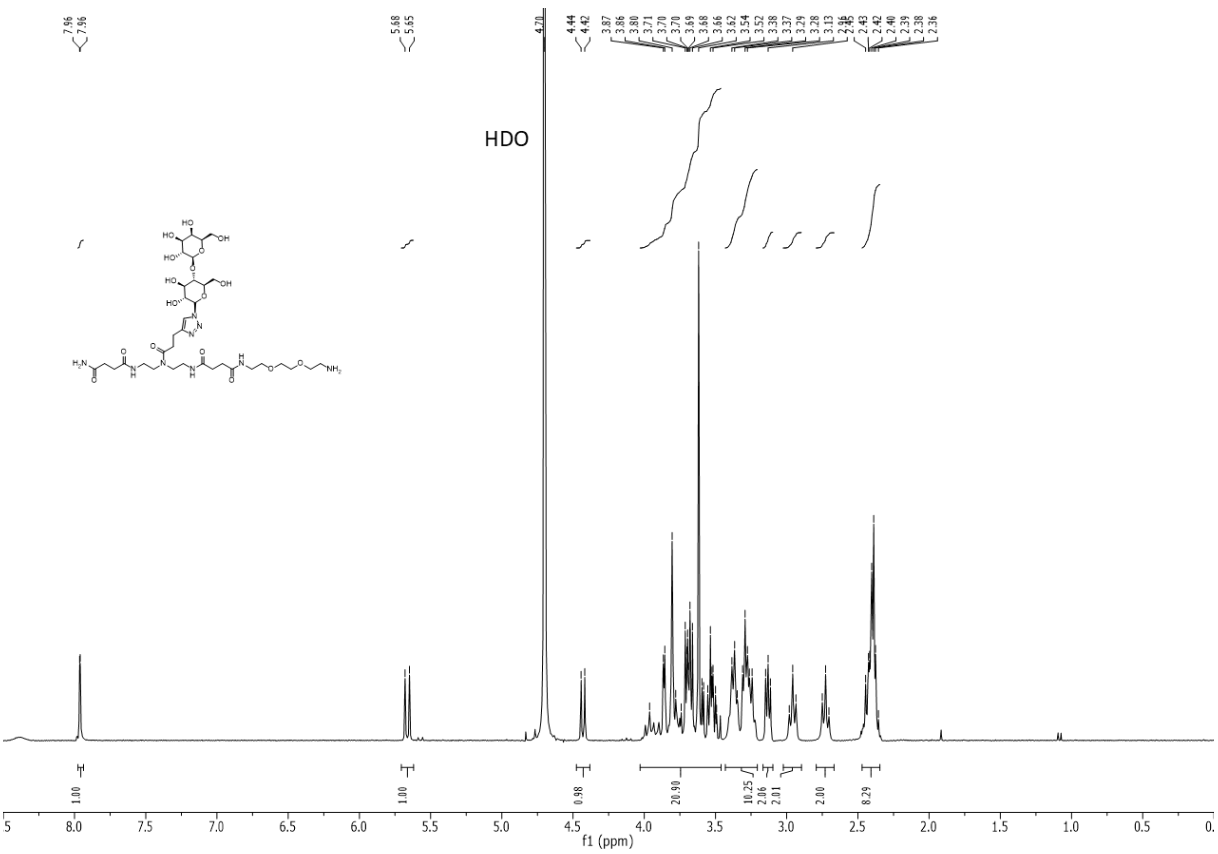

Figure S 2: ^{1}H NMR spectrum of compound **1a.**

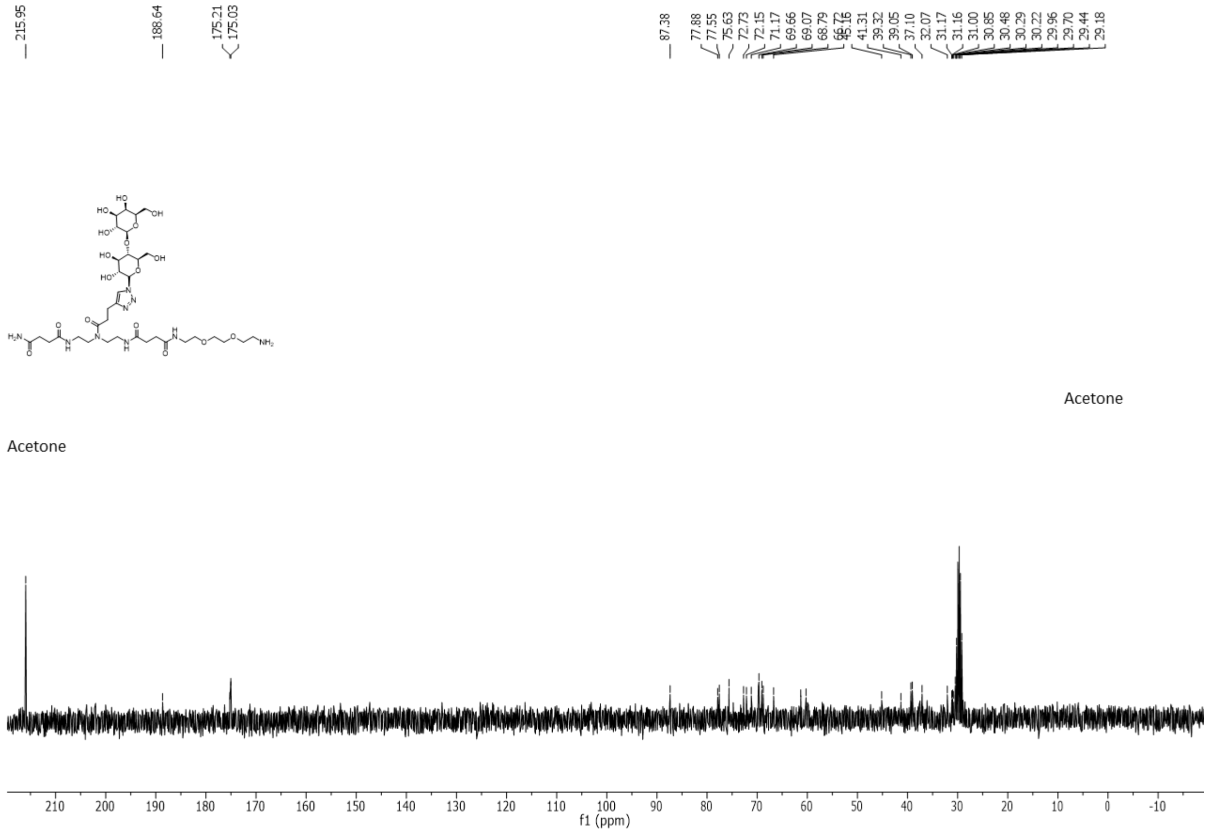

Figure S 3: ^{13}C NMR spectrum of compound **1a.**

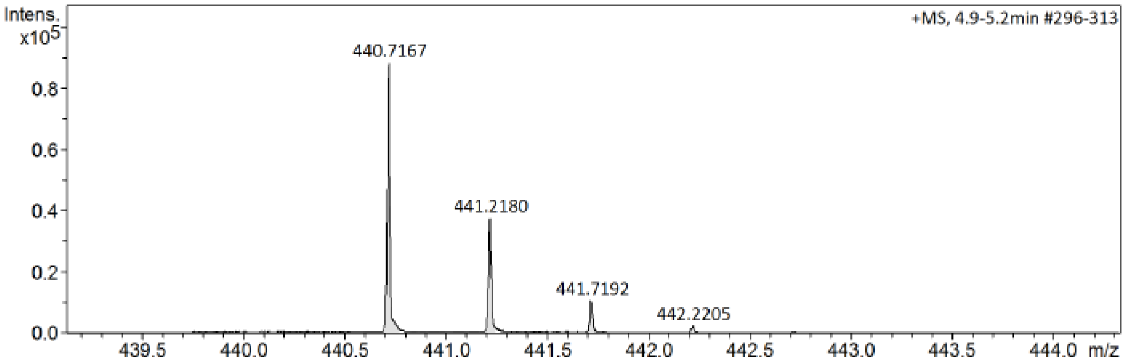

Figure S 4: HR-MS spectrum of compound **1a.**

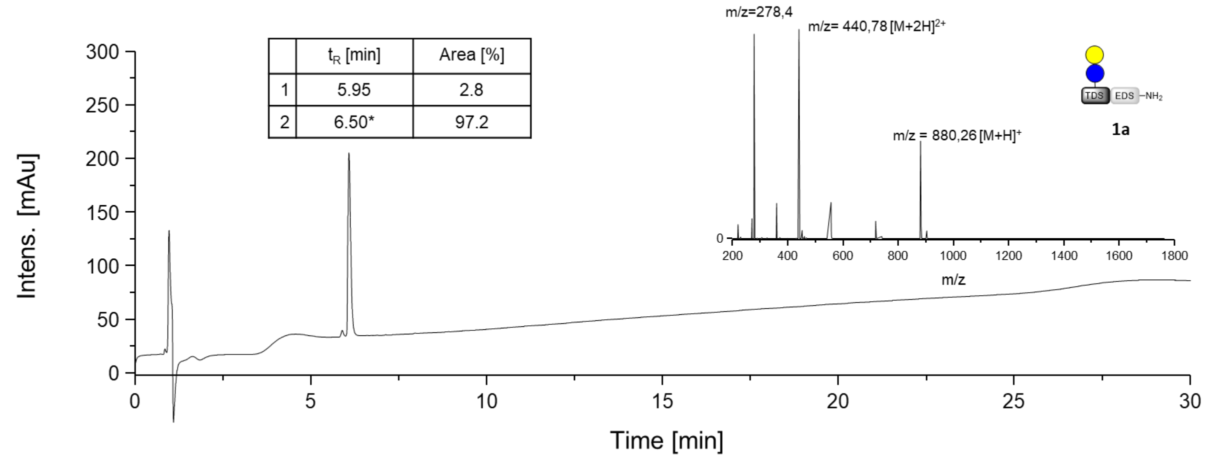

	t_R [min]	Area [%]
1	5.95	2.8
2	6.50*	97.2

Figure S 5: RP-HPLC chromatogram and ESI$^+$-MS spectrum of compound **1a.** Retention time t_R [min] and area [%] of the peaks are given. ESI-MS spectrum of the main peak (*) is shown.

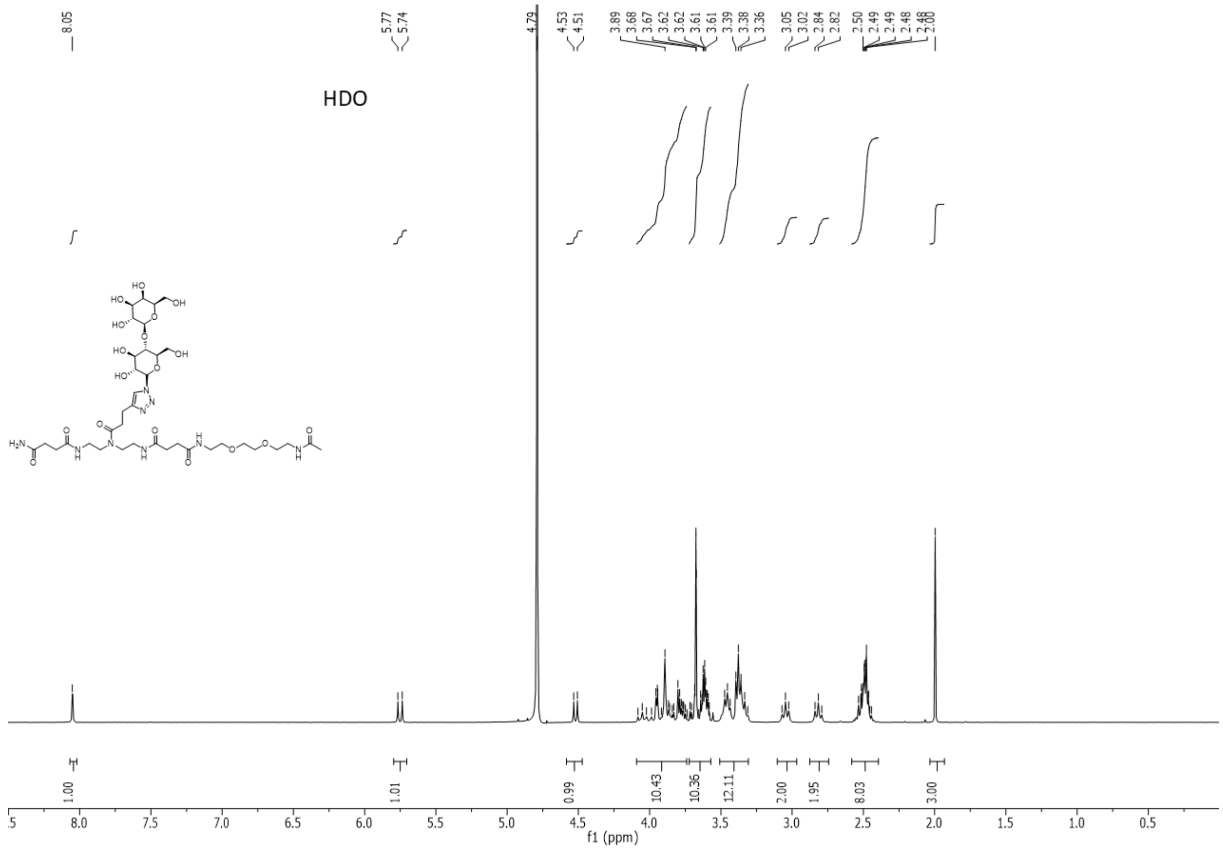

Figure S 6: ^{1}H-NMR spectrum of compound **1b.**

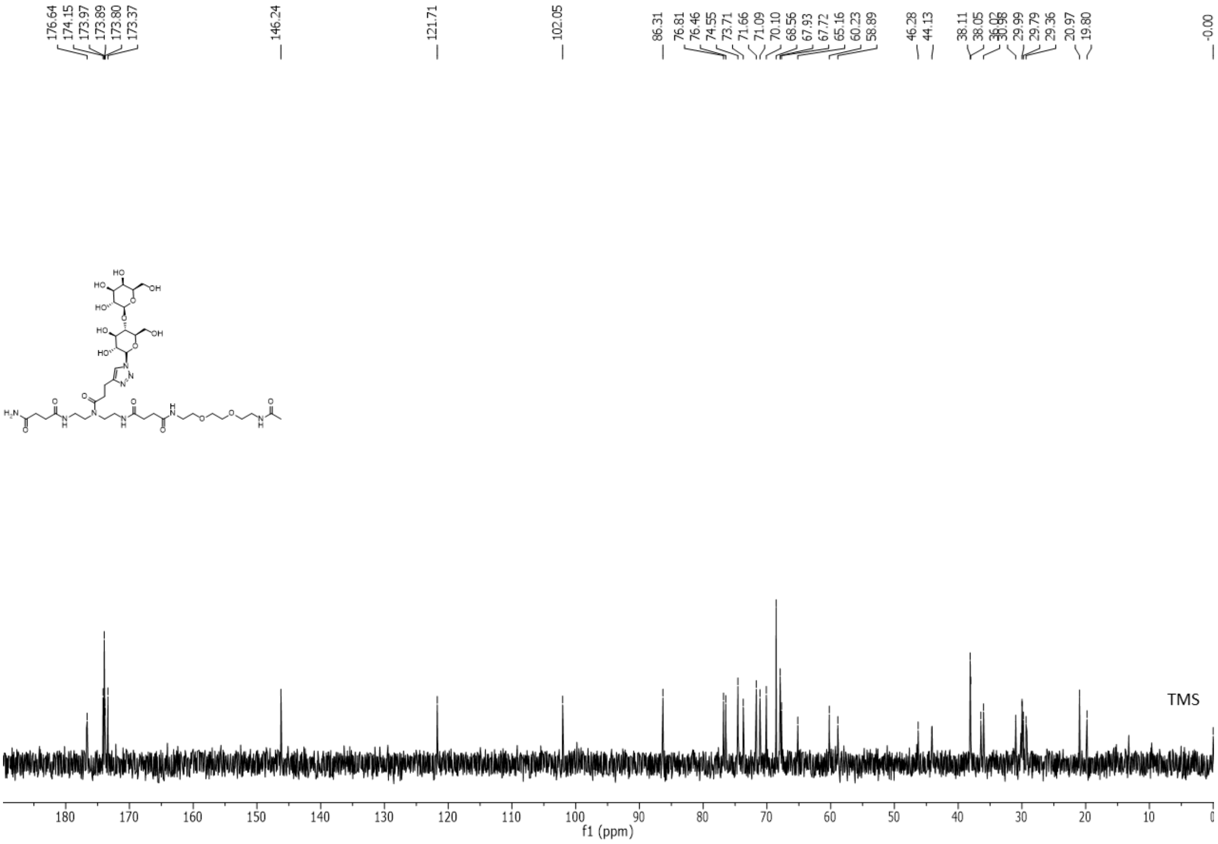

Figure S 7: ^{13}C-NMR spectrum of compound **1b.**

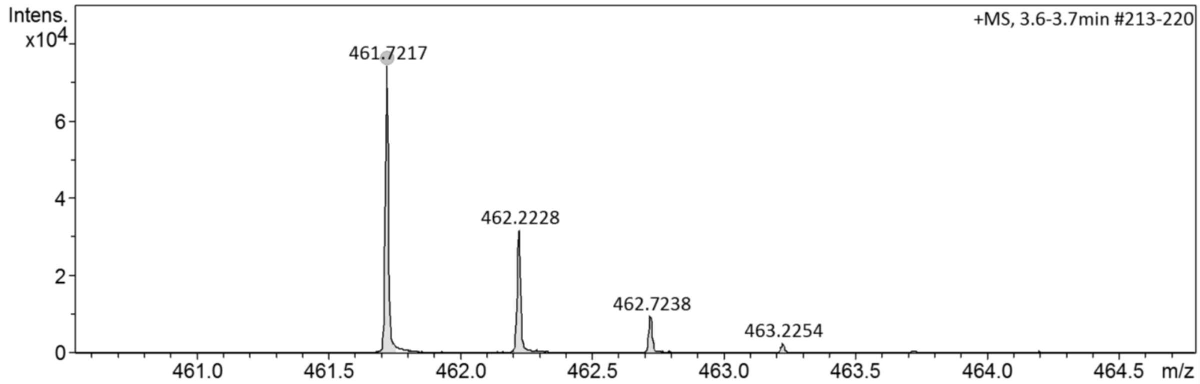

Figure S 8: HR-MS spectrum of compound **1b.**

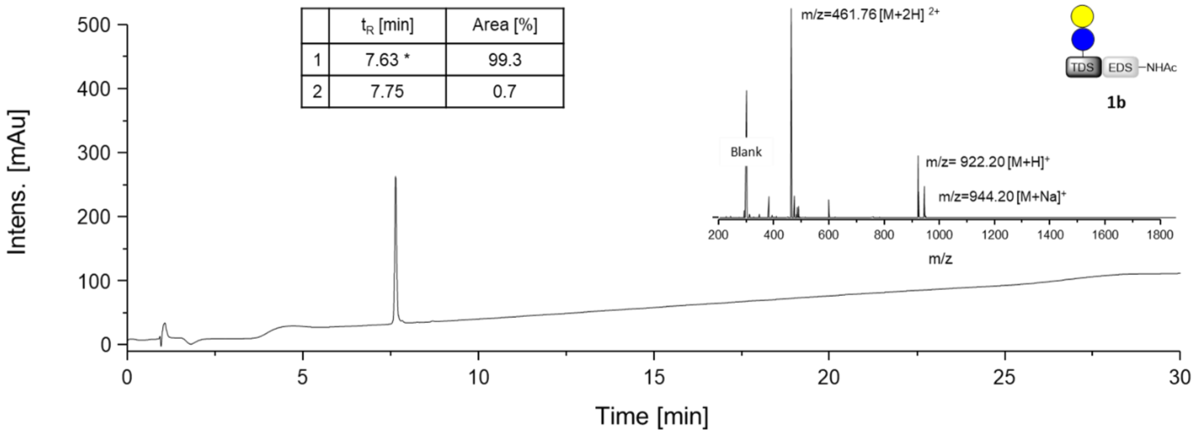

Figure S 9: RP-HPLC chromatogram and ESI$^+$-MS spectrum of compound **1b.** Retention time t_R [min] and area [%] of the peaks are given. ESI-MS spectrum of the main peak (*) is shown.

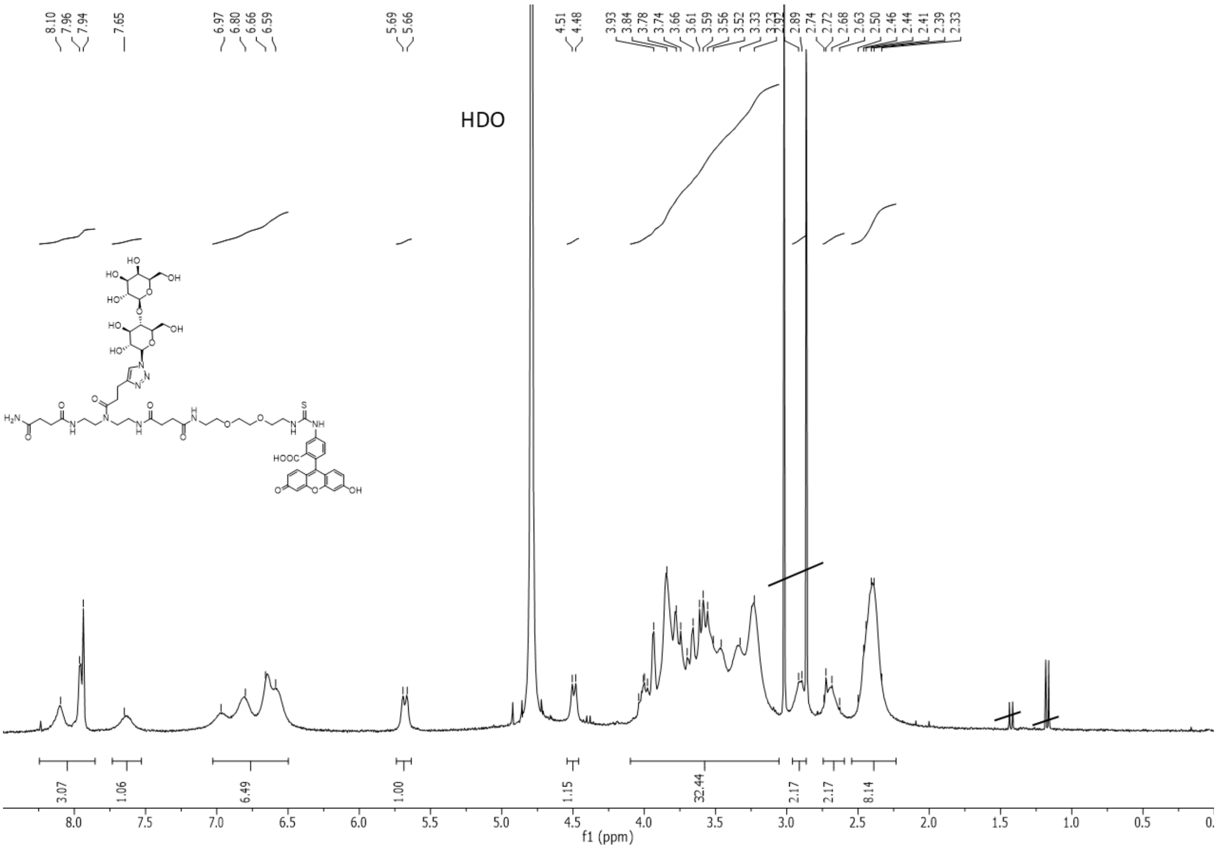

Figure S 10: ^{1}H-NMR spectrum of compound **1c**.

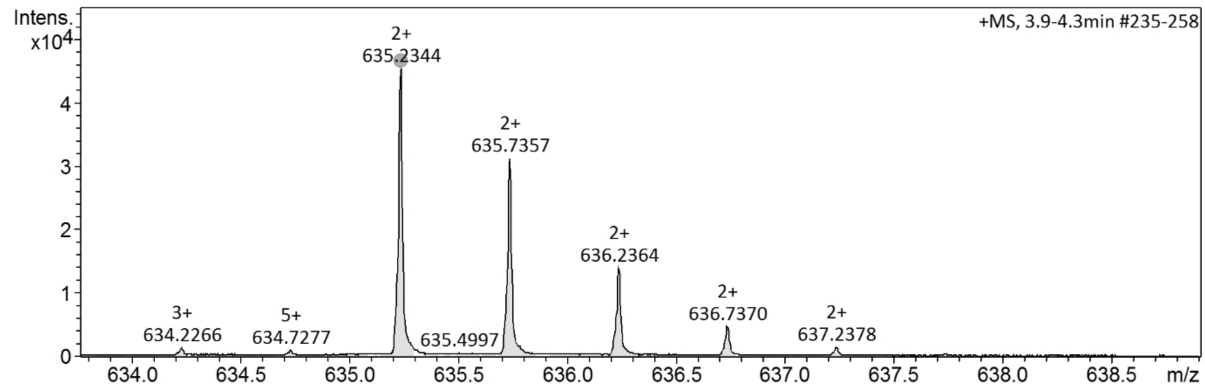

Figure S 11: HR-MS spectrum of compound **1c.**

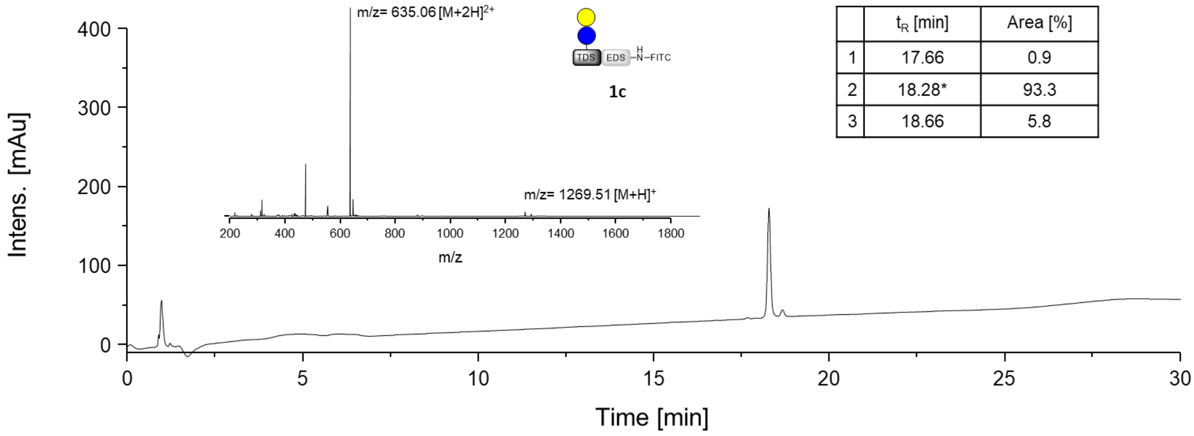

	t_R [min]	Area [%]
1	17.66	0.9
2	18.28*	93.3
3	18.66	5.8

Figure S 12: RP-HPLC chromatogram and ESI^{+}-MS spectrum of compound **1c.** Retention time t_R [min] and area [%] of the peaks are given. ESI-MS spectrum of the main peak (*) is shown.

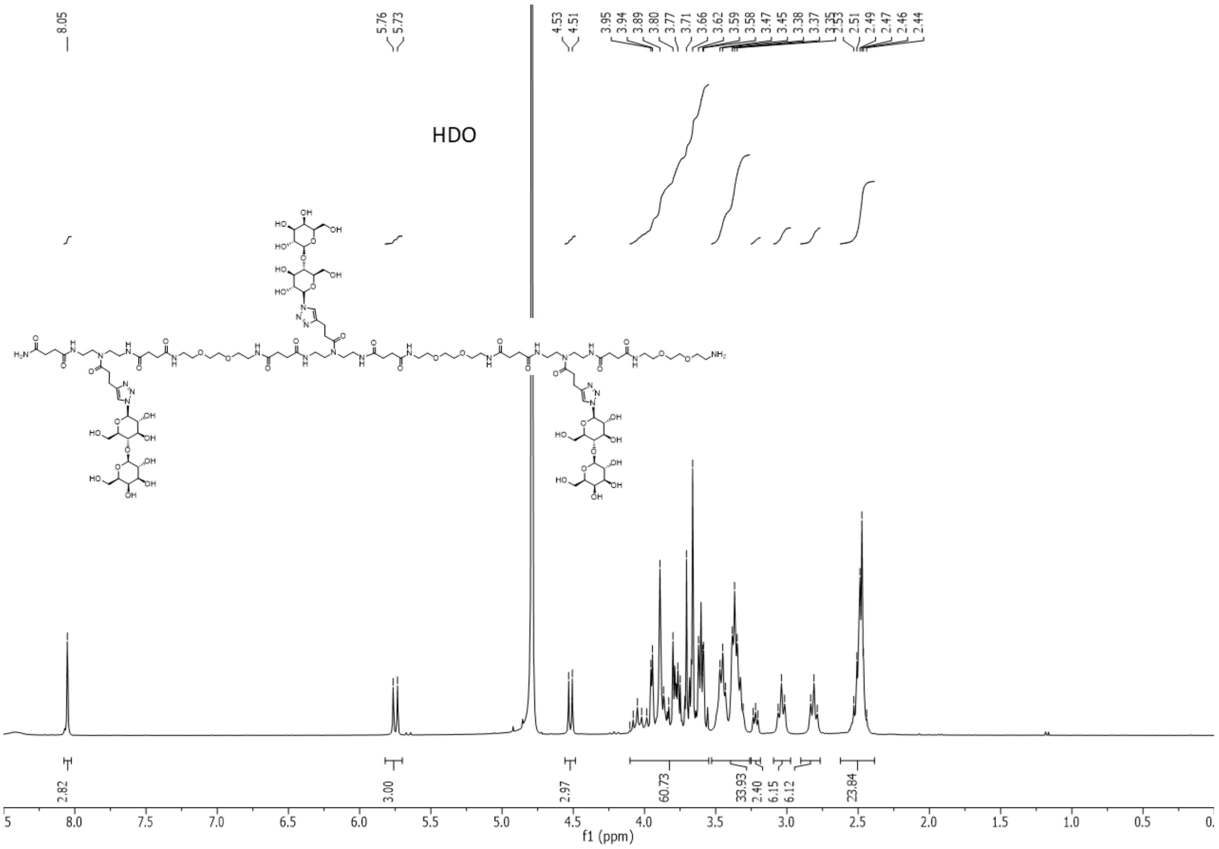

Figure S 13: ^{1}H-NMR spectrum of compound **2a**.

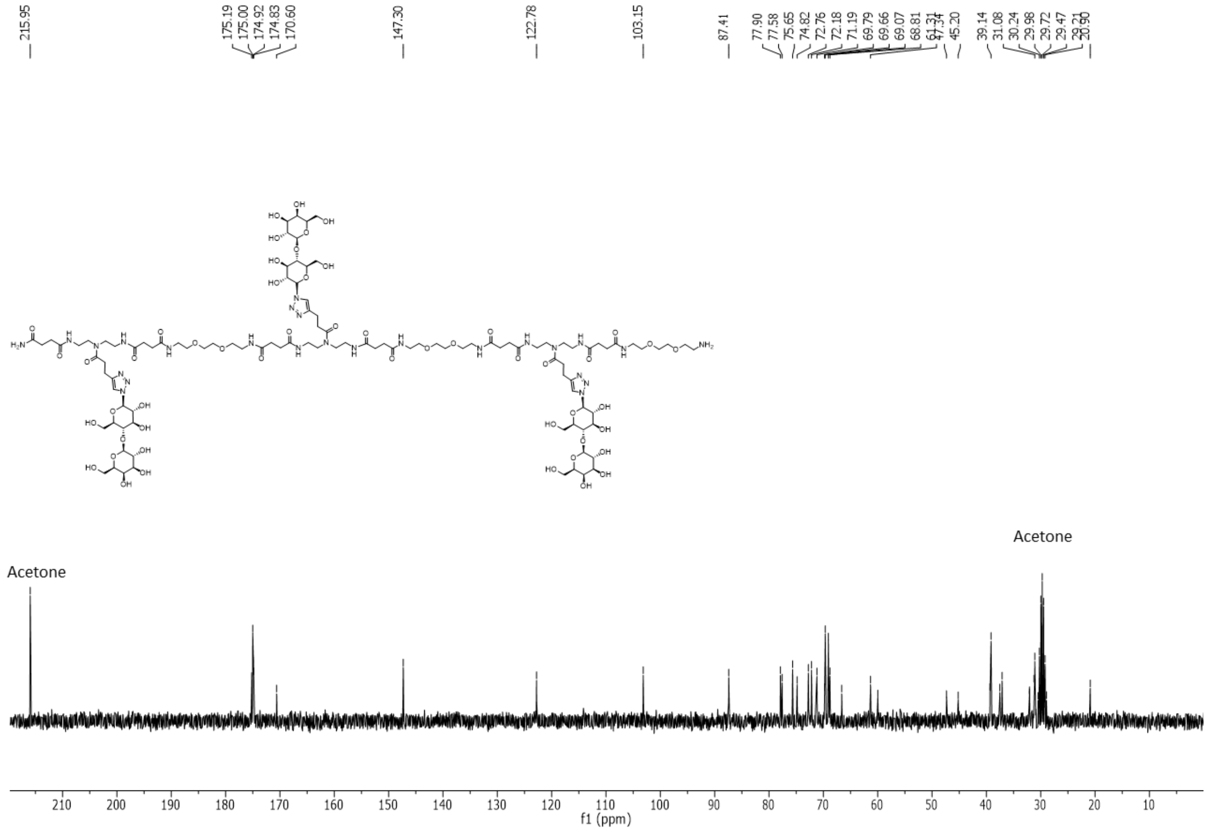

Figure S 14: ^{13}C-NMR spectrum of compound **2a**.

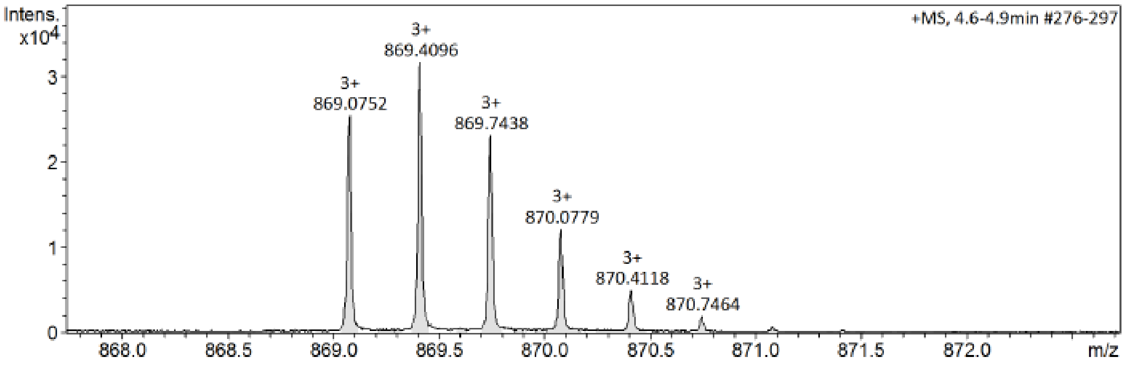

Figure S 15: HR-MS spectrum of compound **2a.**

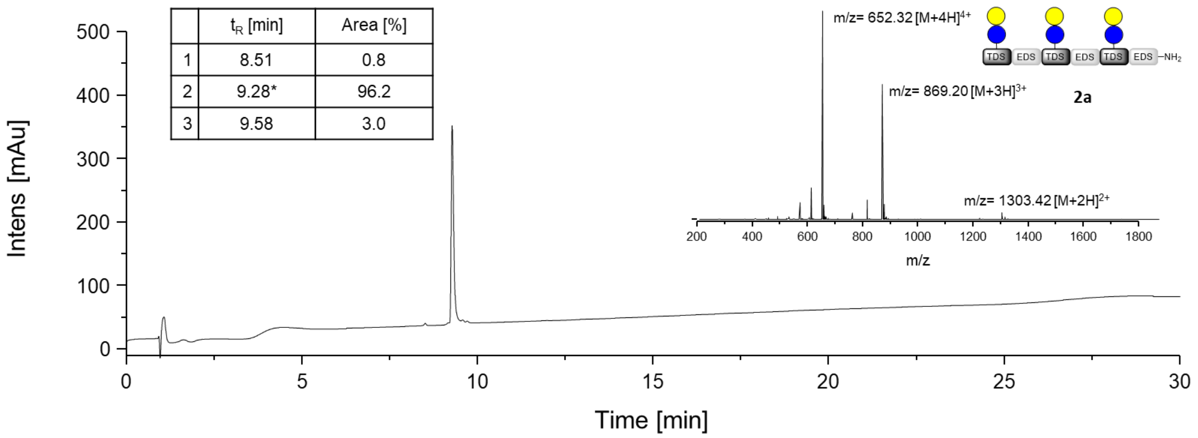

Figure S 16: RP-HPLC chromatogram and ESI^{+}-MS spectrum of compound **2a.** Retention time t_R [min] and area [%] of the peaks are given. ESI-MS spectrum of the main peak (*) is shown.

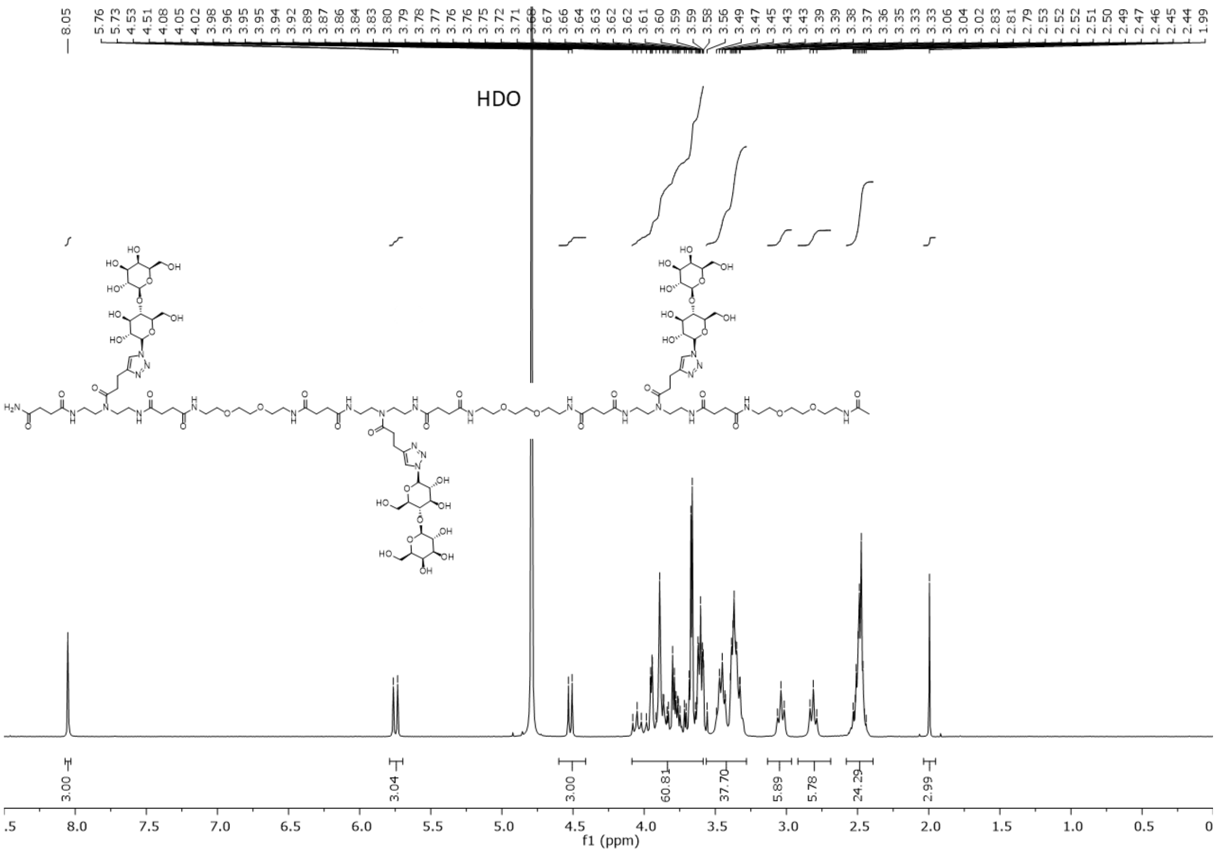

Figure S 17: ^{1}H-NMR spectrum of compound **2b.**

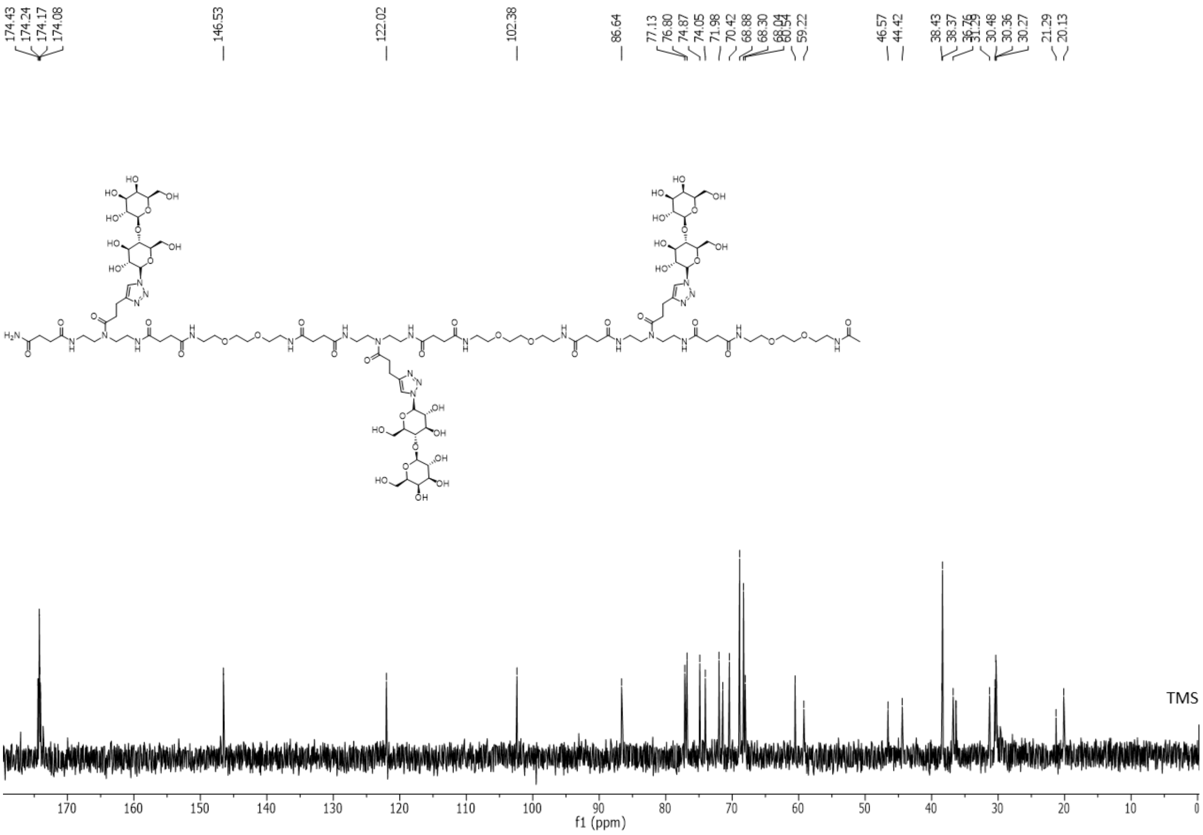

Figure S 18: ^{13}C-NMR spectrum of compound **2b**.

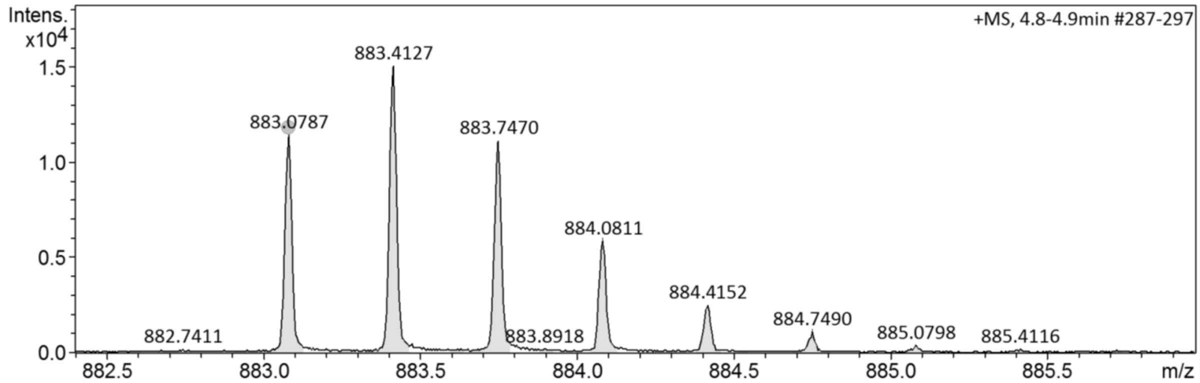

Figure S 19: HR-MS spectrum of compound **2b.**

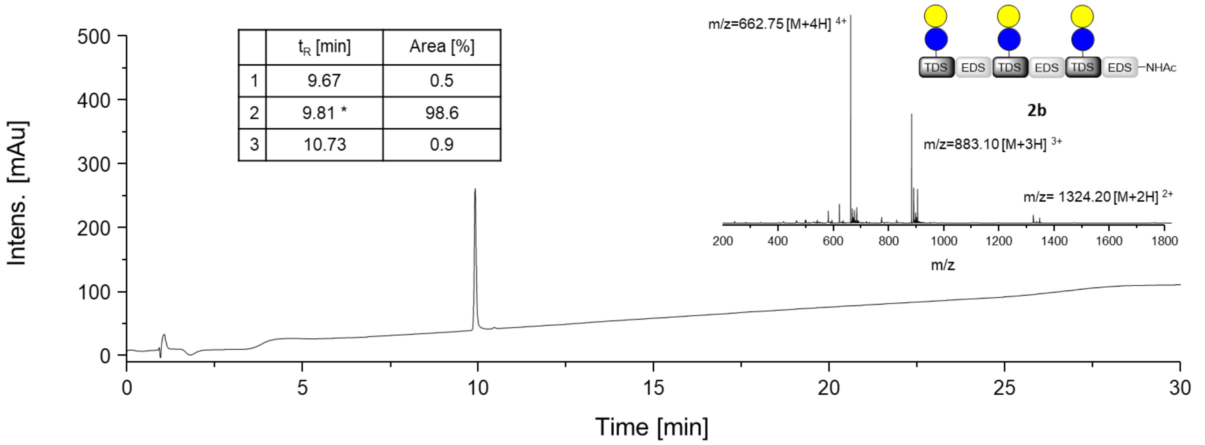

	t_R [min]	Area [%]
1	9.67	0.5
2	9.81 *	98.6
3	10.73	0.9

Figure S 20: RP-HPLC chromatogram and ESI$^+$-MS spectrum of compound **2b.** Retention time t_R [min] and area [%] of the peaks are given. ESI-MS spectrum of the main peak (*) is shown.

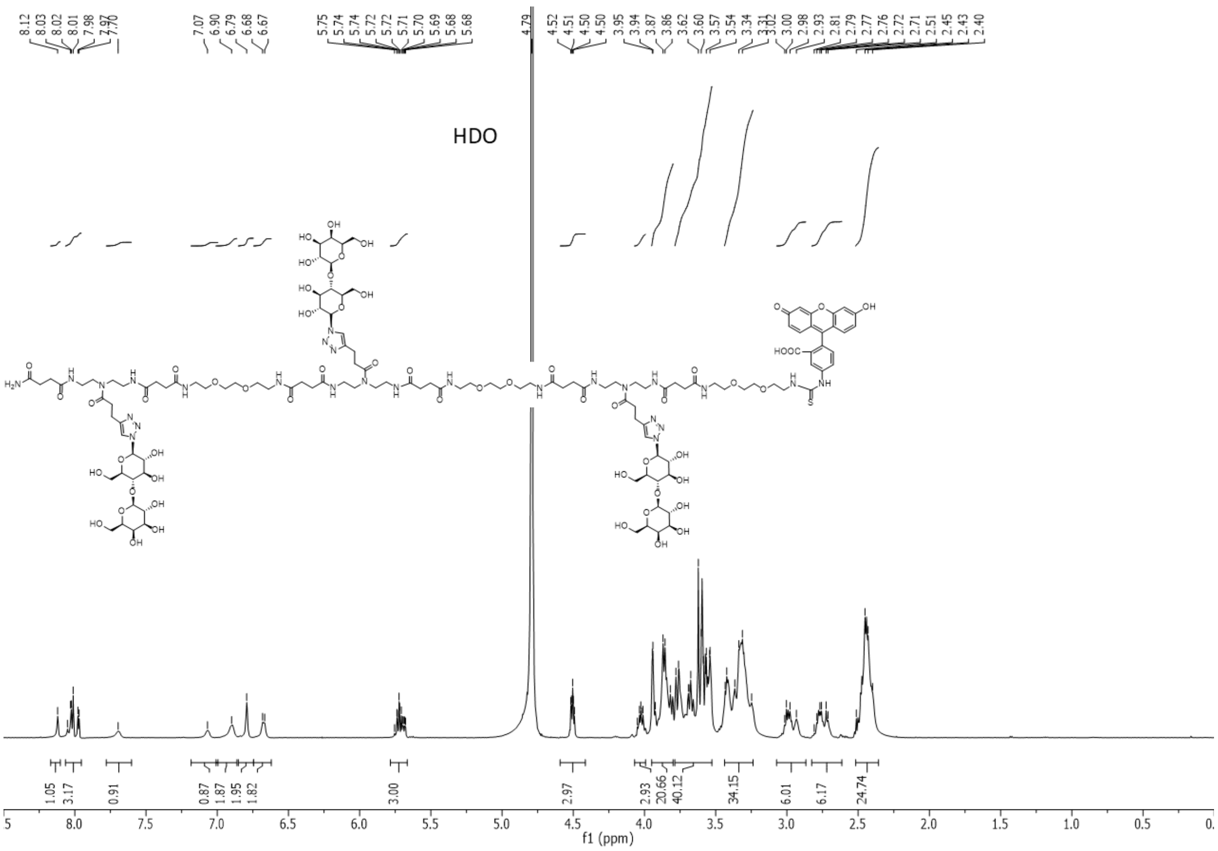

Figure S 21: ^{1}H-NMR spectrum of compound **2c.**

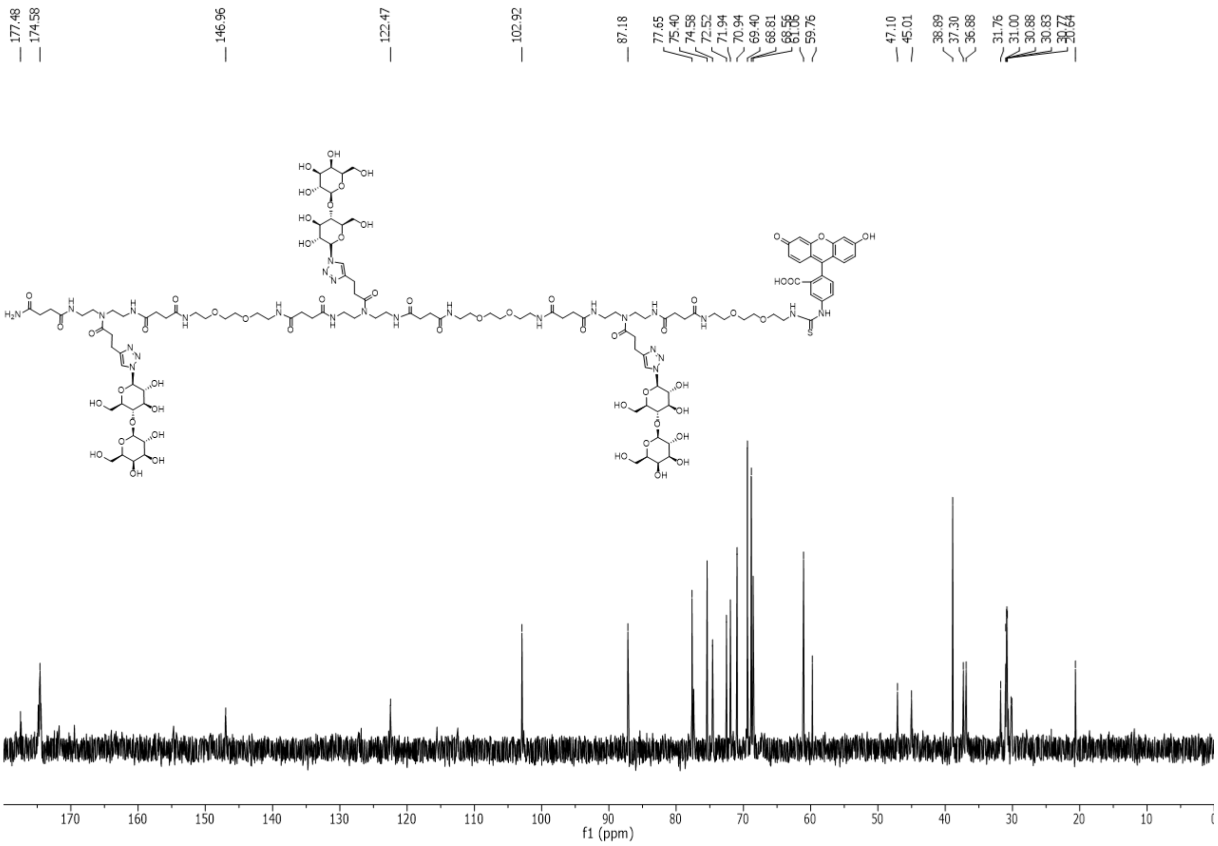

Figure S 22: ^{13}C-NMR spectrum of compound **2c**.

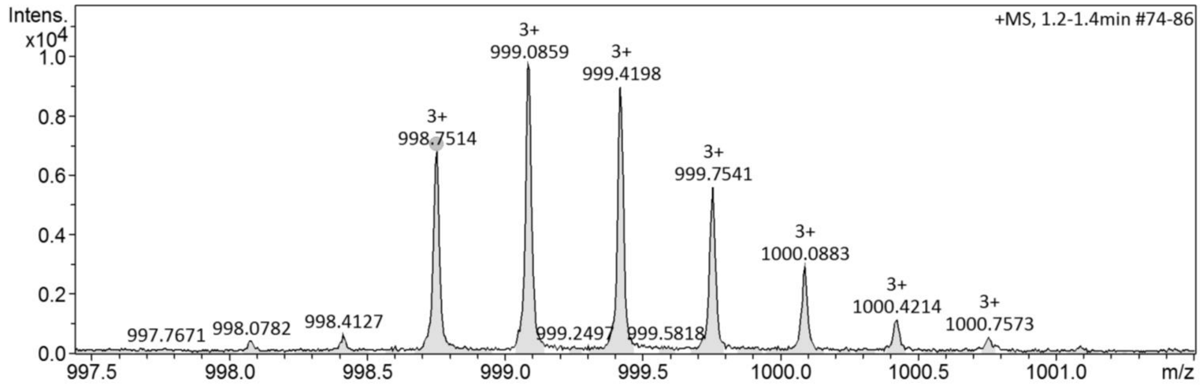

Figure S 23: HR-MS spectrum of compound **2c.**

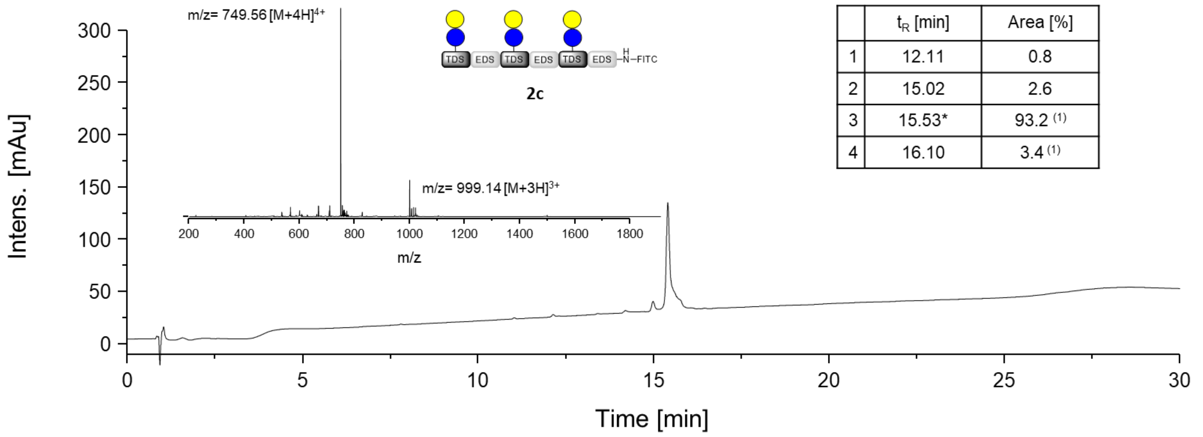

	t_R [min]	Area [%]
1	12.11	0.8
2	15.02	2.6
3	15.53*	93.2 (1)
4	16.10	3.4 (1)

Figure S 24: RP-HPLC chromatogram and ESI$^+$-MS spectrum of compound **2c**. Retention time tR [min] and area [%] of the peaks are given. ESI-MS spectrum of the main peak (*) is shown. (1) Peaks with the same m/z.

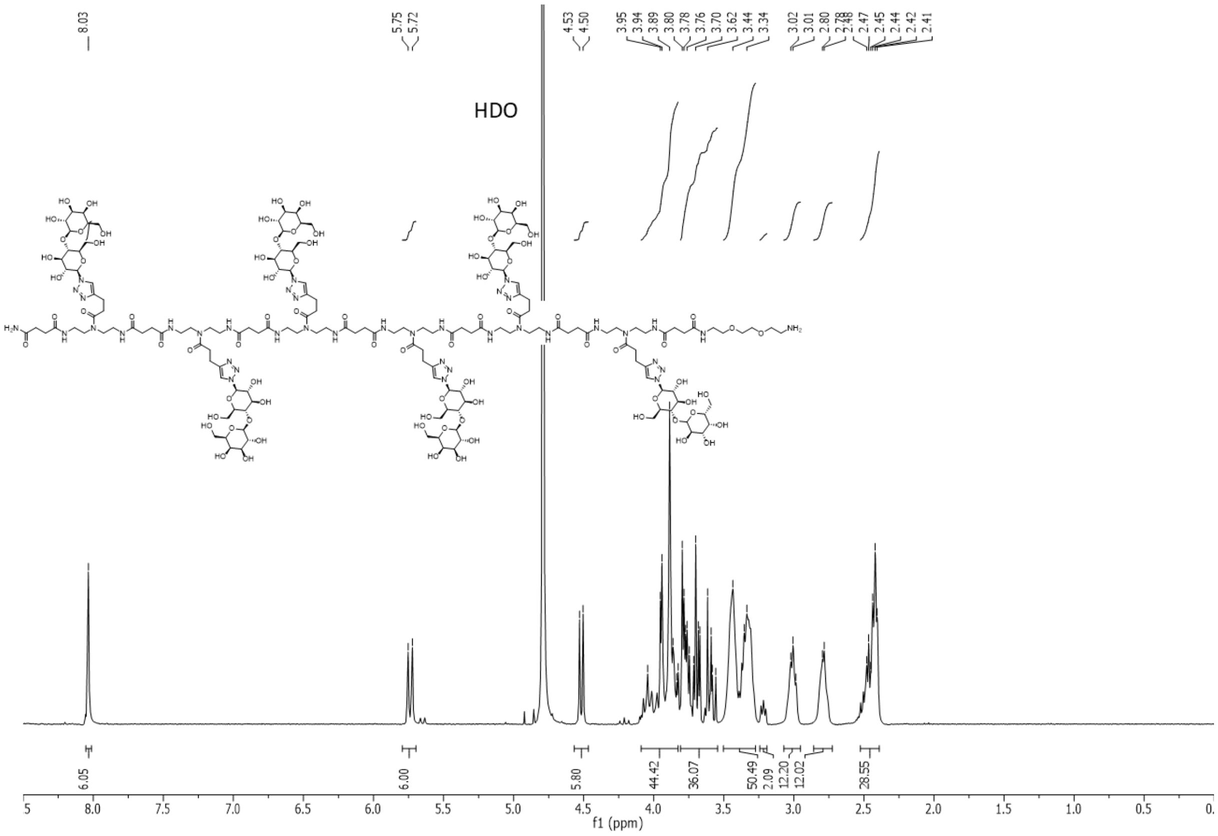

Figure S 25: ^{1}H-NMR spectrum of compound **3a**.

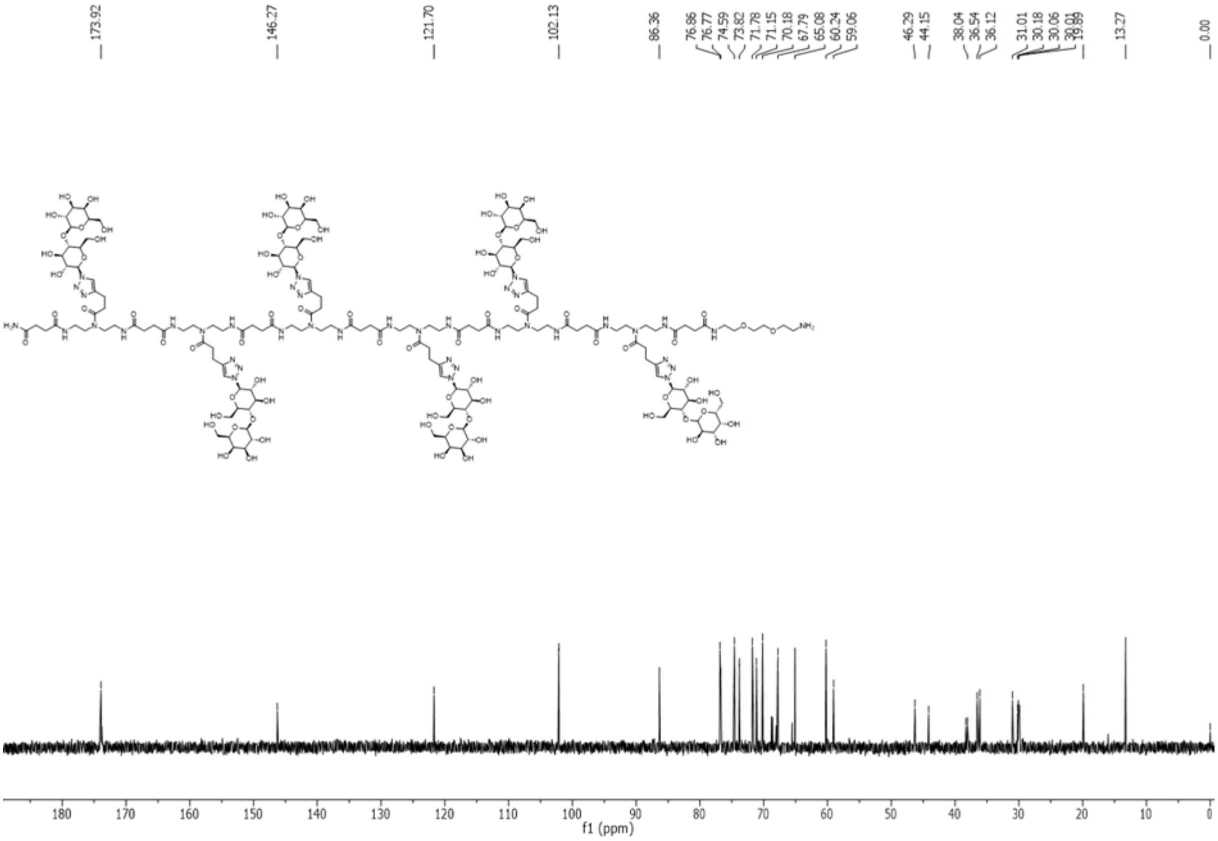

Figure S 26: ^{13}C-NMR spectrum of compound **3a**.

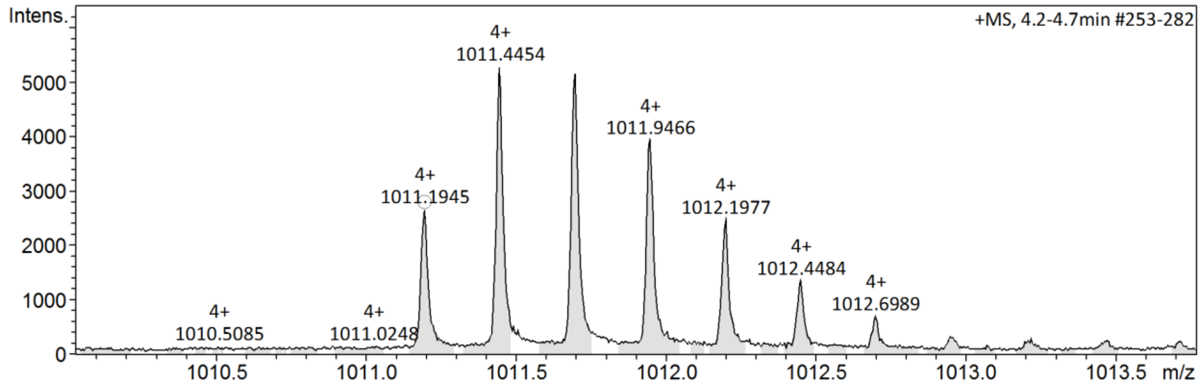

Figure S 27: RP-HPLC chromatogram and ESI^{+}-MS spectrum of compound **3a.**

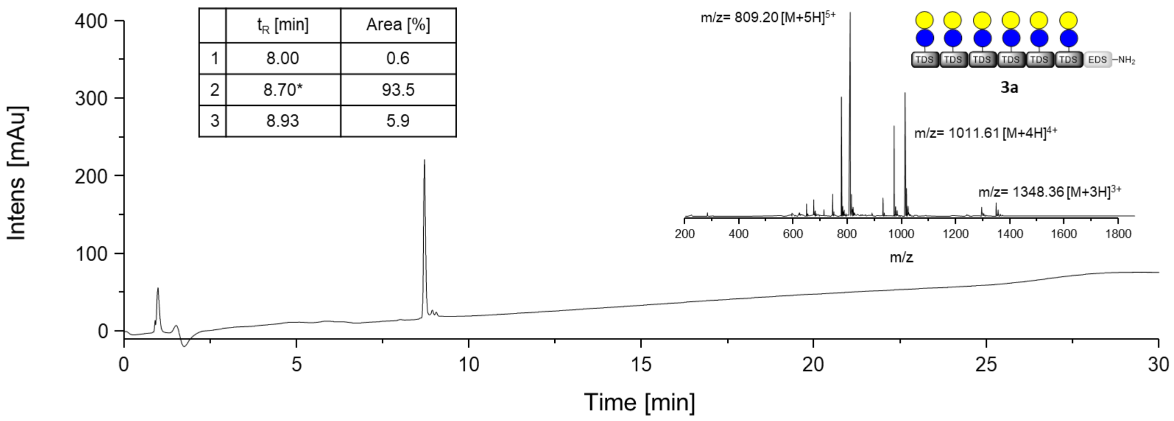

Figure S 28: RP-HPLC chromatogram and ESI$^+$-MS spectrum of compound **3a.** Retention time t_R [min] and area [%] of the peaks are given. ESI-MS spectrum of the main peak (*) is shown.

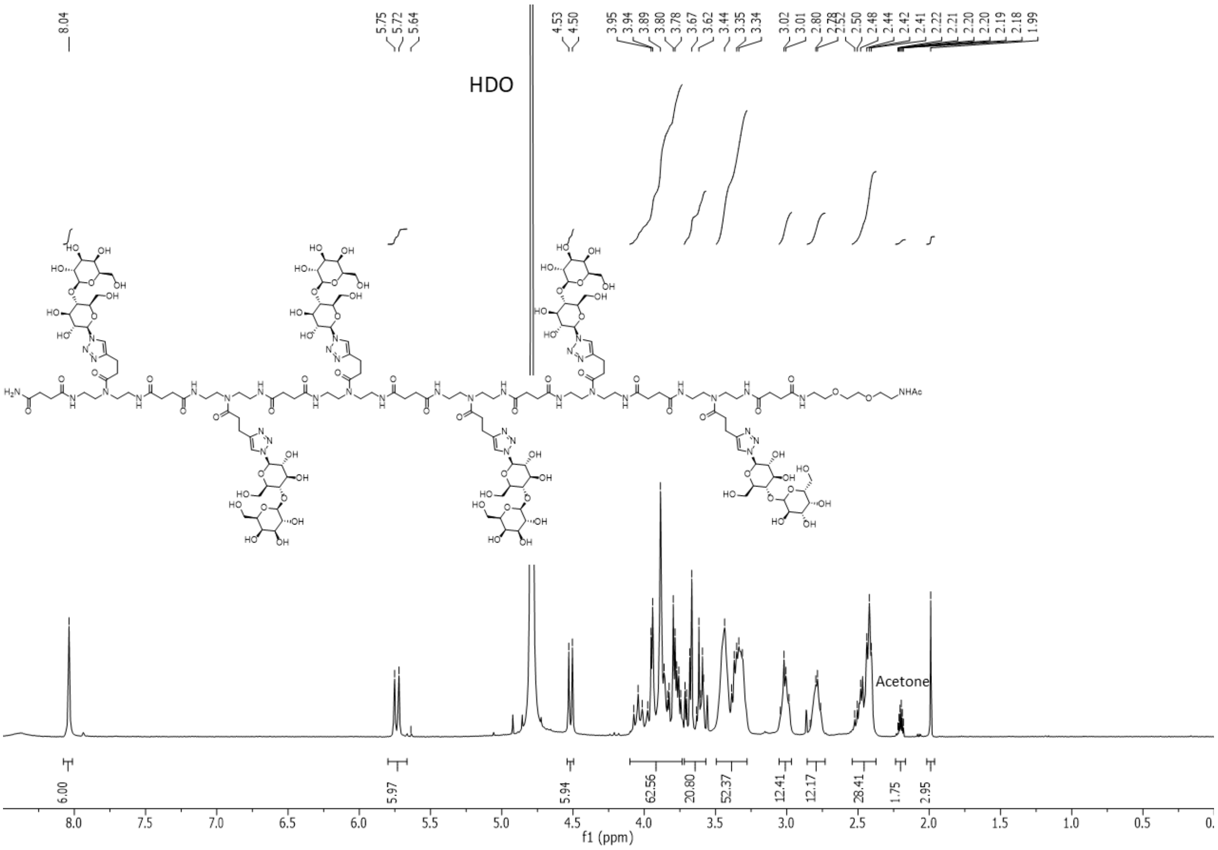

Figure S 29: ^{1}H-NMR spectrum of compound **3b**.

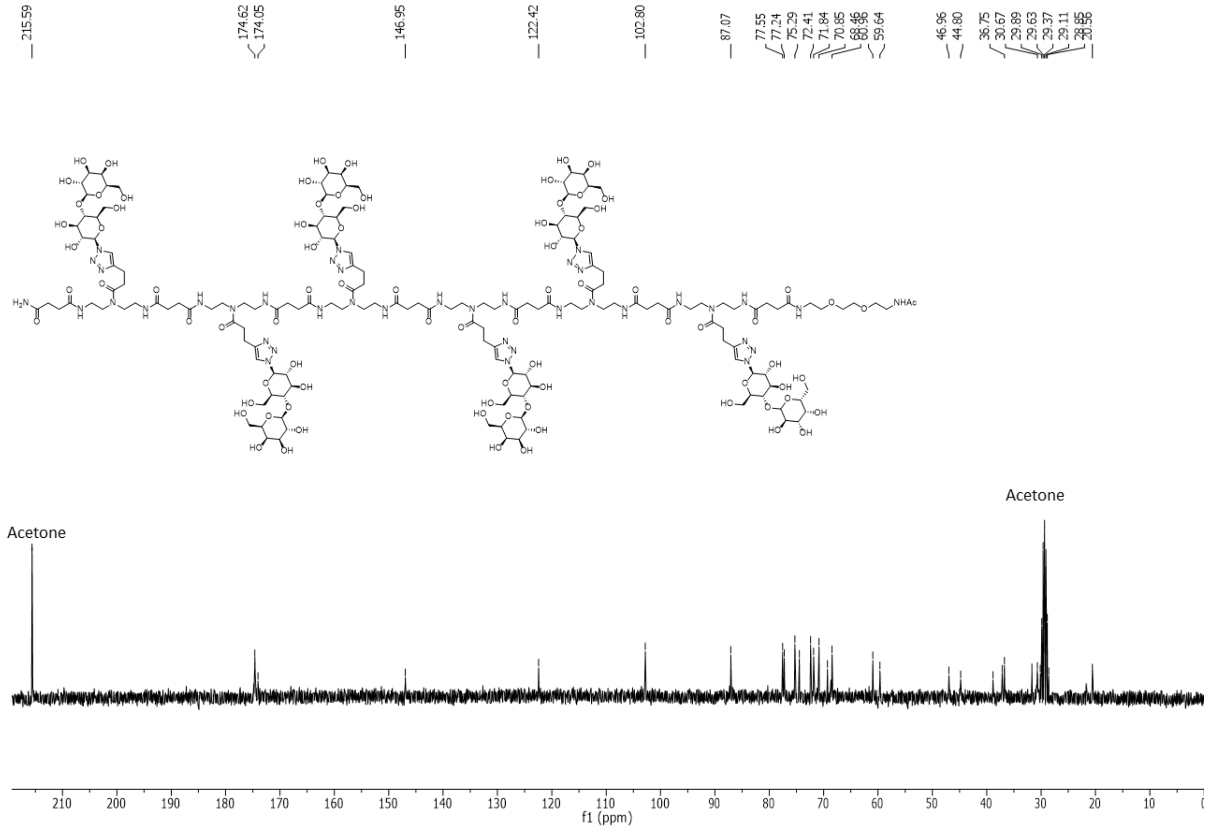

Figure S 30: ^{13}C-NMR spectrum of compound **3b**.

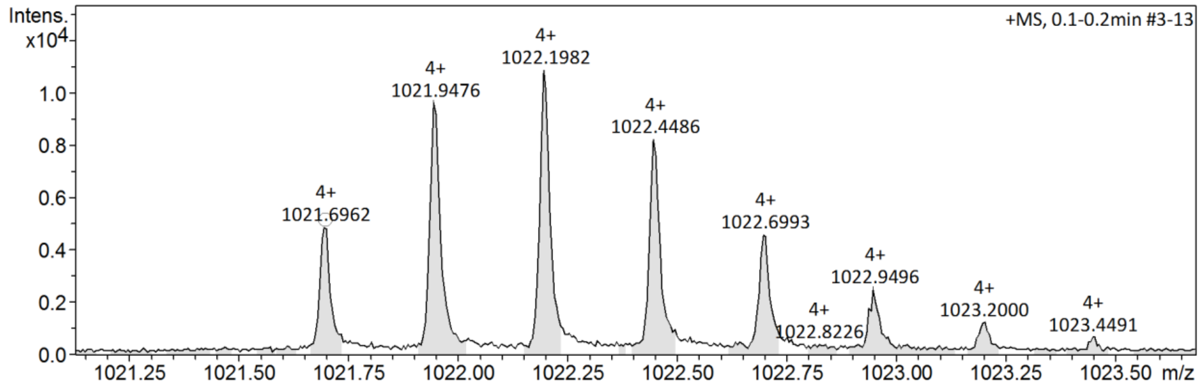

Figure S 31: HR-MS spectrum of compound **3b.**

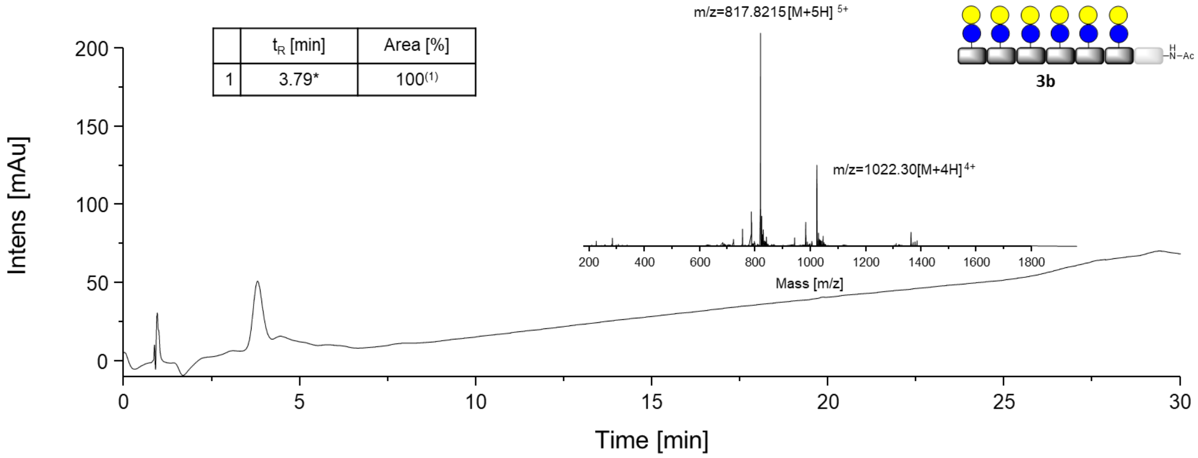

	t_R [min]	Area [%]
1	3.79*	100(1)

Figure S 32: RP-HPLC chromatogram and ESI$^+$-MS spectrum of compound **3b.** Retention time t_R [min] and area [%] of the peaks are given. ESI-MS spectrum of the main peak (*) is shown.

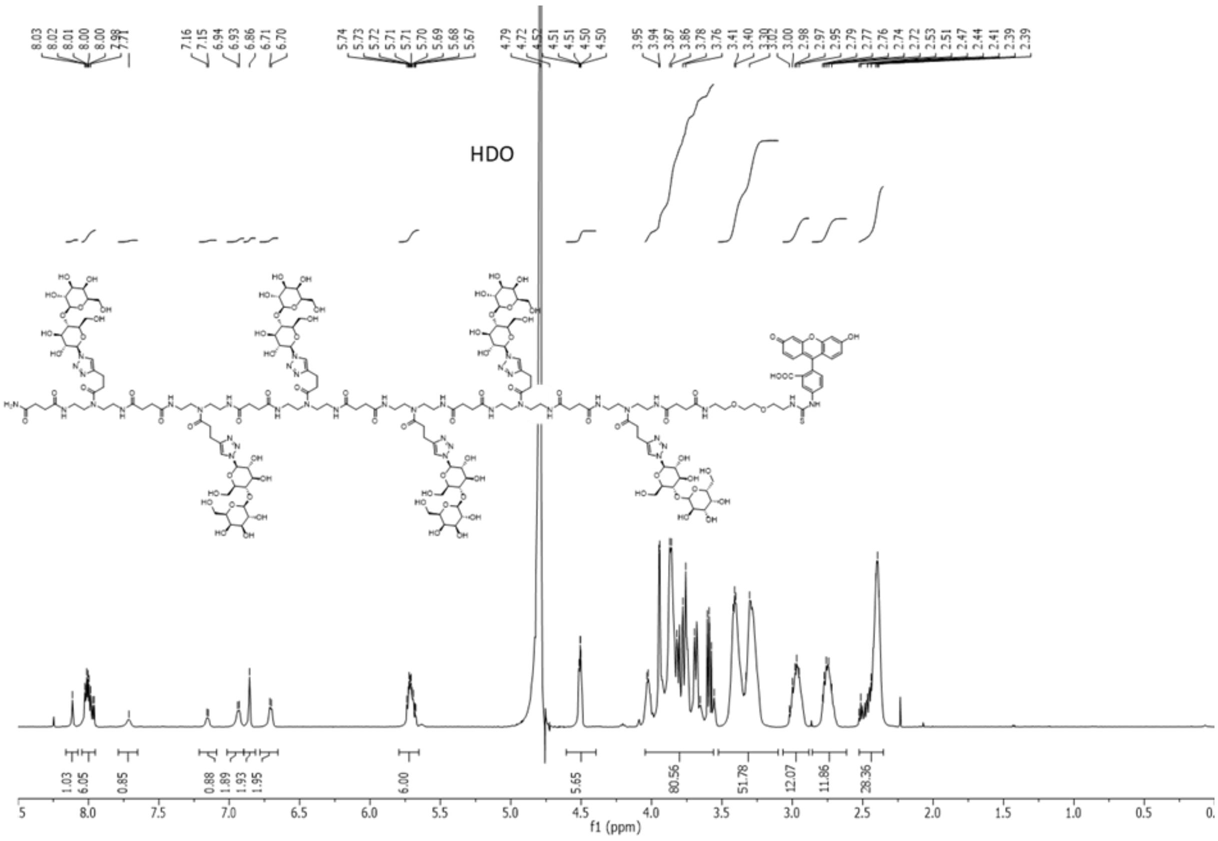

Figure S 33: ^{1}H-NMR spectrum of compound **3c**.

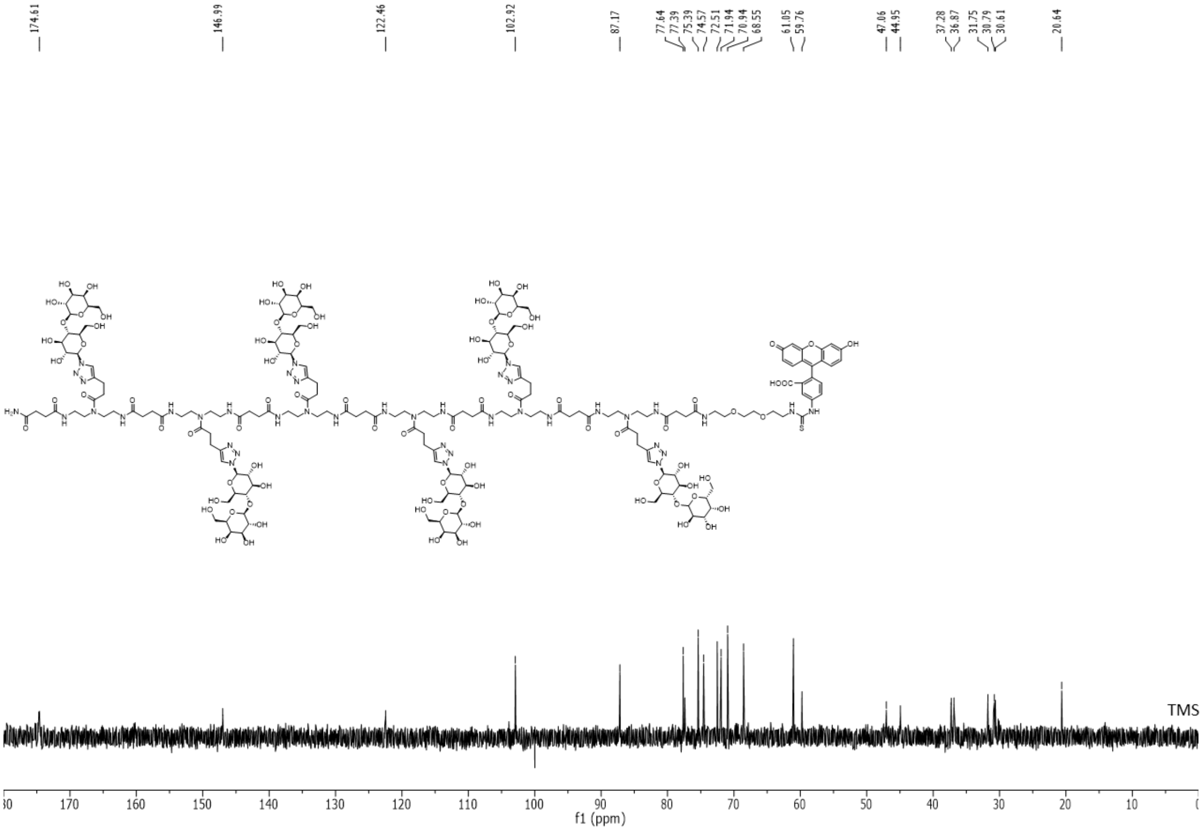

Figure S 34: ^{13}C-NMR spectrum of compound **3c**.

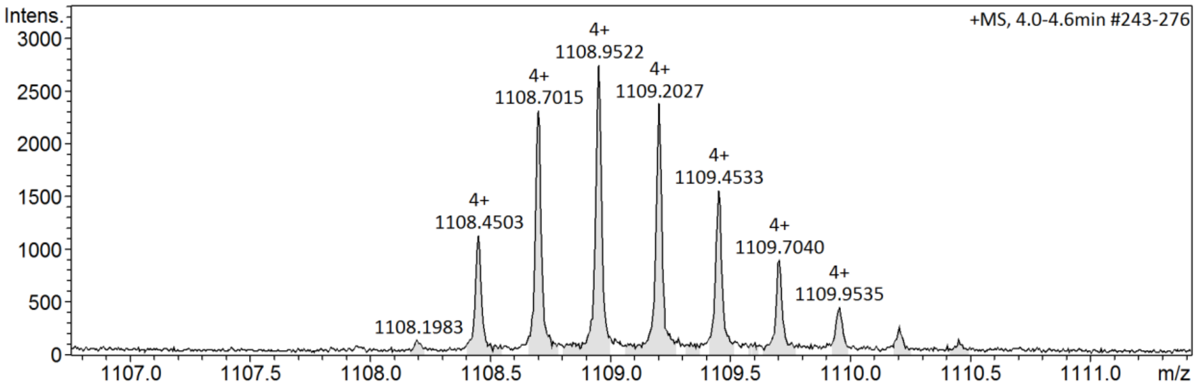

Figure S 35**:** HR-MS spectrum of compound **3c.**

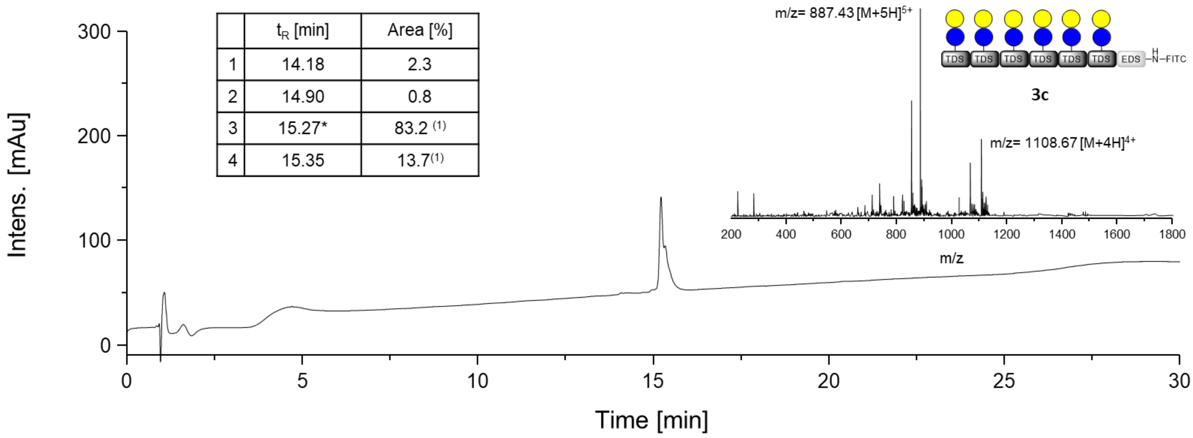

Figure S 36: RP-HPLC chromatogram and ESI$^+$-MS spectrum of compound **3c.** Retention time t_R [min] and area [%] of the peaks are given. ESI-MS spectrum of the main peak (*) is shown. (1) Peaks show the same m/z.

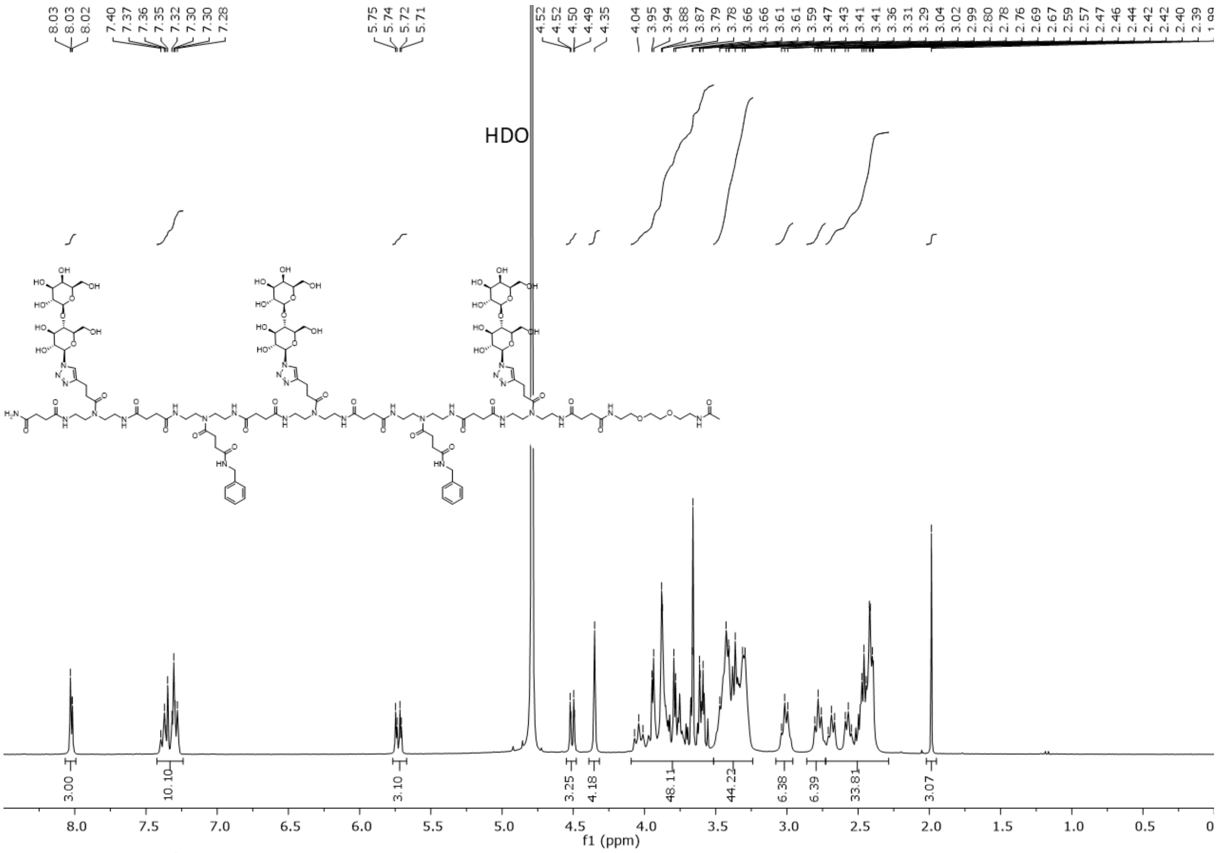

Figure S 37: ^{1}H-NMR spectrum of compound **4b**.

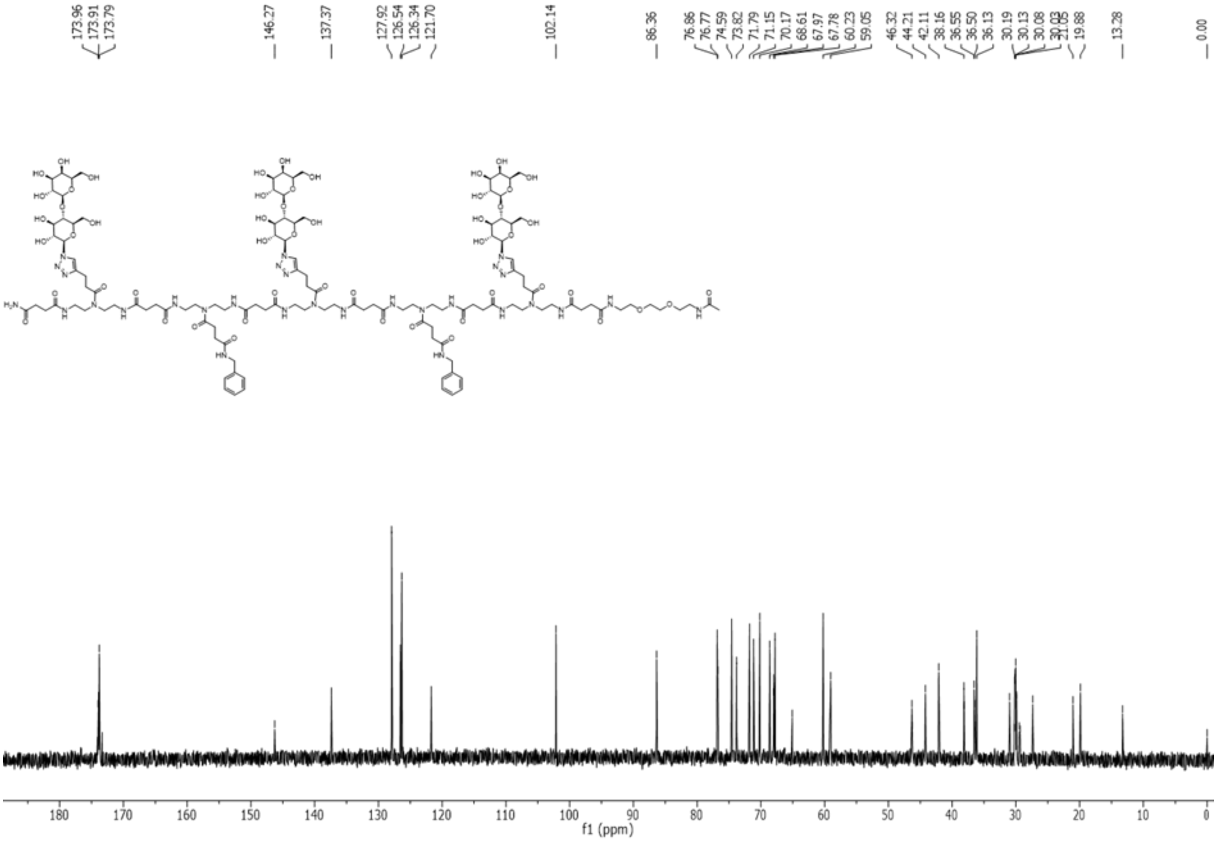

Figure S 38: ^{13}C-NMR spectrum of compound **4b**.

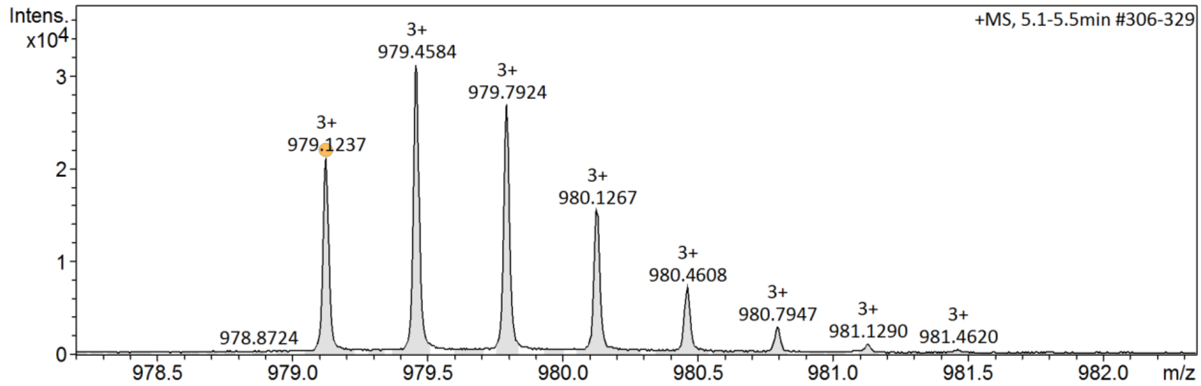

Figure S 39: HR-MS spectrum of compound **4b.**

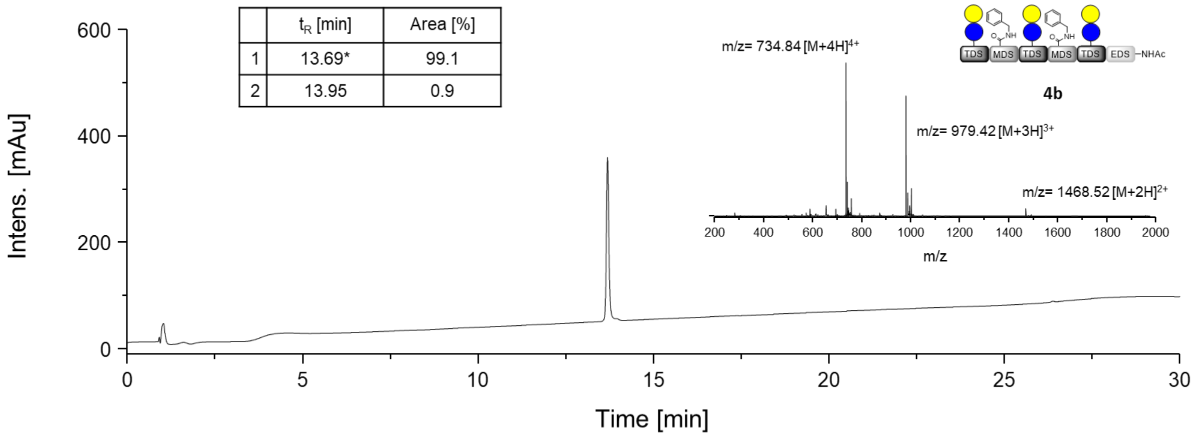

	t_R [min]	Area [%]
1	13.69*	99.1
2	13.95	0.9

Figure S 40: RP-HPLC chromatogram and ESI$^+$-MS spectrum of compound **4b.** Retention time t_R [min] and area [%] of the peaks are given. ESI-MS spectrum of the main peak (*) is shown.

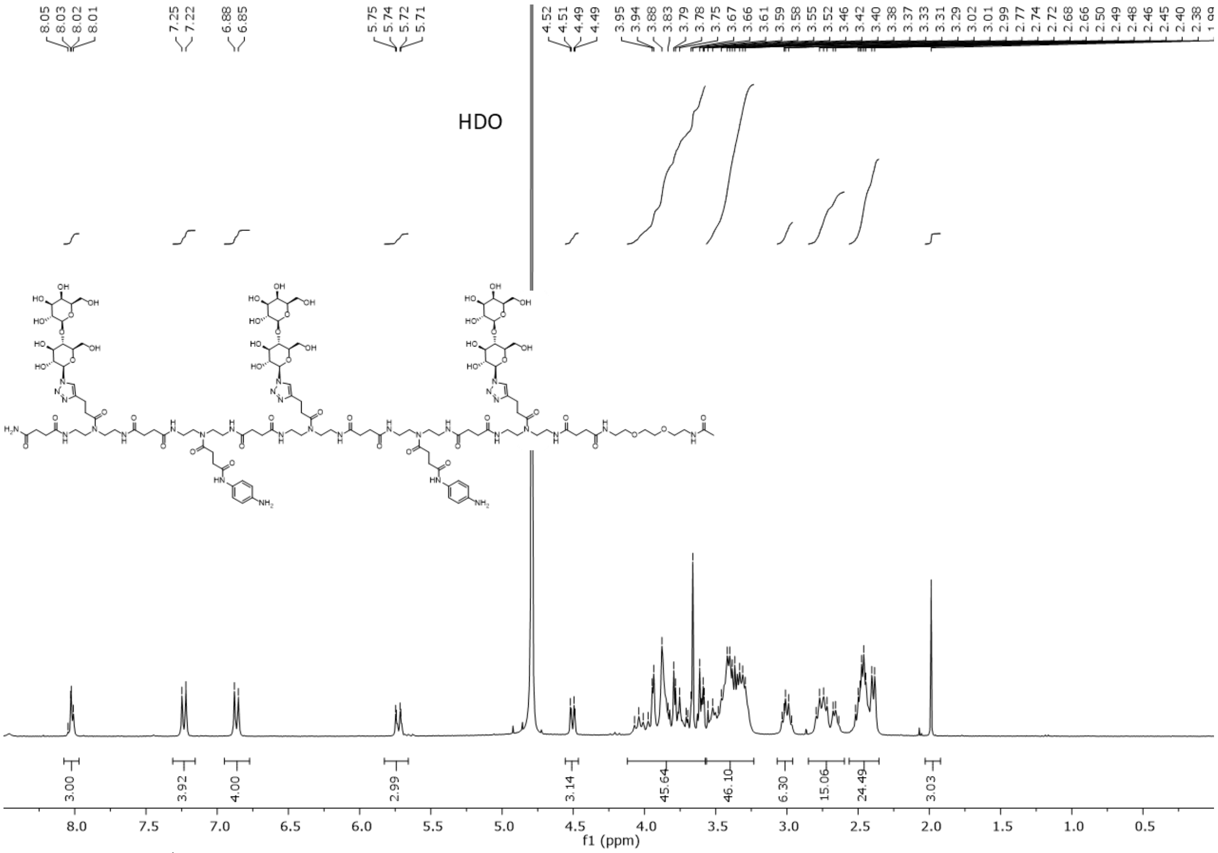

Figure S 41: ^{1}H-NMR spectrum of compound **5b**.

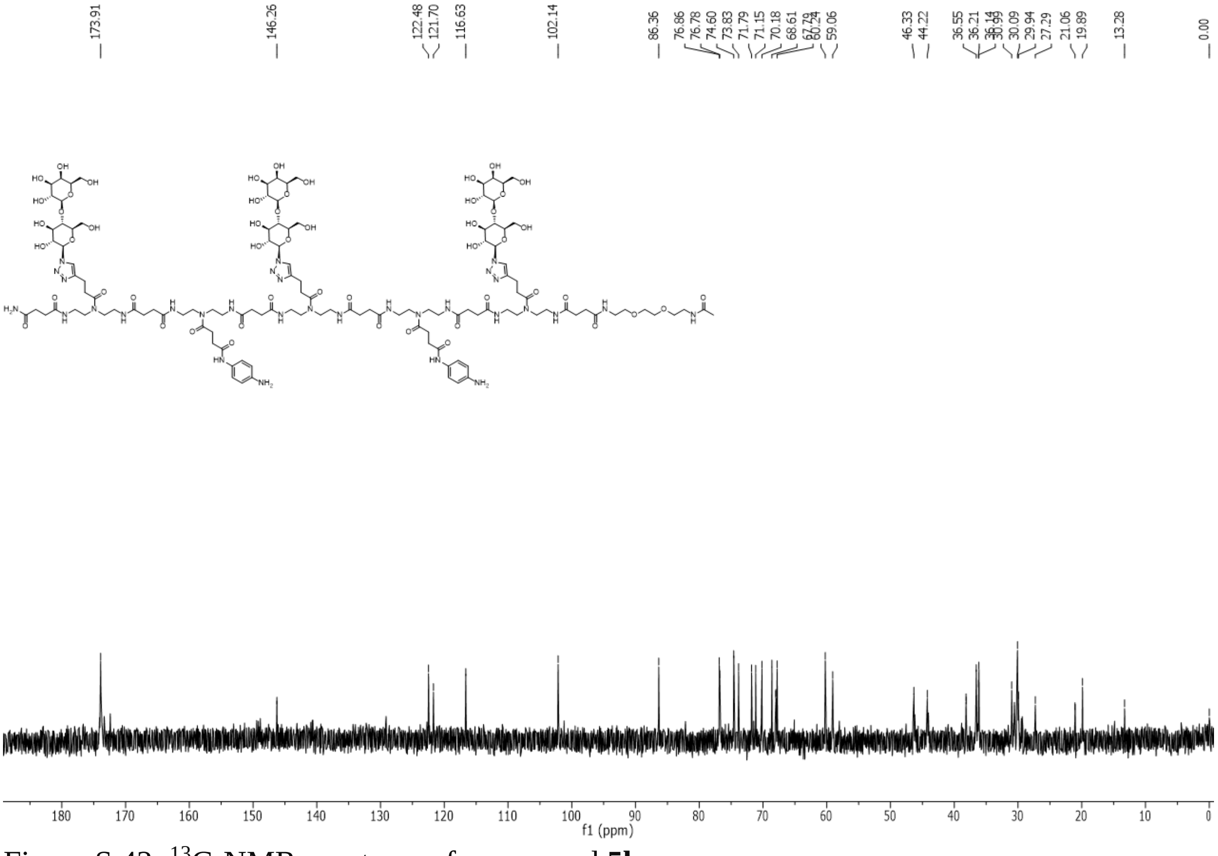

Figure S 42: ^{13}C-NMR spectrum of compound **5b**.

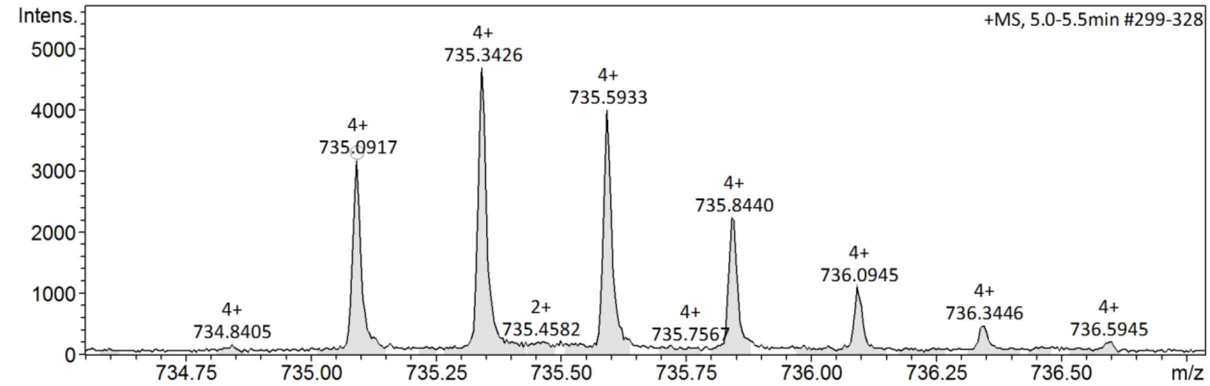

Figure S 43: HR-MS spectrum of compound **5b.**

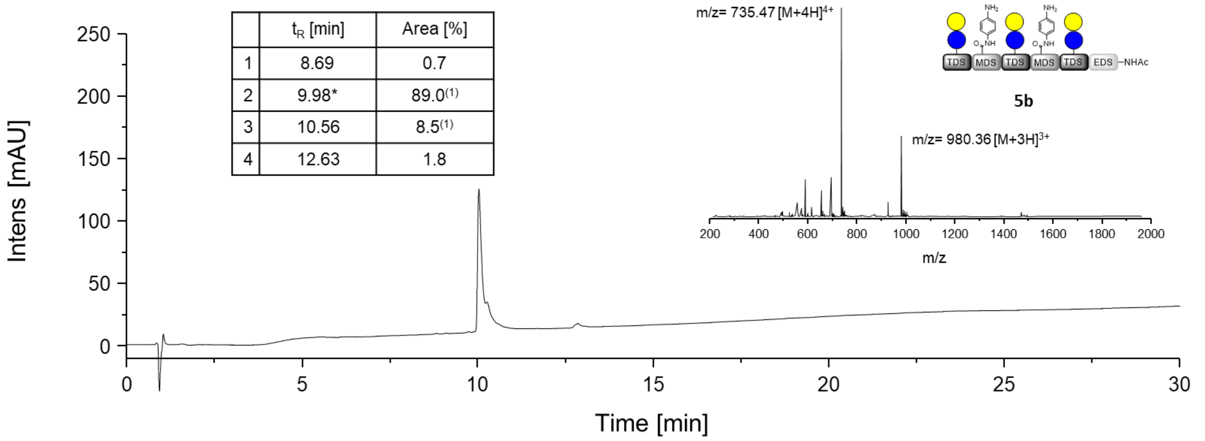

	t_R [min]	Area [%]
1	8.69	0.7
2	9.98*	89.0(1)
3	10.56	8.5(1)
4	12.63	1.8

Figure S 44: RP-HPLC chromatogram and ESI$^+$-MS spectrum of compound **5b.** Retention time t_R [min] and area [%] of the peaks are given. ESI-MS spectrum of the main peak (*) is shown. (1) Peaks with the same m/z.

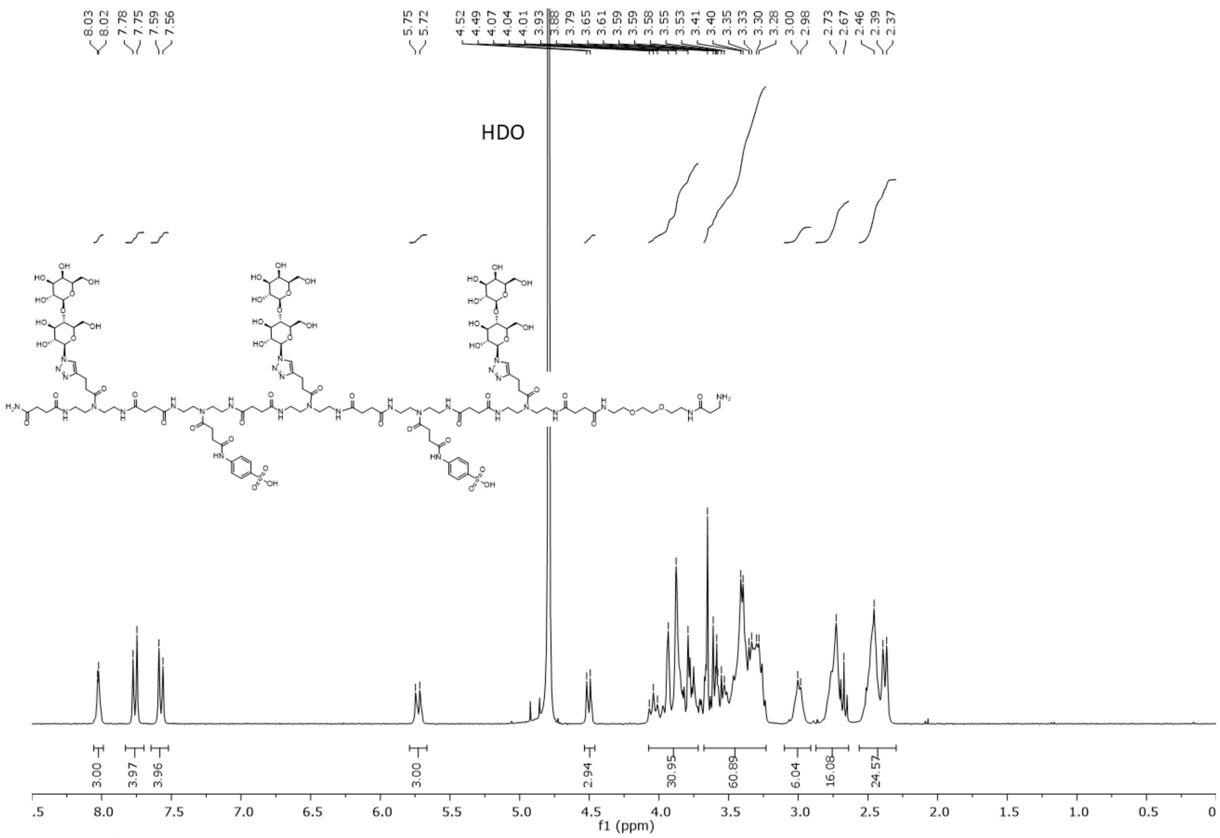

Figure S 45: ^{1}H-NMR spectrum of compound **6a**.

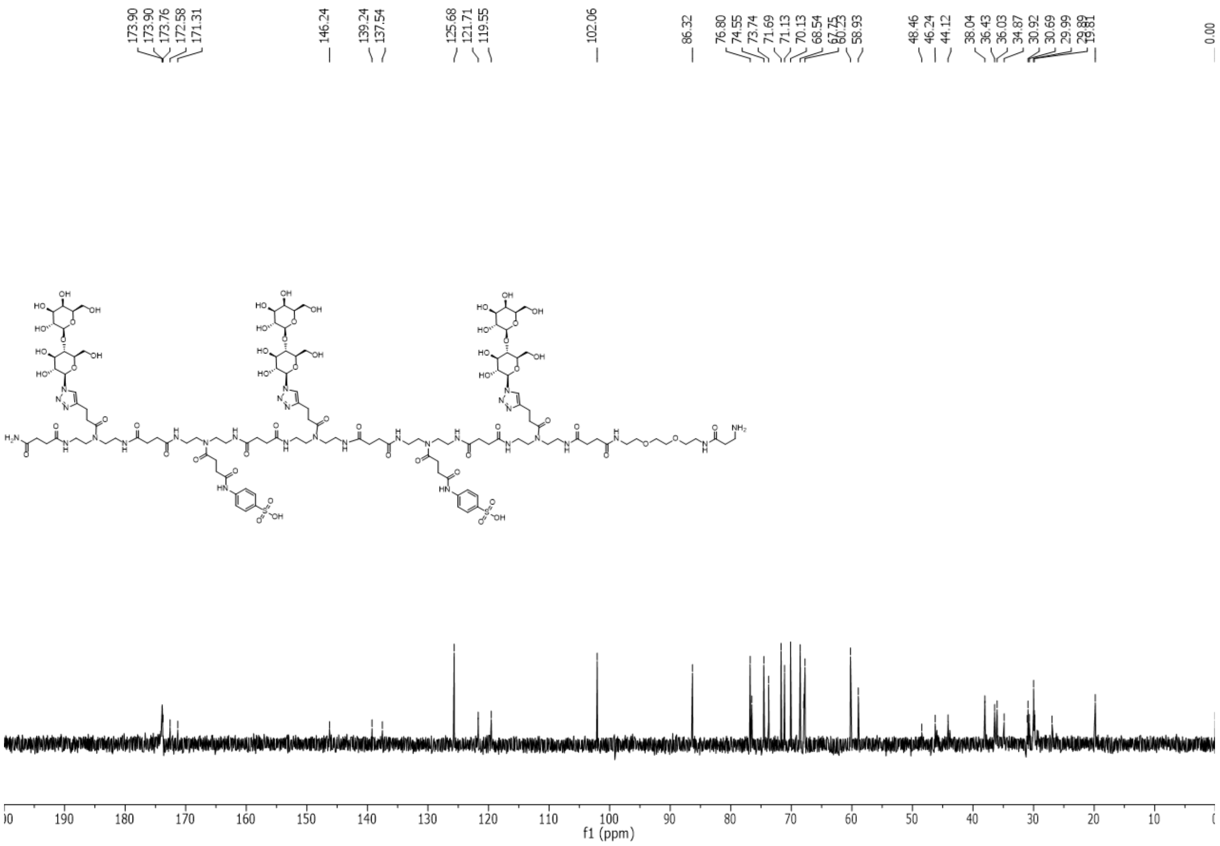

Figure S 46: ^{13}C-NMR spectrum of compound **6a**.

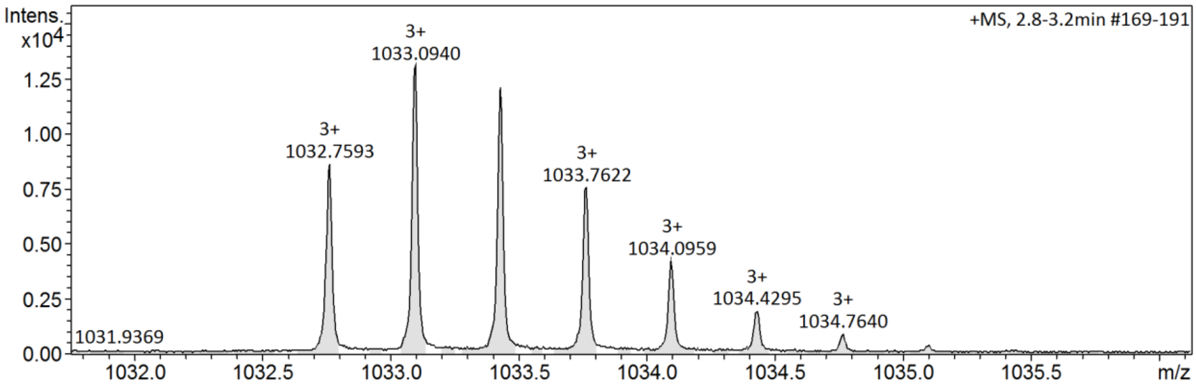

Figure S 47: HR-MS spectrum of compound **6a.**

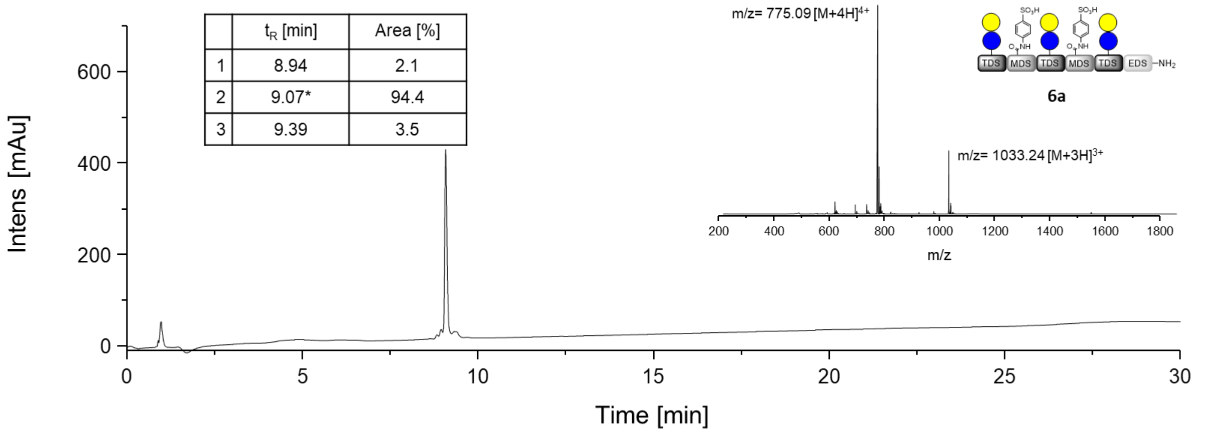

Figure S 48: RP-HPLC chromatogram and ESI$^+$-MS spectrum of compound **6a.** Retention time t_R [min] and area [%] of the peaks are given. ESI-MS spectrum of the main peak (*) is shown.

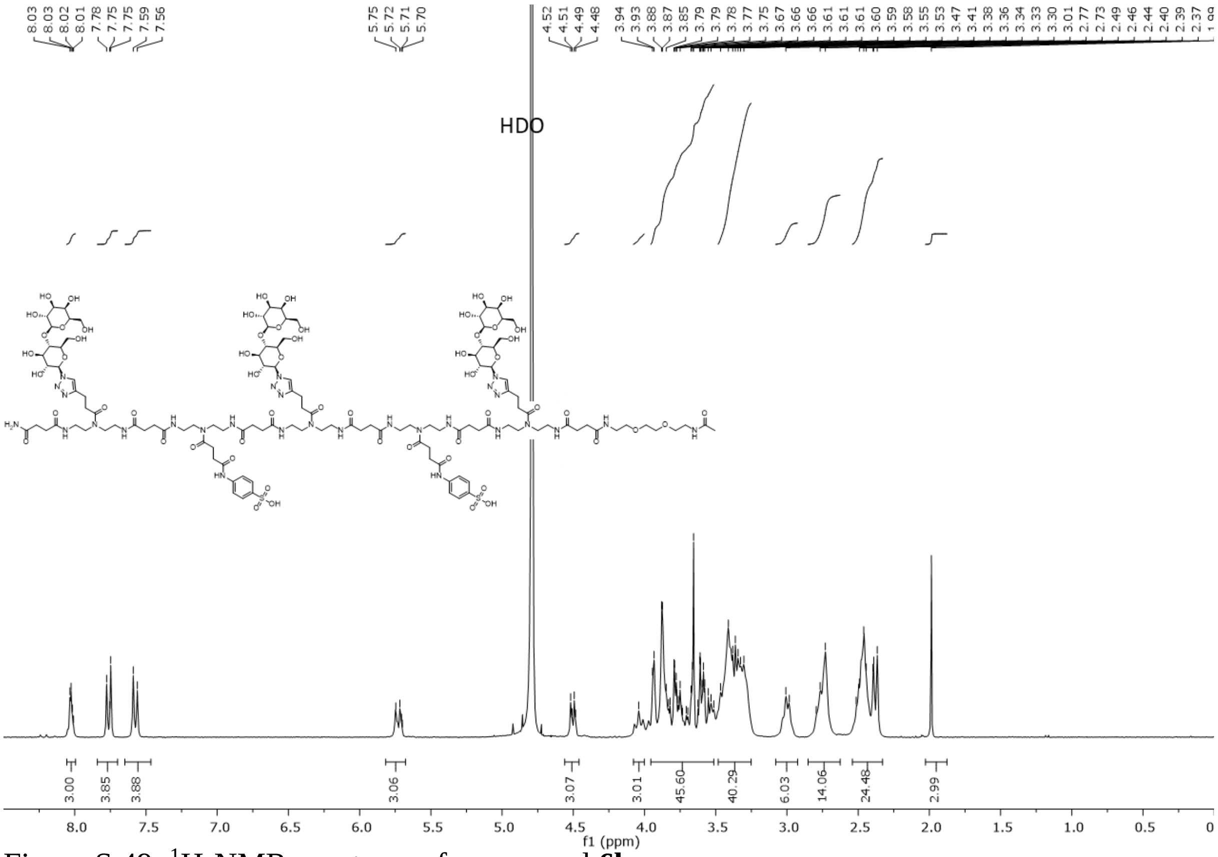

Figure S 49: ^{1}H-NMR spectrum of compound **6b.**

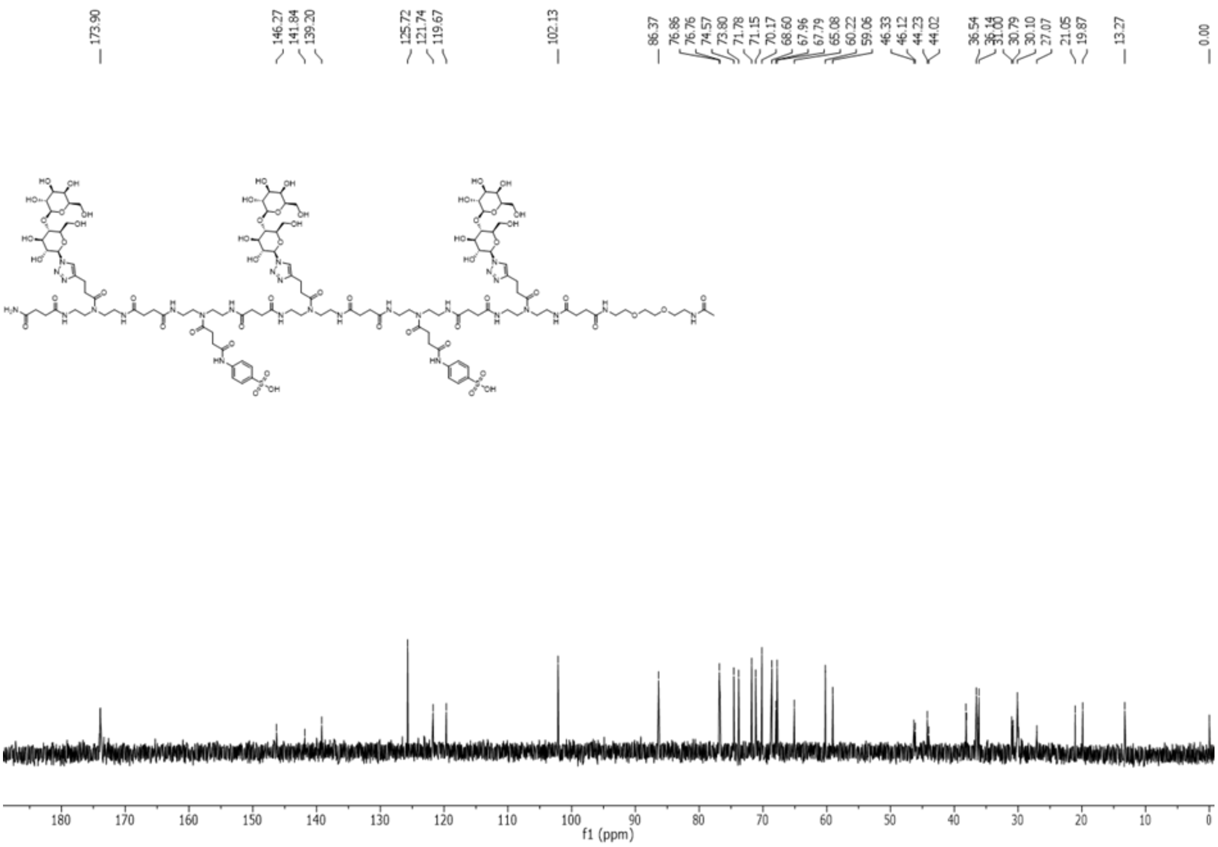

Figure S 50: ^{13}C-NMR spectrum of compound **6b**.

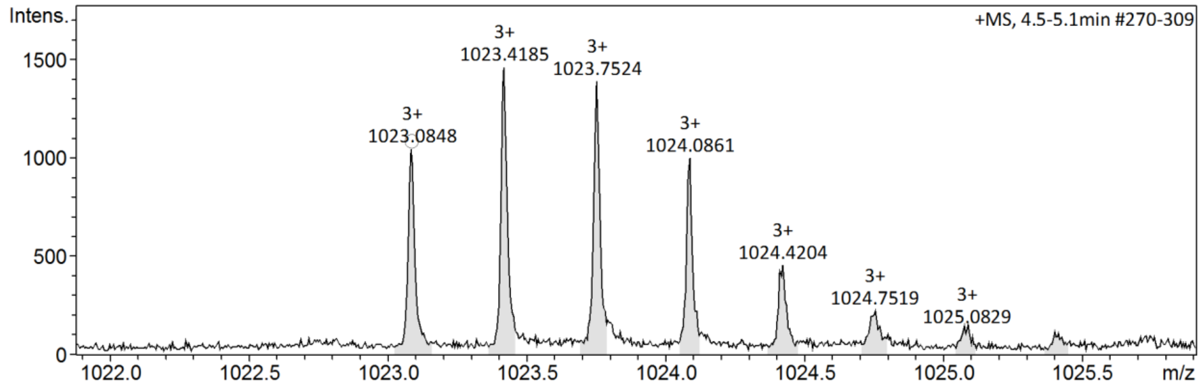

Figure S 51: HR-MS spectrum of compound **6b.**

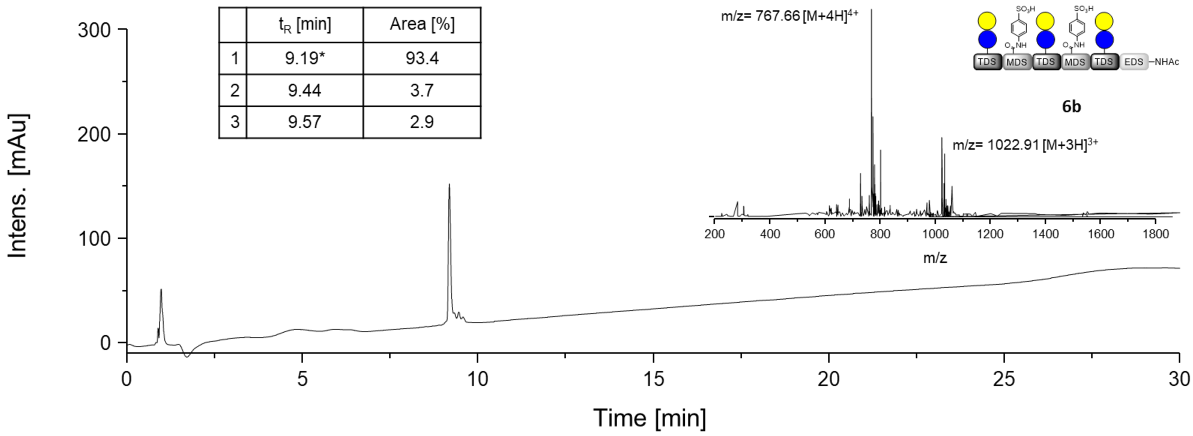

Figure S 52: RP-HPLC chromatogram and ESI$^+$-MS spectrum of compound **6b.** Retention time t_R [min] and area [%] of the peaks are given. ESI-MS spectrum of the main peak (*) is shown.

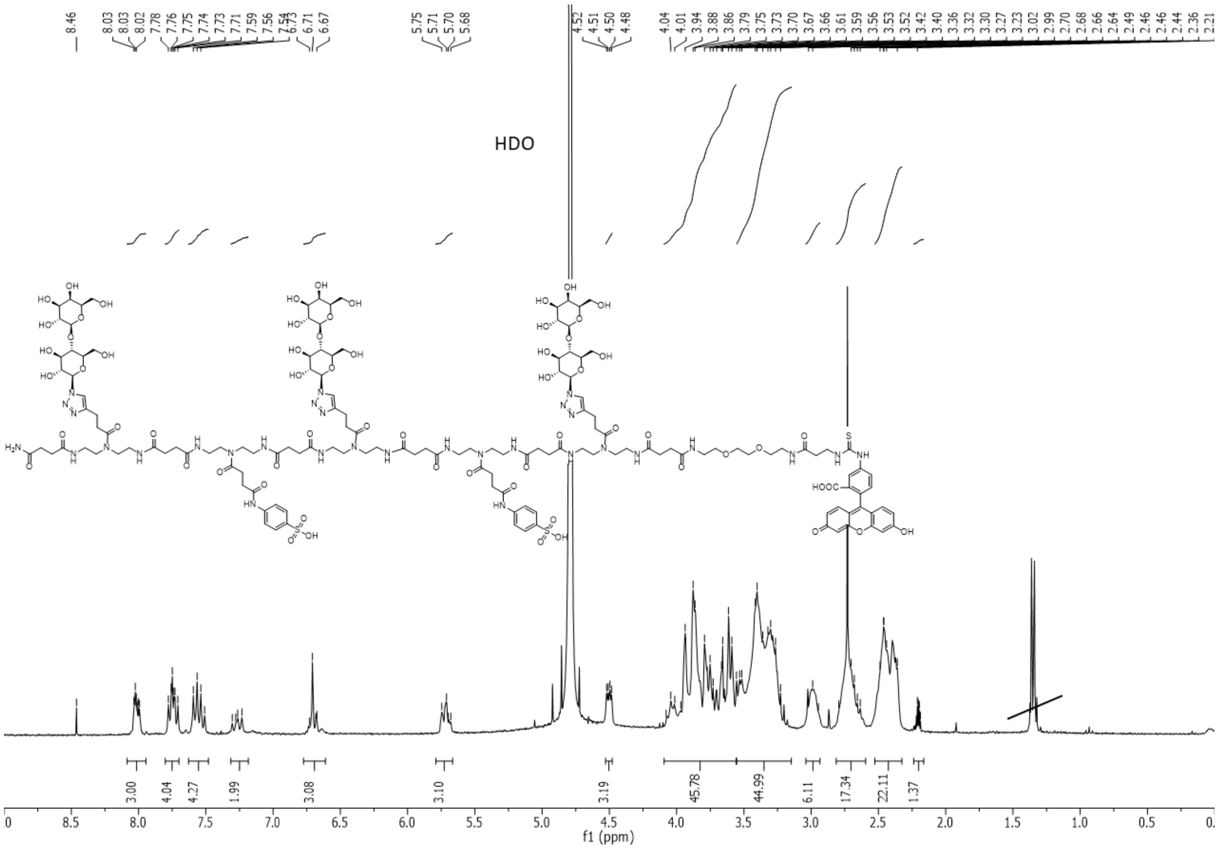

Figure S 53: ^{1}H-NMR spectrum of compound **6c.**

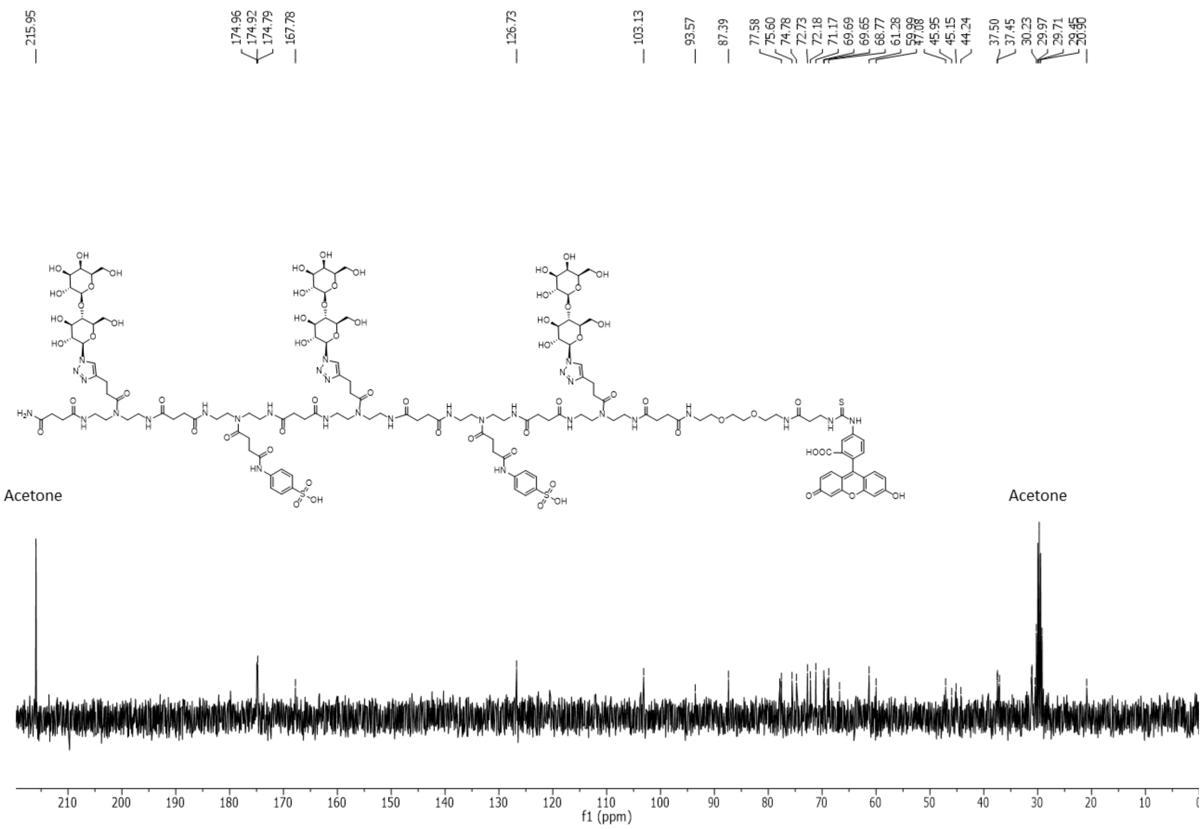

Figure S 54: ^{13}C-NMR spectrum of compound **6c**.

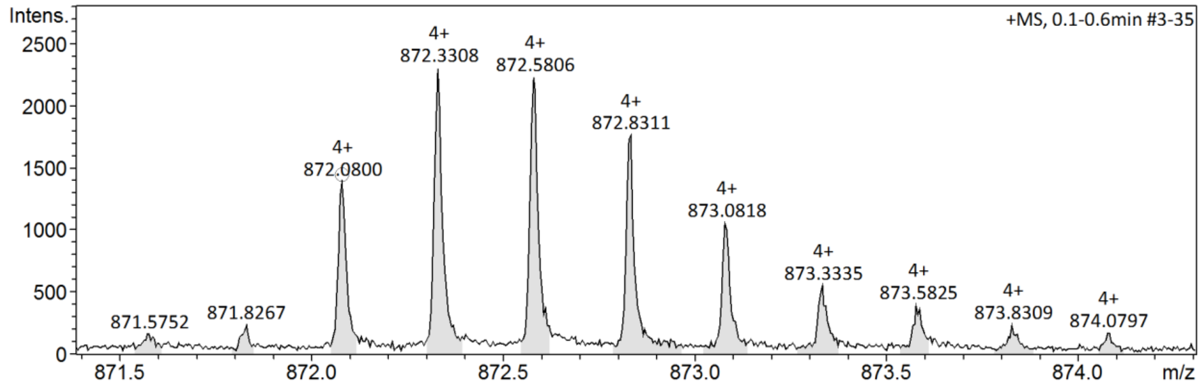

Figure S 55: HR-MS spectrum of compound **6c.**

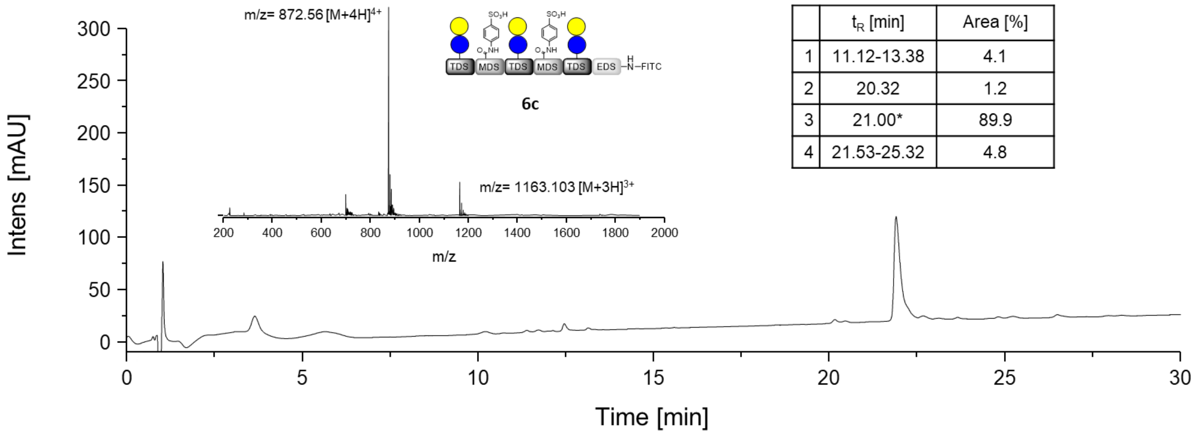

	t_R [min]	Area [%]
1	11.12-13.38	4.1
2	20.32	1.2
3	21.00*	89.9
4	21.53-25.32	4.8

Figure S 56: RP-HPLC chromatogram and ESI$^+$-MS spectrum of compound **6c.** Retention time t_R [min] and area [%] of the peaks are given. ESI-MS spectrum of the main peak (*) is shown.

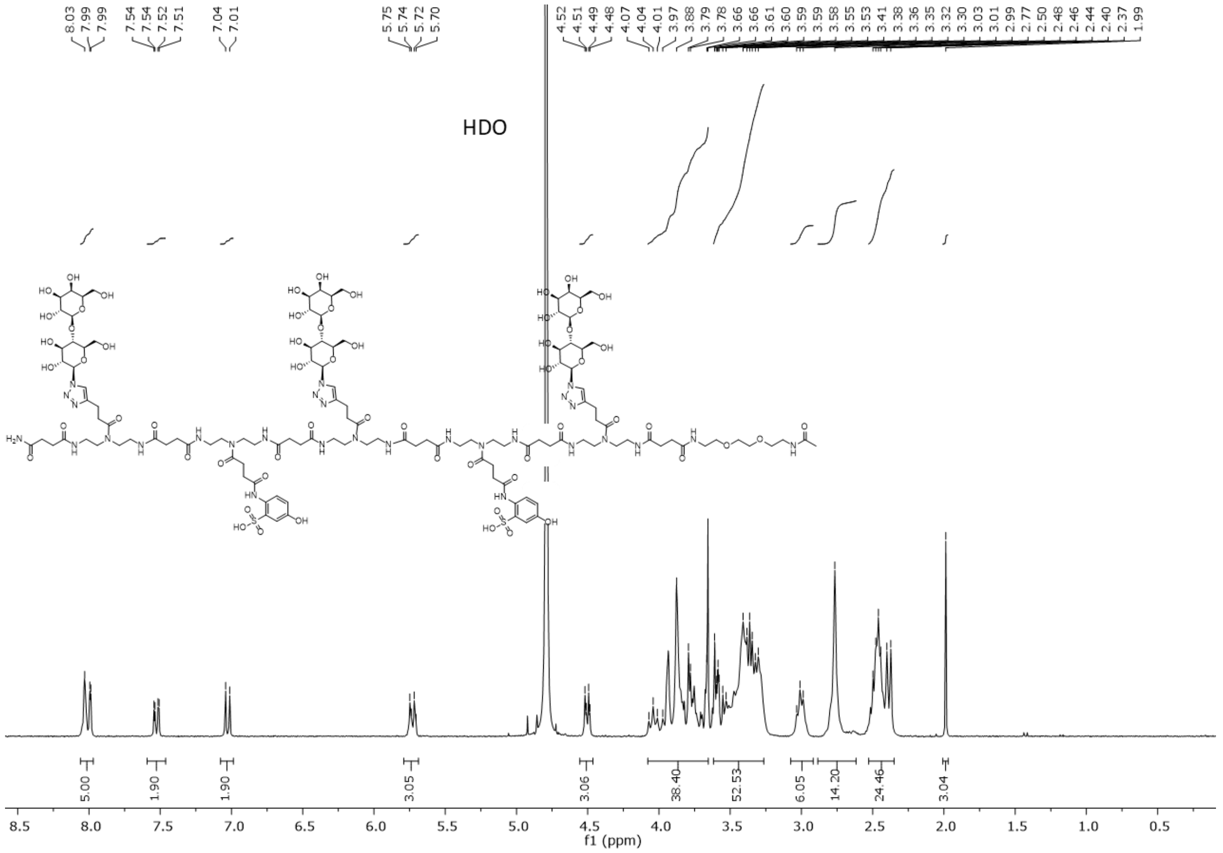

Figure S 57: ^{1}H-NMR spectrum of compound **7b**.

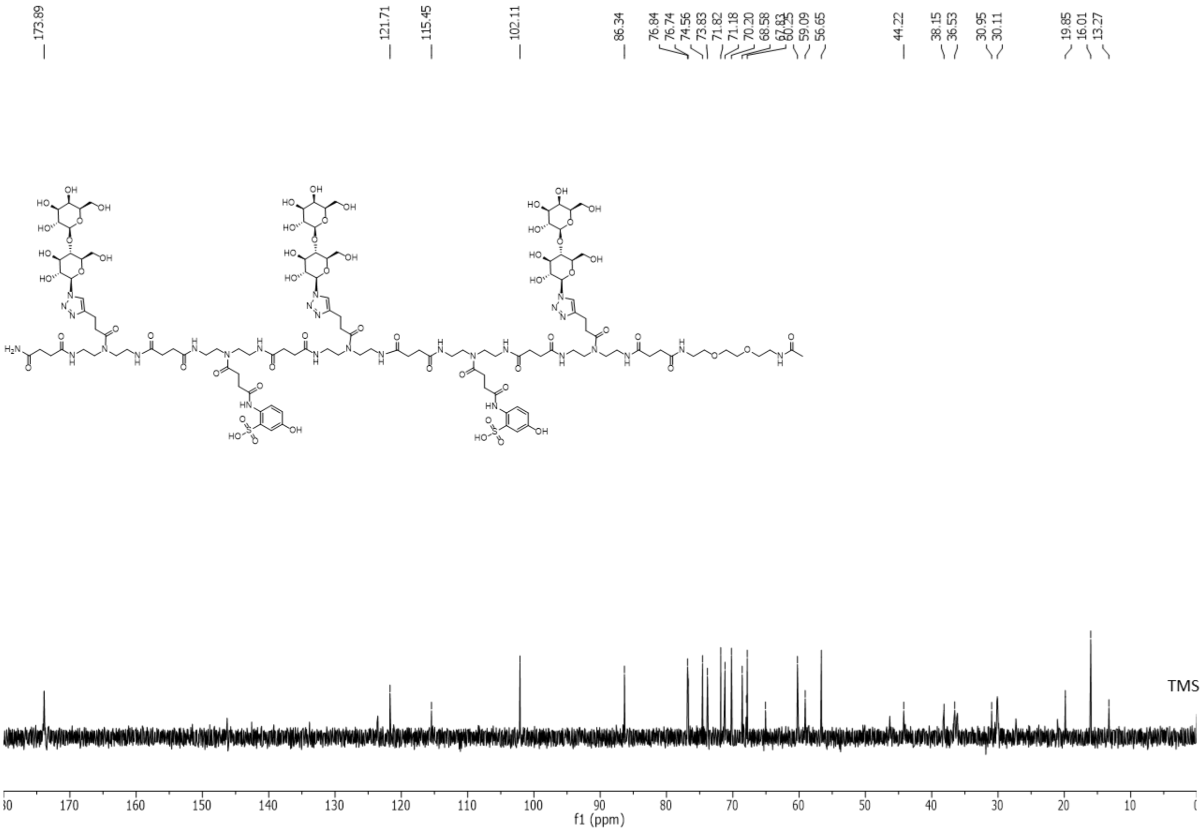

Figure S 58: ^{13}C-NMR spectrum of compound **7b**.

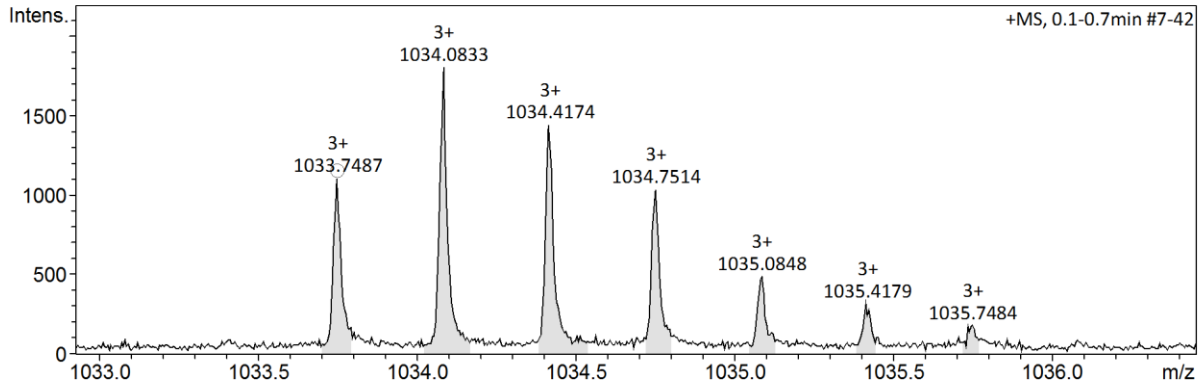

Figure S 59: HR-MS spectrum of compound **7b.**

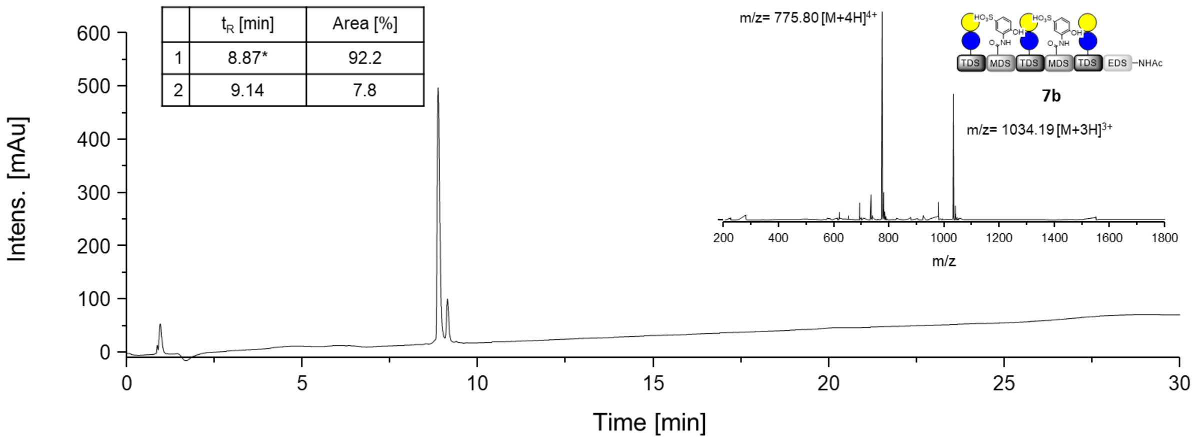

Figure S 60: RP-HPLC chromatogram and ESI^{+}-MS spectrum of compound **7b.** Retention time t_R [min] and area [%] of the peaks are given. ESI-MS spectrum of the main peak (*) is shown.

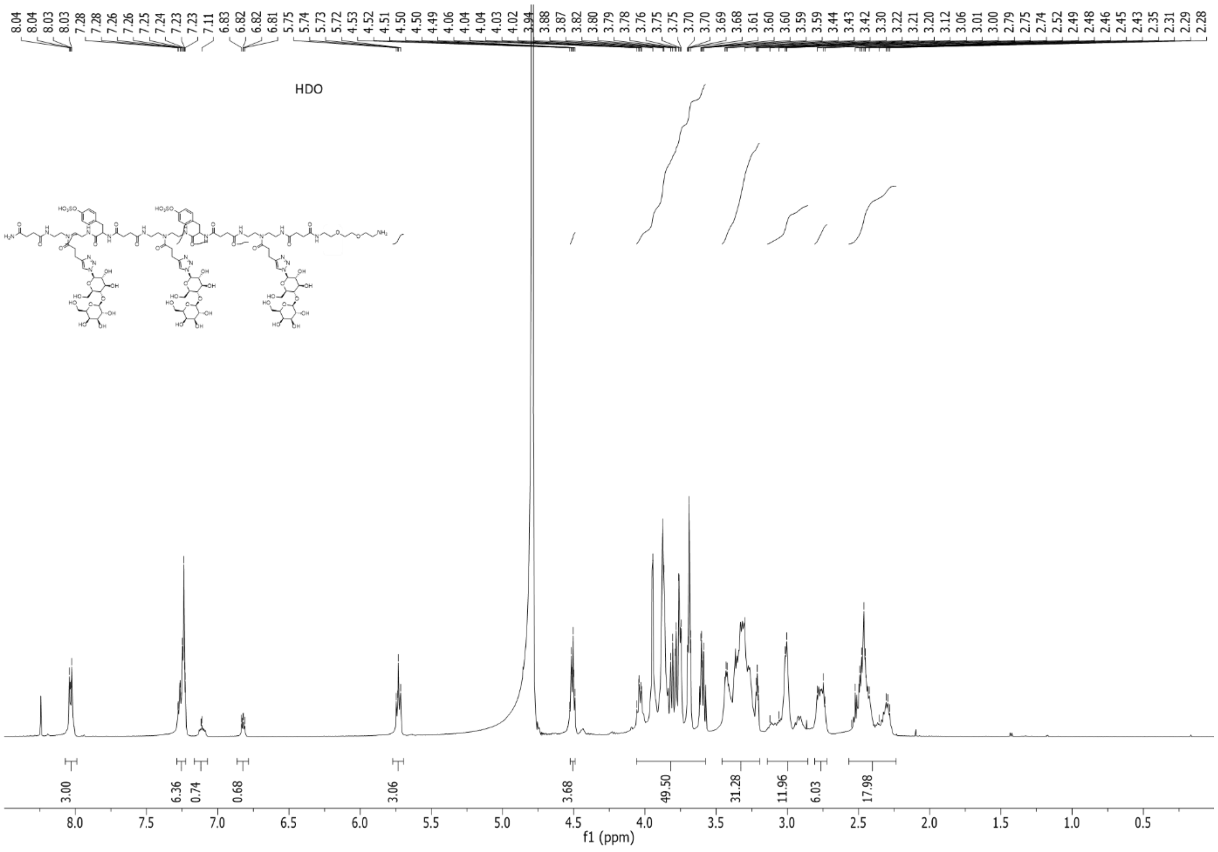

Figure S 61: ^{1}H-NMR spectrum of compound **8a**.

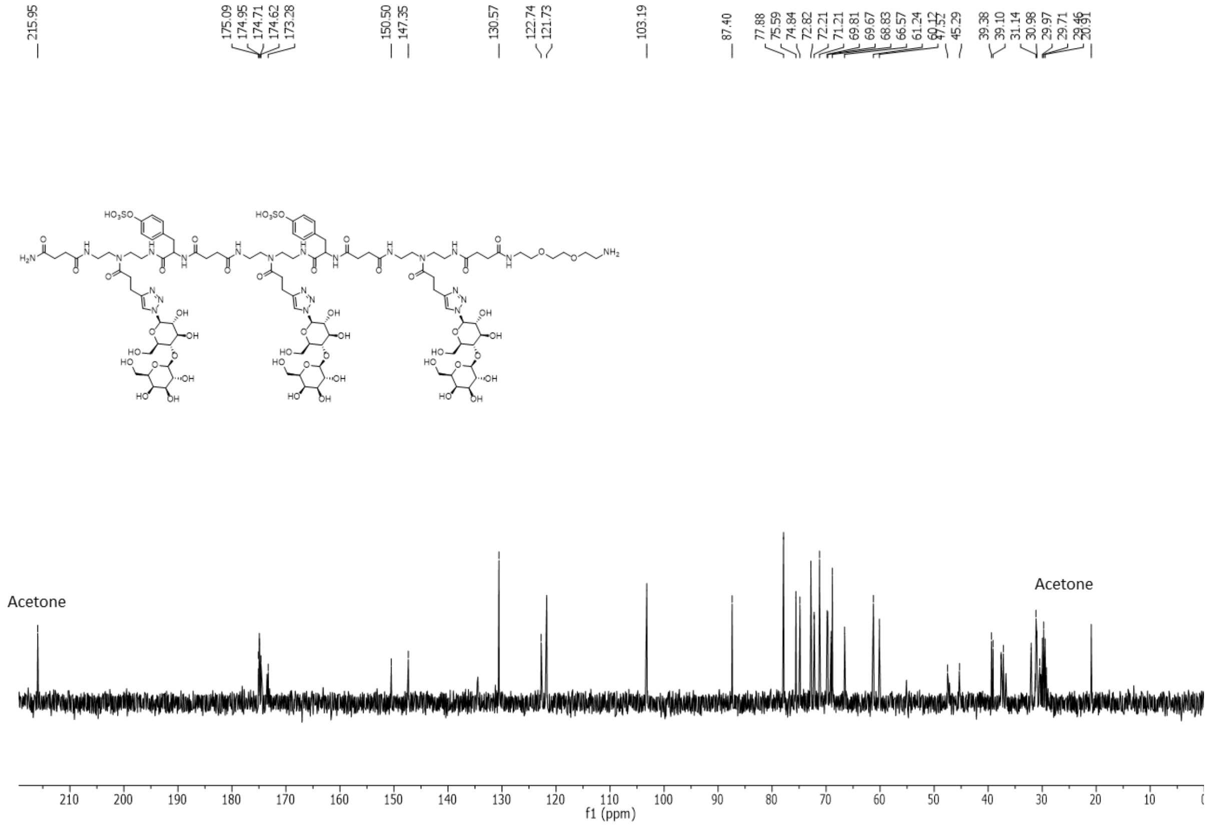

Figure S 62: ^{13}C-NMR spectrum of compound **8a.**

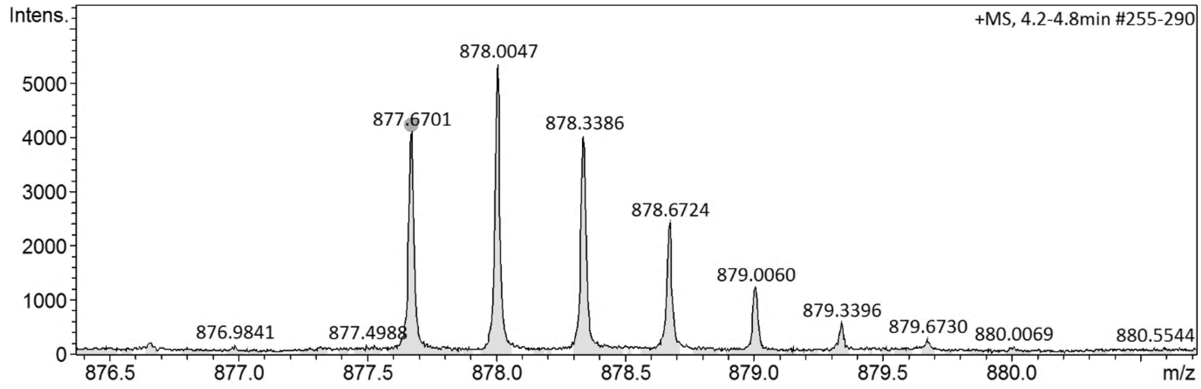

Figure S 63: HR-MS spectrum of compound **8a.**

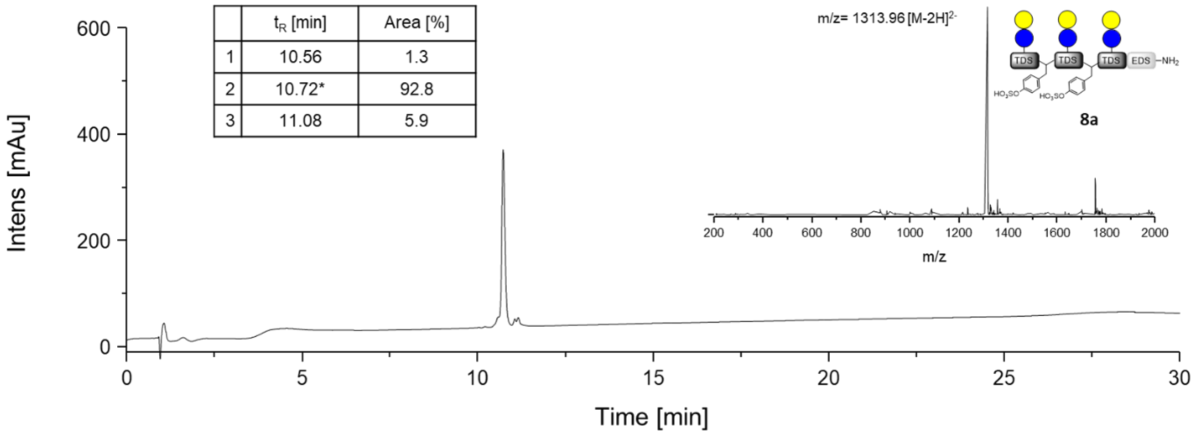

	t_R [min]	Area [%]
1	10.56	1.3
2	10.72*	92.8
3	11.08	5.9

Figure S 64: RP-HPLC chromatogram and ESI$^+$-MS spectrum of compound **8a.** Retention time t_R [min] and area [%] of the peaks are given. ESI-MS spectrum of the main peak (*) is shown.

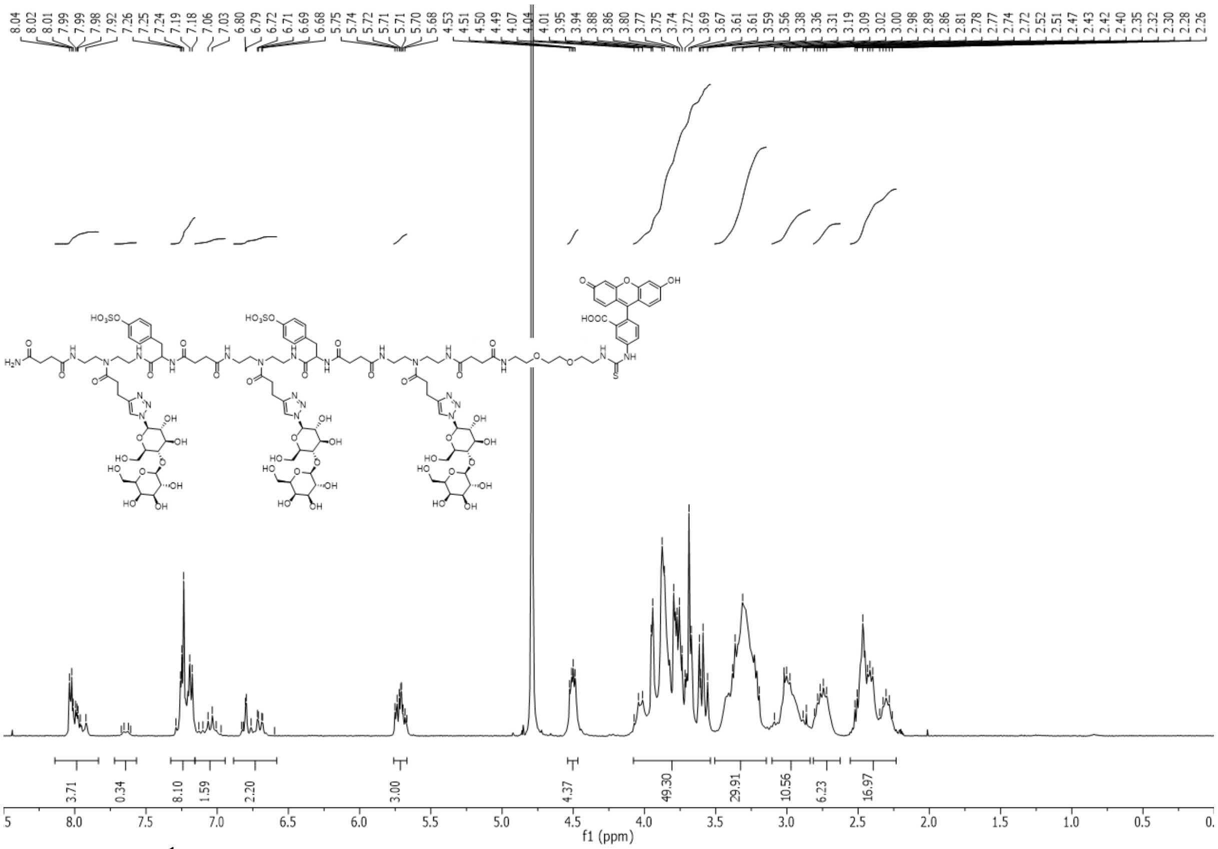

Figure S 65: ^{1}H-NMR spectrum of compound **8c.**

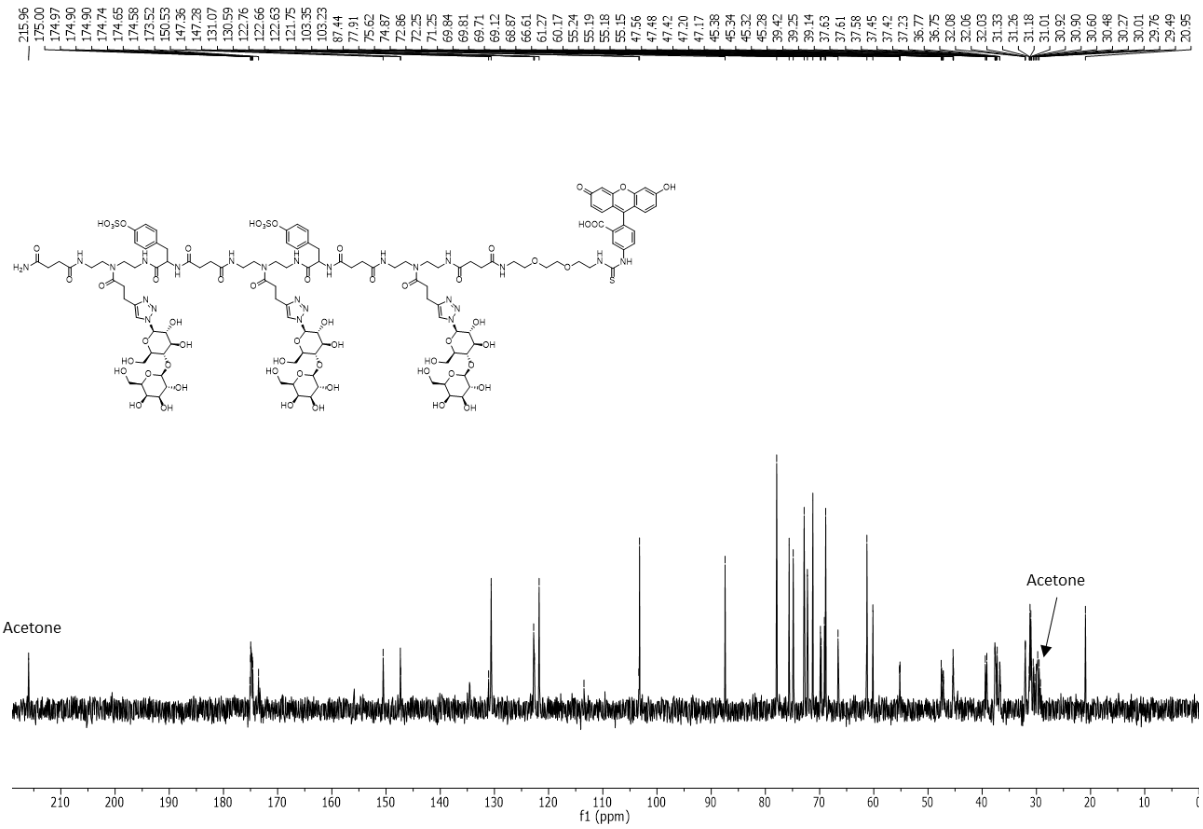

Figure S 66: ^{13}C-NMR spectrum of compound **8a**.

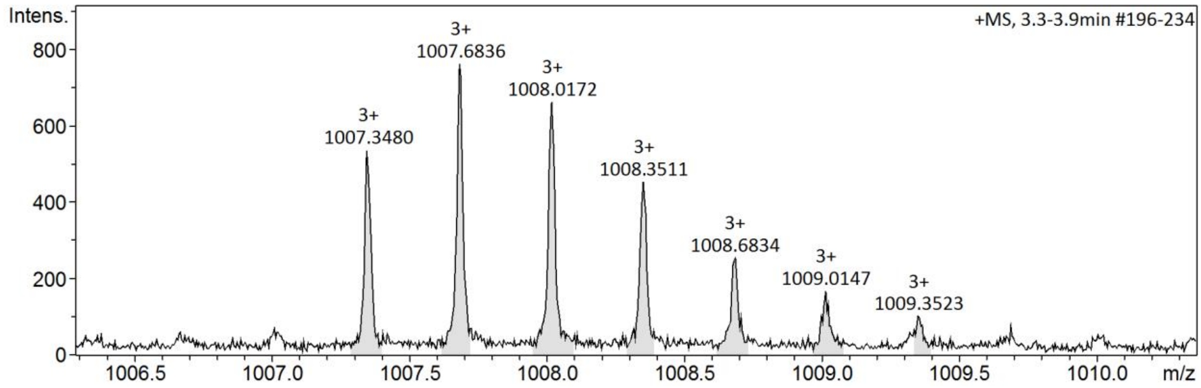

Figure S 67: HR-MS spectrum of compound **8c.**

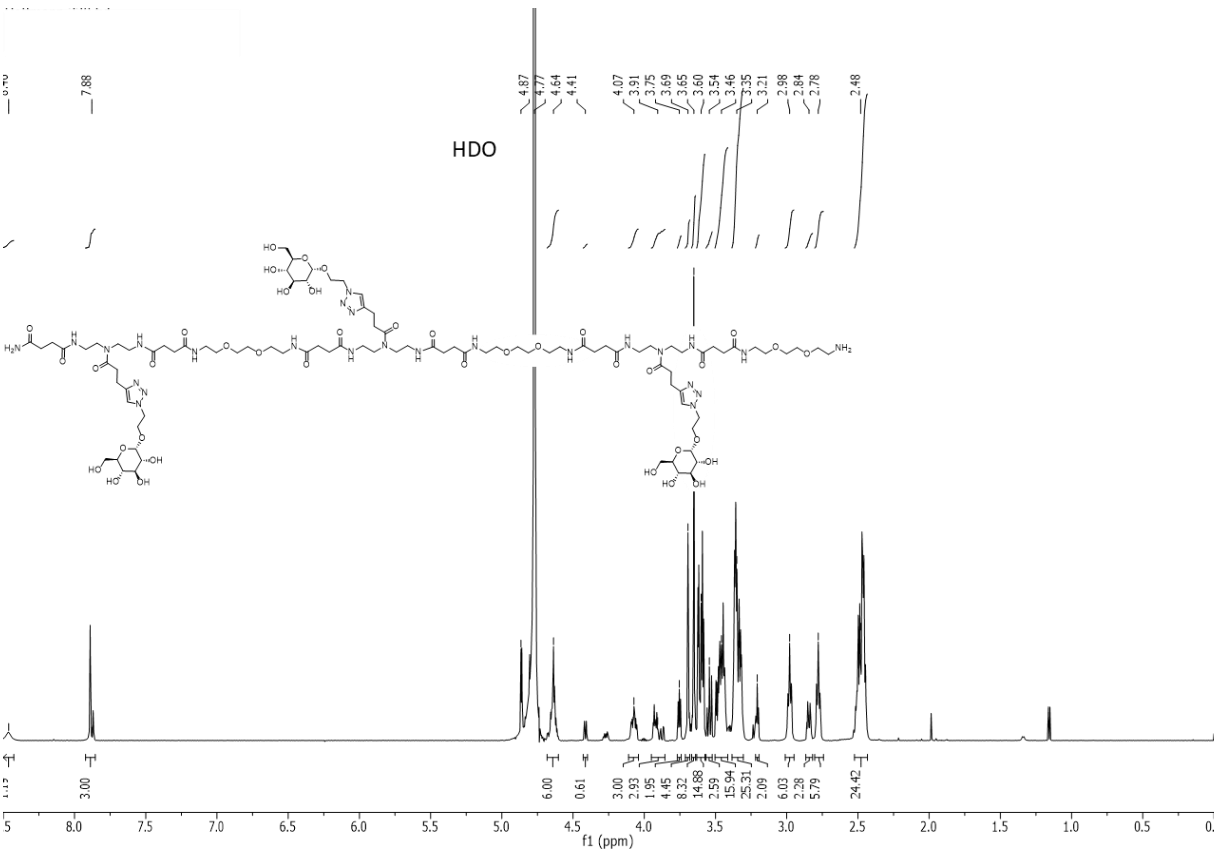

Figure S 68: ^{1}H-NMR spectrum of compound **9a.**

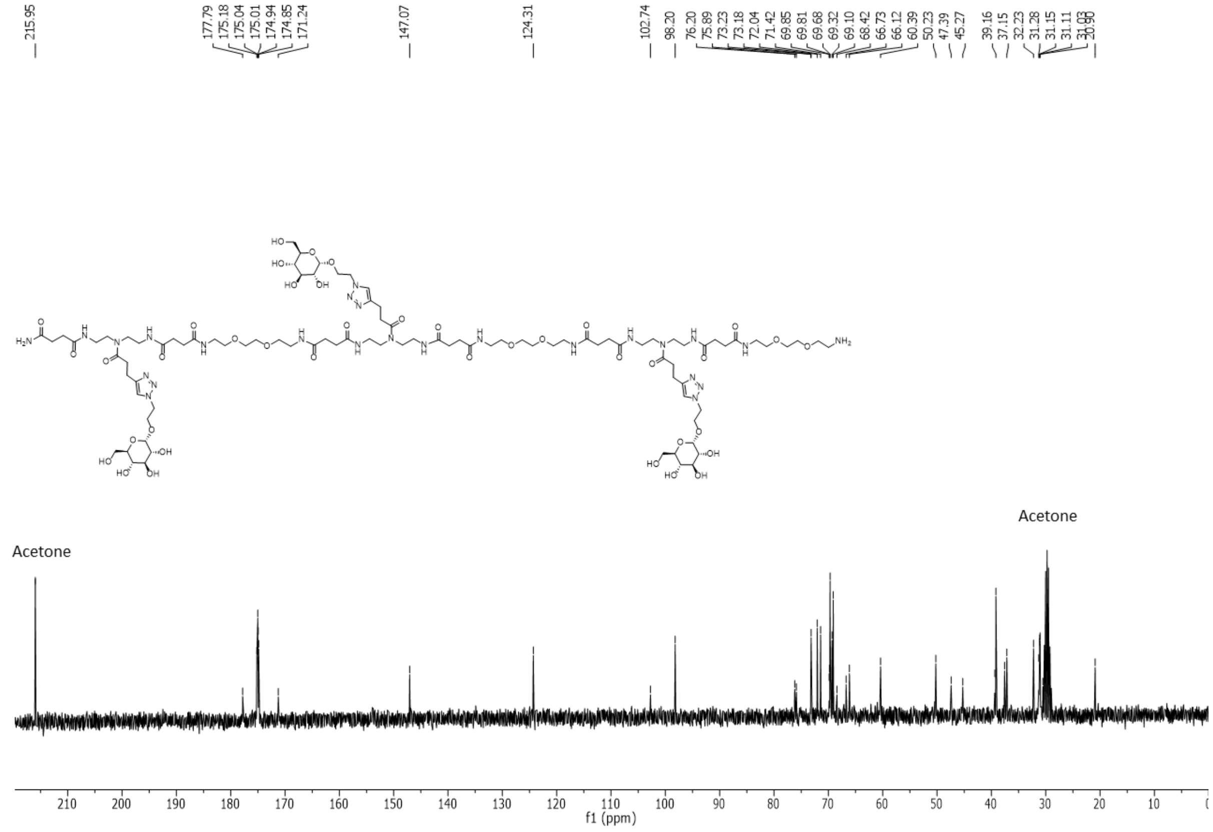

Figure S 69: ^{13}C-NMR spectrum of compound **9a**.

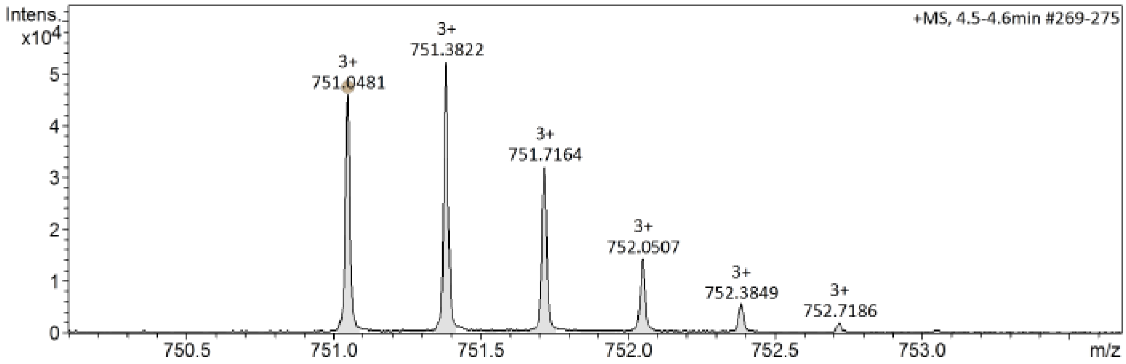

Figure S 70: HR-MS spectrum of compound **9a**.

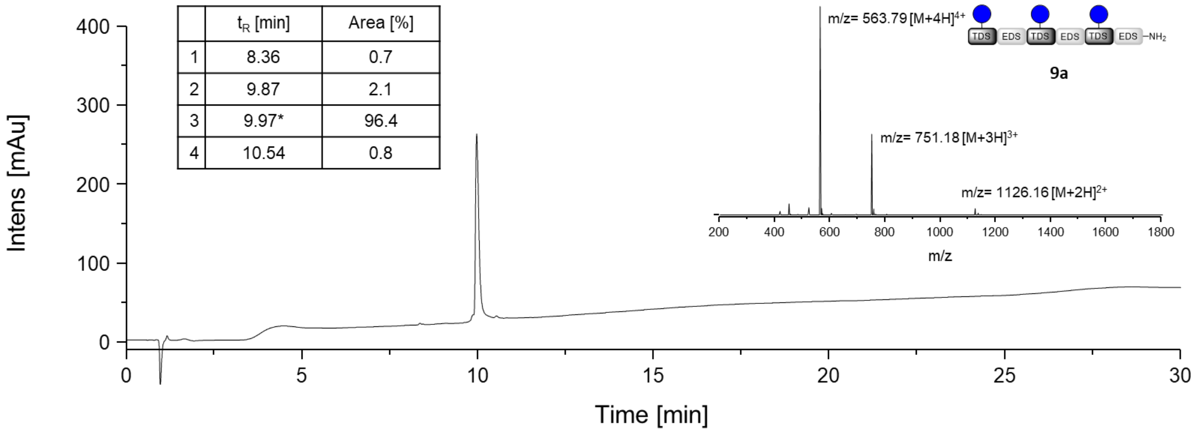

	t_R [min]	Area [%]
1	8.36	0.7
2	9.87	2.1
3	9.97*	96.4
4	10.54	0.8

Figure S 71: RP-HPLC chromatogram and ESI$^+$-MS spectrum of compound **9a.** Retention time t_R [min] and area [%] of the peaks are given. ESI-MS spectrum of the main peak (*) is shown.

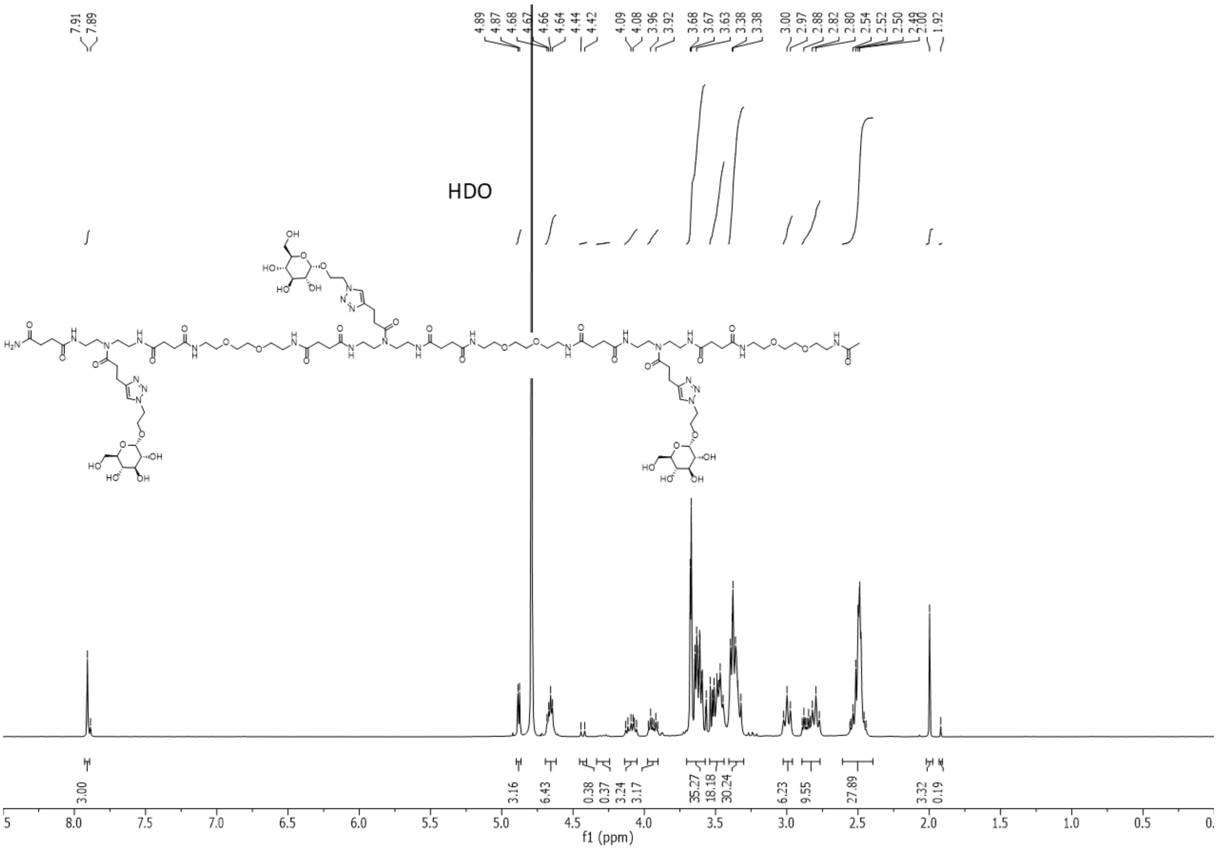

Figure S 72: ^{1}H-NMR spectrum of compound **9b.**

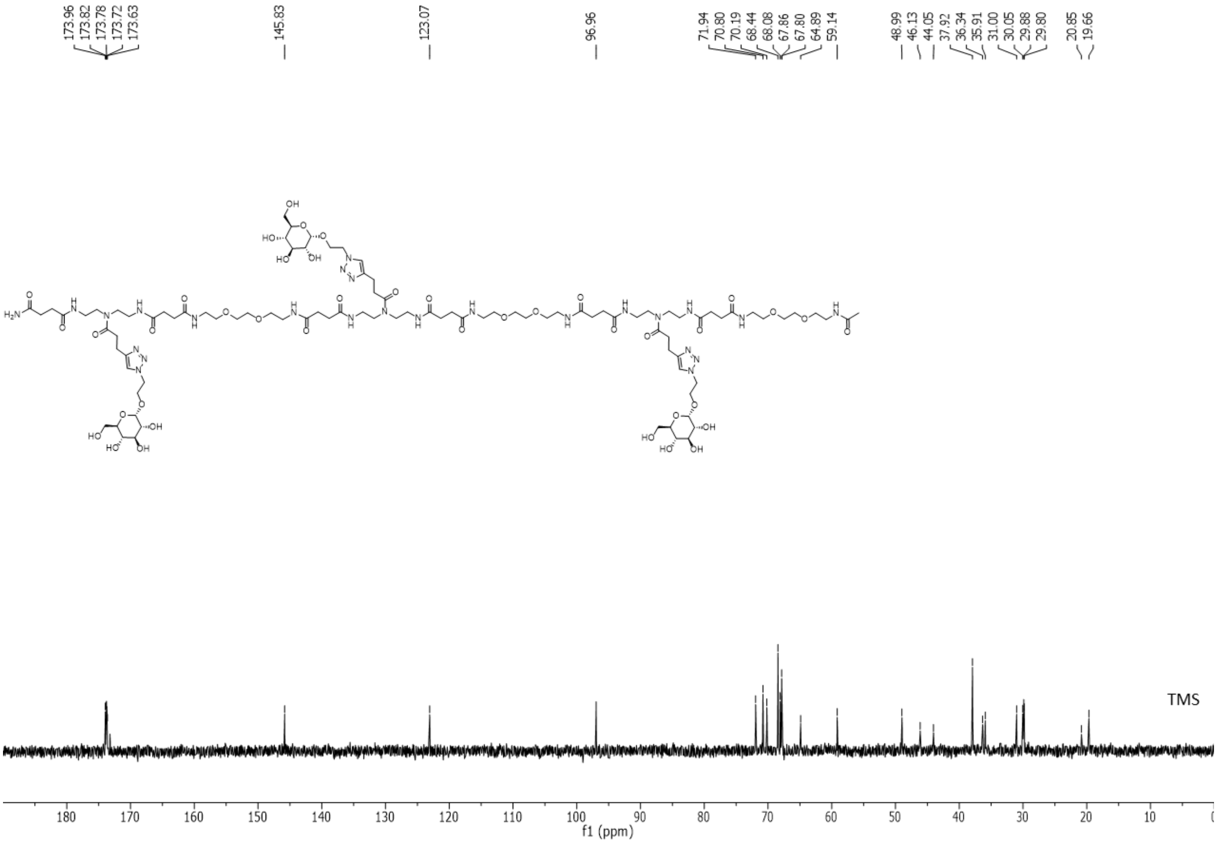

Figure S 73: ^{13}C-NMR spectrum of compound **9b**.

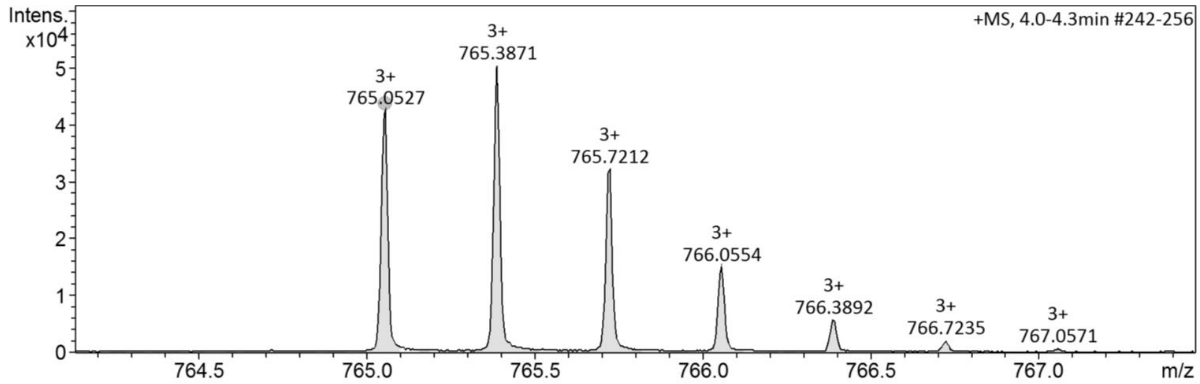

Figure S 74: HR-MS spectrum of compound **9b.**

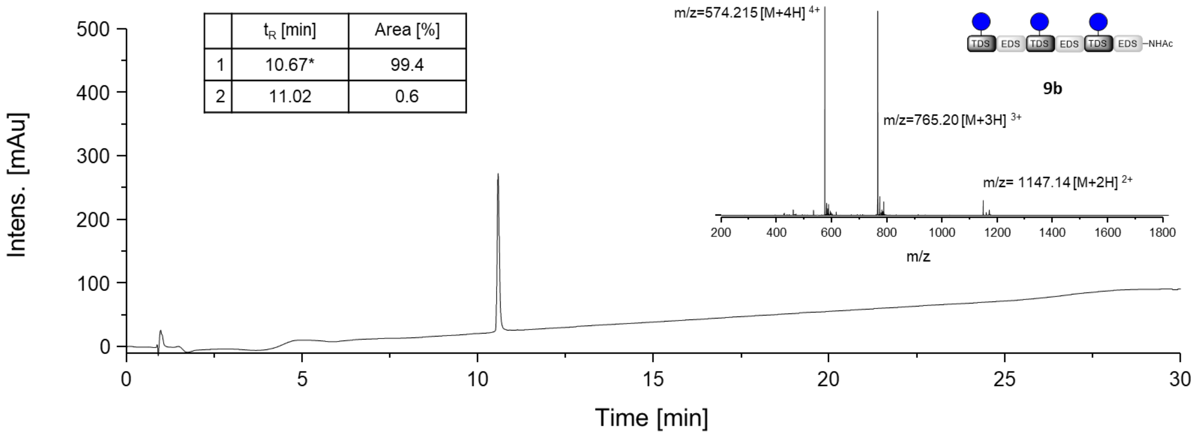

Figure S 75: RP-HPLC chromatogram and ESI$^+$-MS spectrum of compound **9b.** Retention time t_R [min] and area [%] of the peaks are given. ESI-MS spectrum of the main peak (*) is shown.

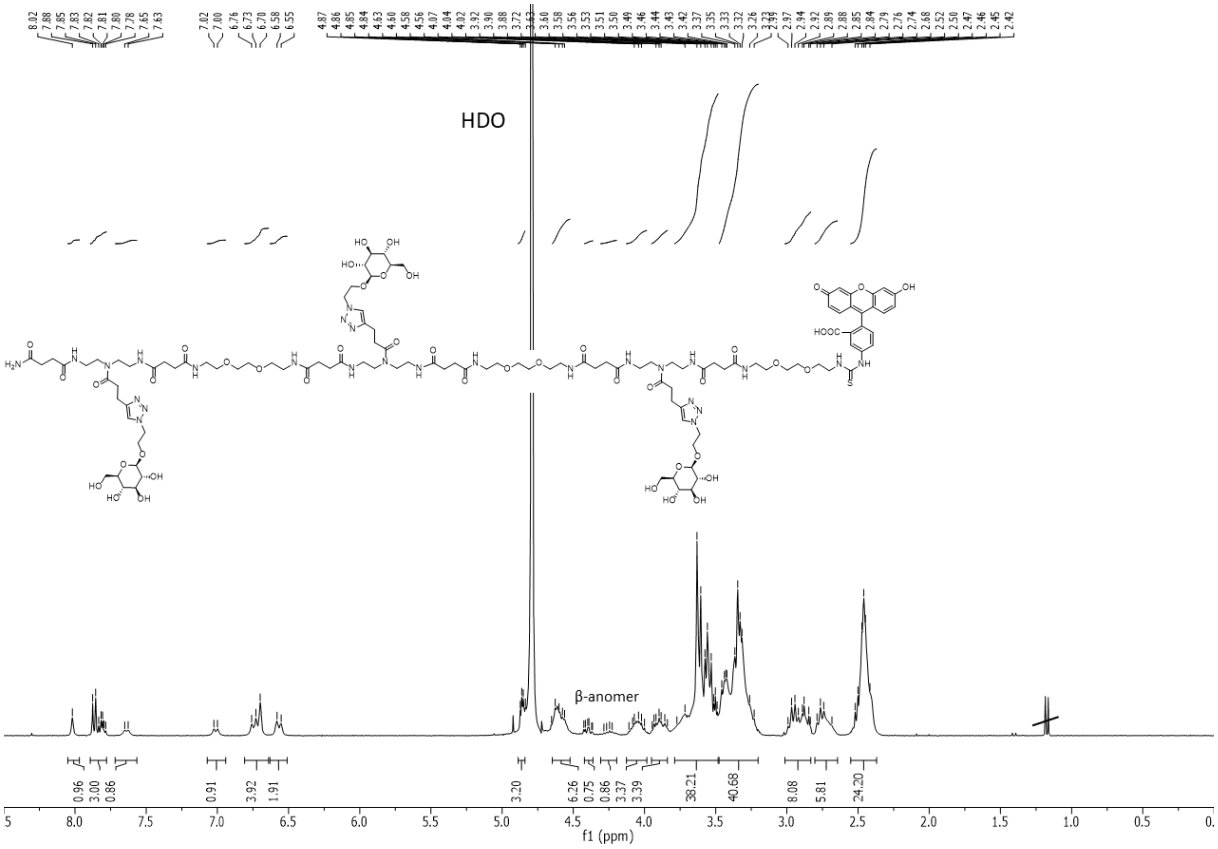

Figure S 76: ^{1}H-NMR spectrum of compound **9c**.

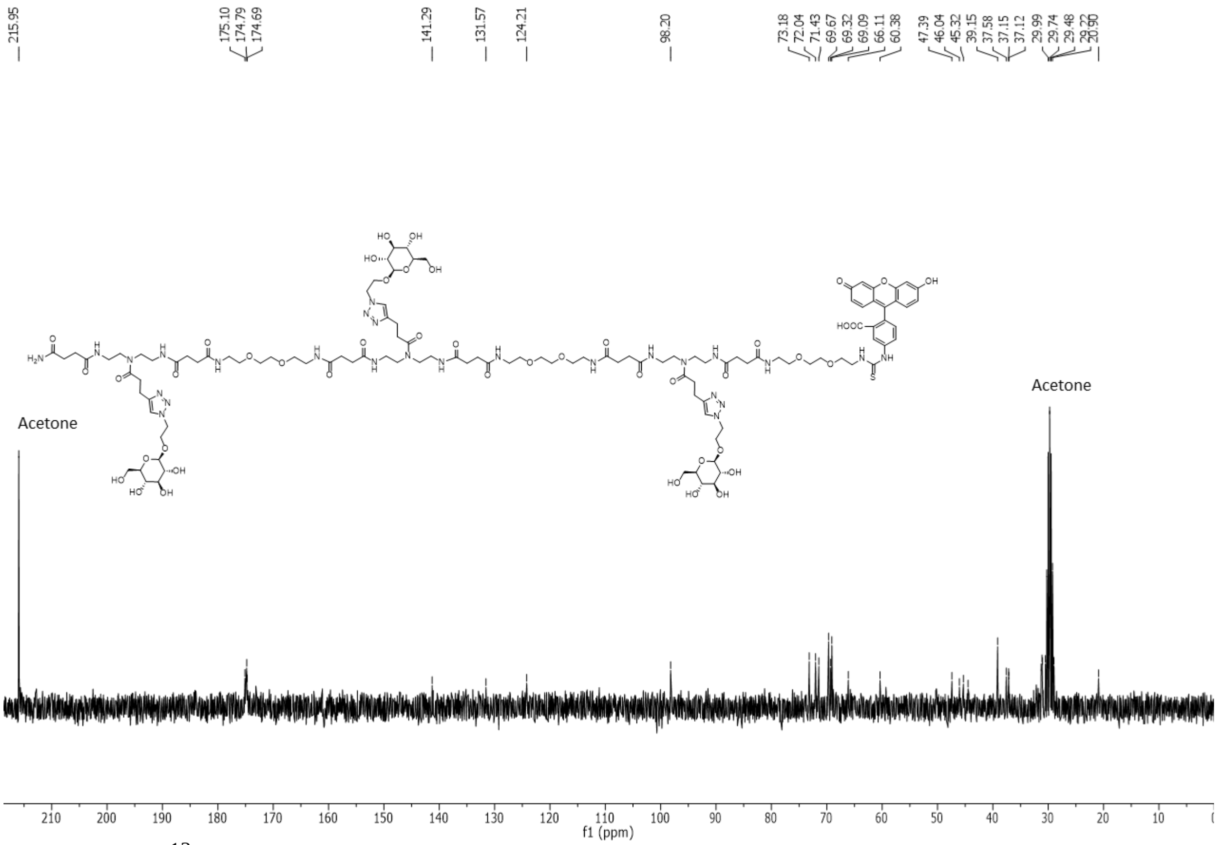

Figure S 77: ^{13}C-NMR spectrum of compound **9c**.

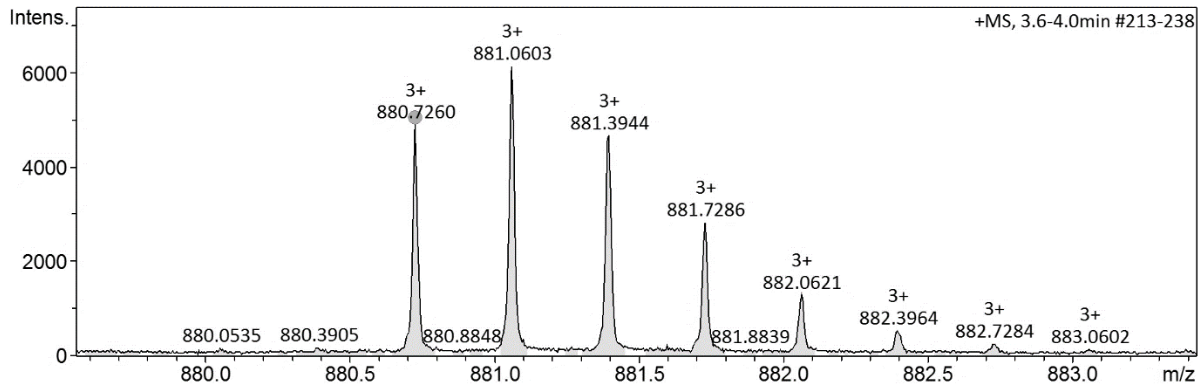

Figure S 78: HR-MS spectrum of compound **9c.**

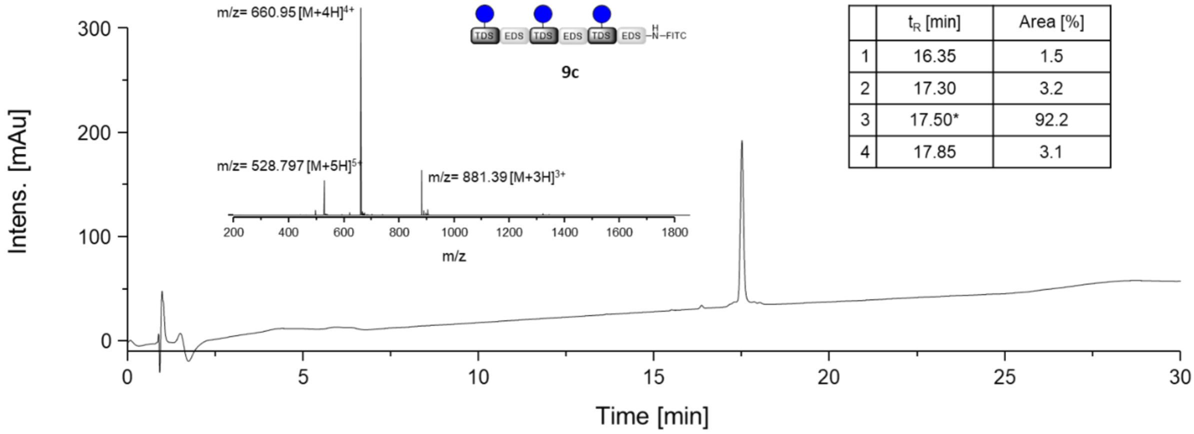

	t_R [min]	Area [%]
1	16.35	1.5
2	17.30	3.2
3	17.50*	92.2
4	17.85	3.1

Figure S 79: RP-HPLC chromatogram and ESI$^+$-MS spectrum of compound **9c.** Retention time t_R [min] and area [%] of the peaks are given. ESI-MS spectrum of the main peak (*) is shown.

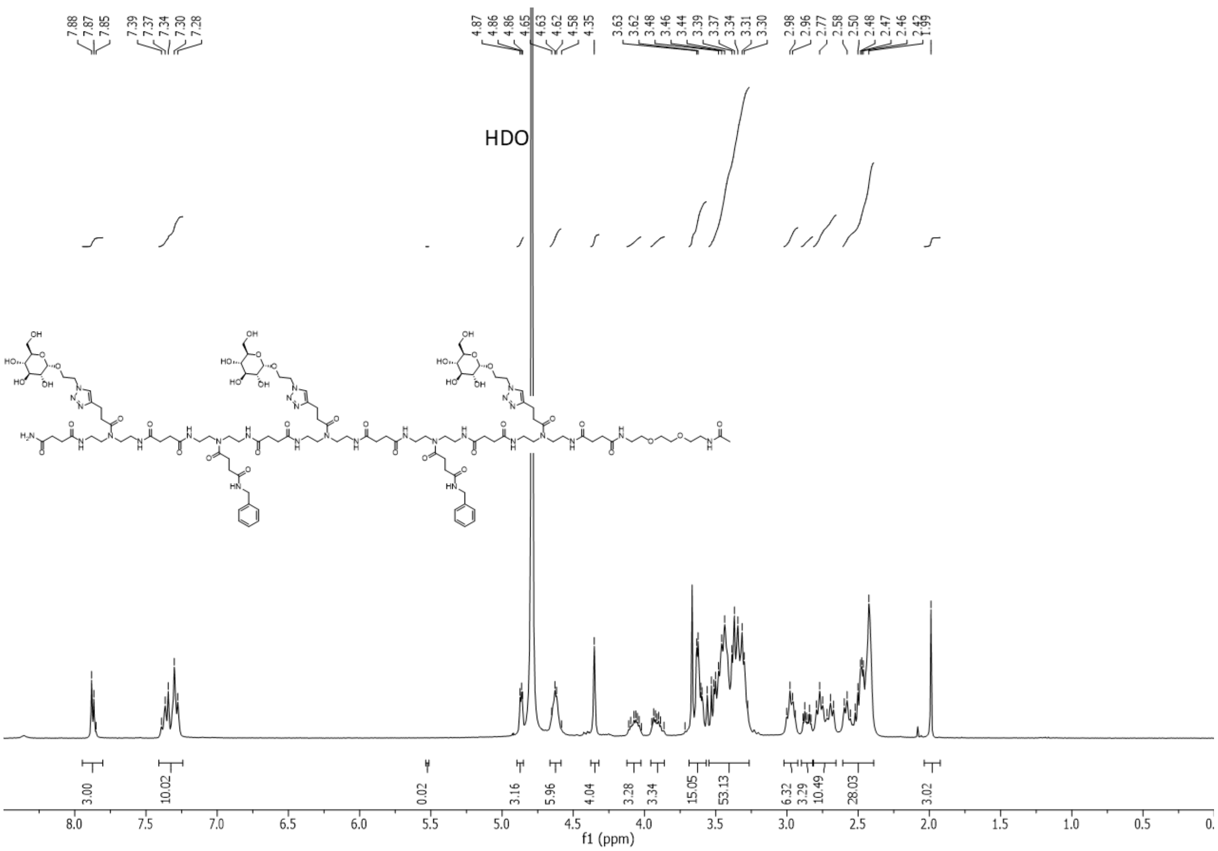

Figure S 80: ^{1}H-NMR spectrum of compound **10b.**

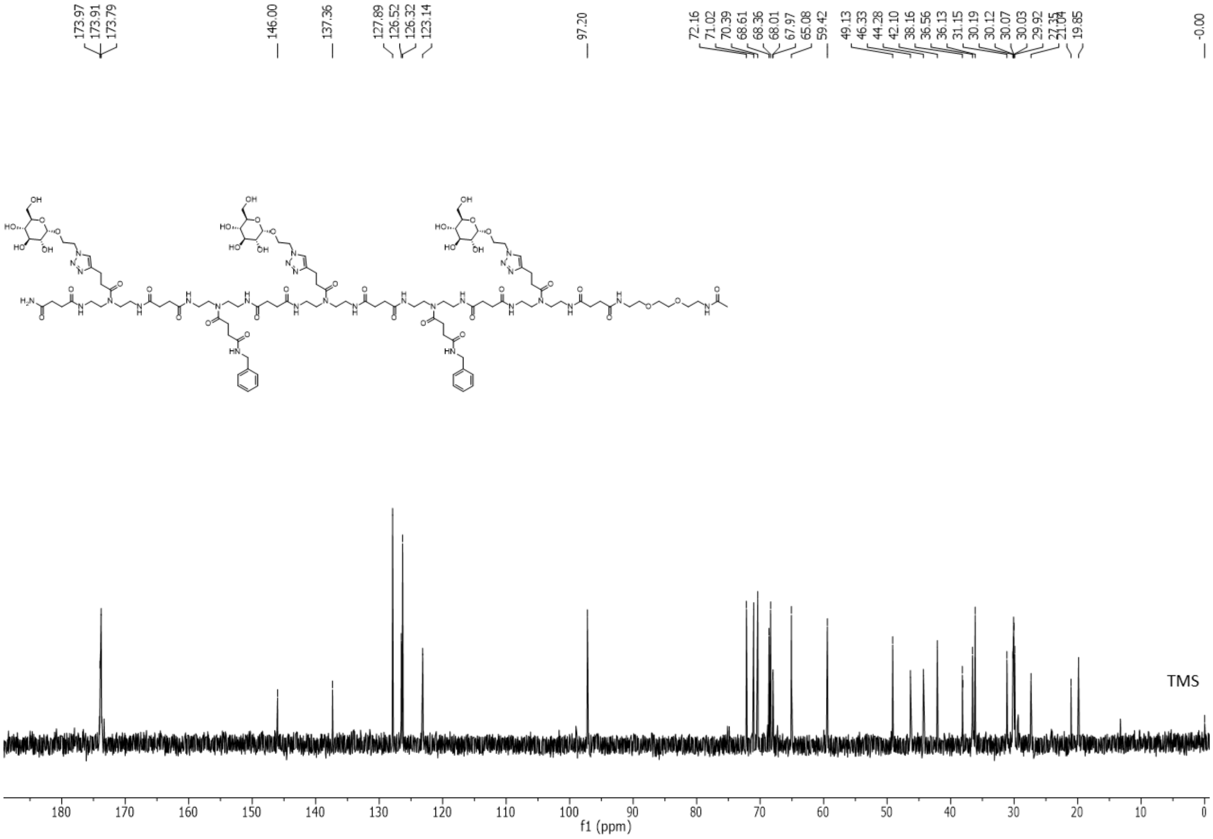

Figure S 81: ^{13}C-NMR spectrum of compound **10b.**

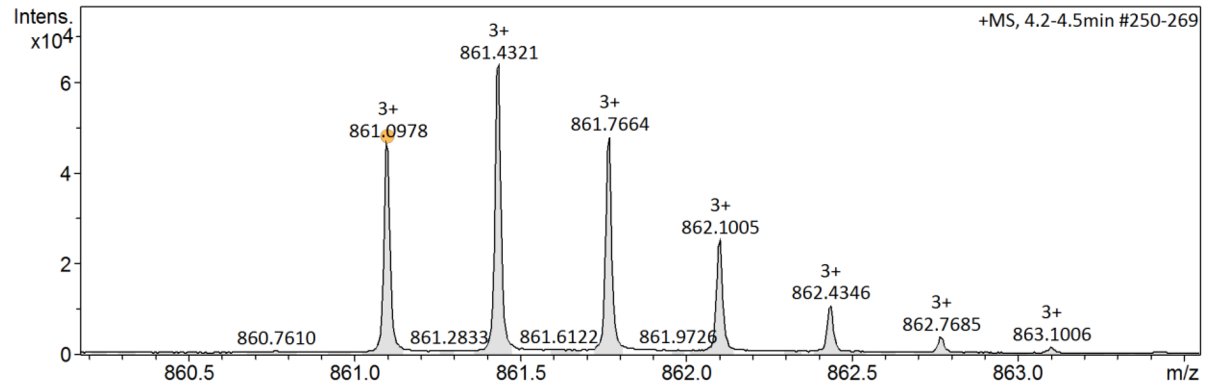

Figure S 82: HR-MS spectrum of compound **10b.**

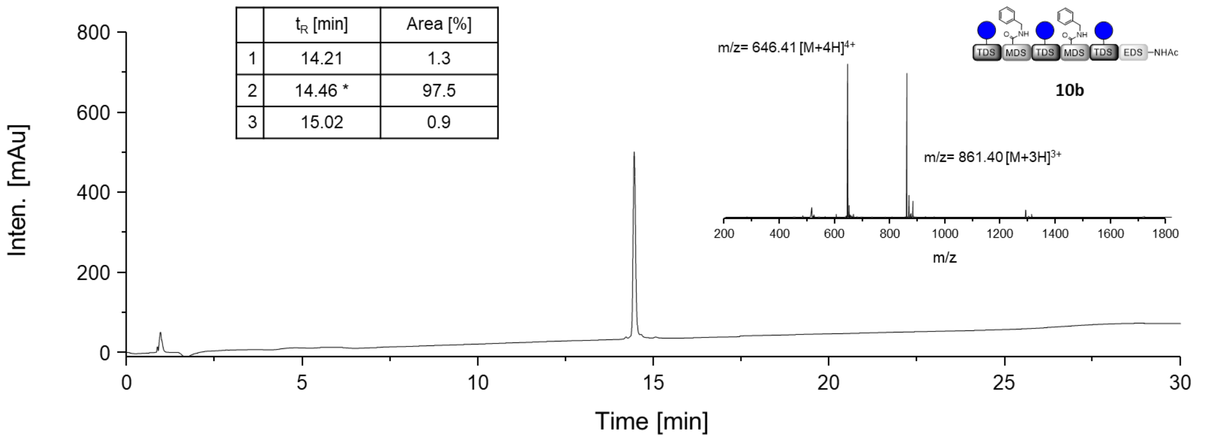

	t_R [min]	Area [%]
1	14.21	1.3
2	14.46 *	97.5
3	15.02	0.9

Figure S 83: RP-HPLC chromatogram and ESI$^+$-MS spectrum of compound **10b.** Retention time t_R [min] and area [%] of the peaks are given. ESI-MS spectrum of the main peak (*) is shown.

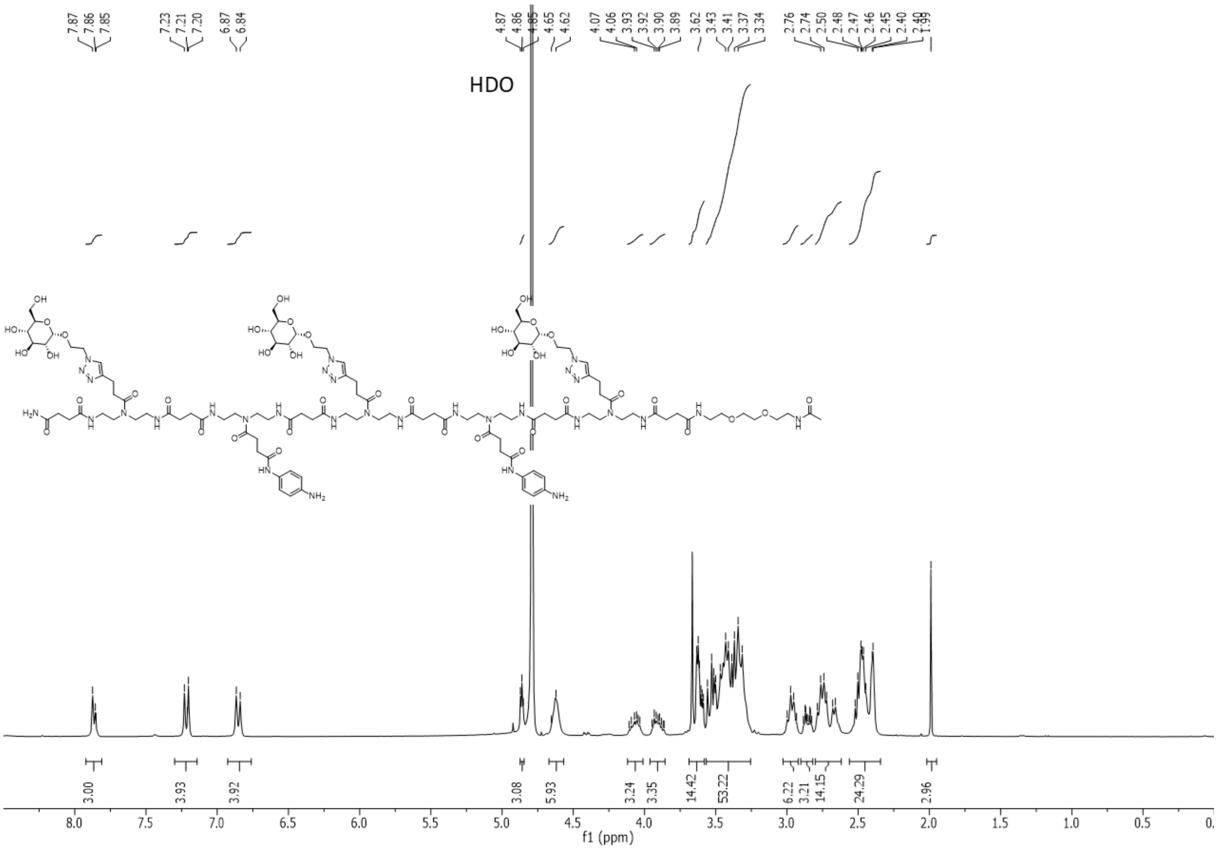

Figure S 84: ^{1}H-NMR spectrum of compound **11b.**

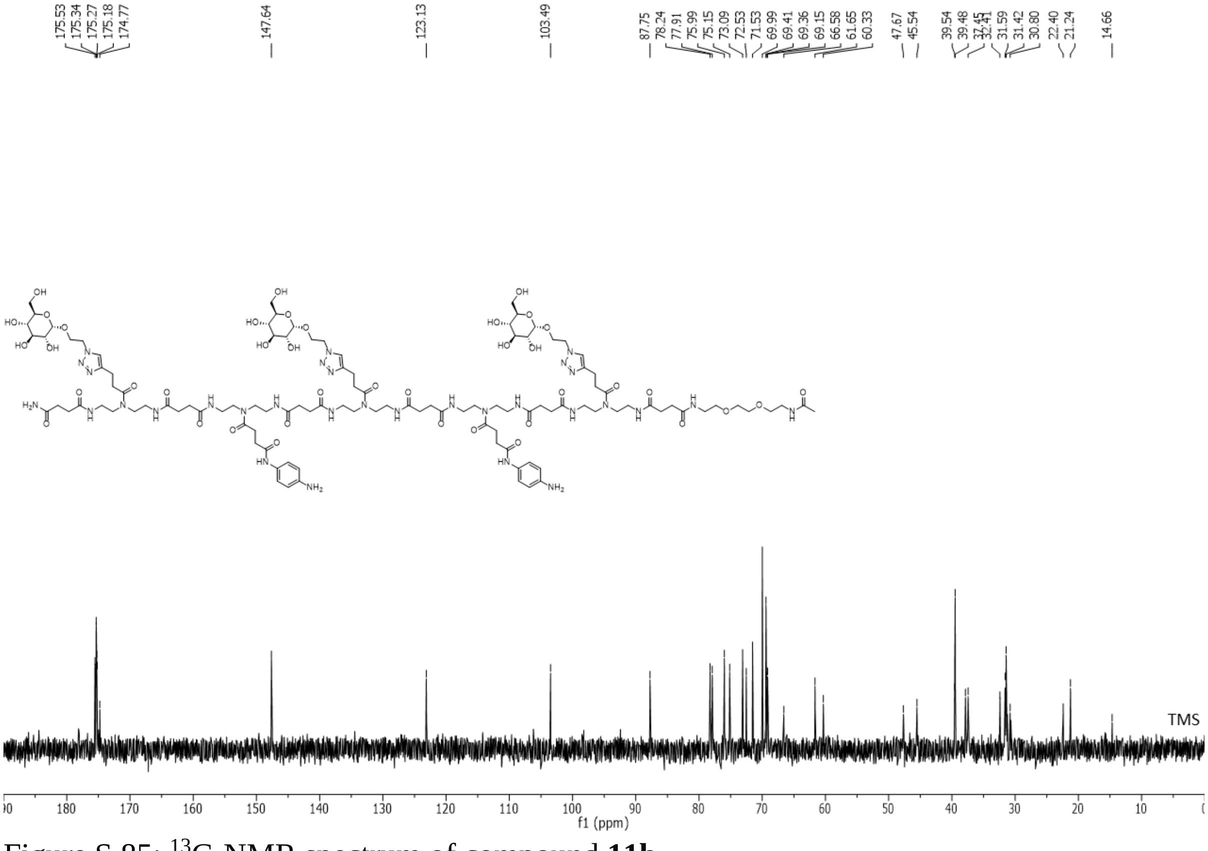

Figure S 85: ^{13}C-NMR spectrum of compound **11b.**

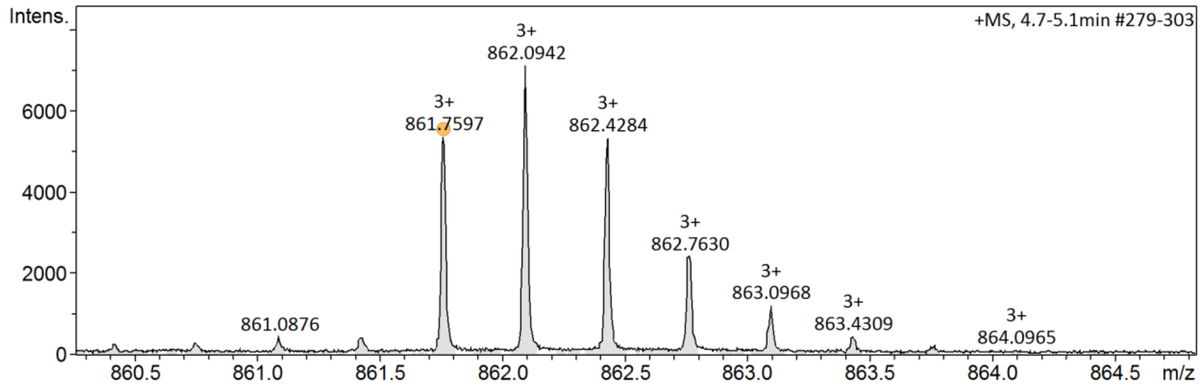

Figure S 86: HR-MS spectrum of compound **11b.**

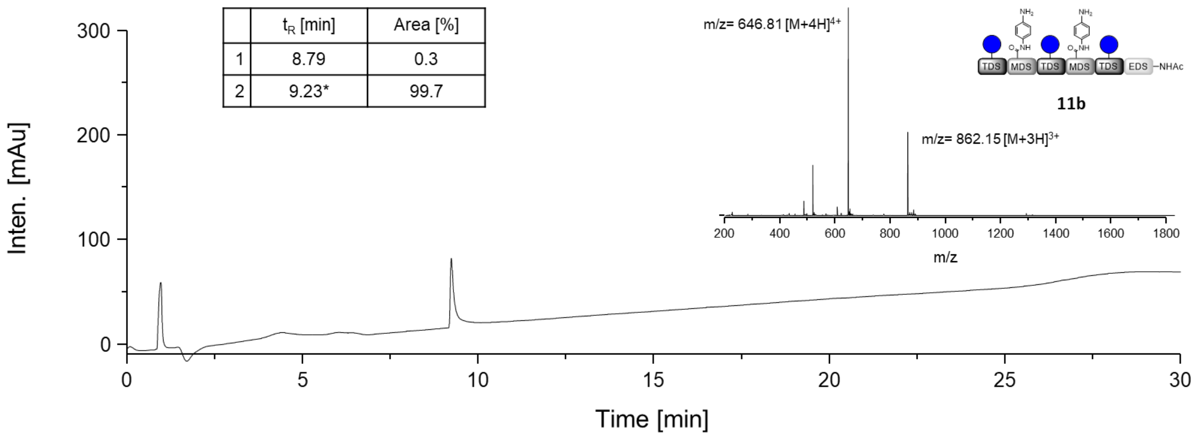

	t_R [min]	Area [%]
1	8.79	0.3
2	9.23*	99.7

Figure S 87: RP-HPLC chromatogram and ESI$^+$-MS spectrum of compound **11b.** Retention time t_R [min] and area [%] of the peaks are given. ESI-MS spectrum of the main peak (*) is shown.

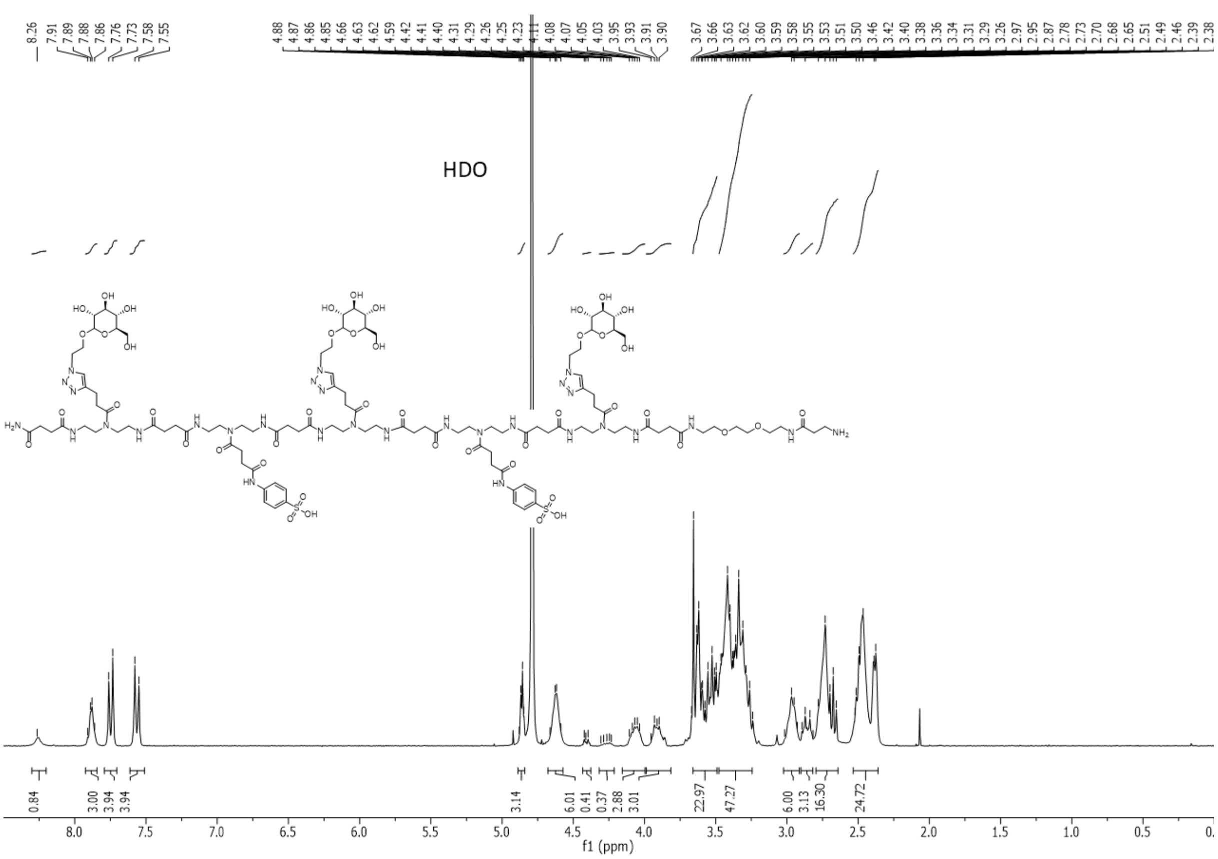

Figure S 88: ^{1}H-NMR spectrum of compound **12a.**

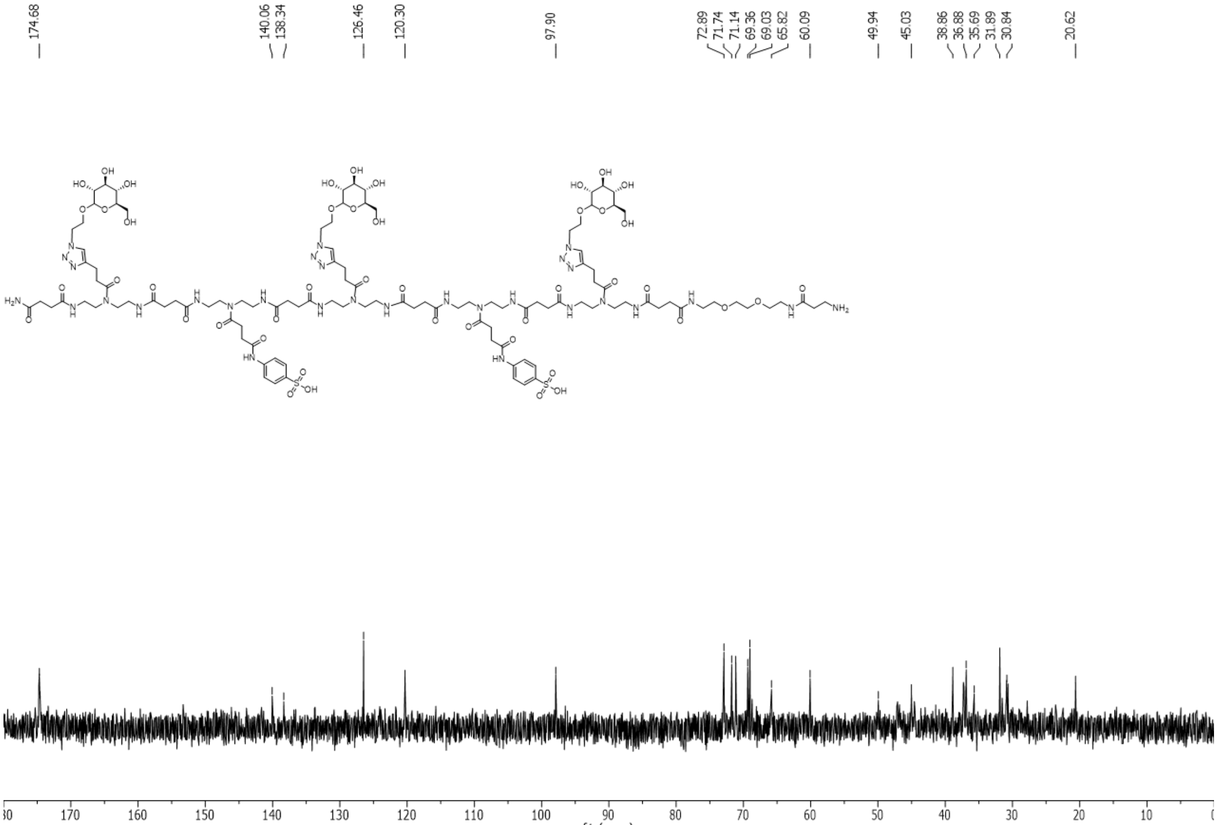

Figure S 89: ^{13}C-NMR spectrum of compound **12a.**

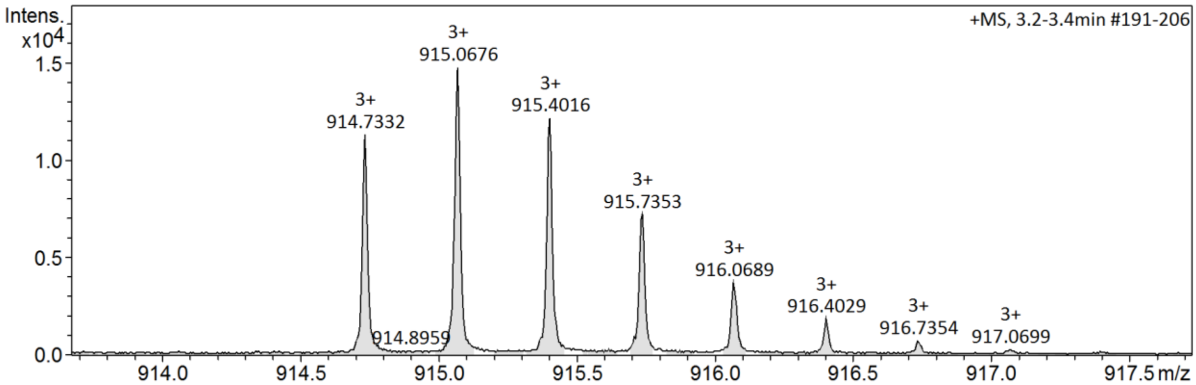

Figure S 90: HR-MS spectrum of compound **12a.**

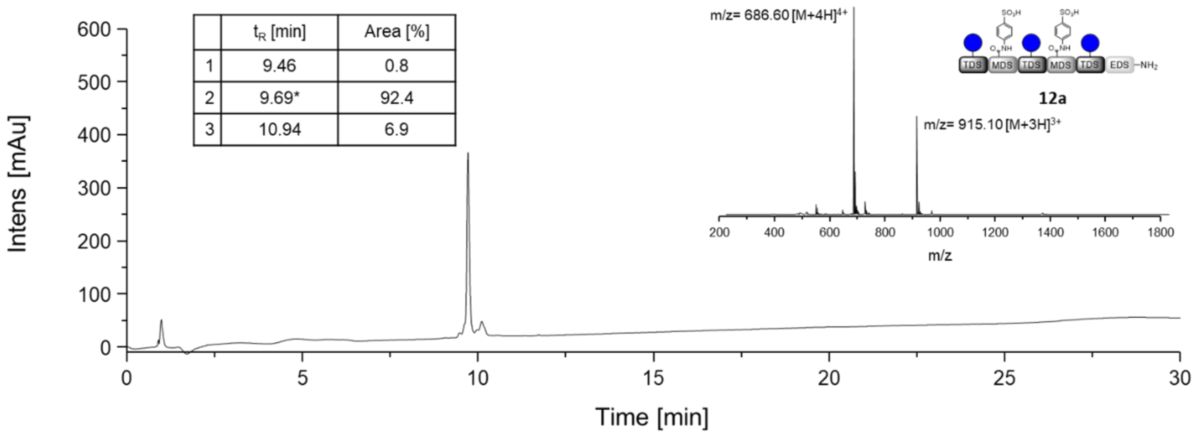

Figure S 91: RP-HPLC chromatogram and ESI^{+}-MS spectrum of compound **12a.** Retention time t_R [min] and area [%] of the peaks are given. ESI-MS spectrum of the main peak (*) is shown.

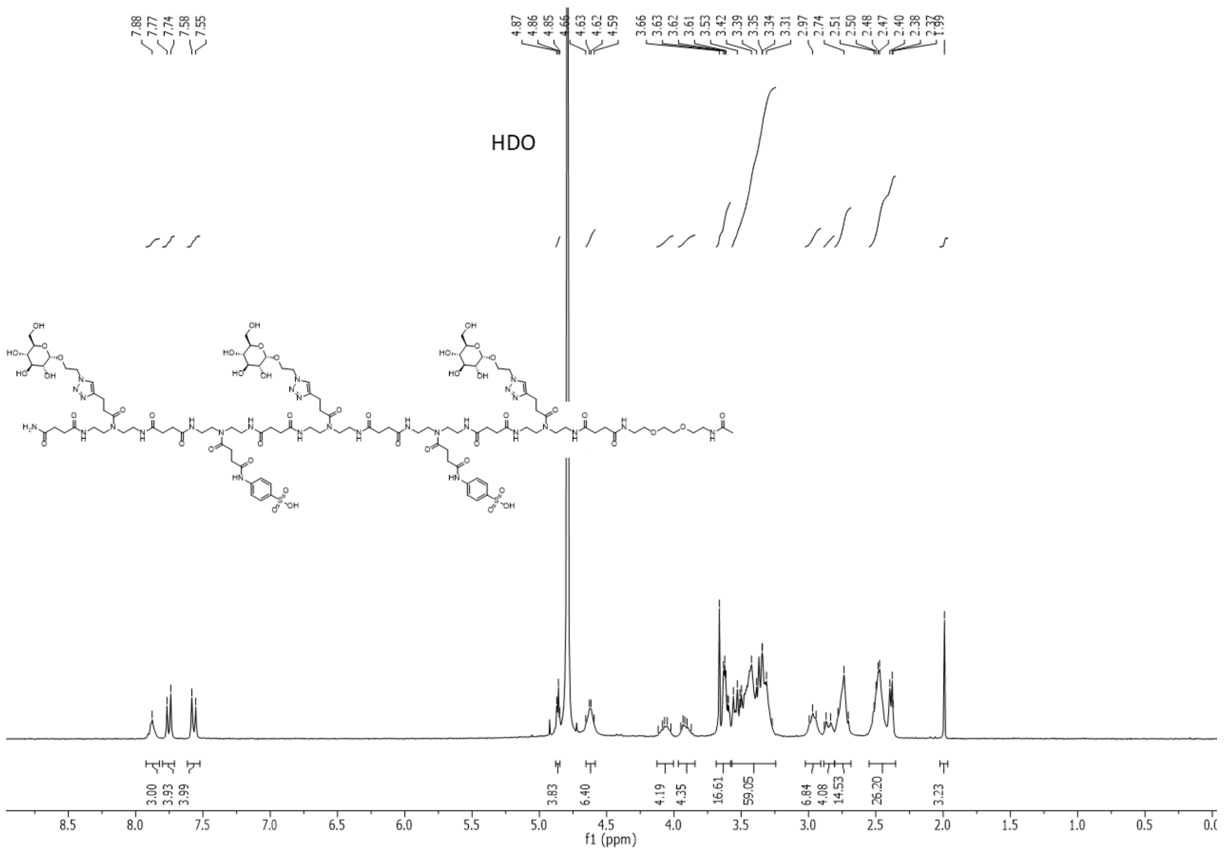

Figure S 92: ^{1}H-NMR spectrum of compound **12b.**

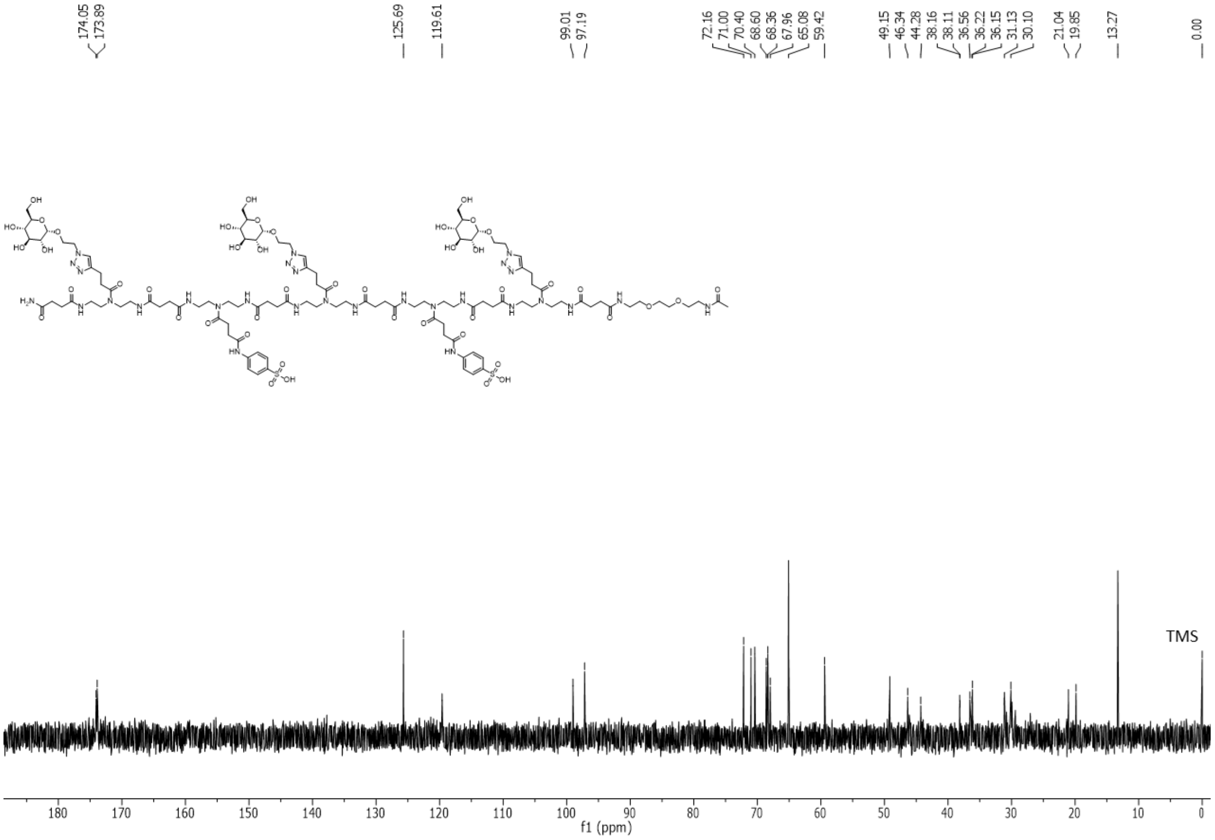

Figure S 93: ^{13}C-NMR spectrum of compound **12b**.

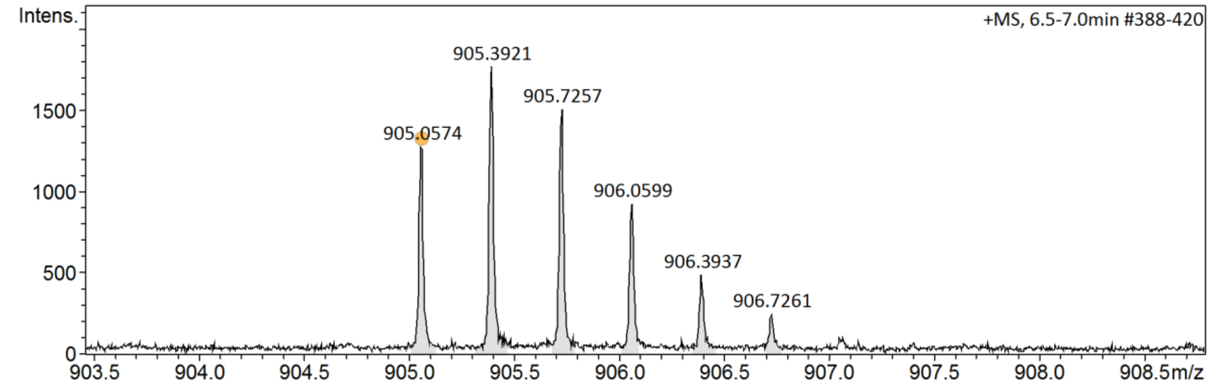

Figure S 94: HR-MS spectrum of compound **12b.**

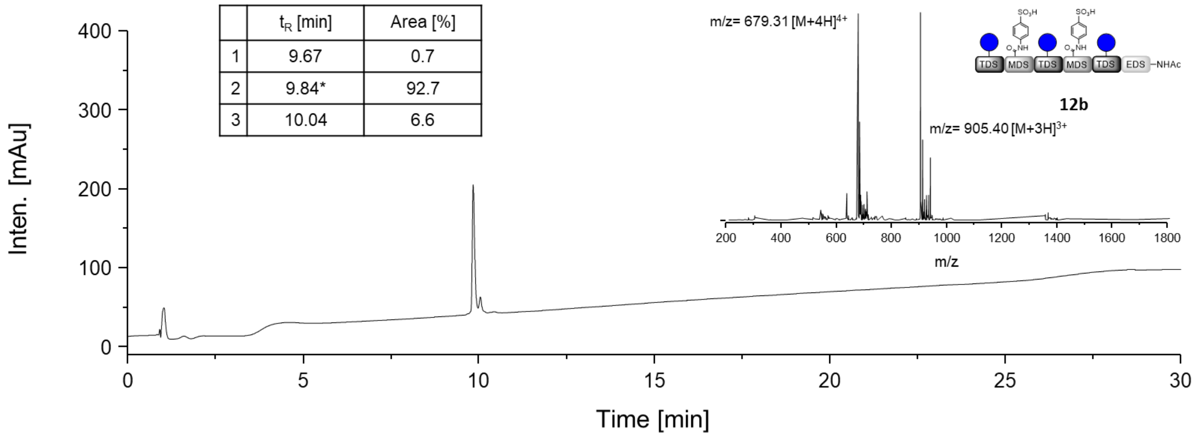

	t_R [min]	Area [%]
1	9.67	0.7
2	9.84*	92.7
3	10.04	6.6

Figure S 95: RP-HPLC chromatogram and ESI$^+$-MS spectrum of compound **12b.** Retention time t_R [min] and area [%] of the peaks are given. ESI-MS spectrum of the main peak (*) is shown.

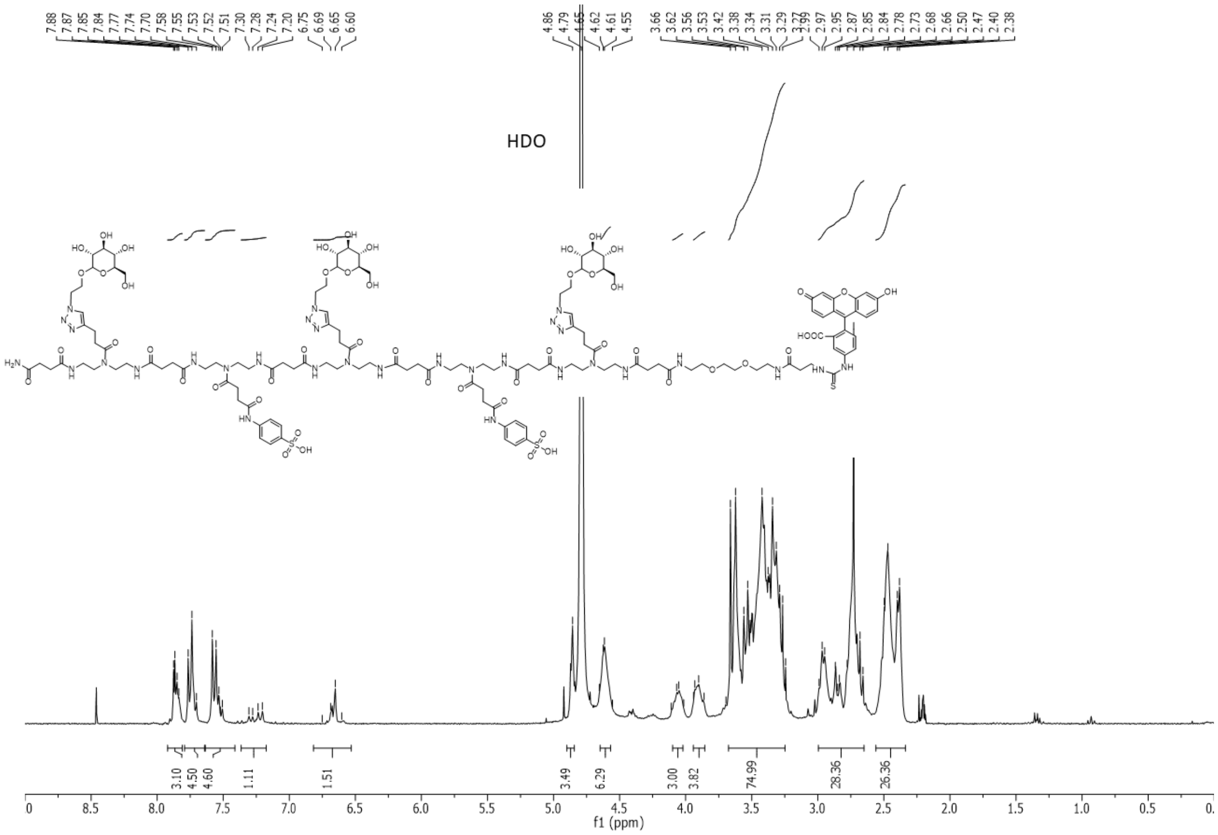

Figure S 96: ^{1}H-NMR spectrum of compound **12c**.

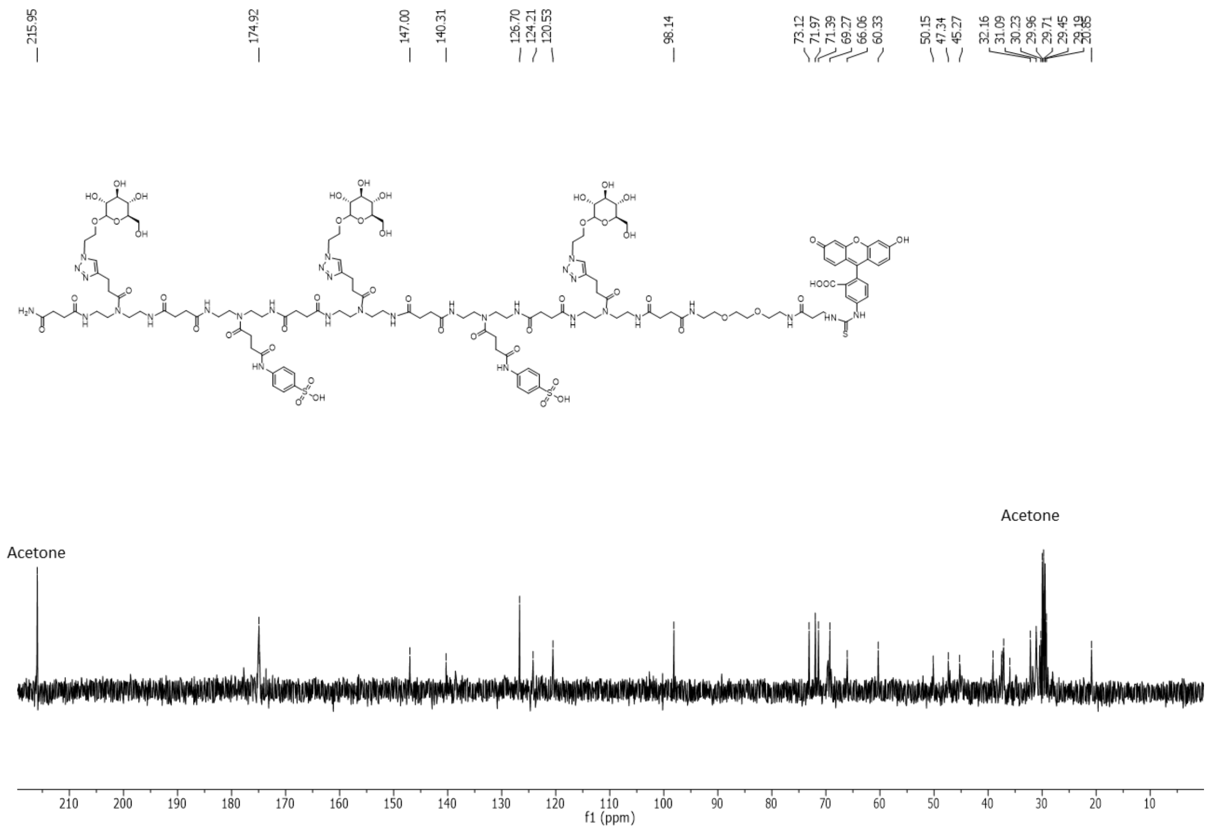

Figure S 97: ^{13}C-NMR spectrum of compound **12c.**

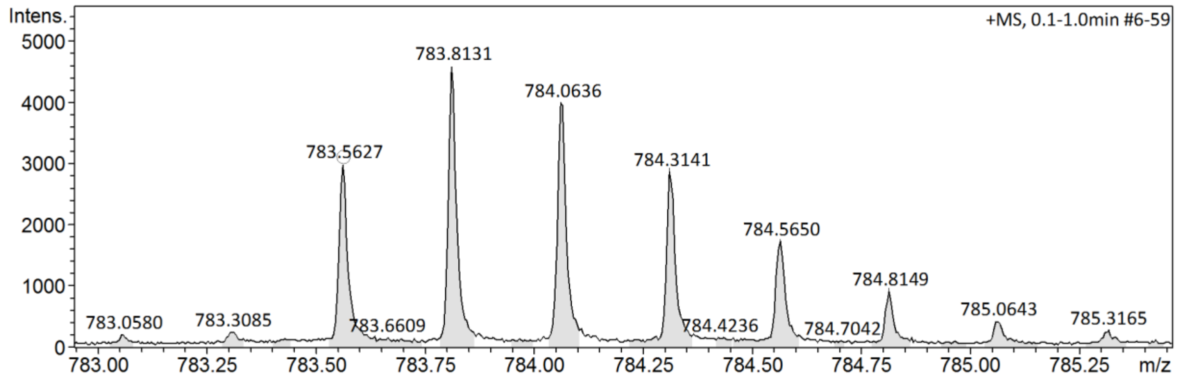

Figure S 98: HR-MS spectrum of compound **12c.**

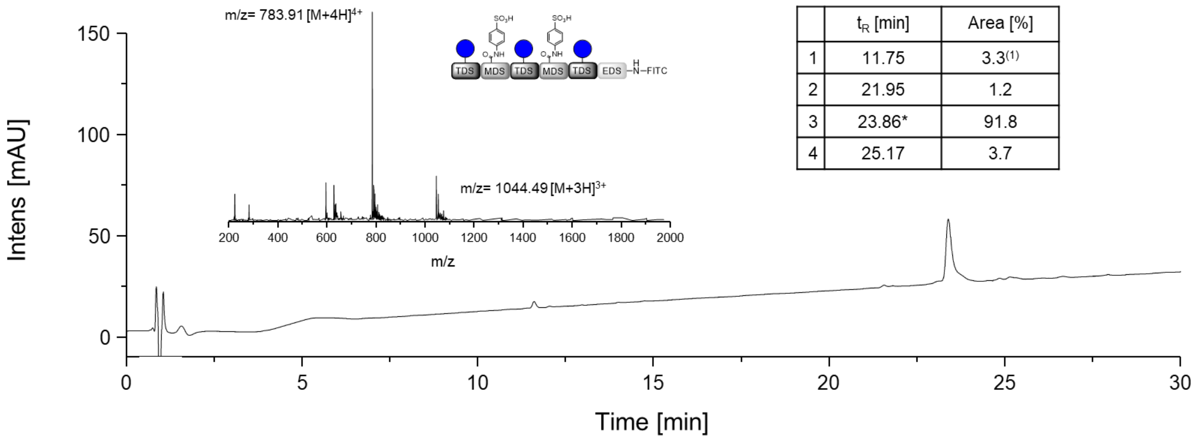

	t_R [min]	Area [%]
1	11.75	3.3(1)
2	21.95	1.2
3	23.86*	91.8
4	25.17	3.7

Figure S 99: RP-HPLC chromatogram and ESI$^+$-MS spectrum of compound **12c.** Retention time t_R [min] and area [%] of the peaks are given. ESI-MS spectrum of the main peak (*) is shown. (1) Unconjugated starting material.

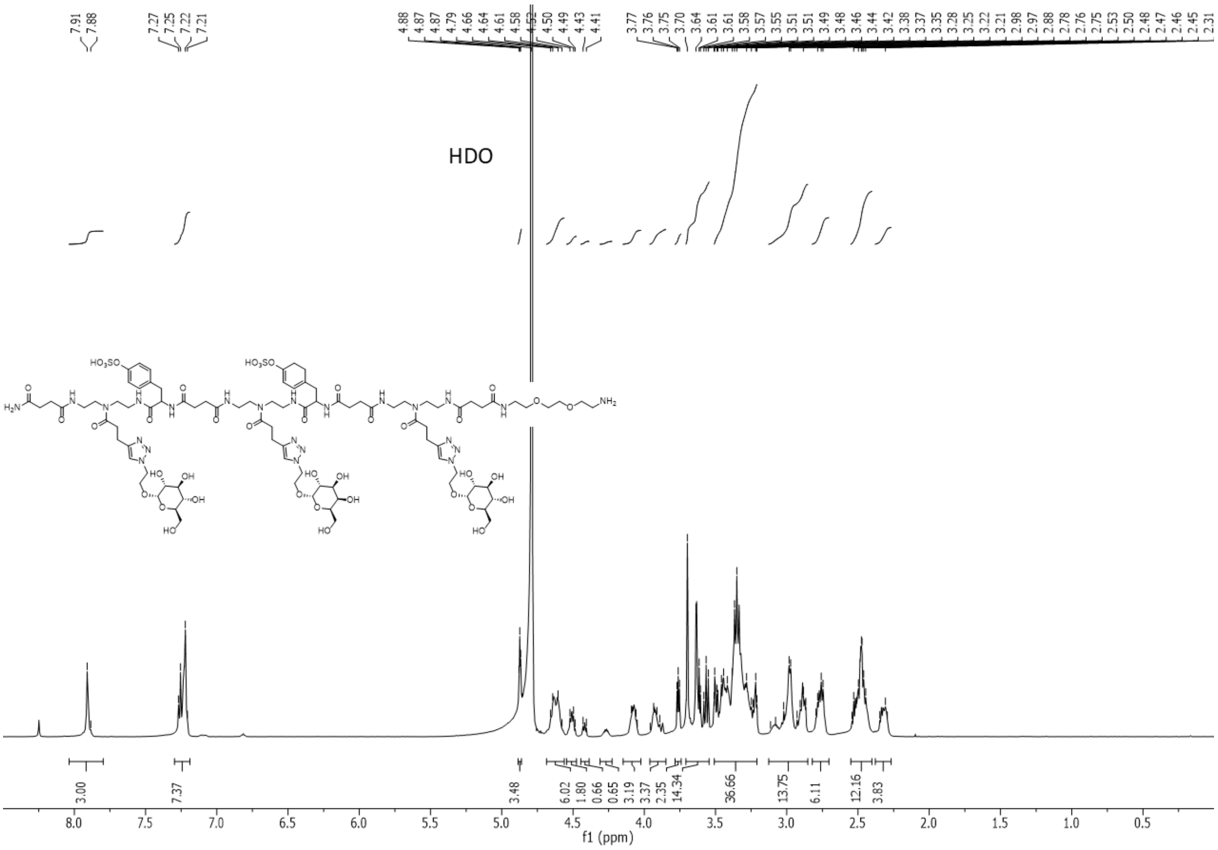

Figure S 100: ^{1}H-NMR spectrum of compound **13a**.

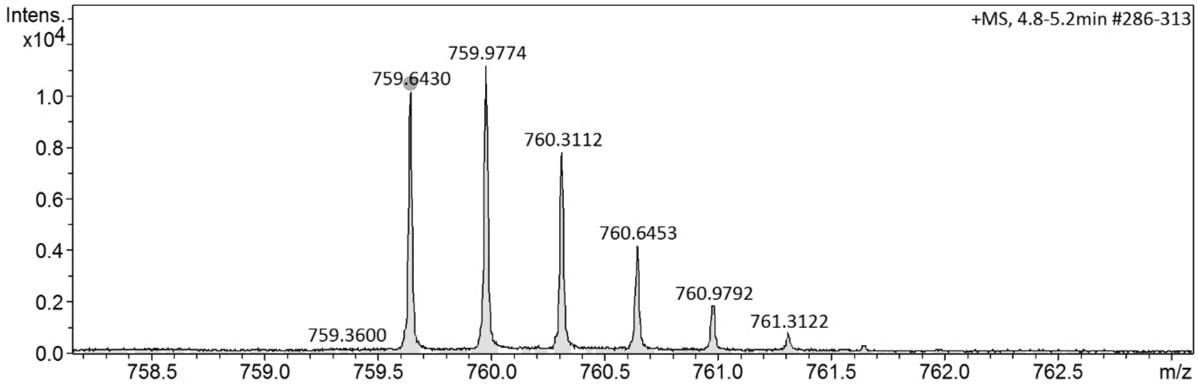

Figure S 101: HR-MS spectrum of compound **13a.**

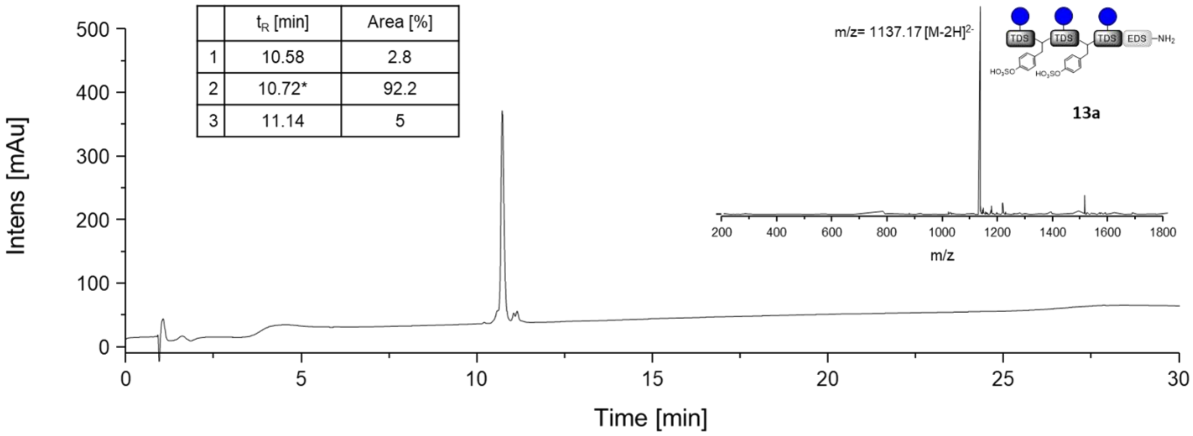

	t_R [min]	Area [%]
1	10.58	2.8
2	10.72*	92.2
3	11.14	5

Figure S 102: RP-HPLC chromatogram and ESI$^-$-MS spectrum of compound **13a**. Retention time t_R [min] and area [%] of the peaks are given. ESI-MS spectrum of the main peak (*) is shown.

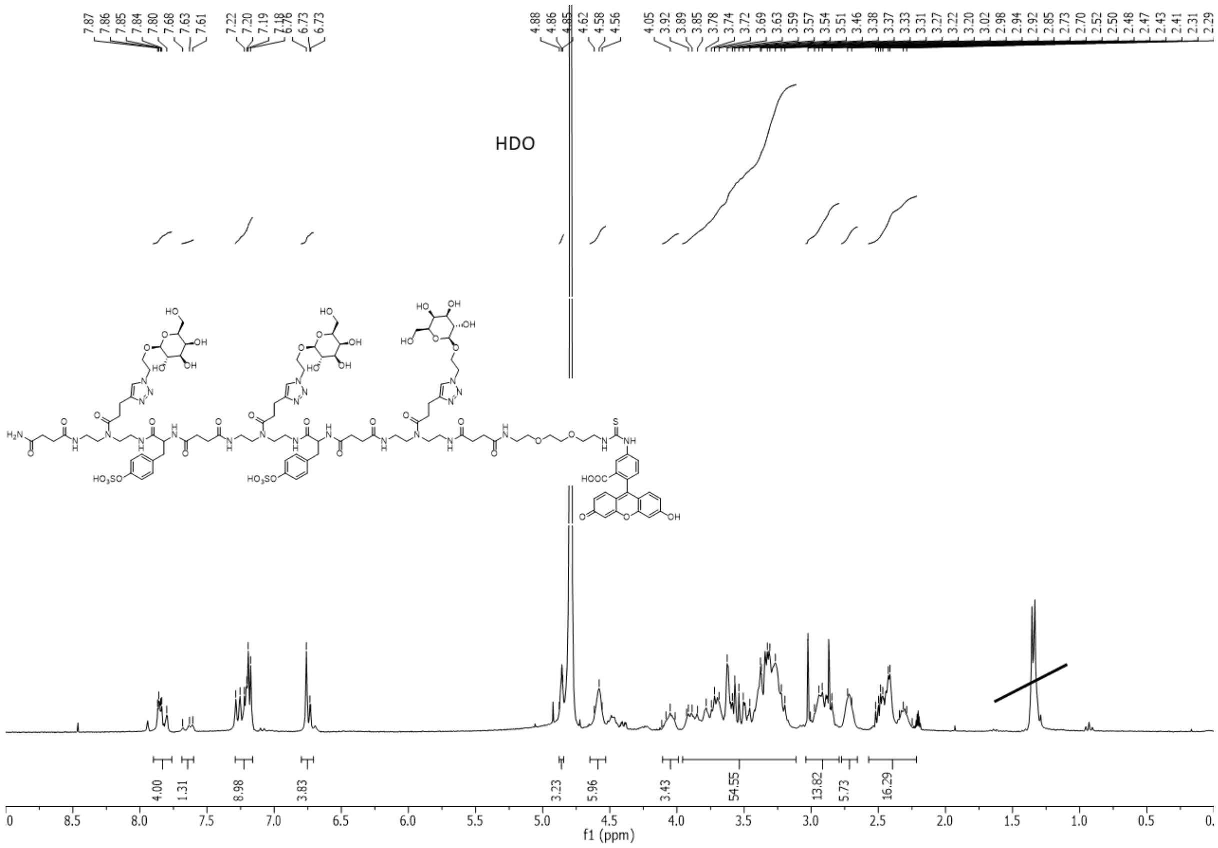

Figure S 103: ^{1}H-NMR spectrum of compound **13c.**

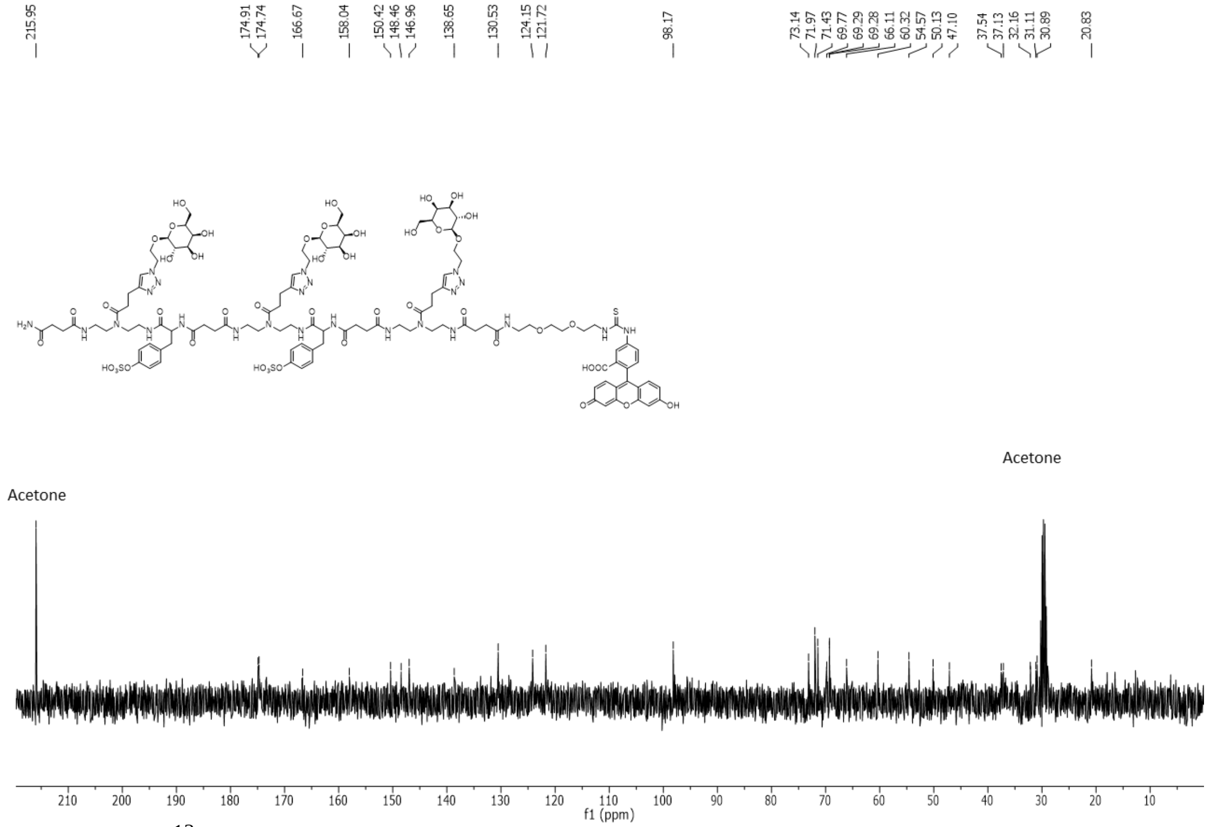

Figure S 104: ^{13}C-NMR spectrum of compound **13c**.

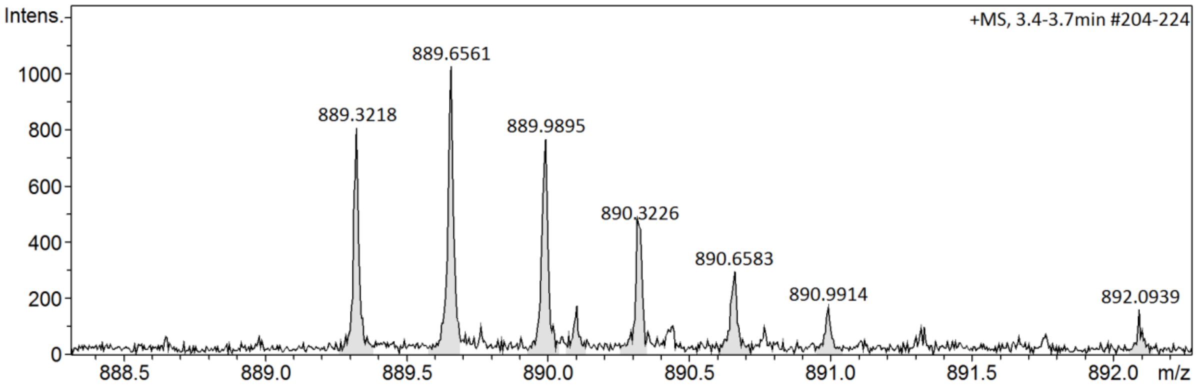

Figure S 105: HR-MS spectrum of compound **13c.**

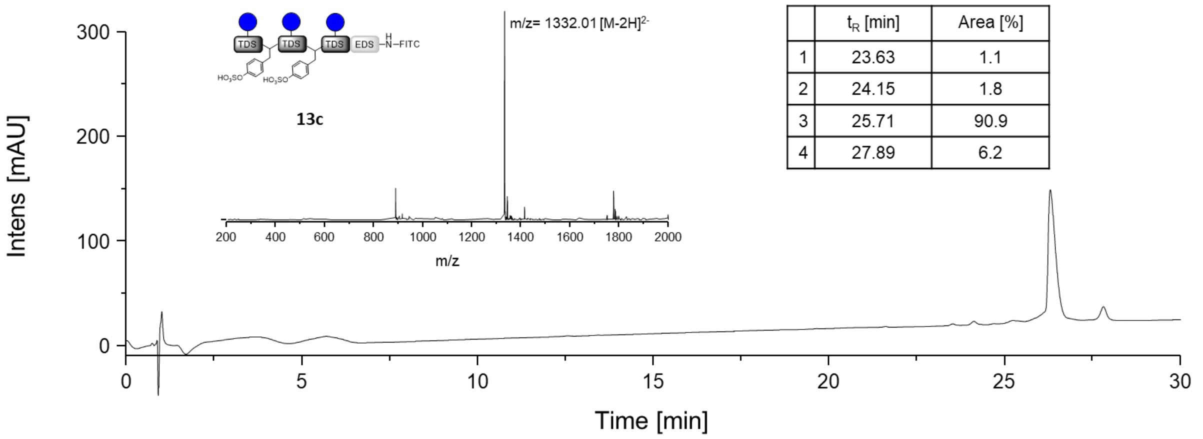

	t_R [min]	Area [%]
1	23.63	1.1
2	24.15	1.8
3	25.71	90.9
4	27.89	6.2

Figure S 106: RP-HPLC chromatogram and ESI$^+$-MS spectrum of compound **13c.** Retention time t_R [min] and area [%] of the peaks are given. ESI-MS spectrum of the main peak (*) is shown.

Figure S 107: ^{1}H-NMR spectrum of compound **14.**

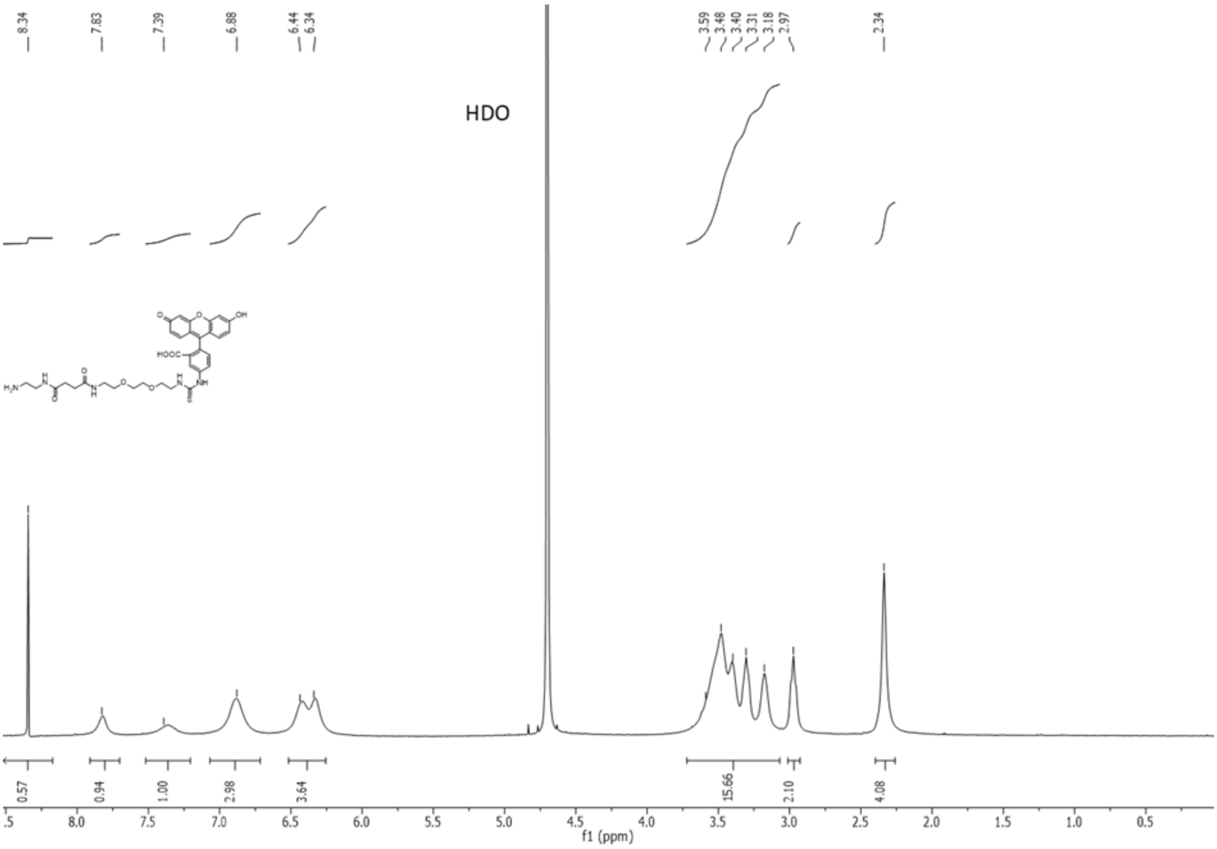

Figure S 107: ^{1}H-NMR spectrum of compound **14.**

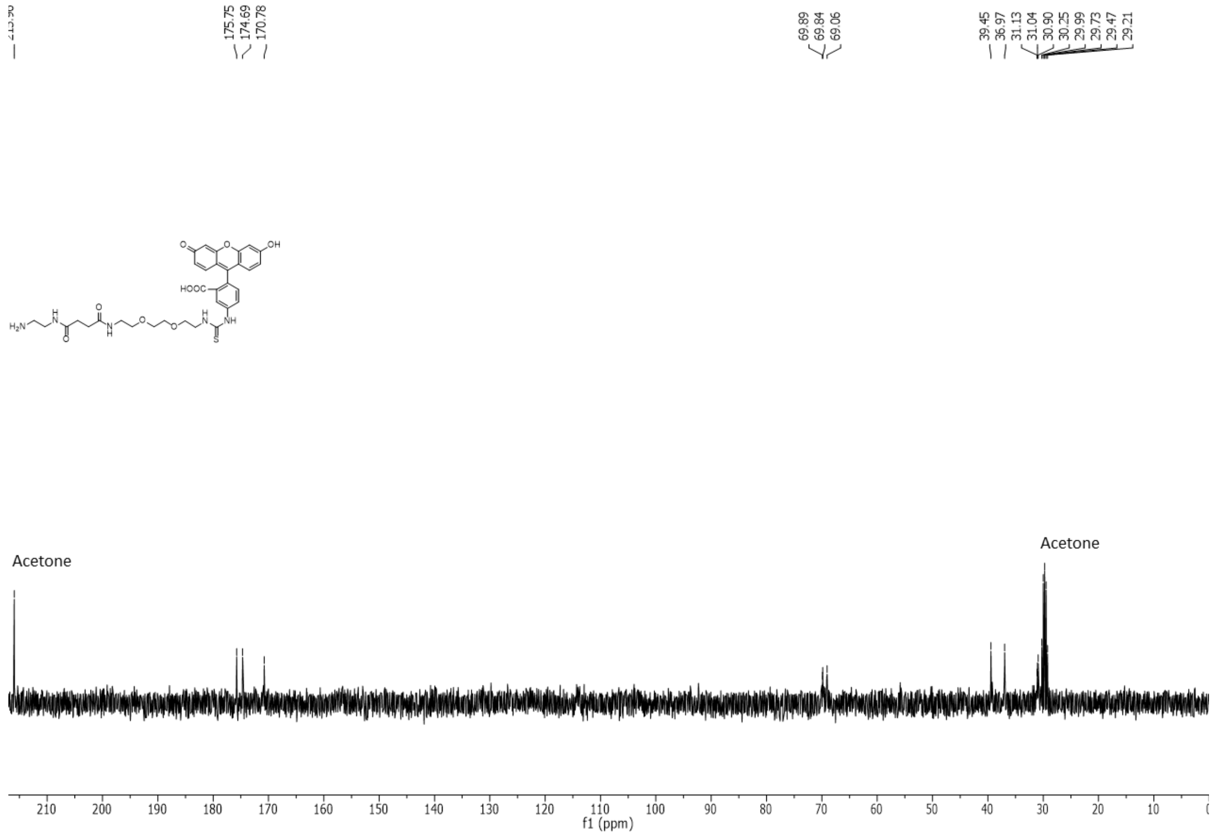

Figure S 108: ^{13}C-NMR spectrum of compound **14.**

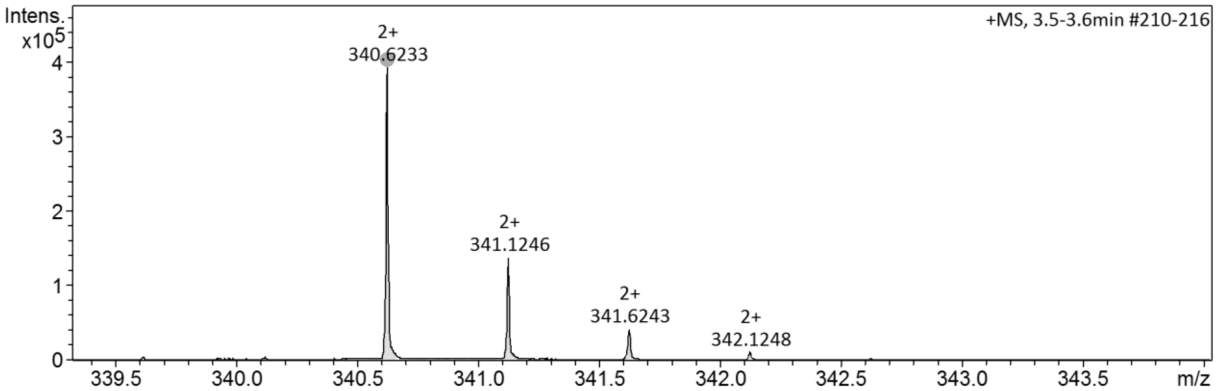

Figure S 109: HR-MS spectrum of compound **14.**

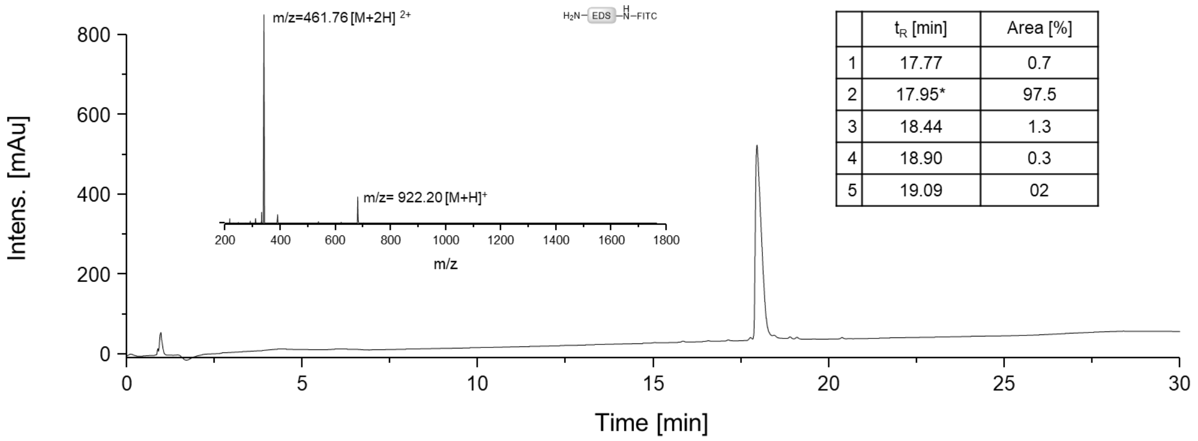

	t_R [min]	Area [%]
1	17.77	0.7
2	17.95*	97.5
3	18.44	1.3
4	18.90	0.3
5	19.09	02

Figure S 110: RP-HPLC chromatogram and ESI$^+$-MS spectrum of compound **14.** Retention time t_R [min] and area [%] of the peaks are given. ESI-MS spectrum of the main peak (*) is shown.

Results Binding Studies

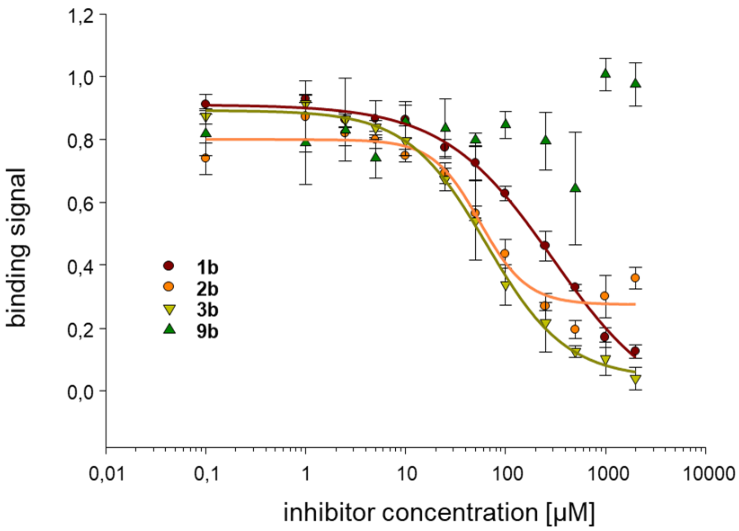

Figure S 111: Inhibition curves of the inhibition competition ELISA-type assays of Gal-1 with compounds **1b-3b** and **9b**.

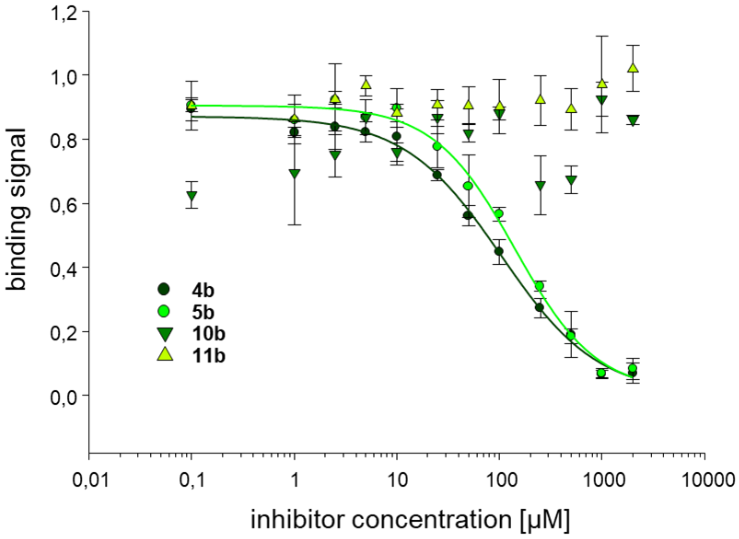

Figure S 112: Inhibition curves of the inhibition competition ELISA-type assays of Gal-1 with compounds **4b-5b** and **10b-11b**.

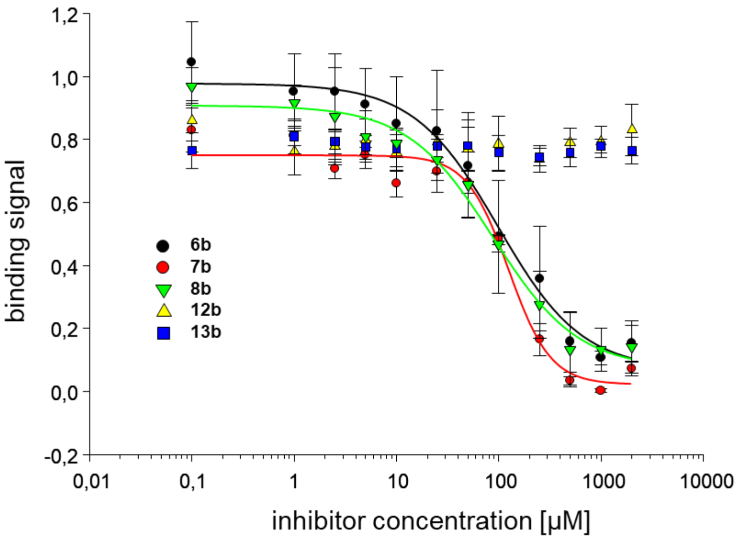

Figure S 113: Inhibition curves of the inhibition competition ELISA-type assays of Gal-1 with compounds **6b-8b** and **12b-13b**.

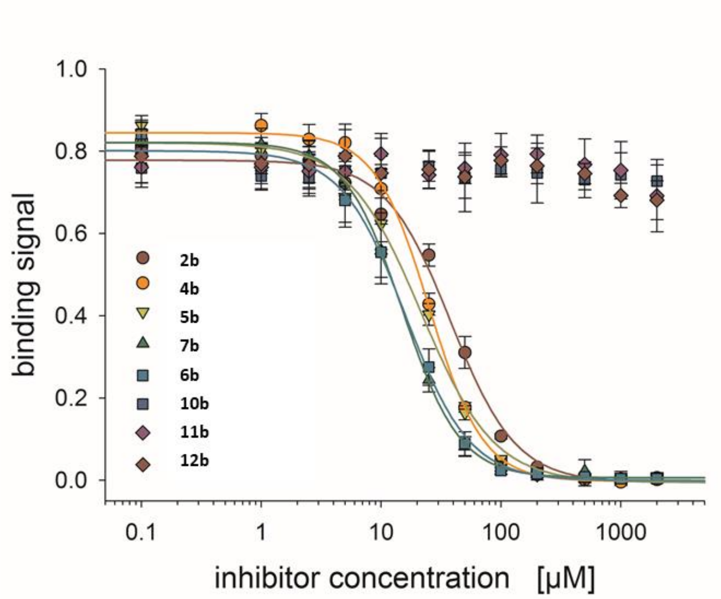

Figure S 114: Inhibition curves of the inhibition competition ELISA-type assays of Gal-3 with compounds **2b,4b-7b** and **10b-12b**.

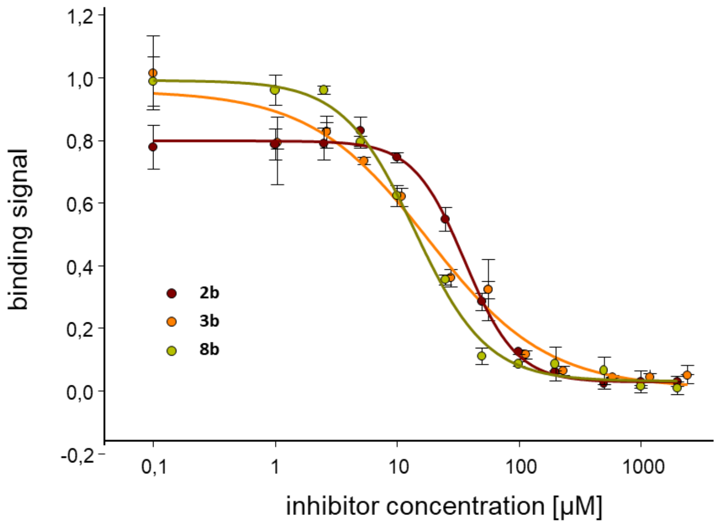

Figure S 115: Inhibition curves of the inhibition competition ELISA-type assays of Gal-3 with compounds **2b**, **3b** and **8b**.

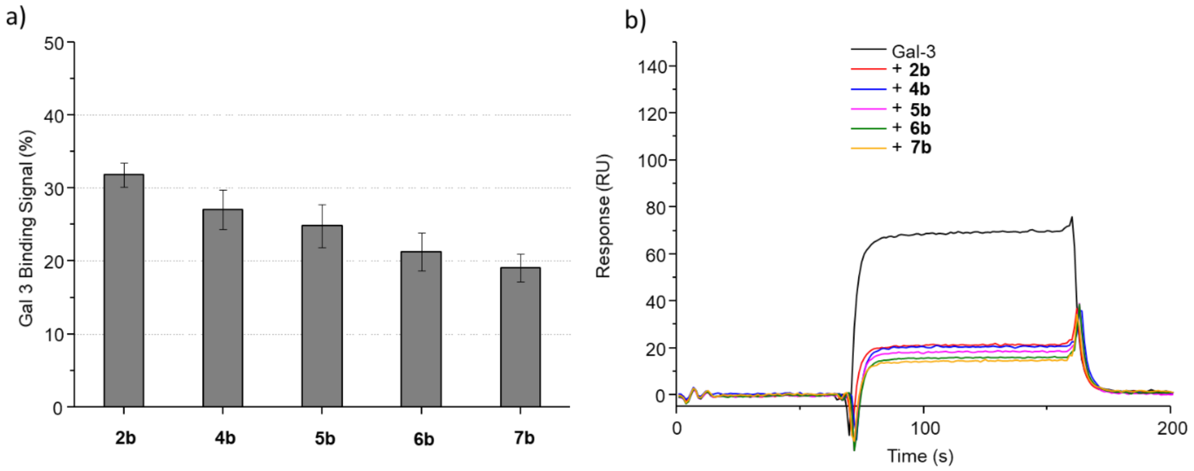

Figure S 116: Results from the SPR inhibition studies of Gal-3 and samples **2b**,**4b**-**7b**. Values are calculated regarding the signal of Gal-3 as 100 % binding signal.

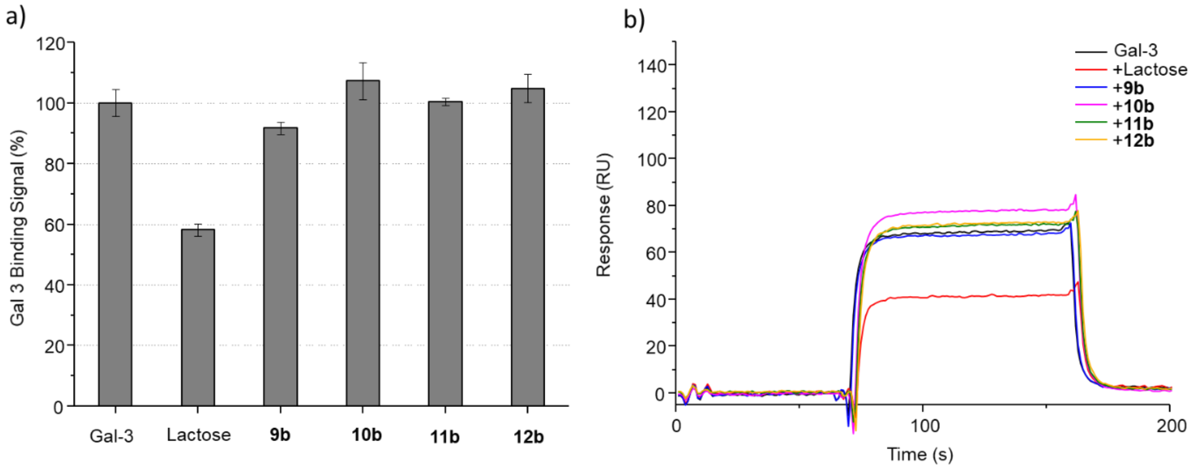

Figure S 117: Results from the SPR inhibition studies of Gal-3 with lactose as control and samples **9b-12b**. Values are calculated regarding the signal of Gal-3 as 100 % binding signal.

Results Cell Assays

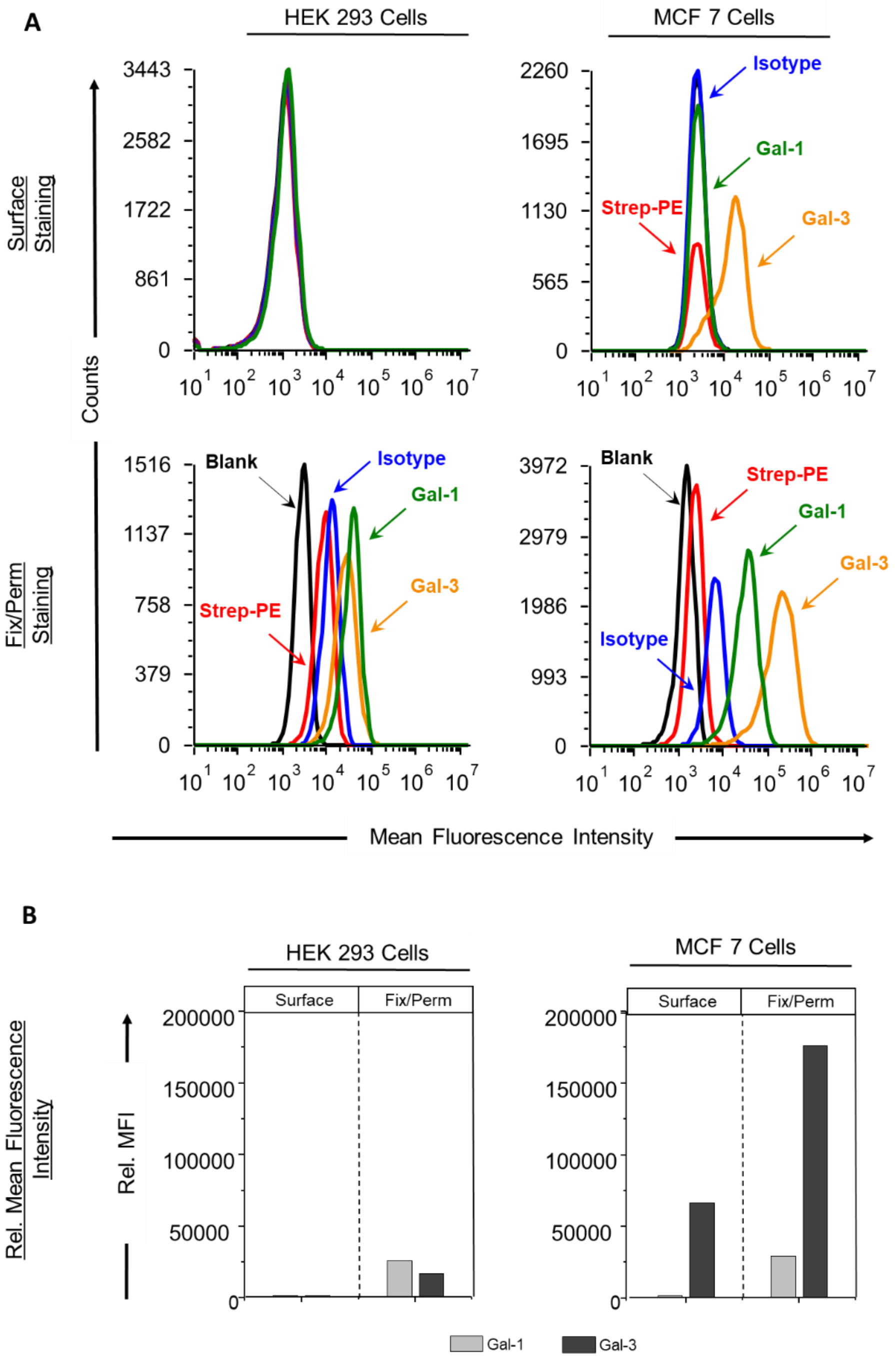

Figure S 118: Flow cytometry of the antibody staining of HEK 293 and MCF 7 cells. A. Histogram of the mean fluorescence intensity (MFI) of the surface staining and intracellular staining after fixation and permeabilization (Fix/Perm.) using biotinylated anti-bodies and PE-conjugated streptavidin. Unstained (black), streptavidin-PE (red), isotype control (blue), anti-Gal-3 (yellow) and anti-Gal-1(green). B. Relative mean fluorescence of the Gal-3 staining on the surface and after fixation and permeabilization. Values are MFI of Gal-1 or Gal-3 subtracted by the MFI of the isotype control.

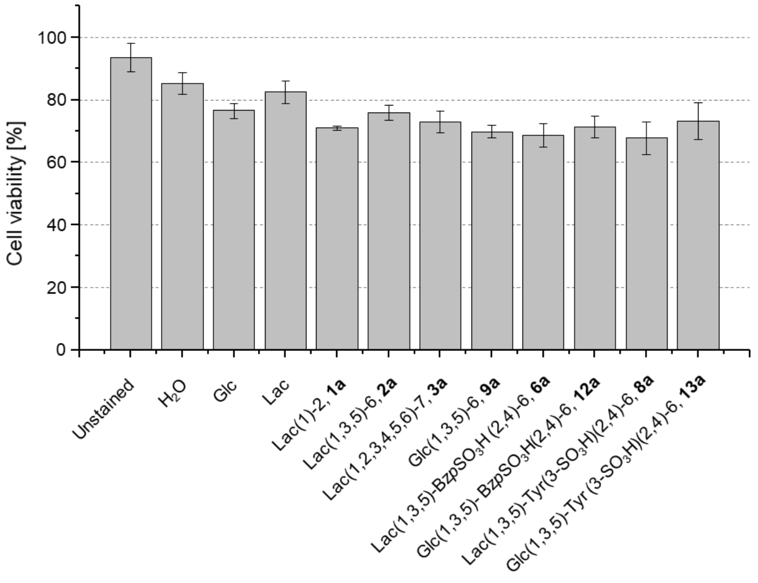

Figure S 119: Results of the MTT cell viability assay for the HEK 293 cell line. Measurements were performed two times in triplicates.

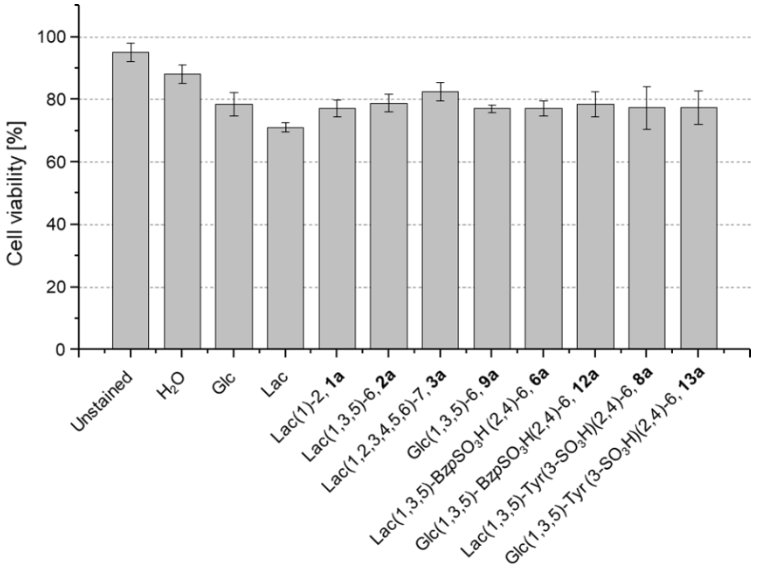

Figure S 120: Results of the MTT cell viability assay for MCF 7 cell line. Measurements were performed two times in triplicates.

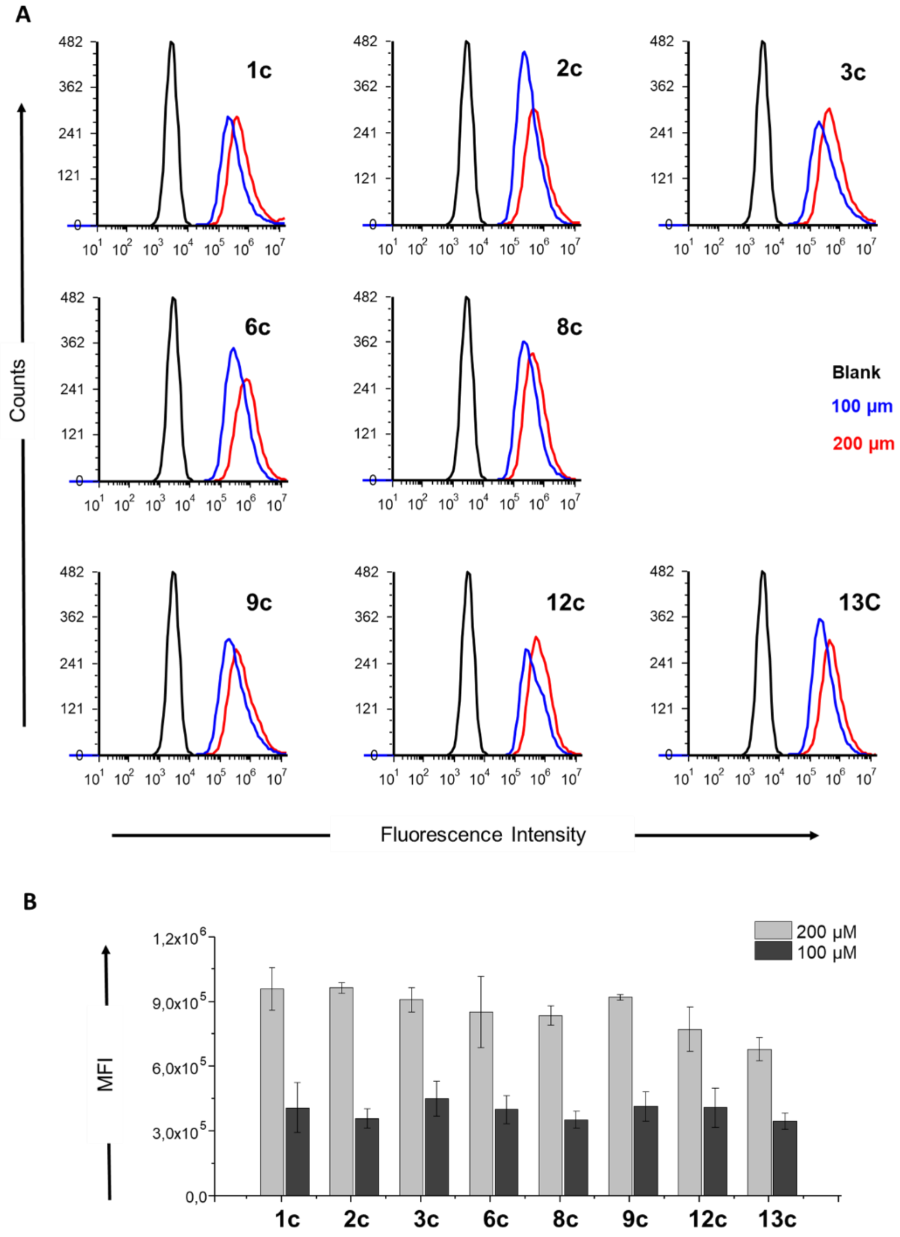

Figure S 121: Results of the uptake studies of the MCF 7 cells for glycomacromolecules **1-3c, 6c, 8c, 9c, 12c, 13c**. A. Histograms of the flow measurements showing the mean fluorescence intensity (MFI) of unstained cells and cells incubated with FITC-labeled glycomacromolecules at final concentrations of 100 and 200 µM. B. Comparison of the MFI-values for the different glycomacromolecules. Measurements were performed in duplicates.

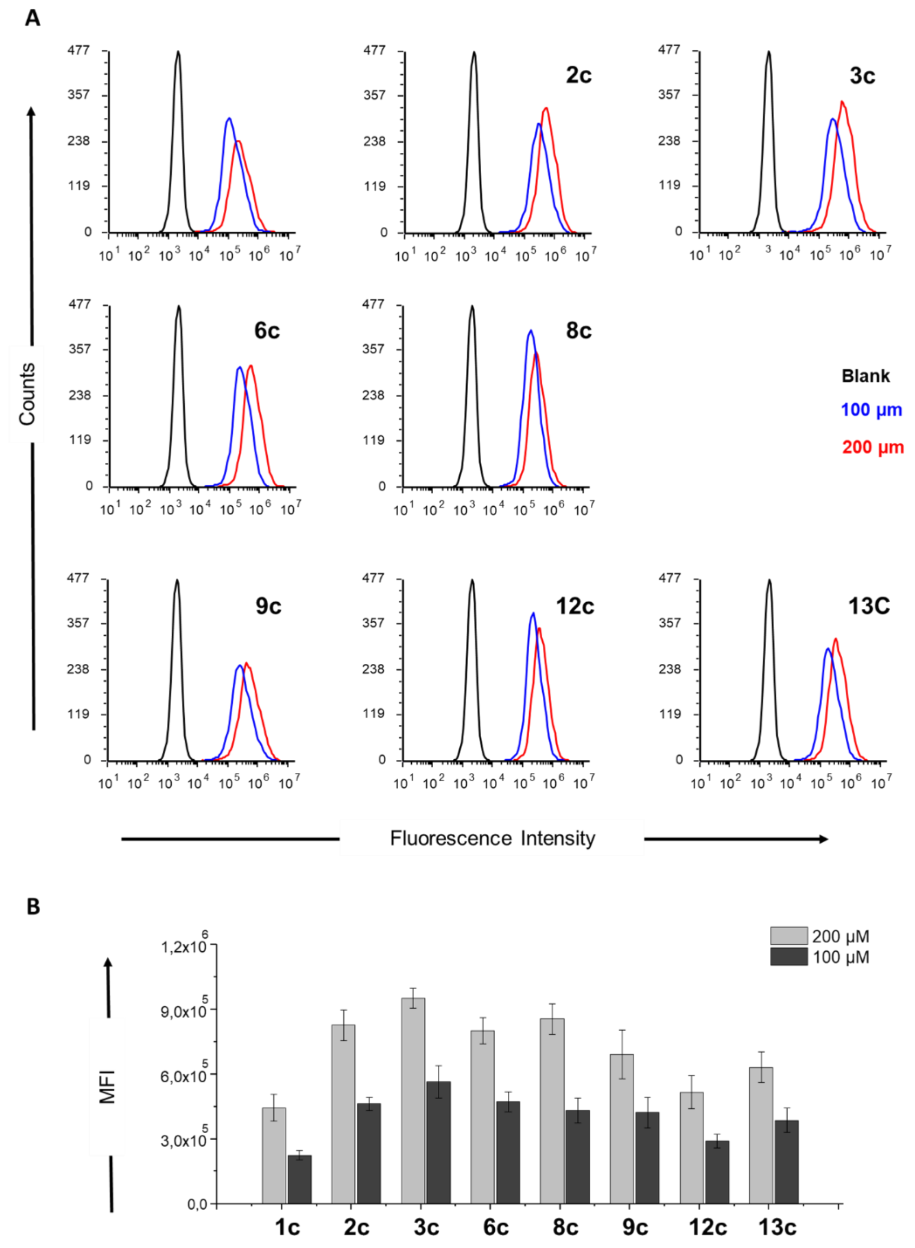

Figure S 122: Results of the uptake studies of HEK 293 cells for glycomacromolecules **1-3c, 6c, 8c, 9c, 12c, 13c**. A. Histograms of the flow measurements showing the mean fluorescence intensity (MFI) of unstained cells and cells incubated with FITC-labeled glycomacromolecules at a final concentration of 100 and 200 µM. B. Comparison of the MFI-values for the different glycomacromolecules. Measurements were performed in duplicates.

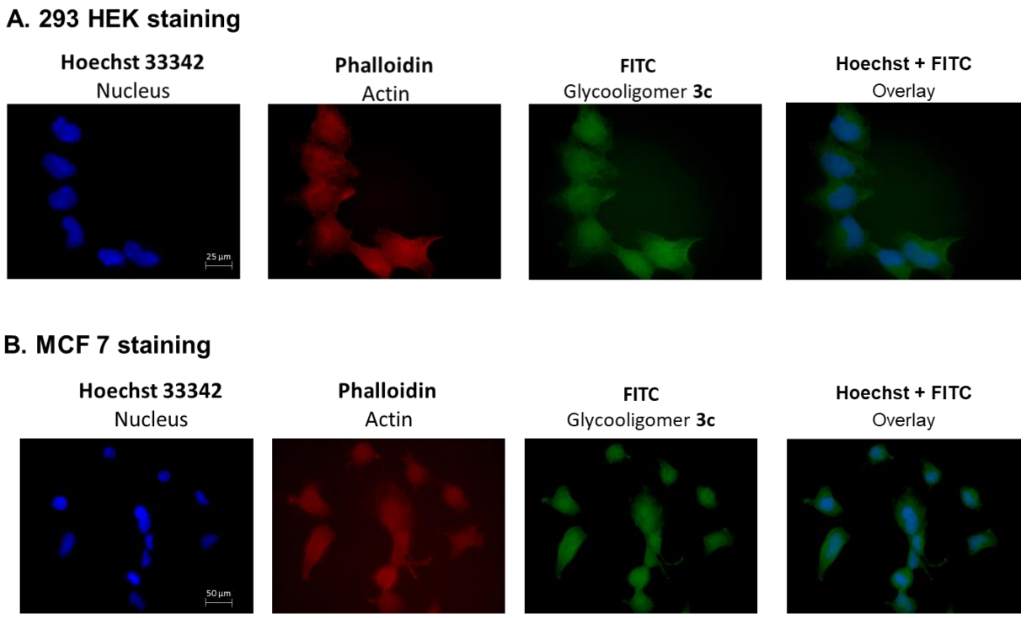

Figure S 123: Exemplary fluorescence microscopy image of HEK 293 (A) and MCF 7 (B) with Hoechst 33342 staining, Phalloidin staining and glycocoligomer **3c**.

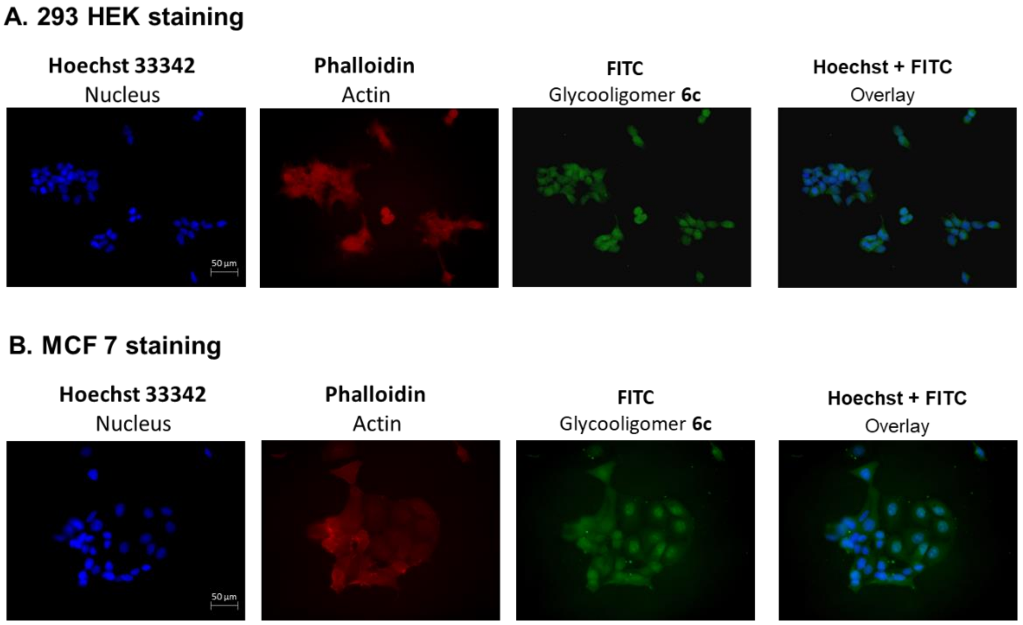

Figure S 124: Exemplary fluorescence microscopy image of HEK 293 (A) and MCF 7 (B) with Hoechst 33342 staining, Phalloidin staining and glycocoligomer **6c**.

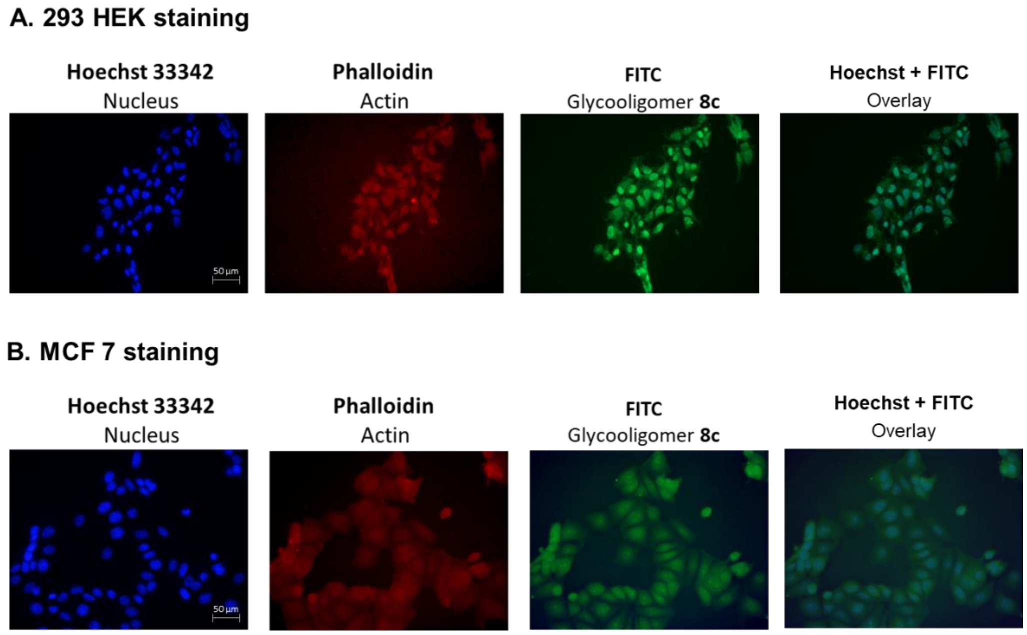

Figure S 125: Exemplary fluorescence microscopy image of HEK 293 (A) and MCF 7 (B) with Hoechst 33342 staining, Phalloidin staining and glycocoligomer **8c**.

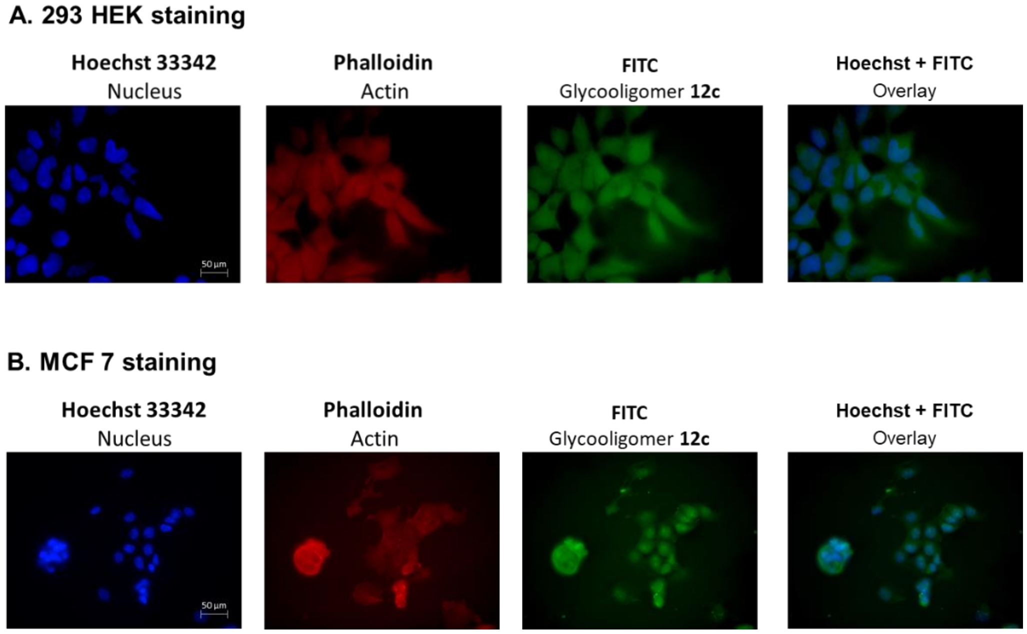

Figure S 126: Exemplary fluorescence microscopy image of HEK 293 (A) and MCF 7 (B) with Hoechst 33342 staining, Phalloidin staining and glycocoligomer **12c**.

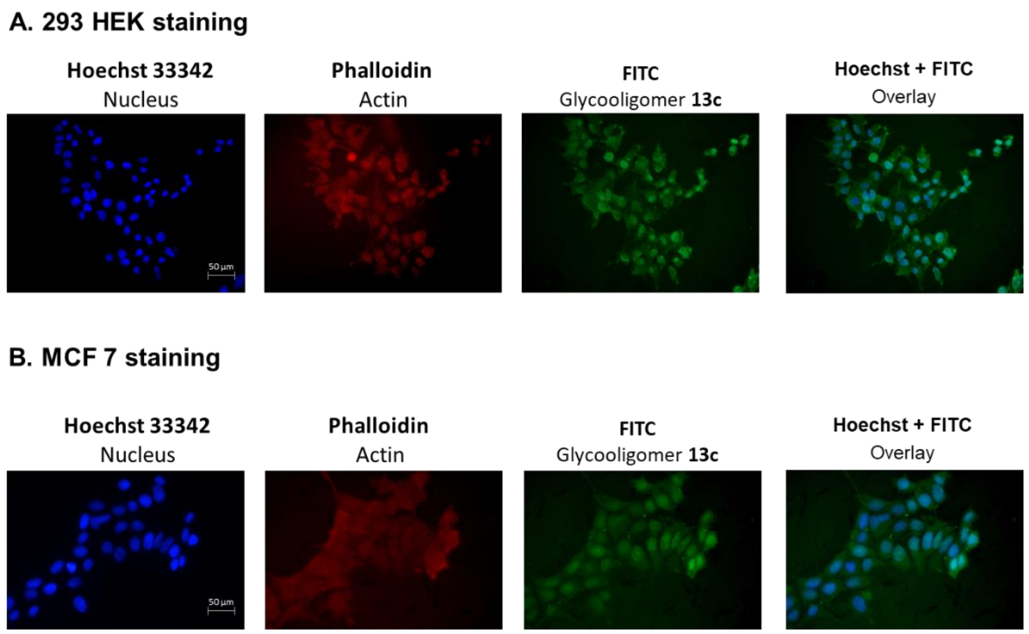

Figure S 127: Exemplary fluorescence microscopy image of HEK 293 (A) and MCF 7 (B) with Hoechst 33342 staining, Phalloidin staining and glycocoligomer **13c**.

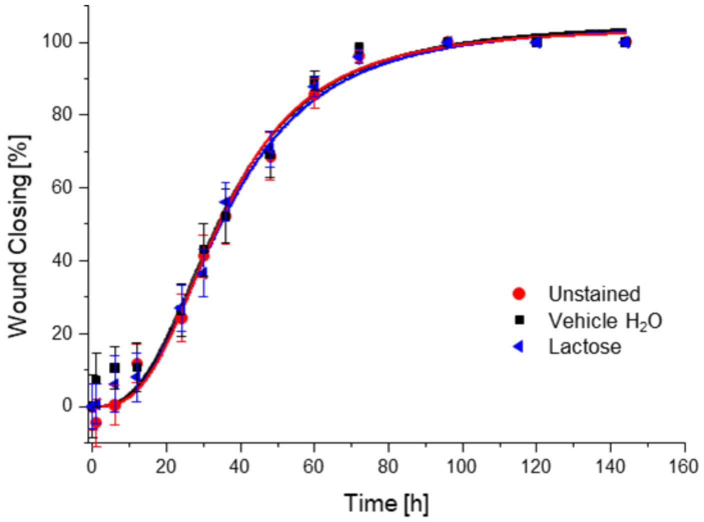

Figure S 128: Results of the migration of assays of MCF7: Untreated (red), vehicle control with H_2O (black) and treated with lactose (blue).

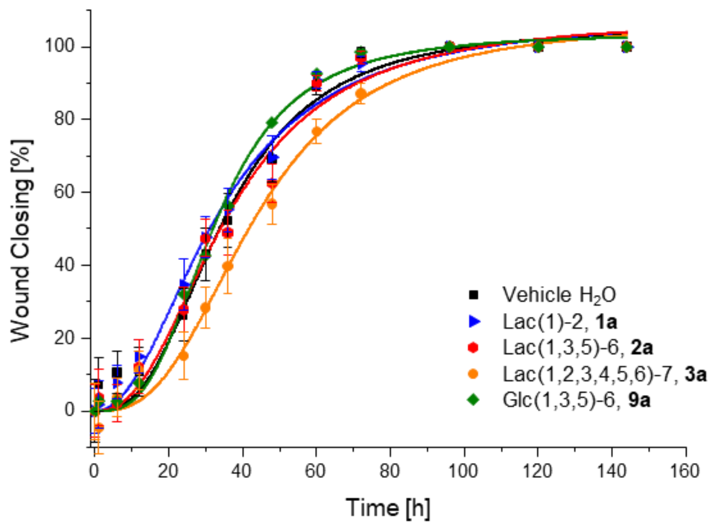

Figure S 129: Results of the migration of assays of MCF7 treated with the vehicle control H_2O (black) and compounds **1a** (blue), **2a** (red), **3a** (orange) and **9a** (green).

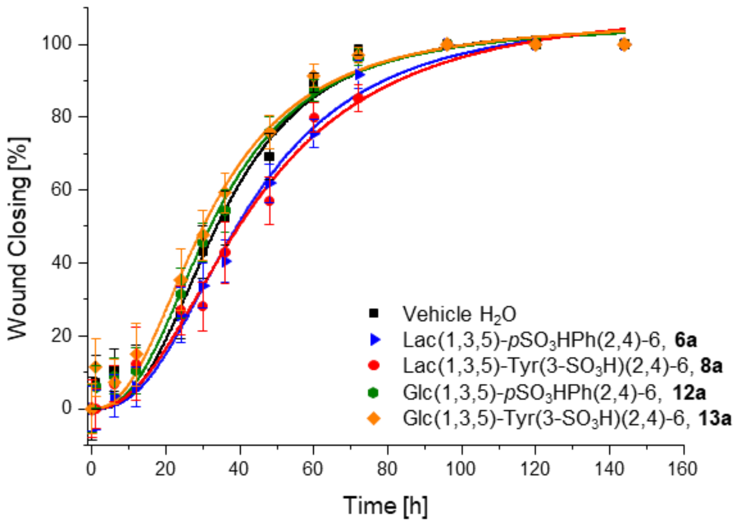

Figure S 130: Results of the migration of assays of MCF7 treated with the vehicle control H_2O (black) and compounds **6a** (blue), **8a** (red), **12a** (green) and **13a** (orange).

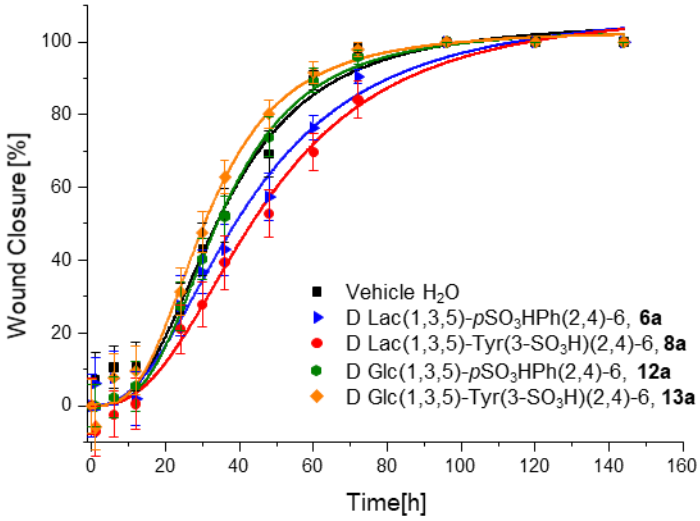

Figure S 131: Results of the migration of assays of MCF7 treated with the vehicle control H_2O (black) and compounds **6a** (blue), **8a** (red), **12a** (green) and **13a** (orange) in a dosing experiment giving extra doses after 12,24,36 and 48 h.

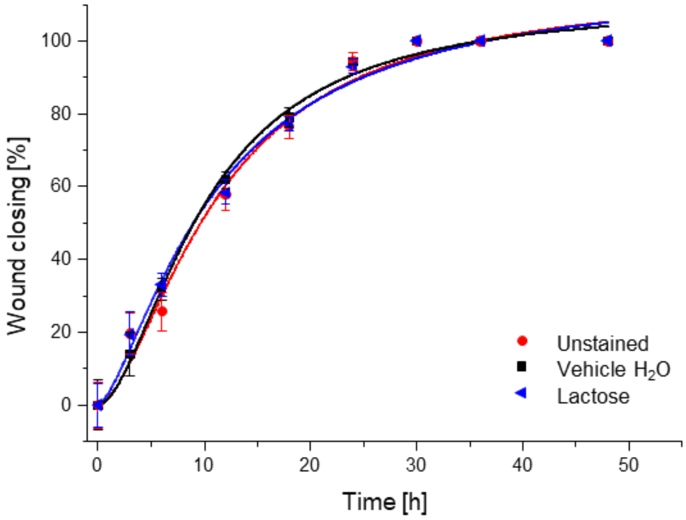

Figure S 132: Results of the migration of assays of HEK 293: Untreated (red), vehicle control with H_2O (black) and treated with lactose (blue).

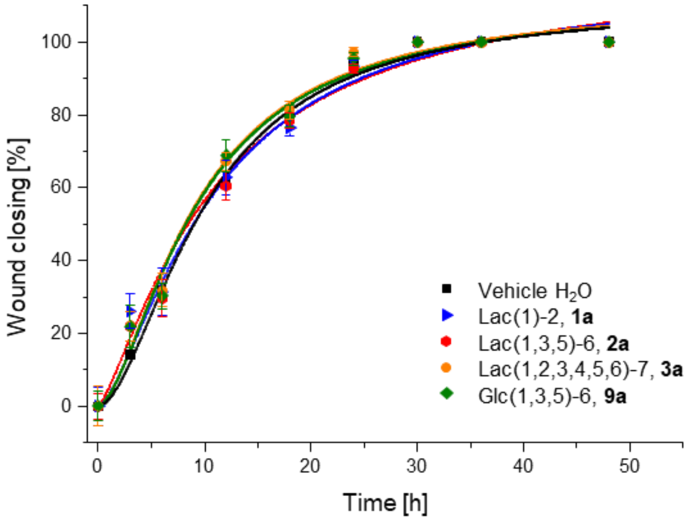

Figure S 133: Results of the migration of assays of HEK 293 treated with the vehicle control H_2O (black) and compounds **1a** (blue), **2a** (red), **3a** (orange) and **9a** (green).

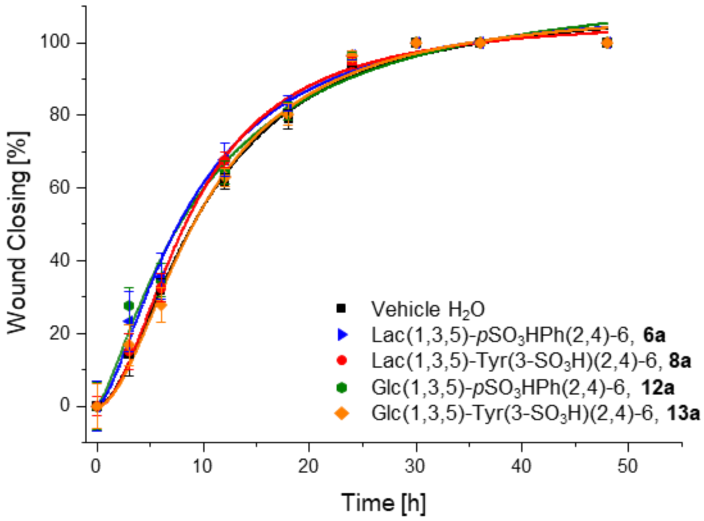

Figure S 134: Results of the migration of assays of HEK 293 treated with the vehicle control H_2O (black) and compounds **6a** (blue), **8a** (red), **12a** (green) and **13a** (orange).

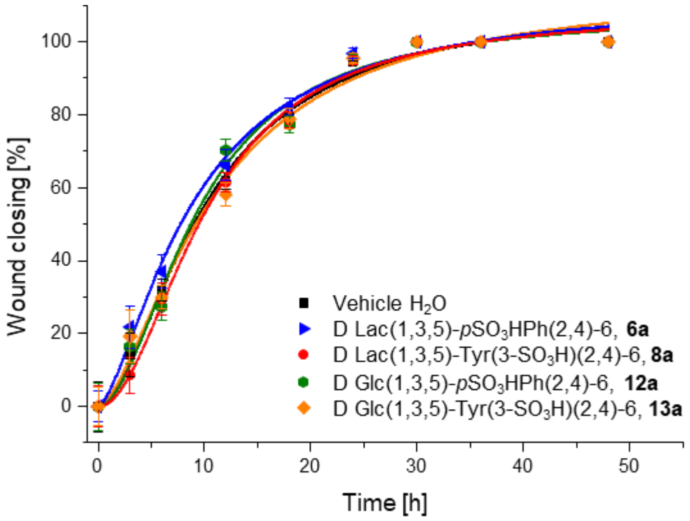

Figure S 135: Results of the migration of assays of HEK 293 treated with the vehicle control H_2O (black) and compounds **6a** (blue), **8a** (red), **12a** (green) and **13a** (orange) in a dosing experiment giving extra doses after 12,24,36 and 48 h.

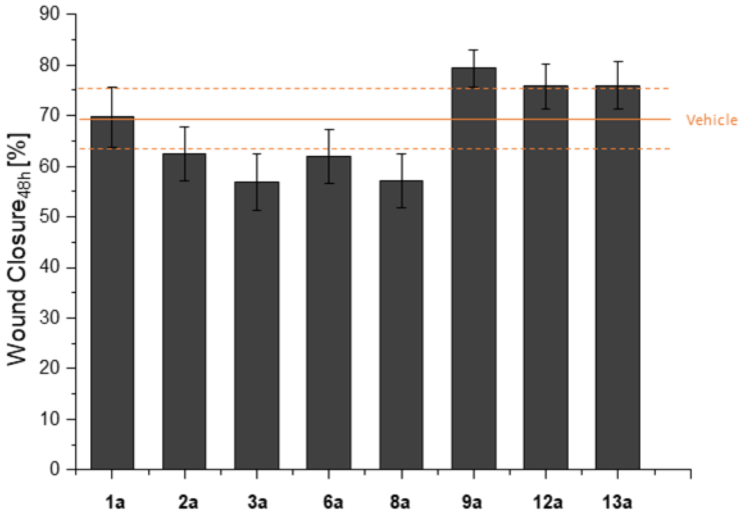

Figure S 136: Comparison of the woundc closure± SD [%] of the MCF 7 cells after 48 h. Vehicle control (orange line) and SD (dotted line).

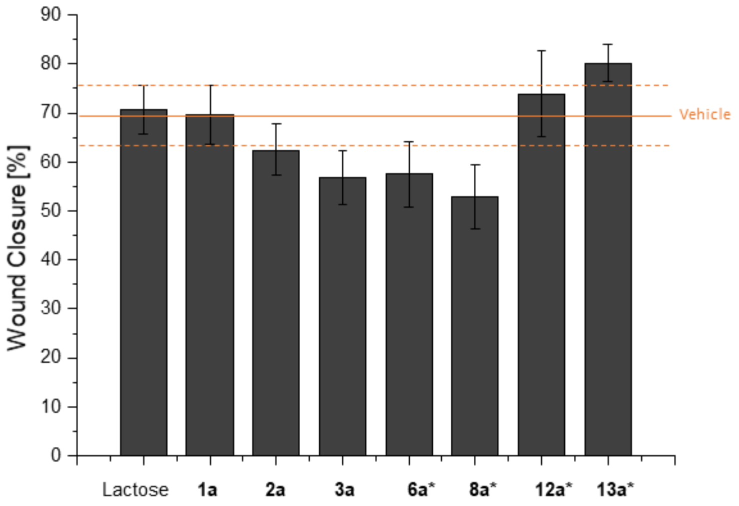

Figure S 137: Comparison of the woundc closure± SD [%] of the MCF 7 cells after 48 h. Vehicle control (orange line) and SD (dotted line). The results of the compounds marked with * are from dosing experiments.

Table S1. Percentage wound closure after 48 h for MCF 7 migration treated with glycoconjugates **1a-3a, 9a** and controls.

Glycoconjugate	Wound closure $_{48\,h}$ + SD [%] [a]
Unstained	69 ± 6
Vehicle Control	69 ± 6
Lactose	71 ± 5
Lac(1)-2, **1a**	70 ± 6
Lac(1,3,5)-6, **2a**	62 ± 5
Lac(1,2,3,4,5,6)-7, **3a**	57 ± 5
Glc(1,3,5)-6, **9a**	79 ± 4

[a] Wound closure with the standard deviation SD [%] was calculated refereeing the distance of the woundfield at time point 48 h to the woundfield at time point 0h. Woundfield distance at 0h was set to 0 % wound closure. Distance value for one timepoint is the average of 25 different distance measurements using an ImageJ software.

Table S2. Percentage wound closure after 48 h for MCF 7 migration treated with glycoconjugates **6a, 8a, 12a** and **13a.**

Glycoconjugates	Wound closure $_{48\,h}$ + SD [%] [a]
Vehicle Control	69 ± 6
Lac(1,3,5)-*p*SO_3HPh(2,4)-6, **6a**	62 ± 5
Lac(1,3,5)-Tyr(3-SO_3H)(2,4)-6, **8a**	57 ± 5
Glc(1,3,5)-*p*SO_3HPh(2,4)-6, **12a**	76 ± 4
Glc(1,3,5)-Tyr(3-SO_3H)(2,4)-6, **13a**	76 ± 5

[a] Wound closure with the standard deviation SD [%] was calculated refereeing the distance of the woundfield at time point 48 h to the woundfield at time point 0h. Woundfield distance at 0h was set to 0 % wound closure. Distance value for one timepoint is the average of 25 different distance measurements using an ImageJ software.

Table S3. Percentage wound closure after 48 h for MCF 7 migration treated with glycoconjugates **6a, 8a, 12a** and **13a** in dosing experiments, giving extra doses after **12, 24,36,48 h.**

Glycoconjugates	Wound closure $_{48\,h}$ + SD [%] [a]
Vehicle Control	69 ± 6
Dosing Lac(1,3,5)-*p*SO_3HPh(2,4)-6, **6a**	58 ± 7
Dosing Lac(1,3,5)-Tyr(3-SO_3H)(2,4)-6, **8a**	53 ± 7
Dosing Glc(1,3,5)-*p*SO_3HPh(2,4)-6, **12a**	74 ± 8
Dosing Glc(1,3,5)-Tyr(3-SO_3H)(2,4)-6, **13a**	80 ± 4

[a] Wound closure with the standard deviation SD [%] was calculated refereeing the distance of the woundfield at time point 48 h to the woundfield at time point 0h. Woundfield distance at 0h was set to 0 % wound closure. Distance value for one timepoint is the average of 25 different distance measurements using an ImageJ software.

Author Contributions

Tanja Freichel synthesized all building blocks and glycooligomers, performed the SPR measurements and cell studies, assisted with the ELISA measurements and drafted the article. Dominic Laaf designed and performed the ELISA measurements in collaboration with Tanja Freichel, Viktoria Heine, Ellie Mackintosh and Nicole L. Snyder. Sophia Sarafova assisted with the design of the flow cytometry assays. Lothar Elling provided analytical methods and assisted with the analysis of binding study data. Nicole L. Snyder and Laura Hartmann conceived the project, contributed to the analysis, discussion and interpretation of the data and finalized the article.
